# Tailored living mycelium macerate for reproducible flexible actuators

**DOI:** 10.1038/s41467-026-76623-z

**Published:** 2026-08-12

**Authors:** Rui Nie, Baoyuan Li, Luxuan Li, Guangyao Hou, Yikai Gao, Xiaonan Wang, Yan Huang, Mei Zhu, Xiaoman Liu, Xin Huang

**Affiliations:** 1 State Key Laboratory of Advanced Inorganic Fibers and Composites, School of Chemistry and Chemical Engineering, Harbin Institute of Technology, Harbin, 150001 P. R. China; 2 School of Materials Science and Engineering, Harbin Institute of Technology, Harbin, 150001 P. R. China; 3 State Key Laboratory of Urban Water Resource & Environment, School of Chemistry and Chemical Engineering, Harbin Institute of Technology, Harbin, 150001 P. R. China

**Keywords:** Polymers, Sustainability, Bioinspired materials

## Abstract

The integration of living attributes, such as self-renewal and responsiveness, into structural materials remains a fundamental challenge, as they typically preclude the mechanical robustness required for practical applications. Here, we report a fungal-based maceration tailored strategy to create living mycelium materials with exceptional mechanical properties and programmable humidity responsiveness. The developed materials demonstrate wide-range adjustable tensile performance, achieving 11 ~ 349% elongation and 0.1 ~ 18.0 MJ·m^−3^ toughness, the highest values reported among flexible mycelium materials. Through multiscale characterization from microstructural to molecular levels, we revealed the hygroscopic deformation mechanism of Janus mycelium macerates which arises from asymmetric hygromechanics and entropy-driven reorganization. Crucially, these materials retain their living functionalities, enabling reversible dormancy-regeneration cycles in 1 year and inheritable Janus structure along with self-renewal of performance. Moreover, we demonstrate their ability to convert environmental humidity fluctuations into quantifiable electrical signals, highlighting their potential as biohybrid environmental sensors and interactive platforms. This interdisciplinary study represents an integrative paradigm in living material design, and exhibits substantial potential for future sustainable and intelligent applications at the intersection of living mycelium materials and ecologically sustainable devices.

## Abstract (web summary)

Designing living materials is of interest for responsive materials, though it is challenging to optimize mechanical properties. Here the authors design a fungal based system for a robust humidity responsive material.

## Introduction

Growing concerns over environmental degradation and energy resource depletion have spurred innovations in sustainable materials^1,2^. Fungal biotechnology offers a promising bioeconomy solution^3^. In this regard, engineered living materials (ELMs) leverage living components for dynamic, responsive functions^4,5^. Unlike non-living materials, ELMs exhibit remarkable capabilities, including self-repair^6,7^, environmental adaptation^8–10^, and dynamic switching between material states^11,12^. Specially, mycelium-based biomaterials which showcase strong degradation capacity and high biomass production, have been widely adopted in material innovation^13–15^, owing to their well-developed mycelium network, which provide excellent bonding strength by using the well-established biomanufacturing techniques.

Pure mycelium materials due to the unique chain entanglement structure offer an excellent opportunity to develop substitutes of flexible synthetic materials, such as sustainable electronics from *Ganoderma lucidum*^16,17^. Integrating the concepts of living materials^18,19^ while preserving fungal cell viability led to the development of the concept of living mycelium materials^20,21^. Researchers exploited dormancy regeneration and self-healing ability of living fungi cells to impart these unique behaviors into nonliving materials^4^, yielding self-repairing living biocomposites^20,22^ and hydrophobic skin of robots^23^. The latest generated responsive living mycelium materials advanced applications of mycelium in sensors. Modifying living *Schizophyllum commune* mycelium with carbon nanotubes^24^ and graphene nanosheets^25^ provided piezoresistive response. Recent breakthroughs have demonstrated robotic control using electrophysiological signals from living *Pleurotus eryngii*^26^. However, despite these pioneering advances, existing mycelium-based materials remain limited by tunability of mechanical properties. Given the target application in rigid structural supports, where mechanical robustness and shape retention are prioritized over plastic deformation, this level of elongation is sufficient and does not pose a limitation. Conversely, for potential future applications requiring high flexibility, this property would need to be addressed. On-demand customization remains a critical challenge for widespread adoption.

With tailored mechanical properties, flexible mycelium materials could be designed to undertake the function of environmental interaction. Humidity regulation constitutes a critical component of environmental regulation, requiring materials capable of dynamic moisture exchange with their surroundings. Current humidity regulation methods rely on energy-intensive electromechanical systems^27^. While sustainable materials for example, lignocellulose aerogel^28^ and geopolymer^29^ offer eco-friendly humidity response, such tough materials lack of flexibility restricts intelligent humidity regulation. The humidity-responsive behavior observed in living *Schizophyllum commune* films by Sinha et al.^30^ provides promising inspiration. Despite lack of scenarios demonstration and mechanisms elucidation on hygroscopic deformation, the application potential of mycelium materials in humidity regulation was attractive, due to the humidity change visualization and moisture diffusion control achieved by deformation process, as well as ecological sustainability.

In this study, we leverage natural mushroom-like fungi to tailored a novel humidity responsive material. Liquid state fermentation (LSF) of selected mushroom species consistently produces mycelium membrane with intrinsic Janus characteristics^31,32^: an air-exposed hydrophobic top layer and a medium-contacting hydrophilic bottom layer. To overcome the inherent brittleness of dry mycelium, we applied a PVA network and glycerol to enhance flexibility and moisture absorption, establishing the concept of “macerate” that preserves cell viability and the Janus structure. By maceration engineering that manipulated the inherent physical property of polysaccharide-dominated fungal networks, we achieved programmable hygroscopic deformation through engineered structural asymmetry. Our findings reveal that the Janus-type deformation arises from molecular-level heterogeneity in both hygromechanics and entropy-driven reorganization. The resulting material system exhibits tunable hydromechanical properties, improved desiccation tolerance, and enhanced hygroscopic deformation capabilities. Based on sustainable dormant living material^33^, we propose a fabrication-service-regeneration-recycle scheme. Overall, this study contributes an alternative strategy to achieve modulation on Janus structure of living mycelium materials and advance their practical application in urgent demand of reproducible and sustainable sensing materials.

## Results

### Fabrication and modulation of Janus mycelium macerates

**Fig. 1:**
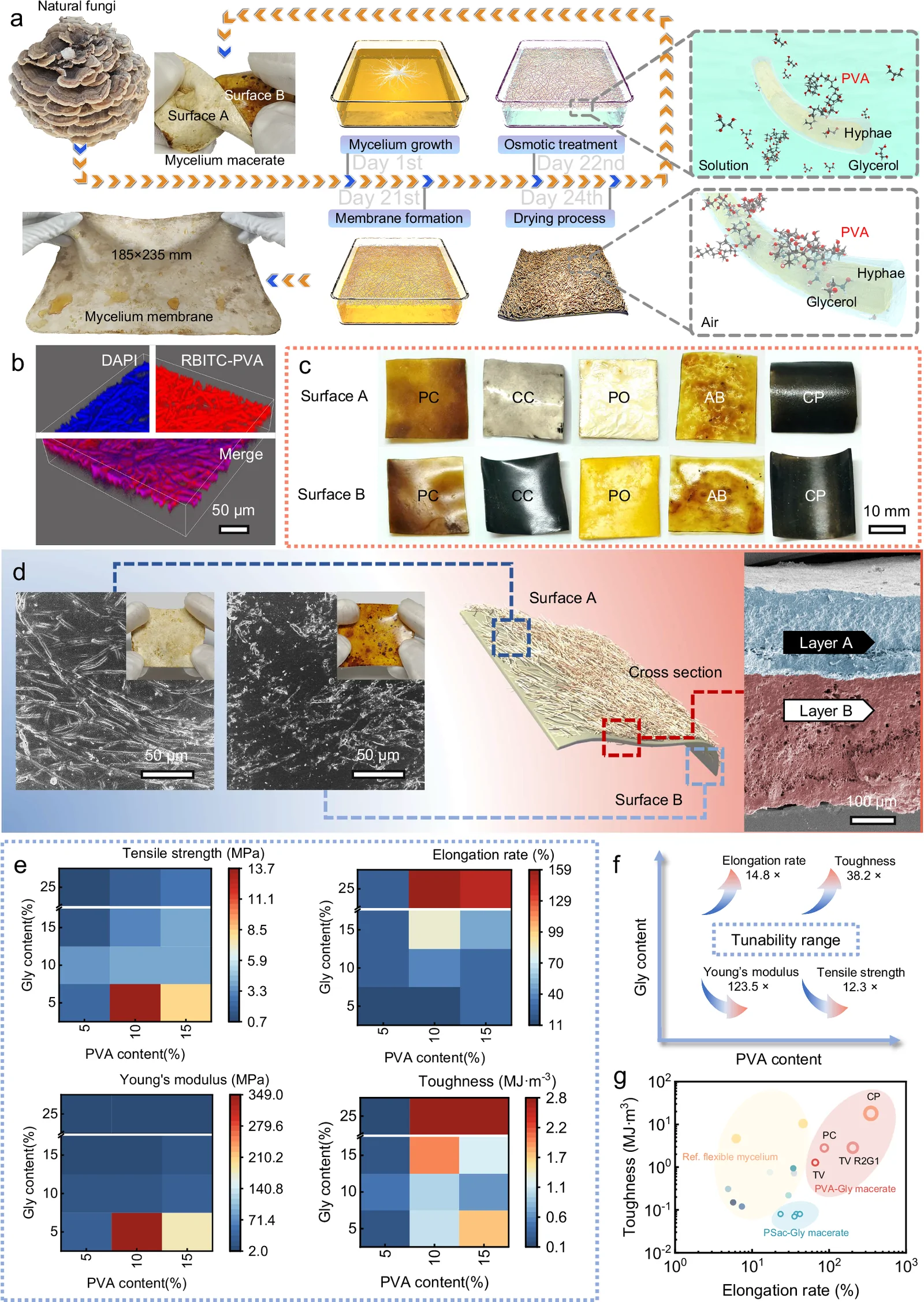
Fabrication and characterization of Janus mycelium macerates. **a** Schematic drawing of liquid state fermentation to preparing Janus mycelium macerates, four steps from mycelium growth, membrane formation, osmotic treatment to drying process. Insets respectively indicate the process of PVA and glycerol permeating into hyphae during osmotic treatment, as well as the concept of macerate that PVA and glycerol are sequestered by hyphae after dehydration. Insets: Common wild Trametes species in Harbin Institute of Technology. Native mycelium membrane of *Trametes versicolor* via liquid state fermentation in scale of 185 × 235 mm. Janus mycelium macerates showcasing top surface A, bottom surface B and flexibility to bend at will. **b** 3D confocal images of DAPI dyed TV@(RBITC-PVA)-Gly section. **c** Optical images from top (surface A) and bottom (surface B) of Janus mycelium macerates fabricated from *Phanerochaete chrysosporium* (PC), *Copyinds comatus* (CC), *Pleurotus ostreatus* (PO), *Agaricus bisporus* (AB), and *Cladophialophora* sp. (CP). **d** Schematic illustration of the Janus TV@P-G structure and SEM images of the top surface (A), bottom surface (B), and cross-section. An internal key within the cross-sectional SEM image defines the layer assignments (A and B). The photographs shown as insets display the top surface (A) and bottom surface (B), respectively. Data are representative of three independent replicates (*n* = 3) with similar results. **e** Heat maps displaying correlation between mechanical properties (tensile strength, elongation rate, Young’s modulus and toughness) and PVA, glycerol content. **f** Summary illustrating the tunability range of mechanical property parameters tailored by maceration engineering. **g** Comparison of elongation rate and thoughness for TV, PC, CP 10%PVA + 10%Gly and other flexible mycelium-based material recently reported in the literature.

To ensure biological safety, we selected *Trametes versicolor* (TV) as the mycelium source due to its well-documented cultivability^32,34^ and robust growth characteristics (Supplementary Fig. 1a, b). The fabrication process comprises four key steps (Fig. 1a): (1) mycelium growth, (2) membrane formation (rate-limiting step), (3) osmo tic treatment and (4) drying process. Crucially, during osmotic treatment (Step 3), PVA and glycerol molecules infiltrate the mycelium network, enhancing the toughness and flexibility of dehydrated mycelium membrane. By scaling up the cultivation system to a container size of 185 × 235 mm, we successfully produced full-size TV membranes. After drying, the mycelium membrane transformed into a flexibly, leather like material capable of free bending while retaining its Janus structure and for clarity, we designate the top surface as A and the bottom surface as B. To determine the optimal cultivation time of *Trametes versicolor*, the entire formation process of native mycelium membrane on the gas-liquid interface was monitored (Supplementary Fig. 1c) and its growth kinetics was achieved (Supplementary Fig. 1d). A remarkable growth and dry weight increase started from day 4, whereas the growth cessation occurring on day 10, due to limitation of nutrition and containers. Taking solid content (Supplementary Fig. 1e), mechanical property in fresh state (Supplementary Fig. 2a) and cultivation period into comprehensive consideration, we selected 20 days as the optimal cultivation period for mushroom specie TV. Fresh native mycelium membrane maintained >90% water content regardless of culture time/treatment (Supplementary Fig. 2b). For air application, all samples were ambient-dried. The dried membrane without glycerol was brittle, highlighting the necessity for a plasticizer to reduce biopolymer interactions. Glycerol enabled mycelium macerate, TV@P-G to achieve high flexibility (bending: Supplementary Fig. 3a and 3b; curling: Supplementary Fig. 3c). PVA provided strong physical crosslinking, with hyphae-PVA co-localization confirmed by confocal microscopy (DAPI-stained hyphae, RBITC-labeled PVA; Supplementary Fig. 4 and Fig. 1b), indicating PVA recruitment by fungal hyphae.

Specially, such developed approach also offers significant advantages in terms of both universal applicability and scalability for large-scale production. Likewise, other mushroom fungi species including *Phanerochaete chrysosporium* BKM-F-1767 (PC), *Coprinus comatus* (CC), *Pleurotus ostreatus* (PO), *Agaricus bisporus* (AB) and even black yeast-like fungi (*Cladophialophora* sp., CP) were utilized to obtain the corresponding robust and Janus mycelium macerates (Supplementary Fig. 5 and Fig. 1c). The Janus structure of mycelium membrane is well reserved after maceration treatment. Difference in surface and cross section micromorphology of TV@P-G was observed by SEM (Fig. 1d). Viewed from cross section, the top layer corresponds to aerial hyphae that grows horizontally over the medium surface (layer A), whereas the bottom layer consists of nutritive hyphae growing inward medium (layer B). By altering the cultivation time, the Janus structure of TV@P-G was apparent in optical and SEM images (Supplementary Fig. 6). Based on Janus structure reservation, the maceration treatment we mentioned could further enhanced toughness of mycelium membrane.

Superior mechanical performance represents a critical prerequisite for the successful implementation of mycelium-based materials in practical applications. Using the above established fabrication protocol, we prepared mycelium macerates incorporating 0-10% PVA and 5–25% glycerol, then systematically evaluated their mechanical performance through uniaxial tensile testing (Supplementary Figs. 7–9, Supplementary Table 1 and 2). We systematically characterized and compared the mechanical properties of various mycelium macerates, including tensile strength (*TS*), elongation rate (*EG*), Young’s modulus (*E*) and toughness (*T*). Heat maps (Fig. 1e) were subsequently generated to visualize the correlation between each performance parameter and the content of PVA and glycerol. Based on the above results, we made a trade-off between *TS* and *T*. Furthermore, an appropriate *E* is crucial for actuators, because a high *E* value will impede the deformation of actuators under stimulation, whereas materials with low *E* easily generate bending moment due to dead weight. Therefore, mycelium membrane treated with 10% PVA and 10% glycerol (TV 10%PVA + 10%Gly) was chosen as optimized model sample in the following research, with proper *E* (23.7 MPa), enough *EG* (36%) and *T* (1.0 MJ·m^−3^) values (Supplementary Fig. 10). Additionally, we defined the tunability range that referred to the degree of freedom of our method to modulate the mechanical properties of materials, intuitively quantifying the regulatory potential (Fig. 1f). We also compared our method with reported flexible mycelium-based materials in mechanical properties (Supplementary Table 3 and Fig. 1g), especially, from which the CP 10%PVA + 10%Gly with *EG* of 350% and *T* of 18.0 MJ·m^−3^ is outstanding (Supplementary Fig. 11). Strengthening mechanism of PVA and plasticizing mechanism of glycerol were both elucidated in our supplementary discussion (Supplementary Fig. 12-17), which can be summarized that PVA enhances the hydrogen bond network among biomacromolecules, while glycerol promotes sliding of macromolecular chains.

Based on the integral strengthening of mycelium macerate, mechanical property differences between Janus structure were investigated. Interestingly, layer B broke later than layer A during tensile tests (Supplementary Fig. 18a–d). Additionally, Janus TV@P-G could be separated along the interface between layer A and B. Tensile tests of separated layer A and B (Supplementary Fig. 18e) further confirmed that *TS*, *EG* and *T* of layer B were higher than layer A, whereas *E* of layer A was larger. The asymmetrical modulus is a basics of inner stress and deformation.

### Programmable humidity response of Janus-structured mycelium macerates

**Fig. 2:**
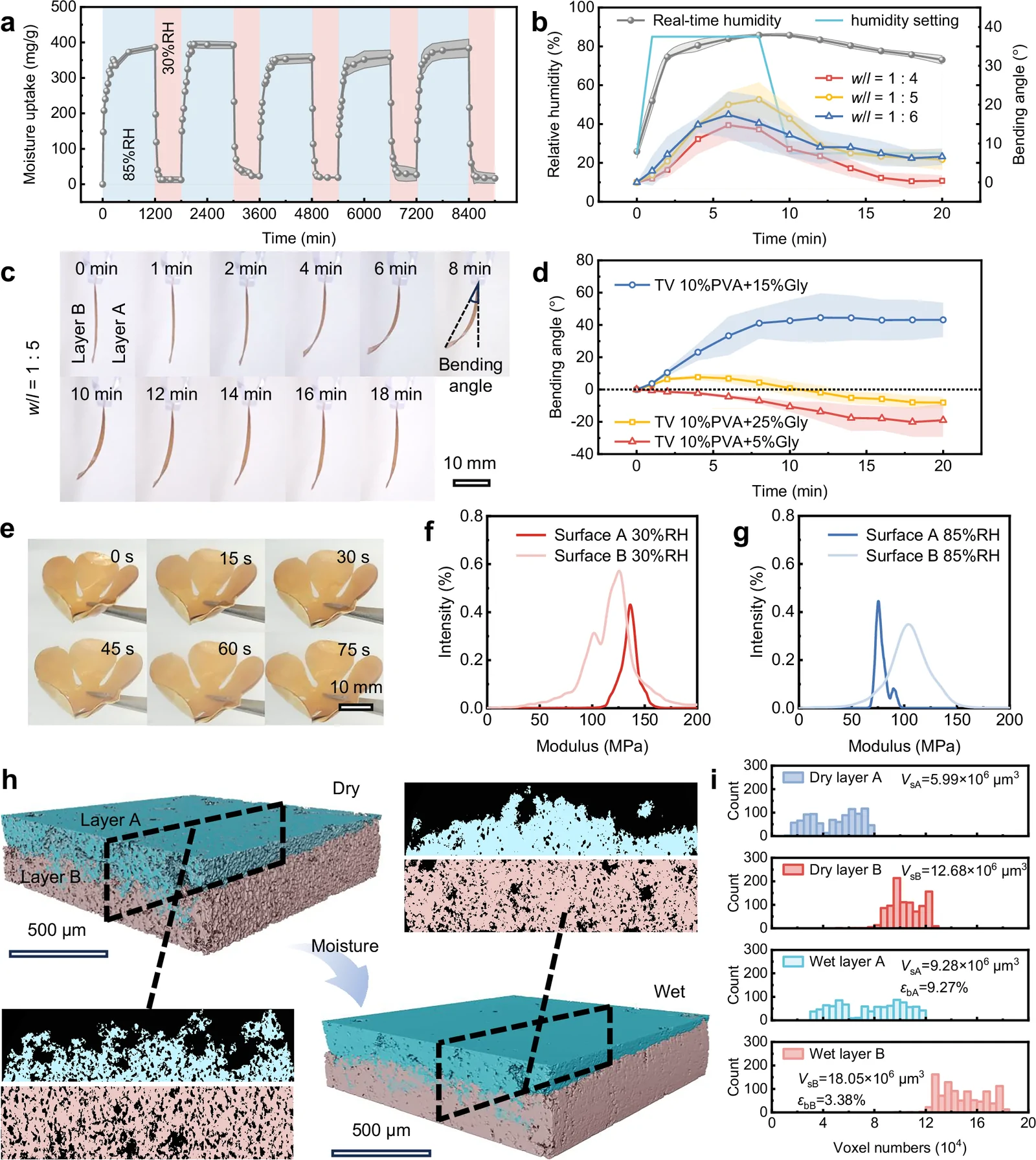
Humidity response behaviors of Janus mycelium macerates. **a** Dynamic vapor sorption (DVS) curve of Janus TV@P-G in 5 cycles (25°C, 85% RH), *n* = 3, with moisture uptake presented as mean values ± SD. **b** Bending angle changes of Janus TV@P-G (10%PVA + 10%Gly) specimen with different width to length ratios (*w*/*l*) in single fixed humidity response experiments. Data are presented as mean values ± SD, *n* = 3 per group. Humidity was controlled with humidity chamber. **c** Optical images of TV@P-G specimen (5 × 25 mm) with single fixed end deforming in hygroscopic process controlled in a humidity chamber, where humidity changed from 20% RH to 85% RH in 8 min, and decreased in the next 12 min. **d** Bending angle changes of Janus TV@P-G treated with different Gly content (5%, 15% and 25%Gly), conducted in humidity chamber. Data are presented as mean values ± SD, *n* = 3 per group. The bending angle is defined as illustration. **e** Optical images of opening Janus TV@P-G flower with humidity response. Difference between DMT modulus distribution of Janus TV@P-G upon exposure to a dry environment (R.T., 30% RH) (**f**) and wet environment (R.T., 85% RH) (**g**), quantifying by AFM. **h** 3D CT images of TV@P-G skeleton structure and 2D binarised images of cross section in dry (R.T., 30%RH) and wet state (R.T., 85%RH) using the Otsu method. Blue and red part represent layer A and B, respectively. **i** Frame-wise statistics for voxel numbers of layer A and B skeleton structure in dry and wet states. Skeleton volume (*V*_s_) and bulk moisture expansion rate (*ε*_b_) were calculated. Error bars (mean ± SD) in (**b** and **d**) were connected and visualized as a shadow effect around the data points.

Given the critical role of Janus structure in hygroscopic deformation, we systematically evaluated the humidity responsive performance of the mycelium macerates, exhibiting excellent reversible hygroscopicity with an average moisture uptake of 374.9 mg·g^−1^ (25 °C, 85% RH) in 5 consecutive cycles (Fig. 2a). Moreover, sorption kinetics analysis confirmed glycerol’s dominant role in moisture absorption (Supplementary Fig. 19). Density measurements via Archimedes method (Supplementary Fig. 20) revealed significant structural changes, showing a 7.94% density reduction.

To be of great interest, due to the interior stress between layer A and B, untethered Janus mycelium macerates showed spontaneous bending towards layer B from fresh to dry state. While, under humidity exposure in chamber (Supplementary Fig. 21), the Janus mycelium macerates exhibited reversible self-directed deformation toward layer A accompanied by progressive curvature reduction by increasing width-length ratios (Supplementary Fig. 22). By contrast, constrained deformation tests with single end constrained revealed consistent bending orientation towards layer B across various width-length ratios (Fig. 2b, c, Supplementary Fig. 23) and Gly contents (Supplementary Fig. 24 and Fig. 2d). The cyclic humidity response (Supplementary Fig. 25) of mycelium macerate demonstrates that its deformation under high humidity conditions is reversible. Therefore, based on above observations, a bioinspired flower-shaped actuator could be achieved which demonstrated programmable humidity-responsive opening behavior (Fig. 2e), highlighting the material’s potential for biomimetic applications, although the mycelium macerates could not recover as short as increasing humidity induced deformation, due to the unequal moisture desorption and adsorption rates.

In this section, we demonstrate a humidity‑responsive material whose equilibrium bending angle can be programmed by tailoring geometric parameters and composition. The angle varies reversibly with relative humidity, and a library of bending angles, (−19.0 ± 10.3)° to (43.2 ± 10.7)° was achieved through structural design.

### Mechanism of hygroscopic deformation

**Fig. 3:**
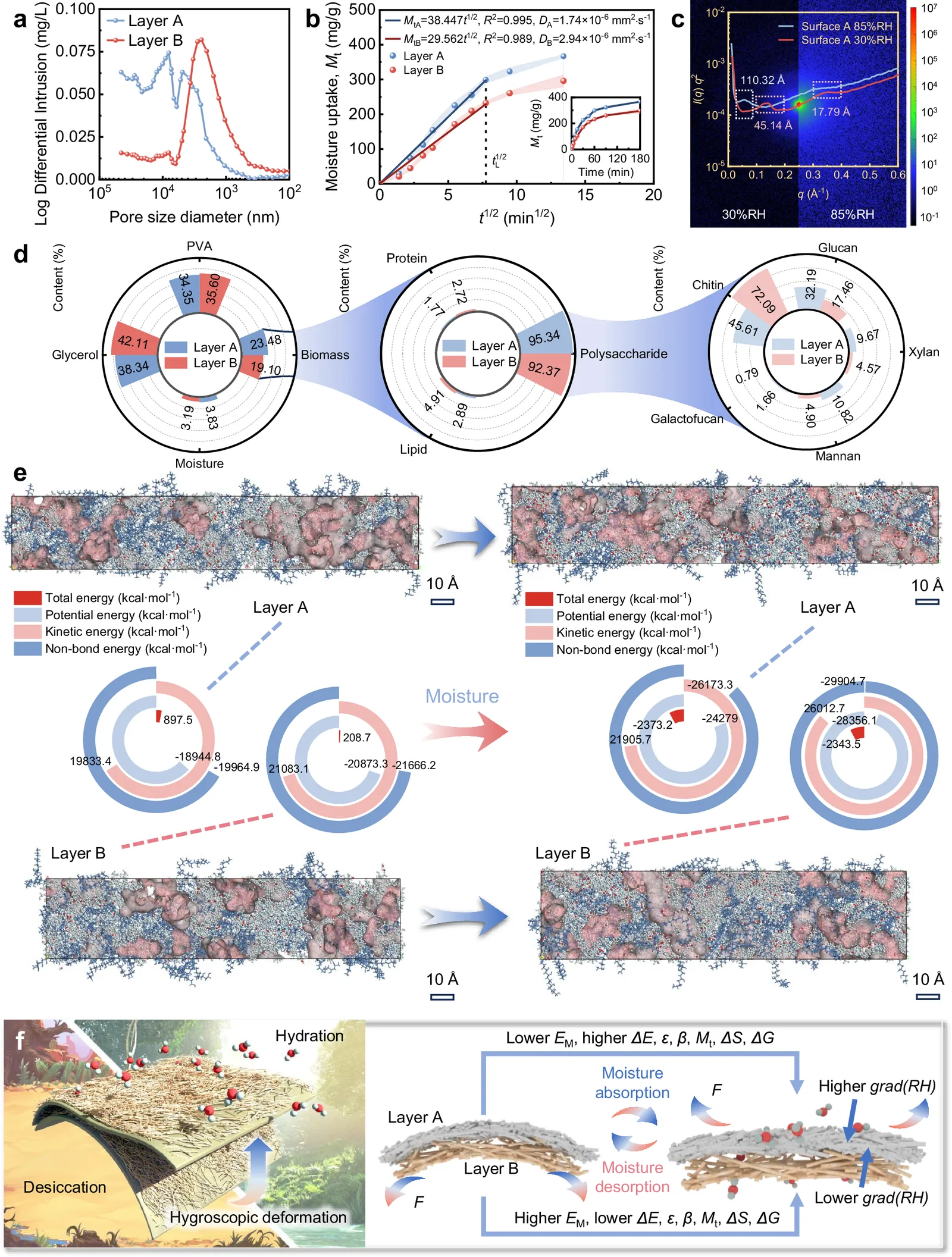
Mechanism investigation on hygroscopic deformation of Janus mycelium macerates. **a** Mercury intrusion pore size distribution curves of layer A and B at dry state. **b** Moisture absorption kinetics curves of layer A and B, *n* = 3. Error bars (mean ± SD) were connected and visualized as a shadow effect around the data points. **c** 2D SAXS pattern and Lorentz-corrected 1D SAXS curves as inset of surface A in dry (R.T., 30% RH) and wet (R.T., 85% RH) environment. **d** Composition and proportion of separated layer A and B in three levels, respectively, material, biomass and polysaccharide. **e** MD simulation for layer A and B consist of 5 kinds of polysaccharide in fungal cell wall, including glucan, galactofucan, xylan, mannan and chitin, interacting with PVA and glycerol before and after 5 min moisture absorption upon exposure to a wet (R.T., 85% RH) environment. Ring bar charts represent total energy, potential energy, kinetic energy and non-bond energy of each model. **f** Schematic illustration of multi-level hygroscopic deformation mechanism.

The hygroscopic deformation of Janus mycelium macerates arises from differential skeletal expansion between layers, driven by asymmetry hygromechanical responses. Sensitivity differentiation of Janus layers in response to moisture (Supplementary Fig. 26 and Supplementary Movie 1), showed layer A close to high humidity exhibited larger bending angles. The Derjaguin-Müller-Toporov (DMT) modulus analysis revealed surface A exhibited a 336.16% greater reduction in modulus and a 22.7 MPa lower moisture modulus (*E*_M_) than surface B (Supplementary Fig. 27, Fig. 2f, g), indicating its greater susceptibility to humidity-induced deformation^35,36^. Necessarily, layer A and B were separated from the entire Janus mycelium macerates to facilitate investigation on expansion rates, pore structure, hygroscopicity, periodicity and molecular composition. Isolated layers A and B showed no statistically significant difference in planar expansion rates (Supplementary Fig. 28), prompting micro-CT examination of the intact macerate during hydration (Fig. 2h). Bulk moisture expansion rate (*ε*_b_) was higher for layer A (9.27%) than layer B (3.38%) (Fig. 2i), revealing that layer A was dominant in hygroscopic deformation. Dry-state porosity was 3.75% higher in layer A (37.31 ± 2.16% vs. 32.42 ± 1.33%, Supplementary Fig. 29). To complement micro-CT data and further resolve sub-micron structure, mercury intrusion porosimetry (MIP) (Fig. 3a and Supplementary Fig. 30 and 31) revealed distinct pore architectures: layer A contains abundant fractal penetrating pores that enhance, while layer B exhibits higher pore surface area and tortuosity, indicative of a denser skeleton with interconnected porosity. Moisture adsorption induced pore compression through skeletal expansion, reducing porosity in both layers (A: *P* = 9.38 ± 1.03%; B: *P* = 7.02 ± 1.05%) (Supplementary Fig. 32) and homogenizing pore morphology, as evidenced by shifts in diameter distribution and sphericity (Supplementary Figs. 33 and 34). Therefore, hygroscopic deformation stems primarily from differential skeletal expansion rather than pore structure alterations.

Further research on hygroscopic deformation mechanism demands separation A from B (Supplementary Fig. 35) and respective characterization. Next, to elucidate microstructure-moisture relationships, dynamic vapor sorption (DVS) analysis revealed that layer A exhibits 1.24-fold greater saturated moisture uptake and faster adsorption kinetics than layer B (Fig. 3b), consistent with its higher porosity. Coefficient of moisture expansion (*β*), as an inherent property was also determined, which reflected that layer A exhibited a larger volume expansion under moisture uptake per unit (Supplementary Fig. 36a). Moreover, moisture diffusion coefficients (*D*), calculated following literature methods^37^, showed an inverse relationship - layer B displayed 1.70-fold faster moisture diffusion than layer A. This apparent paradox suggests layer A maintains a steeper moisture gradient (*grad(RH)*) during adsorption. Both layers exhibited similar critical relative humidity (*CRH* ≈ 60%; Supplementary Fig. 36b), implying that the enhanced adsorption and expansion in layer A likely arise from its ability to sustain higher moisture gradients at the *CRH* threshold.

Based on small angle X-ray scattering (SAXS) results (Fig. 3c, Supplementary Fig. 37), layer A displayed a more periodic microstructure than B in dry state. After moisture absorption, the scattering peak of biomolecules (phospholipid bilayer, 45.14 Å and helix polysaccharides, 17.79 Å) weakened and a scattering peak corresponding to 110.32 Å appeared, indicating hydration-induced disruption of native periodicity and formation of hydrated structures. Additionally, Porod fitting (Supplementary Fig. 38a) demonstrated that the interfaces between phases became more distinct. Increase of fractal dimension (*α*) (Supplementary Fig. 38b) indicated a more complex skeleton fractal caused by moisture absorption. These observations also support a hydration mechanism where water incorporation increases system entropy, with differential entropy changes between layers driving the observed hygroscopic deformations.

Quantification of PVA and glycerol distribution in the macerates (Supplementary Figs. 39–41) revealed that glycerol preferentially localized in layer B, while PVA distribution between layers varied with glycerol content (0–25%). Notably, all glycerol concentrations (5%, 10%, 15% and 25%) induced bending in the same direction, demonstrating that deformation direction is independent of glycerol distribution. Although glycerol distribution did not govern the hygroscopic deformation mechanism, we found that the relative proportions of PVA, glycerol, and mycelium biomass - both in the overall macerate and within individual layers - serve as key tunable parameters for controlling the magnitude and dynamics of hygroscopic deformation and recovery.

Furthermore, to elucidate the relationship between hygroscopicity difference and molecular composition, we also quantified each component proportion in three levels of hierarchy, including material, biomass and polysaccharide (Fig. 3d), where total lipid, protein and polysaccharide in biomass, as well as chitin, glucan, mannan, xylan and galactofucan in polysaccharide (Supplementary Figs. 42–44) were all quantified. According to these results, molecular dynamics (MD) simulations (Supplementary Fig. 45-51) of each layer before and after moisture absorption were adopted, respectively. It is important to note that this model represents a simplified view of the cell wall. The calculated interaction energies reflect the local chemical environment, but do not account for the long-range physical constraints imposed by the global network architecture. For both layer A and B, the total energy (*e*) decreased from desiccation to dampness (Fig. 3e), illustrating thermodynamically spontaneous moisture absorption and hydration. Layer A exhibited a *Δe* that was 1.28 times greater than that of layer B, further indicating greater Gibbs free energy (*ΔG*) change, consistent with entropy increase. Moreover, electrostatic interaction energy (Supplementary Fig. 52) and numbers of hydrogen bond (Supplementary Fig. 53) between biomacromolecules and glycerol/PVA reduced after moisture absorption, due to the competition of water molecules. In particular, enhancement of interaction energy (van der Waals in *E*_int_) and increasing numbers of hydrogen bond between biomacromolecules and glycerol in layer B demonstrated a lower volume expansion, also reflected by a more concentrated radial distribution function (*RDF*) and increasing coordination number (Supplementary Fig. 54). Simulated mean square displacement (*MSD*) (Supplementary Fig. 55) and X-ray scattering (Supplementary Fig. 56), respectively evidenced the lower *D* of moisture and more significant disordering in layer A, consistent with aforementioned results.

Overall, the multifactorial mechanism of Janus mycelium macerate’s hygroscopic deformation was summarized (Fig. 3f): hygromechanics difference between Janus architecture directly induced hygroscopic deformation, due to the larger modulus change (*ΔE*), lower *E*_M_, higher *ε* and *β* of layer A; the higher *M* and *grad(RH)* of layer A confirmed by diffusion kinetic, as well as its higher *ΔS* derived from disordering transform of intermolecular periodic structures acted as the actuating force; further molecular-level mechanistic analysis elucidated that the asymmetrical composition of molecules fundamentally induced asymmetry in macromolecule-moisture interactions and *ΔG* (before and after moisture adsorption) within the Janus mycelium architecture.

### Application of responsiveness in humidity regulation

**Fig. 4:**
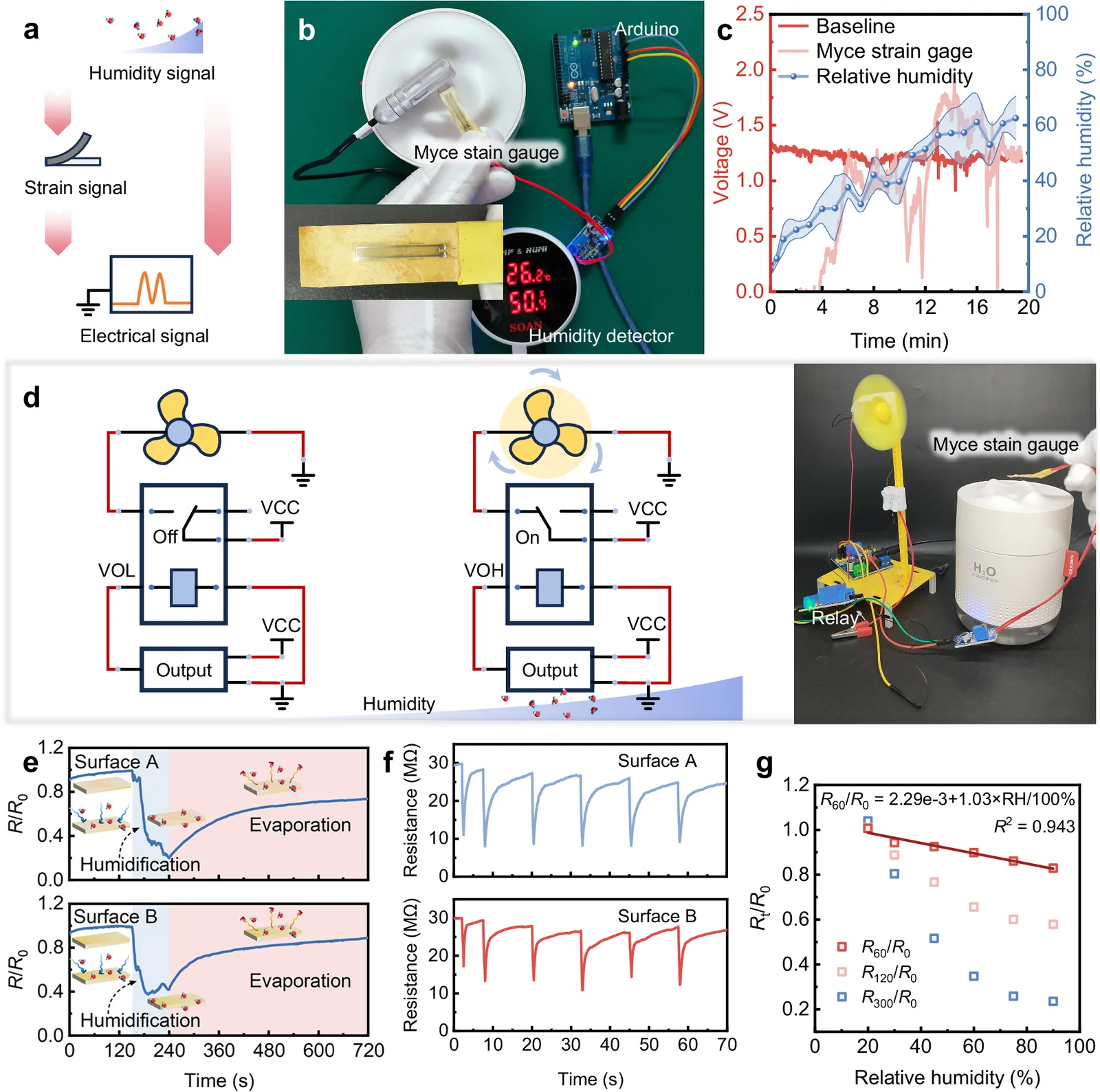
Janus mycelium macerate applied to sensing. **a** Schematic diagram of mycelium macerate converting humidity signals into other signals, used to summarize the research content in this section. **b** Myce strain gauge in collaboration with a humidity probe and Arduino for humidity and voltage signal acquisition, simultaneously. Inset: photograph of Myce strain gauge (Janus mycelium macerate attached with strain gauge). **c** Myce strain gauge output with humidity increasing. Baseline was obtained by a single strain gauge without Janus mycelium macerate. For real-time humidity recorded, three parallel experiments were conducted. Error bars (mean ± SD) were connected and visualized as a shadow effect around the data points. **d** Application of Myce strain gauge in collaboration with a relay to control circuit as a humidity response switch. Schematic illustration and photograph demonstrate that Myce strain gauge switched on the fan under high humidity. **e** Response and recovery curve of Janus mycelium macerate stimulated from surface A and B. **f** Resistance response stability in dry-wet cycles of Janus mycelium macerate from surface A and B. **g** Time dependent response of Janus mycelium macerate in a constant humidity chamber set from 20% RH to 90% RH.

To further demonstrate an interdisciplinary application scenario of the Janus mycelium macerate, two signal conversion paths were designed (Fig. 4a). First, by utilizing the function of strain gauge to transform strain into electrical signal, hygroscopic deformation of the Janus mycelium macerates can be developed as the bridge from humidity to strain. A Myce strain gauge was fabricated by affixing a commercial strain gauge onto the Janus mycelium macerates. A custom-programmed Arduino microcontroller was employed to acquire real-time electrical signals from the sensor module, while a humidity probe concurrently monitored ambient humidity near the Myce strain gauge (Fig. 4b). Due to difference in modulus between the strain gauge and TV@P-G, interface stress occurred and decreased the original output voltage. However, as humidity increasing and moisture adsorption, the voltage output exceeded baseline, indicating an effective transformation from humidity to electrical signal via strain of hygroscopic deformation (Fig. 4c). To demonstrate practical functionality, we integrated the Myce strain gauge into a relay-controlled circuit, enabling switchable activation of an output device (Supplementary Fig. 57). When the environment humidity exceeded the critical value (~60%), consistent with DVS data, the output voltage reached the relay’s activation threshold (~1.7 V), and the fan in output circuit was switched on (Fig. 4d). Notably, when the hygroscopically deformed Myce strain gauge was exposed to airflow from the activated fan, dehydration-induced shape recovery occurred, thereby interrupting the circuit and turning off the fan as a self-regulating feedback (Supplementary Movie 2), which as a proof-of-concept study, validates the Janus mycelium macerate as an effective humidity-responsive sensor with potential applications in environmental monitoring and automated control systems.

In addition to humidity-to-voltage transduction through stress-mediated sensing, the Janus mycelium macerates exhibited significant humidity-dependent resistance changes. Real-time resistance monitoring was performed using a Keithley 2400 source meter unit (SMU), revealing an immediate resistance decrease upon moisture adsorption (Supplementary Fig. 58). Then, to investigate the Janus structure’s influence, we characterized the asymmetric resistance response by exposing surfaces A and B to humidity gradients independently. Surface A demonstrated a 25.51% greater resistance reduction amplitude compared to surface B (Fig. 4e), consistent with its higher moisture adsorption capacity established in previous studies. This difference was further evidenced during cyclic humidity testing, where surface A-stimulated samples maintained lower resistance peaks (Fig. 4f). Control experiments confirmed the humidity-specific nature of this response: mechanical compression (finger pressure) produced <45% of the humidity-induced resistance change (Supplementary Fig. 59). Therefore, humidity response on resistance of the Janus mycelium macerate could be considered to be effective.

**Fig. 5:**
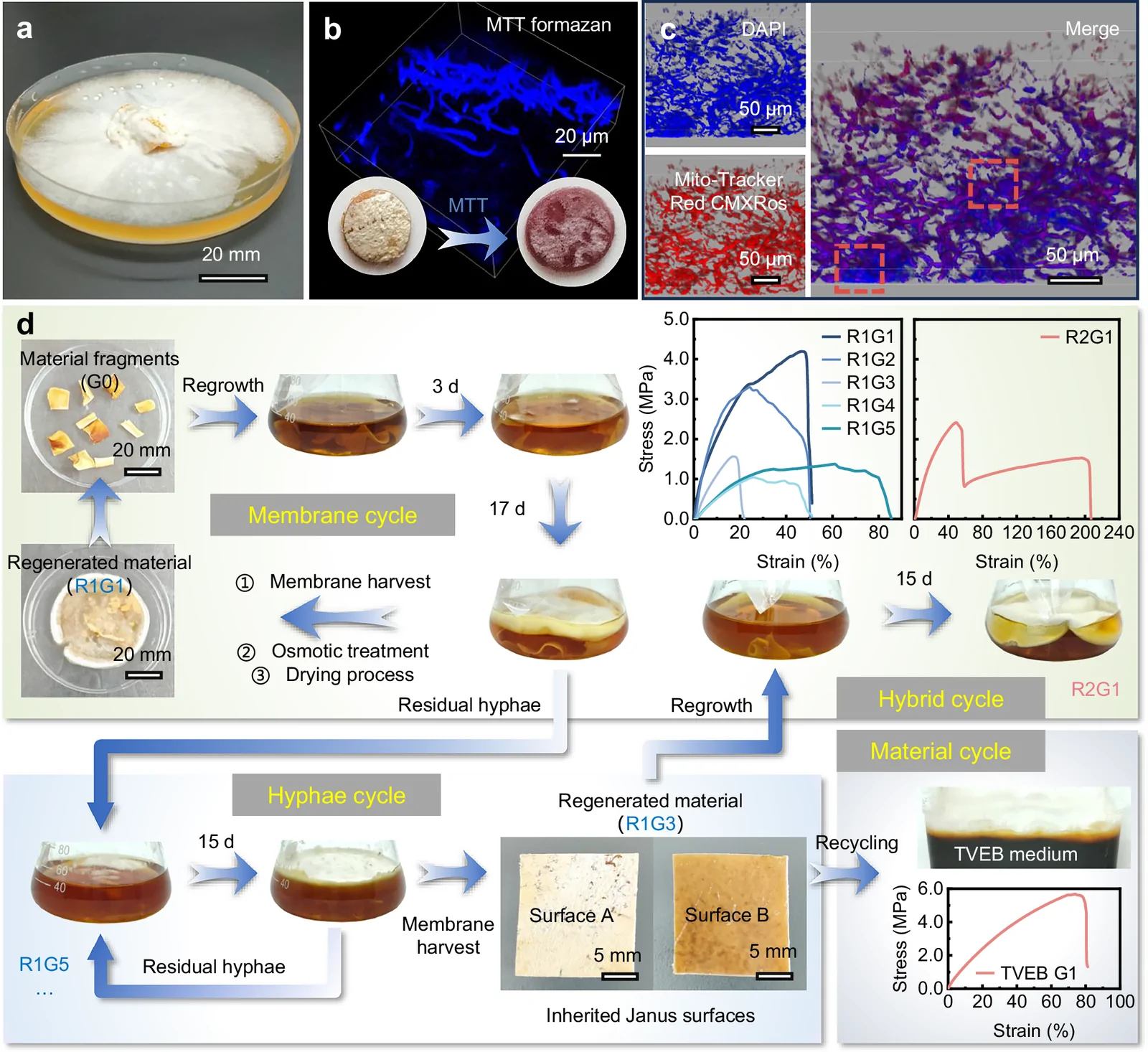
Conceptual presentation of Janus mycelium macerates as dormant living material. **a** Regeneration of TV@P-G hibernating for 3 months under desiccation state. **b** Photograph of MTT treated TV@P-G. **c** 3D reconstruction of confocal images of TV@P-G section stained with DAPI and Mito-Tracker Red CMXRos. Colocalization of fungal structures (hyphae and spores) with intact mitochondria indicates viable mycelium; living spores are outlined with frame lines. (Color assignments are defined by the in-image key.). **d** Four regeneration pathways of mycelium macerates. Membrane cycle: regrowth process of TV@P-G fragment in MEB liquid medium. New mycelium membrane regrew and matured on gas-liquid interface. Hyphae cycle: residual hyphae regrew and form mycelium membrane repeatedly. Hybrid cycle: regeneration from regrew mycelium macerates through membrane cycle and hyphae cycle. Material cycle: recycling mycelium macerates into culture medium to generate new mycelium membrane. “R” and “G” referred to “Recycling” and “Generation”. Insets, stress-strain curves of regrowth mycelium macerates (rectangular specimen with a width of 10 mm, a total length of 100 mm, and initial grip separation of 80 mm) through different regeneration pathways, and optical images of Janus surfaces of regenerated mycelium macerate.

Accordingly, we systematically quantified the humidity-resistance relationship using controlled humidity chamber (20 ~ 90% RH, and humidification lasted for 300 s). Selected from resistance response curves of each group (Supplementary Fig. 60a), resistance change rates (*R*_t_/*R*_0_) of the Janus mycelium macerate after humidifying for 60, 120, and 300 s were plotted with relative humidity (Fig. 5g), where *R*_60_/*R*_0_ exhibited a linear correlation with relative humidity (R^2^ = 0.943). In high humidity environments, R_t_/R_0_ signal of the Janus mycelium macerate deviated from linearity, which was still reflected in continuous humidification (Supplementary Fig. 60b). Combined with hygroscopic deformation, radius of curvature of the Janus mycelium macerate reflected humidity and resistance changes (Supplementary Fig. 60c and Supplementary Movie 3), a response that differs from that of conventional static humidity sensors.

Unlike conventional humidity sensors that aim for fast response (seconds or less) to enable real-time monitoring, our device operates on a minute-scale timescale by design. This slow actuation and recovery provide an intrinsic low-pass filtering effect: short-term humidity spikes are effectively suppressed, while sustained high-humidity events (minutes to hours) are integrated and retained as a measurable deformation. Accordingly, our approach is well-suited for applications requiring long-term exposure rather than real-time control.

### Organism-like sustainability derived from livingness

The fabricated Janus mycelium macerate represents a representative paradigm in dormant-type living materials that combines long-term cellular viability with functional performance. This innovative material system maintains fungal cells in a metabolically inactive yet viable state during operation, while preserving their biological responsiveness to environmental stimuli. To verify the cell viability under desiccated conditions, we employed a comprehensive staining approach (Supplementary Fig. 61). Initial propidium iodide (PI) staining revealed membrane damage in both hyphae cells and spores (Supplementary Fig. 62), yet remarkably, fungal cells in mycelium macerate maintained viability through 3-month desiccation and regenerative capacity upon rehydration (Fig. 5a). Metabolic activity was confirmed through Thiazolyl Blue (MTT) assays, where the Janus mycelium macerate developed characteristic purple formazan deposits (Fig. 5b). The linear correlation between formazan yield and sample mass (Supplementary Fig. 63), proving that the reduction of MTT was attributed to fungal cells in Janus mycelium macerates. This was further supported by hyphae-formazan colocalization (Supplementary Fig. 64). Considering that the reduction of MTT generally occurs in living mitochondrion, catalyzed by reductase, specific Mito-Tracker Red CMXRos was applied to labeled mitochondrion of living hyphae cells. Contrast with fresh mycelium, hyphae cells in the Janus mycelium macerate were also colocalized by Mito-Tracker Red CMXRos (Supplementary Fig. 65 and Fig. 5c). These findings collectively demonstrate that fungal cells in Janus mycelium macerates maintain functional mitochondria with intact enzyme activity as well as membrane potential persists despite desiccation, which satisfies key criteria for classification as a dormant living system.

Although fungal cells in hibernation state showed insignificantly reduced free radical scavenging capacity (Supplementary Fig. 66) concomitant with decreased mannitol content (Supplementary Fig. 67), exogenous glycerol supplementation effectively compensated for this protective function, enabling cell survival during desiccation through a mechanism analogous to endogenous mannitol protection. Therefore, after transferred into nutrient medium, fungal cells could regenerate and bridge the crack of cut mycelium macerate (Supplementary Fig. 68).

Moreover, similar with fabrication processes, fragments of TV@P-G could also undergo regrowth and maturation, forming a newborn mycelium membrane (Fig. 5d) that was then treated with PVA and glycerol, as well as drying process. Thus, regrew mycelium macerate (recorded as R1G1, where “R” and “G” referring to “Recycling” and “Generation”, respectively) was obtained and surprisingly performed increasing mechanical properties, compared with original specimen (G0). On the other hand, residual regrown hyphae could be unceasingly cultivated into R1G2 and R1G3, etc. The Janus structure was observed to be inherited by R1G3, of which performance is consistent with the behavior of living organism. From R1G4, mycelium macerates recovered *EG* and transferred into a more flexible form. Although mechanical performance gradually declined with successive generations (evident in R1G3), we could still recycle R1G3 as strain to generate R2G1, which performed over 200% of *EG*. This study highlights the sustainability of living mycelium materials, with the specificality of dormancy, regeneration, toughening and inheritance. Moreover, used macerate samples outside of sterility for 1 year could even be recycled on bacterial resistance medium to generate TV (Supplementary Fig. 69) and could be made into bio-based culture medium (TVEB) to cultivate new mycelium membranes. This regeneration process was mainly achieved by dormant spores, and the livingness of mycelium macerates could be quantified and compared through the radial growth rate (Supplementary Fig. 70). To maintain the livingness of spores in macerates, the dry storage period should be limited within 1 year. Remarkably, TVEB-cultivated macerate exhibited superior mechanical properties compared to conventional counterparts, which might suggest that iterative recycling may further enhance material performance through evolutionary adaptation of cell wall composition across generations. These cascading regeneration pathways collectively demonstrate the inherent sustainability and livingness of this material system, offering an opportunity for circular material design.

Additionally, life cycle assessment demonstrates that the carbon footprint of PVA‑modified mycelium materials could be reduced through optimized PVA loading and bio‑based alternatives for sustainability (Supplementary Figs. 71–73, Supplementary Table 11). For instance, natural polysaccharides (PSac), including carboxymethyl cellulose, polysaccharides from *Trametes*, pullulan and pectin could be adopted to replace PVA as bonding agent yet with a compromised mechanical performance (Supplementary Figs. 74 and 75). Adoption of bio-based alternatives to PVA still demand our further exploration on flexibly customizing the mechanical properties and responsiveness of mycelium materials.

## Discussion

Although living mycelium materials have been designed with multifunction, there still remained room for improvement performance of mechanical flexibility and sustainability. In this study, we develop a strategy to construct stretchable living mycelium macerates and establish a paradigm for integration of living mycelium-based materials with humidity regulation, paving the way for next-generation smart materials with sustainable solutions. The system leverages the natural Janus architecture of native mycelium membranes, which arises from distinct molecular compositions between aerial and nutritive hyphae (primarily chitin, glucans, mannans, and xylans in fungal cell walls). In facile and mild maceration engineering, rigid mycelium matrix in Janus structure was packed with high molecular weight PVA as bonding agent and small molecule glycerol as plasticizer and humectant. Asymmetrical molecular composition results in heterogeneous intermolecular interaction between biomacromolecules, PVA and glycerol, for the Janus structure of mycelium, where the proportion of each component is influenced. Therefore, different physical properties, involving Young’s modulus, moisture absorption rate, coefficient of moisture expansion and entropy change occur and induce Janus mycelium macerates hygroscopic deformation behavior. Sufficient mechanical properties and livingness make Janus mycelium macerates stand out from other sustainable moisture-response materials, which then allowed the possibility to achieve the construction of humidity-responsive sensor with potential applications in environmental monitoring and automated control systems. As an emerging class of environmental responsive material with livingness and sustainability, overcoming the material waste and recycling problem of conventional actuators, it can not only act like seeds to regenerate new mycelium membrane, but also be upcycled as raw materials of culture medium with better mechanical properties.

This study not only contributes a methodology to advance living material design but also establishes a sustainable platform for next-generation ecologically robust living materials. Especially given the vast diversity of fungal species in nature, by deliberately select the fungi, tailored Janus mycelium macerate could be created. Such as, in addition to TV, we incorporated other mushroom-forming fungi to produce Janus composites. Comparative analysis of five distinct fungal species revealed variations in cross-sectional microstructure (Supplementary Figs. 76 and 77) and hygroscopic response behavior. Despite undergoing identical processing conditions, these macerates exhibited divergent deformation patterns under humidity exposure (Supplementary Fig. 78). Notably, PO@P-G displayed hygroscopic deformation opposite to that of TV@P-G, while CC@P-G exhibited a complex deformation response. The opposite deformation direction across species is a key support for the “tailorable” claim. We interpret these differences primarily as a result of species-specific Janus structural asymmetry (Supplementary Figs. 79 and 80, Supplementary text of discussion). These findings suggest that species-dependent structural and molecular composition differences in layer A/B critically influence hygroscopic performance. By strategically selecting fungal species and processing parameters, Janus mycelium macerates with programmable deformation behaviors can be engineered and tailored, advancing the development of sustainable fungal-based materials for further applications in different scenarios. Moreover, such dormant type living material enables the realization of “bio-welding”, which then open up opportunities for modular construction of Janus structures to extend the structure diversity. For instance, through turn-over growth, a newborn layer A_2_ would generate upon layer B, and a sandwich-like A_1_BA_2_ structure could be realized (Supplementary Fig. 81). This structural versatility, combined with programmable deformation, would position fungal-based living materials as a versatile platform for adaptive intelligent systems. Therefore, by unifying ecological sustainability with tunable functionality, such studied work could enable opportunities for engineered living materials—from environmentally responsive sensors to various modular structures, and such fusion of biological specificity with materials engineering principles could support the development of biologically integrated innovative application scenarios.

## Methods

### Materials

The following chemicals were used as received: glucose (Glc, Oxoid), potato extract (AOBOX), agar (Biosharp), malt extract broth (Shanghai Bio-way technology Co., Ltd.), ammonium nitrate (NH_4_NO_3_, Sigma-Aldrich), calcium chloride (CaCl_2_, Aladdin), potassium phosphate dibasic (K_2_HPO_4_, Aladdin), ferric trichloride (FeCl_3_, Aladdin), potassium dihydrogen phosphate (KH_2_PO_4_, Aladdin), yeast extract (Oxoid), magnesium sulfate heptahydrate (MgSO_4_·7H_2_O, Aladdin), sodium chloride (NaCl, Aladdin), Mowiol® PVA-117 (Mw ~145,000, Aladdin), glycerol (Biosharp), rhodamine B isothiocyanate (RBITC, Sigma-Aldrich), DMSO (Energy Chemical), 4’,6-diamidino-2-phenylindole (DAPI, ThermoFisher), isononyl isononanoate (Macklin), methyl tert-butyl ether (MBTE, Aladdin), methanol (Aladdin), urea (Aladdin), sodium carbonate (Aladdin), dithiothreitol (DTT, Aladdin), BCA protein quantitative kit (APPLYGEN), Fucose (Fuc), rhamnose (Rha), arabinose (Ara), galactose (Gal), xylose (Xyl), mannose (Man), fructose (Fru), ribose (Rib), galacturonic acid (GalA), glucuronic acid (GlcA), N-acetylgalactosamine (GalN) hydrochloride, N-acetylglucosamine (GlcN) hydrochloride, guluronic acid (GulA), and mannosamine (ManA) purchased from BoRui Saccharide Biotech Co. Ltd., trifluoroacetic acid (TFA, ACROS), Sodium hydroxide solution (50%, Alfa Aesar), sodium acetate (ThermoFisher), dextranase from *Chaetomium erraticum* (Sigma-Aldrich), fluorescein diacetate (FDA, Aladdin), propidium iodide (PI, Aladdin), 3-(4,5-dimethylthiazol-2-yl)−2,5-diphenyltetrazolium bromide (Thiazolyl Blue, MTT, Aladdin) and Mito-Tracker Red CMXRos (Beyotime), DPPH Free Radical Scavenging Capacity Assay Kit (BIOESN), ampicillin (Aladdin), kanamycin sulfate (Aladdin), carboxymethyl cellulose (M.W. 700000, DS = 0.9, 2500–4500 mPa·s, Macklin), pectin (65%, from apple, Macklin), pullulan (Sigma-Aldrich).

### Fungi species

*Trametes versicolor* and *Phanerochaete chrysosporium* BKM-F-1767 were purchased from Shanghai Microbiological Culture Collection Co.,Ltd. *Coprinus comatus* (CC), *Pleurotus ostreatus* (PO), *Agaricus bisporus* (AB) were purchased from Zhou Yulin Edible Fungus Research institute, Hongshan District, Wuhan. *Cladophialophora* sp. was separated from wild *Trametes versicolor* in Harbin Institute of Technology.

### Preparation of growth medium

Solid PDA medium was prepared by mixing 20 g glucose, 10 g potato extract and 15 g agar, fixed volume with deionized water to 1 L. After sterilized at 121 °C for 15 min, pour into plates. 1 L Liquid MEB medium contained 5 g malt extract broth, 1 g NH_4_NO_3_, 0.1 g CaCl_2_, 0.5 g K_2_HPO_4_, 0.02 g FeCl_3_, 0.5 g KH_2_PO_4_, 0.02 g yeast extract, 0.5 g magnesium sulfate heptahydrate and 1 g NaCl. After sterilized at 121 °C for 15 min, store at 8 °C.

### Mycelium culture

Fungi strains recovered on PDA plates were transferred into MEB liquid medium, cultivated in a stationary state. The liquid state fermentation was kept at 28 °C and 85% relative humidity for 20 days when the native mycelium membranes matured.

### Dry weight and solid content measurements

We harvested the native mycelium membranes and washed with sterile water. The fresh weight and dry weight after lyophilization were recorded as *m(*fresh*)* and *m(*dry*)*. Solid content (*S*) and water content (*W*) of native mycelium membranes could be deduced respectively:

1 $$S\,=\,\frac{m({{\rm{dry}}})}{m({{\rm{fresh}}})}\times 100\%$$

2 $${W}=\,\left(1-\frac{m({{{\rm{dry}}}})}{m({{{\rm{dry}}}})}\right)\times 100\%$$

### Preparation of Janus mycelium-based material

PVA-117 and glycerol were dissolved in deionized water at 60 °C and sterilized at 121 °C for 20 min. Drying the surface with filter paper, native mycelium membranes were submerged into PVA and glycerol mixture solution for 24 h. It took 2 days for macerates to dry in a sterile environment, after wiping away surface solution.

### Mechanical tensile tests

Uniaxial tensile tests were conducted utilizing a universal mechanical testing machine (SUNS UTM2102) equipped with a 100 N-capacity load cell. The tests were performed by applying a constant displacement rate of 5 mm·min^−1^. Following ASTM D882-18 standard, sample dimensions were normalized in width of 10 mm, initial grip separation of 80 mm, total length of 100 mm. These rectangular shape specimens were prepared by a mold cutter. The thickness of specimens was measured by a vernier caliper. The test speed was set as 5 mm/min, and environmental conditions stayed in 23 ± 2 °C, 30 ± 5% RH. The toughness (*T*, MJ·m^−3^) was calculated by integrating the area under stress-strain curves.

3 $$\,{T}={\int }_{\!\!\!\!\!0}^{{EG}}\sigma {{{\rm{d\varepsilon}}}}$$

Here, *EG* is fracture elongation rate; *σ* and *ε* are nominal stress and strain respectively.

### Fluorescent labeling of PVA

2 g PVA-117 and 50 μL triethylamine was dissolved in 50 mL anhydrous DMSO at 60  °C. 100 μL RBITC stock solution (1 mg·mL^−1^ in acetone) was added after PVA completely dissolved. The reaction lasted 10 h protecting from light and then was transferred into dialysis bags with molecular weight cut-off of 35 kDa. The purified RBITC-PVA was lyophilized and collected.

### Optical and confocal microscopy of macerates

We used two double-sided blades to prepare macerates section for microscopy imaging. The optical images of material sections were observed with an inverted microscope (Leica DMI8). RBITC-PVA was adopted at osmotic treatment process to show PVA distribution in macerates. DAPI staining macerates section was carried out in a concentration of 10 μg·mL^−1^. The confocal images were collected using a laser confocal microscope (Leica TCS SP8). Excitation wavelength at 405 nm and 552 nm were selected, with a fluorescence detector for wavelengths from 440 nm to 640 nm. By setting a z position range, series of 2D images were scanned for 3D reconstruction, which was conducted using image analysis software (LAS X).

### Scanning electron microscopy

Mycelium membranes and macerates were tread with liquid nitrogen and lyophilized. Surface and cross-section micromorphology of mycelium membranes and macerates were imaged with a scanning electron microscope (Hitachi SU8010). Samples underwent gold spraying were observed under an acceleration voltage of 10 kV.

### Fourier transform infrared spectra

After dried in an oven at 140 °C, KBr was grinded and pressed into tablets as background. Lyophilized native and PVA treated mycelium membranes were mixed with KBr in the mass ratio of 1:100. A Fourier transform infrared spectrometer (PerkinElmer Spectrum Two) was employed to collect infrared spectra and 2D correlation spectra. Wavenumber ranges were set from 500 to 4500 cm^−1^.

### Density functional theory calculation

Polymer chains including PVA, chitin, and glucan were simplified into fragment to calculate electron density, electrostatics and population analysis. Water, glycerol, PVA, chitin and glucan were geometry optimized in Fine quality, where generalized gradient approximation (GGA) and BLYP functional were adopted. In electronic settings, integration accuracy and SCF tolerance were set as Fine. Core treatment applied DFT Semi-core Pseudopots (DSPP). Basis set was DNP and basis file was 3.5. In energy task, parameters and functional were same with geometric optimization. Electrostatic potential (ESP) mapping was realized by population analysis with electron density and potentials imported.

To calculate interaction energy (*E*_int_) between Water, glycerol, PVA, simplified chitin and glucan, respectively, models of two molecules interacting were processed with geometry optimized, where SCF was set 1.0e^−6^ of SCF tolerance with multipolar expansion as Hexadecapole. For density mixing, charge value was 0.04 and spin value was 0.1. Global orbital cutoff was set as 5.2 Å. When calculating properties of population analysis in energy task, GGA and PBE functional were adopted, and integration accuracy, SCF tolerance were set as Fine. DSPP was applied as core treatment. Basis set was DNP and basis file was 3.5.

4 $${E}_{{{\mathrm{int}}},{{\rm{A}}}-{{\rm{B}}}}\,={E}_{{{\rm{AB}}}}-(E{{\rm{A}}}+{E}_{{{\rm{B}}}})$$

Here, E_AB_ refers to total energy of A and B complex, *E*_A_ or *E*_B_ refer to individual energy of A or B.

### Thermogravimetry tests

Thermogravimetry curves of mycelium samples were measured with a thermogravimetric analyzer (HENVEN HCT-). Samples were cut into small pieces and weighed (3 ~ 5 mg). Transferred into Al_2_O_3_ crucibles (100 mg), the sample was heated from 25 to 800 °C in a heating rate of 10 °C·min^−1^, with a holding time of 15 min at 800 °C.

### Component proportion tests

The initial weight of all TV 10%PVA + 10%Gly samples were recorded as *m*_*0*_. According to the disparity of water dissolution temperature between PVA and glycerol, the samples were submerged into deionized water at R.T. for 3 h twice in order to remove glycerol. After dried at 60 °C until dehydration, weigh the dried samples as *m*_1_. Similarly, to dissolve PVA effectively, samples removed glycerol were submerged into deionized water at 80 °C with stirring for 6 h twice. The final dried samples were weighed as *m*_2_. Therefore, the proportion (*P*) of mycelium biomass, glycerol and PVA were respectively calculated as:

5 $$P({{\rm{mycelium}}})={m}_{2}/{m}_{0}\times 100\%$$

6 $$P({{\rm{glycerol}}})=({m}_{0}-{m}_{1})/{m}_{0}\times 100\%$$

7 $$P({PVA})=({m}_{1}-{m}_{2}) / {m}_{0}\times 100\%$$

### Dynamic vapor sorption and sorption kinetics tests

All mycelium-based material samples were dehydrated in a vacuum drying oven at 80 °C for 10 h as pretreatment. For dynamic vapor sorption tests, temperature of a constant temperature and humidity chamber (model: UV-U3, voltage: 220 V, flow: 10-20 L/min, power: 24 W, vacuum: −90 kPa, YOUCHENGINDUSTRIALCO.LTD.) was kept at 25 °C, with humidity varied from 10 to 90% RH, where the weight of dehydrated and hydrated samples was recorded. Humidity increased by 10% after sorption equilibrium maintained for 60 min. Sorption kinetics curves were collected by recording the weight of samples at a constant temperature 25 °C and humidity 85% RH from dehydration to sorption equilibrium. And then transfer the samples to a dry environment (25 °C, 20% RH) for desorption kinetics tests. These experiments were repeated independently more than three times with similar results.

### Atomic force microscope

TV 10%PVA + 10%Gly samples were treated with dehydration at 80°C in vacuum for 10 h and hydration at 25 °C, 85% RH for 1 h, respectively. Height and DMT modulus images were scanned with an atomic force microscope (Bruker Dimension® Icon™). Probes for dry surface A, dry surface B, wet surface A, and wet surface B were selected as RTESPA-525, RTESPA-300, RTESPA-150-30, and RTESPA-300-30, respectively. Modulus was analyzed with NanoScope software.

### Density measurements

Inspired by Archimedes method, we selected isononyl isononanoate to show the volume of TV 10%PVA + 10%Gly. Because isononyl isononanoate is not miscible with both water and glycerol, additionally, of which density is smaller than water and glycerol. Isononyl isononanoate was added in a precision measuring cylinder with a division value of 25 μL. The volume of isononyl isononanoate was recorded as *V*_0_. Dry samples weighed in *m*_1_ were cut into pieces and completely submerged into isononyl isononanoate, raising the liquid level to *V*’. Thus, the volume of dry samples (*V*_1_) was determined:

6 $${V}_{1}=V^{\prime}-{V}_{0}$$

Treat dry samples weighed in *m*_1_’ with moisture at 85% RH for 2 h, obtaining wet samples in *m*_2_. Moisture rate (*w*) of TV 10%PVA + 10%Gly was calculated as:

7 $$w=({m}_{2}-{{m}_{1}}^{\prime})/{{m}_{1}}^{\prime}\times 100\%$$

The liquid level raised to *V*’’ after wet samples were completely submerged. Similarly, the volume of dry samples (*V*_2_) was determined:

8 $${V}_{2}=V^{{\prime}{\prime}{\prime}}-{V}_{0}$$

Varying the volume of dry and wet samples, series of data were collected. By plotting and linear fitting, the slopes of fitting line corresponded to density (*ρ*_1_, *ρ*_2_) of dry and wet TV 10%PVA + 10%Gly samples, respectively.

9 $${\rho }_{1}=\frac{{{{\rm{d}}}}{m}_{1}}{{{{\rm{d}}}}{V}_{1}}$$

10 $${\rho }_{2}=\frac{{{{\rm{d}}}}{m}_{2}}{{{{\rm{d}}}}{V}_{2}}$$

Furthermore, the expansion volume (Δ*V*) of samples from dehydration to hydration was calculated as:

11 $$\Delta V={V}_{2}-{V}_{1}$$

Therefore, the expansion rate could be deduced as:

11 $$\frac {\Delta {V}}{{V}_{1}}=(\frac{(1+w){\rho }_{1}}{{\rho }_{2}}-1)\times 100\%$$

### Dynamic vapor sorption

Samples of TV 10%PVA + 10%Gly as well as separated layer A and B were treated in vacuum at 80 °C for 24 h to remove moisture, then the dehydrated samples were weighed as *m*_0_. After moisture adsorption for *t*, the weight of samples was measured as *m*_t_. Therefore, moisture uptake (*M*_t_) was calculated.

12 $${M}_{{{\rm{t}}}}=({m}_{{{\rm{t}}}}-{m}_{0})/{m}_{0}$$

DVS curves when exposure to a constant humidity of 85% RH (*M*_t_-*t*) and increased humidity (*RH*) from 10% RH to 90% RH (*M*_t_-*RH*) were respectively collected. In the constant humidity, *M*_t_ was also plotted with *t*^1/2^, here, *M*_t_ and *t*^1/2^ kept a linear relation when *t* ≤ *t*_L_^37^.

13 $${M}_{{{\rm{t}}}}=\frac{4{M}_{{{\rm{m}}}}}{h{\pi }^{1/2}}\sqrt{{Dt}}$$

*M*_m_ is the moisture uptake in saturation, *h* is thickness of samples, *D* is diffusion coefficient (*D*) of moisture. Therefore, *D* could be calculated according to the slope when *t* ≤ *t*_L_. For layer A and B, average thickness was measured as 0.22 and 0.30 mm, respectively.

14 $$D=\pi {\left(\frac{h}{4{M}_{{{\rm{m}}}}}\right)}^{2}{{{\rm{Slope}}}}^{2}$$

### Small angle X-ray scattering

SAXS was carried out using a SAXS instrument (Xenocs Xuess3.0). Before testing, TV 10%PVA + 10%Gly was cut into 2 × 2 cm and treated with desiccation and humidity respectively. By altering the surface to be tested, SAXS data corresponded to surface A and B were collected.

### Mycelium layers separation

A scalpel was used to separated TV@R-G macerate along the interface between layer A and B (Supplementary Fig. 39a). We used a freezing microtome (Leica CM1950) to peel off a frozen monolayer from the mycelium membrane, which had been embedded in optimal cutting temperature (OCT) compound, (Tissue-Tek®), a mixture of polyethylene glycol (PEG) and PVA. By utilizing the microtome’s thickness adjustment function and referencing our SEM images of mycelium cross-sections, we were able to control the thickness of the peeled layer from the entire membrane. The step distance for sampling was set to 50 μm. To eliminate interfacial effects, the interface region was peeled off together with monolayers A and B, while the pure layers B/A were preserved, as shown in the new Supplementary Fig. 35b and c. After removing the OCT compound from the remaining embedded B/A layer, we conducted ingredient testing for lipids, proteins, and polysaccharides.

### Mercury intrusion porosimetry

MicroActive AutoPore V 9600 was employed to carry out mercury intrusion tests of separated layer A and B. Pressure ranged from 0.4 to 30,000 psia and mercury temperature was 20.98 °C. According to Washburn’s equation, where *γ* refers to the surface tension of mercury (485 dynes/cm), *θ* refers to the contact angle (130°) between mercury and the sample surface, *P* is the applied pressure (psia) and *r* is pore diameter.

15 $${Pr}=-2\gamma \cos \theta$$

Pore structure parameters were analyzed by AutoPore V 9600 software, including total pore area, median pore diameter, average pore diameter, bulk density, skeletal density, porosity, tortuosity factor, tortuosity, percolation fractal dimension and backbone fractal dimension.

### Micro-CT

X-ray microscopy (ZEISS Xradia 515 Versa) was employed to conduct tomography of in situ moisture adsorption for Janus mycelium macerate at 0.5 μm resolution. The sample was kept at 30% RH, 25 °C when scanned as dry state. Then we used a box to maintain local humidity at approximately 85% RH to perform in situ moisture adsorption and tomography. Slice thickness was 500 μm in average and scanning consumed 4 h. Thresholding method was adopted to distinguish skeleton and pore structure of mycelium macerate. Pores were extracted from μCT data via the Pore Network Modeling (PNM) extension of Avizo.

### Total lipid contents measurements

Separated layer A and B were grinded in liquid nitrogen and then lyophilized. The initial weights of lyophilized samples were recorded as *m*_A0_ and *m*_B0_. Treat the samples with methyl tert-butyl ether and methanol (volume ratio of MBTE: methanol: water = 10: 2: 5) in ultrasonic bath for 6 h. Centrifuge at 3000 rpm for 15 min to separate phases. The upper MTBE phase was removed and the lower water phase containing the samples was transferred to a new EP tube and dried. Weigh the defatted samples and record the mass as *m*_A1_ and *m*_B1_. Therefore, total lipid contents (*P*_LA_, *P*_LB_) were calculated as:

16 $${P}_{{{\rm{LA}}}({{\rm{LB}}})}=\frac{{m}_{{{\rm{A}}}0({{\rm{B}}}0)}-{m}_{{{\rm{A}}}1({{\rm{B}}}1)}}{{m}_{{{\rm{A}}}0({{\rm{B}}}0)}}\times 100\%$$

### Total protein contents measurements

Extracting solution for protein was prepared containing 8 M urea and 0.2 M Na_2_CO_3_. Grinded in liquid nitrogen and lyophilized samples of layer A and B weighed *m’*_A0_ and *m’*_B0_ were treated with extracting solution and 65 mM DTT. The system was kept in ice bath and oscillated in a shaker with 70 rpm for 1 h. Centrifuge with 11,000 rpm at 4 °C for 20 min and transfer the supernatant to determine protein concentrations with BCA protein quantitative kit (APPLYGEN). Reacting at 25 °C for 2 h, measure the absorbance at 562 nm (*A*_562_) of supernatant and BCA working solution mixture (1: 50) with an ultraviolet-visible spectrophotometer (Eppendorf BioSpectrometer basic). In addition, BSA standard curve (*A*_562_ = 0.1457+*c*×9.911e–4, *R*^2^ = 0.9955) was measured, where *c* is mass concentration (μg·mL^−1^) of BSA standard solutions. Total protein contents (*P*_P_) of samples could be deduced as:

17 $${P}_{{{{\rm{P}}}}}=\frac{10000\times \left({A}_{562}-0.1457\right)\times {V}_{S}}{9.911\times {m}_{0}^{{\prime} }}\times 100\%$$

Here, *m’*_0_ is initial weight of lyophilized samples; *V*_*S*_ is volume of supernatant reacted with BCA.

### Ion chromatogram

15 monosaccharide standards (fucose, rhamnose, arabinose, galactose, glucose, xylose, mannose, fructose, ribose, galacturonic acid, glucuronic acid, galactosamine hydrochloride, glucosamine hydrochloride, guluronic acid, and mannuronic acid) were individually weighed at 5 mg each and placed into ampoules containing 2 mL of 3 M trifluoroacetic acid (TFA). Hydrolysis was performed at 120 °C for 3 h. The acid hydrolysates were accurately transferred to a nitrogen-drying apparatus, followed by the addition of 5 mL of water, vortex mixing, and preparation of the standard stock solution. The concentration of each monosaccharide in the mixed standard solution was determined using the absolute quantification method, and the molar ratios were calculated based on the molecular weights of the respective monosaccharides.

The defatted and deproteinized samples of separated layer A and B were accurately weighed in 5 mg and transferred into ampoules containing 2 mL of 3 M TFA. The hydrolysis process was performed and acid hydrolysates were treated in the same method with standards. The 100 µL of hydrolysates mixture was then diluted with 900 µL of deionized water, followed by centrifugation at 12,000 rpm for 5 min. The supernatant was collected for further analysis.

Analysis was performed using a ThermoFisher ICS5000 ion chromatograph. A Dionex CarbopacTM PA10 column (4 × 250 mm) was used, with the mobile phase and elution program detailed in Supplementary Table 4. The flow rate was 1.0 mL·min^−1^, the injection volume was 25 µL, the column temperature was maintained at 30 °C, and an electrochemical detector was employed.

Results of chromatogram were shown as Supplementary Table 5 and Supplementary Table 6 where contents (*C*_x_, μg/mg) of 15 monosaccharides in hydrolyzed layer A and B samples were calculated.

18 $${C}_{x}=\frac{{c}_{s}\times \frac{{A}_{x}}{{A}_{s}}\times D\times {V}_{i}}{{m}_{s}}$$

Here, *c*_s_ represents the concentration of monosaccharide standards (mg/L), *A*_s_ and *A*_x_ represent the peak area of standards and samples, *D* is dilution factor, *V*_i_ is initial volume (mL) of hydrolyzed sample solutions, and *m*_s_ is mass (mg) of samples.

Although monosaccharide composition of hydrolyzed mycelium was measured, it is nonnegligible to convert monosaccharide contents into polysaccharide contents. Due to the high molecular weight of polysaccharide, we simply calculated polysaccharide contents (*C*_y_, μg/mg) according to molar mass (*M*, g/mol) of monosaccharide and H_2_O.

19 $${C}_{{{\rm{y}}}}={C}_{{{\rm{x}}}}\times \left(1-\frac{M\left({{{\rm{H}}}}_{2}{{\rm{O}}}\right)}{M\left({{\rm{monosaccharide}}}\right)}\right)$$

It is worth noting that chitin was hydrolyzed into N-acetylglucosamine trifluoroacetate salt, therefore, the monosaccharide unit of chitin, N-acetylglucosamine was undetectable in ion chromatogram, when N-acetylglucosamine hydrochloride as standard. Chitin contents in layer A and B were measured by enzymatic hydrolysis method.

### Chitin contents measurements

Defatted and deproteinized samples of layer A and B were treated with dextranase (11.0–13.0 μg/mL) to hydrolysis glucan and other polysaccharides except chitin. Enzymatic hydrolysis condition was kept at 50 °C and pH 4.5. After reaction for 48 h, samples were washed with deionized water in centrifuge to remove enzyme and salts. According to weight loss of lyophilized samples before and after hydrolysis, content of glucan could be calculated. Then we could regard residue of deglucaned samples as chitin. Result of enzymatic hydrolysis method offered a lower content than IC testing, we reasoned that the dextranase we adopted could not act on each kind of glucosidic bond in glucan of fungi cell wall. Additionally, much solid residue appeared indicated that other polysaccharide such as mannan and xylan were not hydrolyzed. High similarity in FTIR spectra of layer A and B samples before and after hydrolysis proved that lipid, protein and glucan accounted for low contents in TV. Combined with IC results, we concluded that chitin was majority of TV cell wall.

### Molecular dynamics simulations

To construct models that accord with the true composition of mycelium materials is extremely tough. Limited with our calculation conditions, we simplified the length of polymer chains, including PVA, chitin, glucan, mannan, xylan and galactofucan shown in Supplementary Fig. 45. For force field assigned, parameters were set as: forcefield as COMPASSⅡ, quality as Medium and summation method as Atom based, cutoff distance as 12.5 Å. Geometric optimization was conducted with quality as Fine and algorithm as Smart. Considering that the measured contents of lipid and protein were relatively lower than polysaccharide, and fungi cell wall occupied almost all of mycelium structure, thus we referred hierarchical structure of fungi cell wall, chitin located underneath glucan and xylan, while mannan spreading upon the top layer. Here, we could just make assumptions about that distribution of water, PVA and glycerol in cell wall was homogeneous. Amorphous cells for models before and after moisture adsorption were constructed according to the testing results of molecular composition ratio in Fig.4d, as well as the moisture uptake at 5 min of layer A and B. Force field adopted COMPASSⅡ. And in summation method, electrostatic applied Ewald, while van der Waals applied Atom based, with cutoff distance as 12.5 Å. Quality was selected as medium and initial density was set as 0.5. Temperature at construction was set as 298 K.

We successively carried out geometric optimization, annealing and dynamics processes, with parameters: force field as COMPASSⅡ, algorithm as Smart, quality as Medium, summation method as Ewald for electrostatic and Atom based for van der Waals, cutoff distance as 12.5 Å. Annealing consisted of 5 cycles from 298 to 395 K, with heating ramps per cycle as 6 and dynamics steps per cycle as 10,000. We set parameters in anneal process: force field as COMPASSⅡ, algorithm as Smart, quality as Medium, summation method as Ewald for electrostatic and Atom based for van der Waals, cutoff distance as 12.5 Å. For forcite dynamics in anneal, parameters were set: ensemble as NPT, initial velocities as Random, pressure as 1.0e-4 GPa, time step as 1 fs, thermostat as Andersen, collision ratio as 1.0, barostat as Berendsen, decay constant as 0.1 ps.

Last, dynamics task was carried out with force field as COMPASSⅡ, algorithm as Smart, quality as Medium, summation method as Ewald for electrostatic and Atom based for van der Waals, cutoff distance as 12.5 Å. In dynamics process, we set ensemble as NPT, initial velocities as Random, temperature as 298 K, pressure as 1.0e-4 GPa, time step as 1 fs, total simulation time as 1000 ps, thermostat as Andersen, collision ratio as 1.0, barostat as Berendsen, decay constant as 0.1 ps.

Amorphous cells corresponding to layer A and B in dry and wet state underwent dynamics equilibrium for 1000 ps at 298 K were obtained. Then we started to calculate and visualize the Connolly surface of each system, with grid resolution as Course, grid interval as 0.75 Å, vdW scale factor as 1.00 and Connolly radius as 1.00 Å.

Interaction energy (*E*_int_) in the whole system between biomacromolecules (Bio) and PVA, glycerol (Gly), water respectively was calculated.

20 $${E}_{{{\mathrm{int}}},{{{\rm{A}}}}-{{{\rm{B}}}}}={E}_{{{{\rm{AB}}}}}-({E}_{A}+{E}_{B})$$

Here, *E*_AB_ refers to total energy of A and B complex, *E*_A_ or *E*_B_ refer to individual energy of A or B in system.

Numbers of hydrogen bond (*HB*) in system between biomacromolecules (Bio) and PVA, glycerol (Gly), water respectively was calculated.

21 $${{HB}}_{{{\rm{A}}}-{{\rm{B}}}}={{HB}}_{{{\rm{AB}}}}-({{HB}}_{{{\rm{A}}}}+{{HB}}_{{{\rm{B}}}})$$

Here, *HB*_AB_ refers to total numbers of hydrogen bond in A and B complex, *HB*_A_ or *HB*_B_ refer to numbers of hydrogen bond intra A or B in system.

Radial distribution function (*RDF*), mean square displacement (*MSD*) and scattering were calculated in Forcite analysis. For *R*_g_ calculation, bin width was set as 0.01 Å. In *MSD* analysis, diffusion coefficient (*D*) of water molecules in layer A and B was calculated.

22 $${MSD}=6{Dt}$$

For scattering simulation, cutoff was 50 Å and model size correlation was adopted with radius as 1.7 Å.

### Hygroscopic deformation

Free deformation of untethered TV@P-G was conducted by altering the humidity in a constant temperature and humidity chamber. TV@P-G was cut into strips (2 × 20 mm, 5 × 20 mm and 7 × 20 mm), of which the initial shapes in desiccation (R.T., 30% RH) were photographed. With humidity increasing (R.T., 85% RH) and decreasing, free deformation and recovering of untethered samples was recorded.

Tethered deformation was carried out within a humidity gradient field produced by a humidifier with 30–50 mL·h^−1^ of humidification rate. Strip samples (4 × 16 mm, 4 × 20 mm and 4 × 24 mm) were held vertically by a clamp, with humidification towards the free end. The deformation processes were recorded by video, where the grid paper was adopted as a reference.

### Locomotion

Based on the free deformation amplitude of TV@P-G, we placed the sample (5 × 30 mm) onto a 3D-printed ratchet surface. By humidifying from downstream, TV@P-G sample would deform and motion along the back of the ratchet teeth. Deformation was switched on by humidifying for 5 min in a transparent PMMA box, where humidity was stayed at 85%RH. Due to the structure of the ratchet, the sample could only move forward along the ratchet. When ambient humidity reduced, the sample took 1 h to recover its shape, and left its initial position with the restriction of the ratchet. The pitch of ratchet was designed into 7 mm and 4 mm, whereas the 7-mm pitch was much wider than deformation amplitude of the sample. Locomotion on ratchet surface with 4-mm pitch was then realized.

### Myce strain gage and data collection

Strain gage modules (BF350-20AA) with CMOS dual operational amplifier (TP09-SR 3PEAK) were purchased from Shenzhen RunesKee Co., Ltd. We used instant glue (ergo5400, Switzerland) to bond a strain gage onto TV@P-G, obtaining Myce strain gage. Output voltage of blank and Myce strain gage could be tested by a voltmeter. Furthermore, in order to collect real-time voltage signals, we adopted an Arduino microcontroller (UNO-R3) and a program we wrote. Meanwhile, a humidity probe (SNT957W-DE, Shenzhen SOAN Co., Ltd) was fixed nearby the Myce strain gage upon exposure to moisture, which visualized the real-time humidity around the humidifier. We plotted output voltage of blank and Myce strain gage, along with real-time humidity values to showcase the response process and synchronization between humidity and electrical signal of Myce strain gage.

### Humidity sensor for circuit control

The output of Myce strain gage modules was connected with a relay to control electrical appliances. Here, we selected a high-level triggered relay (DC 5 V) considering the rising trend of input signal from Myce strain gage when humidity increasing, with the circuit shown as Supplementary Fig. 56. The reason why a fan was adopted as the electrical appliance was that the triggered fan would promote moisture diffusion and evaporation of adsorbed water in Myce strain gage, resulting in shape recovery and voltage signal decreasing, realizing a feedback regulation.

### Resistance measurement

A Keithley 2400 source meter unit (SMU) was employed to collect real-time resistance values of mycelium materials. In open environment (20 °C, 15 – 20% RH), one-sided humidification was applied to surface A or B of TV@P-G that was hold by electrode clamps. And homogeneous humidity was realized by fixing the TV@P-G specimen in a constant humidity chamber, with resistance measurement synchronous.

### Determination of cell viability

Dormant TV@P-G was cut into 5 mg of pieces, washing twice with PBS. FDA (10 mg·mL^−1^ in acetone), PI (1 mg·mL^−1^ in water), MTT (5 mg·mL^−1^ in PBS) and Mito-Tracker Red CMXRos (200 μM in DMSO) stock solutions were respectively prepared. For FDA-PI staining, 500 μL of working solution with 50 μg·mL^−1^ of FDA and 5 μg·mL^−1^ of PI was adopted, incubated at 25 °C for 30 min and then washing twice with DI water before sectioning.

For MTT treatment, MTT working solution (25 μL of stock solution in 500 μL of DI water) was filtered with a 0.22-μm filter membrane for sterilization and then reacted with dormant mycelium at 28 °C in the dark for 4 h. After removing the supernatant, MTT formazan produced in dormant TV@P-G was extracted with 500 μL of DMSO oscillated in 70 rpm at 25 °C until the purple of samples faded. Ultraviolet-visible spectra from 300 nm to 600 nm of MTT formazan extracted from samples in different weights were collected with an ultraviolet-visible spectrophotometer (Eppendorf BioSpectrometer basic). Absorbance at 490 nm was plotted and analyzed with linear fitting. Fluorescence excitation and emission spectra of MTT formazan in DMSO were collected with a fluorescence spectrometer (FL 8500 PerkinElmer) at 25 °C.

Mito-Tracker Red CMXRos stock solution was diluted 1000 times into 1 mL of working solution. Native mycelium membrane and TV@P-G sample was incubated in working solution at 37 °C for 30 min.

Confocal images were captured with the following excitation (*λ*_ex_) and emission (*λ*_em_) wavelength detection: FDA, *λ*_ex_ = 488 nm, detection 500–530 nm; PI, *λ*_ex_ = 552 nm, detection 610–640 nm; MTT formazan, *λ*_ex_ = 405 nm, detection 430–480 nm; Mito-Tracker Red CMXRos, *λ*_ex_ = 552 nm, detection 580–610 nm.

### Total antioxidant capacity

DPPH Free Radical Scavenging Capacity Assay Kit (BIOESN) was employed to quantify the antioxidant capacity of antioxidant substances with Trolox equivalent. Native mycelium membrane and dormant TV@P-G were weighed in *m*_Tot_, grinded in liquid nitrogen and then mixed with 10 times of extracting solution. Trolox standard solution was control group. After homogenization in ice bath and centrifugation at 4 °C for 10 min, the supernatant was mixed with DPPH agent by a volume ratio of 2:3 and incubated at 25 °C in dark for 20 min. Absorbance at 515 nm (*A*_515_) of DPPH radical was measured using an ultraviolet-visible spectrophotometer (Eppendorf BioSpectrometer basic). DPPH free radical scavenging rate (*FRSR*) and total antioxidant capacity (*TAOC*) of native mycelium membrane and dormant TV@P-G were deduced as:

23 $${FRSR}=\left(1-\frac{{A}_{515}\left({{\rm{sample}}}\right)}{{A}_{515}\left({{\rm{Trolox}}}\right)}\right)\times 100\%$$

24 $${TAOC}=\left(\frac{{FRSR}}{100}+0.003\right)-\frac{0.0287\times V}{{m}_{{{{\rm{Tot}}}}}\times D}$$

In particular, *D* refers to proportion of dry mycelium in fresh mycelium membrane and dried TV@P-G, obtained from above experiments, respectively, in 5.805% for fresh mycelium membrane and 20.33% for dried TV@P-G.

### Regeneration

Dormant TV@P-G samples kept in a sterile environment for 3 months was transferred onto PDA plates. Mycelium regeneration obviously appeared at 28 °C, 85% RH from the 5th day, and then regrew and completely spread on PDA surface in 10 days.

### Self-healing

Dormant TV@P-G samples were cut and separated. Placed onto PDA plates at 28 °C, 85% RH, with the cross sections contacting closely, both parts of separated samples started regeneration and extended into each other until the cross sections were repaired. We added mixed solution of 10% PVA and 10% glycerol around the healed crack and then dried the samples. The healed crack was imaged with SEM and the healed mechanical properties were characterized by uniaxial tensile tests.

### Recycled cultivation

Fragments of dormant TV@P-G were transferred into MEB medium with antibiotics (ampicillin and kanamycin sulfate) and cultured at 28 °C. After 20-day regeneration, the newborn mycelium membrane was treated with the mixed solution of 10% PVA and 10% glycerol.

The old TV@P-G fragments were also useful to be recycled into culture medium. Grind samples into powder and extract nutrients with 20 times weight of water, finally sterilize to obtain the culture medium named as TVEB. It took 30 days for *Trametes versicolor* to grow and formation a newborn mycelium membrane. Mechanical properties of the finally dried newborn TV@P-G were tested.

### Life cycle assessment

This study adheres to the framework of ISO 14040/14044 standards to perform a cradle-to-gate life cycle assessment (LCA) of the material production process. The assessment was conducted using SimaPro software, which is linked to background databases including Ecoinvent. The IPCC 2021 GWP100 methodology was employed to specifically evaluate the global warming potential (GWP) of the product over a 100-year time horizon, with results reported in units of “kilograms of carbon dioxide equivalent (kg CO₂-eq)”, (Supplementary Table 7). The functional unit was defined as “the production of 1 kg of the sample”. The assessment scope encompassed all processes from raw material acquisition and energy production to the point of product dispatch (the “gate”). This included the manufacturing of key raw materials (e.g., water, malt extract, glycerol, PVA), electricity consumption, and direct CO₂ emissions, but excluded post-dispatch stages such as transportation, usage, and end-of-life disposal, (Supplementary Tables 8–10). The model did not explicitly incorporate the construction and decommissioning of infrastructure (e.g., factory buildings and equipment) as capital goods, nor did it account for associated long-term emissions. A systematic analysis of the inventory analysis and impact assessment results was conducted to identify carbon emission hotspots within the process, thereby providing a scientific basis for subsequent process optimization and environmental decision-making.

### Degradation

Soil was collected from Harbin Institute of Technology. TV@P-G pieces (~30 mg) were buried in soil at 28 °C, 30% RH. Remove samples from soil every three days, wipe off the surface soil, dry, and weigh. The mass reduction of samples was plotted and degraded morphology was observed with SEM.

### Plasticizer migration measurements

Plasticizer diffusion was measured following ISO 177:2016 with modifications. Samples were prepared with diameter of 10 mm. For each cyclic humidity condition (85%RH for 12 h to 35%RH for 12 h and 35% RH for 24 h) at room temperature, three parallel experiments were conducted. Each sample was placed between two layers of 2‑mm‑thick contact materials and clamped firmly: filter paper, PVA sheets, or polytetrafluoroethylene (PTFE) sheets. The PVA sheets were prepared by pouring and drying with 10%PVA solution. Mass loss was measured each 24 h by weighing the sample. Three independent replicates were performed for each combination of humidity and contact material.

### Reporting summary

Further information on research design is available in the Nature Portfolio Reporting Summary linked to this article.

## Supplementary information

Supplementary Information

Description of Additional Supplementary Files

Supplementary Movie 1

Supplementary Movie 2

Supplementary Movie 3

Supplementary Data 1

Reporting Summary

Peer Review File

## Source data

Source data

## Data Availability

All experimental data supporting the findings of the study are available within the article and its Supplementary Information. Source data are provided with this paper. All data are available from the corresponding author upon request. Optimized atomic coordinates and molecular dynamics trajectories are provided as a supplementary data file. Source data are provided with this paper.
