# Supplementary material for: Tailored living mycelium macerate for reproducible flexible actuators
Source: Nat Commun. 2026 Aug 12;17:9682. doi: 10.1038/s41467-026-76623-z (PMC13558639; doi:10.1038/s41467-026-76623-z)
Supplement: Supplementary file 1 [file 41467_2026_76623_MOESM1_ESM.pdf]

Supplementary Information

Tailored Living Mycelium Macerate for Reproducible Flexible Actuators

Rui Nie[1], Baoyuan Li[1], Luxuan Li[1], Guangyao Hou[2], Yikai Gao[1], Xiaonan Wang[3], Yan Huang[1], Mei Zhu[1], Xiaoman Liu[1]* and Xin Huang[1]*

[1]State Key Laboratory of Advanced Inorganic Fibers and Composites, School of Chemistry and Chemical Engineering, Harbin Institute of Technology, Harbin 150001, P. R. China.
[2]School of Materials Science and Engineering, Harbin Institute of Technology, Harbin, Heilongjiang 150001, P. R. China.
[3]State Key Laboratory of Urban Water Resource & Environment, School of Chemistry and Chemical Engineering, Harbin Institute of Technology, Harbin 150001, P. R. China.

*Corresponding author. E-mails: liuxiaoman@hit.edu.cn; xinhuang@hit.edu.cn

Supplementary Tables and Figures

Supplementary Table 1 Precision limitations for each measured parameter

Parameter	Accuracy	Standard
Width, length	± 0.01 mm	Accuracy of vernier caliper
Tensile strength	± 0.1 MPa	Significant figure rules
Young's modulus	± 0.1 MPa	Significant figure rules
Elongation rate	± 1%	ASTM D882-18
Toughness	± 0.1 MJ/m^3	Significant figure rules
Bending angle	± 0.2°	Image pixel resolution

Supplementary Table 2 Mechanical properties of TV@P-G macerates

PVA content (%)	Glycerol content (%)	*E* (MPa)	*TS* (MPa)	*EG* (%)	*T* ($MJ\ m^{-3}$)
5	5	25.8 ± 3.4	2.3 ± 0.2	14 ± 2	0.2 ± 0.1
5	10	18.5 ± 1.1	3.3 ± 0.1	25 ± 1	0.5 ± 0.1
5	15	5.9 ± 0.4	1.4 ± 0.2	24 ± 3	0.2 ± 0.1
5	25	2.8 ± 0.2	0.7 ± 0.0	24 ± 1	< 0.1
10	5	348.7 ± 78.6	13.7 ± 3.0	11 ± 4	1.1 ± 0.4
10	10	23.7 ± 6.0	4.1 ± 0.4	36 ± 8	1.0 ± 0.2
10	15	12.5 ± 1.7	3.3 ± 0.3	81 ± 35	2.0 ± 0.9
10	25	5.0 ± 0.3	1.8 ± 0.3	158 ± 58	2.3 ± 1.2
15	5	180.2 ± 26.4	8.3 ± 0.8	26 ± 16	1.5 ± 0.8
15	10	31.8 ± 2.6	3.7 ± 0.4	27 ± 8	0.7 ± 0.3
15	15	17.1 ± 1.8	3.7 ± 0.3	47 ± 8	1.2 ± 0.3
15	25	9.9 ± 0.2	2.4 ± 0.4	124 ± 61	2.3 ± 1.1

Supplementary Table 3 Comparison of tensile properties of flexible mycelial materials

Viability	Fungi	Treatment	State	*E* (MPa)	*TS* (MPa)	*EG* (%)	*T* (MJ m^{-3})	Research
Dead	*G. lucidum*	Native	Fresh	101.28	3.88	5.92	N.A.	1
Dead	*G. lucidum*	Native	Dried	142.58	3.98	8.10	N.A.	1
Dead	*F. fraxinea*	Hotpress	Dried	3.94	3.99	3.62	N.A.	2
Dead	*F. fraxinea*	Hotpress +15%Gly	Dried	4.08	4.54	20.32	N.A.	2
Dead	*G. lucidum*	Hotpress +2%Gly	Dried	9.90	3.44	35.08	0.72	3
Dead	*R. delemar*	Press+TGB	Dried	180	6.92	17	0.76	4
Dead	*A. koreana*	Deacetylation +tannic acid+30%Gly	Dried	159.65	3.18	3.53	0.08	5
Dead	*Pleurotus ostreatus*	Deacetylation +25%Poly(glycerol-3)	Dried	416.8	10.8	13.11	N.A.	6
N.A.	*S. commune*	8%Gly	Dried	124	6.4	5.67	0.15	7
N.A.	*S. commune*	32%Gly	Dried	3	1.8	29.6	0.22	7
N.A.	*S. commune*	16%PG-3	Dried	80	5.8	24.9	N.A.	8
Living	*G. lucidum*	Native	Dried	11.85~22.65	0.92~1.11	8.00~13.88	N.A.	9
Living	*T. versicolor*	Native	Dried	456.7	10.07	4.87	0.31	10
Living	*A. niger*	Filtered	Dried	1.9	4.97	34.33	0.94	11
Living	*S. commune*	5%CNT+2.5%PVA	Dried	29.8	270	7.36	0.12	12
Living	*S. commune*	Fiber dispersion	Dried	200~4500	27.1~118.9	6.2~45.9	10.5	13
Dead	*P. eryngii*	PVA+4% dialdehyde cellulose	Dried	N.A.	13.8	33.9	N.A.	14
Living	*T. versicolor*	10%PVA+10%Gly	Dried	13.4	4.1	36	1.0	This Work
Living	*T. versicolor*	10%PVA+5%Gly	Dried	348.7	23.7	11	1.1	This Work
Living	*P. chrysosporium*	10%PVA+10%Gly	Dried	17.8	4.1	87	2.8	This Work
Living	*T. versicolor*	10%PVA+10%Gly, R2G1	Dried	8.7	2.4	204	2.9	This Work
Living	*Cladophialophora* sp.	10%PVA+10%Gly	Dried	9.7	6.9	349	18.0	This Work

Continued Supplementary Table 3

Viability	Fungi	Treatment	State	*E* (MPa)	*TS* (MPa)	*EG* (%)	*T* (MJ m^{-3})	Research
Living	*T. versicolor*	2%CMC+10%Gly	Dried	1.0	0.4	38	< 0.1	This Work
Living	*T. versicolor*	10%TVE+10%Gly	Dried	1.4	0.4	35	< 0.1	This Work
Living	*T. versicolor*	10%Pul+10%Gly	Dried	2.4	0.4	23	< 0.1	This Work
Living	*T. versicolor*	10%Pec+10%Gly	Dried	1.1	0.4	42	< 0.1	This Work
Living	*T. versicolor*	10%Pul+10%DG	Dried	116.3	1.6	3	< 0.1	This Work

Supplementary Table 4 Mobile phase and elution process of IC testing

Time (min)	A (H_2O) %	B (500 mM NaOH 50mM NaOAc) %	C (20mM NaOH) %
0.000	0.0	0.0	100.0
30.000	50.0	0.0	50.0
30.100	70.0	30.0	0.0
46.000	70.0	30.0	0.0

Supplementary Table 5 Results of IC testing and calculation for layer A

Monosaccharide	Peak area	Retention time	Molar ratio	C_x (μg/mg)	C_y (μg/mg)
Fucose	3.43	4.967	0.0278	15.973	14.21987
Rhamnose	0	8.217	0	0	0
Galactosamine hydrochloride	0	8.383	0	0	0
Arabinose	0	9.6	0	0	0
Glucosamine hydrochloride	0.296	9.9	0.0007	0.491	0.545649
Galactose	0.662	12.175	0.0041	2.596	2.336631
Glucose	149.076	13.142	0.5675	357.634	321.9024
Mannose	20.954	14.342	0.1908	120.218	108.2069
Xylose	44.095	14.6	0.2092	109.853	96.67064
Fructose	0	16.408	0	0	0
Ribose	0	18.275	0	0	0
Galacturonic acid	0	36.433	0	0	0
Gururonic acid	0	36.833	0	0	0
Glucuronic acid	0	38.025	0	0	0
Mannuronic acid	0	39.367	0	0	0

Supplementary Table 6 Results of IC testing and calculation for layer B

Monosaccharide	Peak area	Retention time	Molar ratio	C_x (μg/mg)	C_y (μg/mg)
Fucose	1.166	4.975	0.0185	5.43	4.834024
Rhamnose	0	8.217	0	0	0
Galactosamine hydrochloride	0.356	8.4	0.0015	0.566	0.42305
Arabinose	0	9.6	0	0	0
Glucosamine hydrochloride	0.805	9.867	0.0035	1.335	1.483588
Galactose	0.879	12.183	0.0107	3.447	3.102606
Glucose	80.871	13.15	0.6032	194.01	174.6262
Mannose	9.481	14.35	0.1691	54.395	48.96033
Xylose	20.823	14.617	0.1935	51.876	45.65088
Fructose	0	16.408	0	0	0
Ribose	0	18.275	0	0	0
Galacturonic acid	0	36.433	0	0	0
Gururonic acid	0	36.833	0	0	0
Glucuronic acid	0	38.025	0	0	0
Mannuronic acid	0	39.367	0	0	0

Supplementary Table 7 LCA inventory for 100 g mycelium macerate (10%PVA+10%Gly).

Items	Corresponding LCI	Amount	Unit
	Mycelium macerate (10%PVA+10%Gly)		
Sample	Products	100	g
	Materials/fuels		
Water	Water, decarbonised {GLO}| diethyl ether production | Cut-off, U	1333.3	g
Malt extract	Wheat flour {RoW}| market for wheat flour | Cut-off, U	96.7	g
Glycerine	Glycerine {RoW}| market for glycerine | Cut-off, U	44.9	g
PVA	Self-mode	34.9	g
	Electricity/heat		
Electricity	Electricity, low voltage {GLO}| market group for electricity, low voltage | Cut-off, U	0.35	kWh
	Emissions to air		
CO_2	Carbon dioxide	78.4	g

Supplementary Table 8 LCA inventory for 1.0 kg PVA production

Items	Corresponding LCI	Amount	Unit
	PVA		
PVA	Products	1	kg
	Materials/fuels		
VAc	Vinyl acetate {GLO}| market for vinyl acetate | Cut-off, U	1.65	kg
Methanol	Methanol {RoW}| market for methanol | Cut-off, U	0.45	kg
NaOH	Sodium hydroxide, without water, in 50% solution state {RoW}| market for sodium hydroxide, without water, in 50% solution state | Cut-off, U	0.08	kg
Water	Tap water {GLO}| market group for tap water | Cut-off, U	10	kg
	Electricity/heat		
Electricity	Electricity, low voltage {GLO}| market group for electricity, low voltage | Cut-off, U	1.07	kWh
Steam	Steam, in chemical industry {GLO}| smelting and refining of nickel concentrate, 16% Ni | Cut-off, U	5	kg
	Emissions to air		
CO_2	Carbon dioxide	2.5	kg
	Emissions to water		
COD	COD (Chemical Oxygen Demand), CN	120	g
	Waste to treatment		
Wastewater	Wastewater, average {RoW}| clinker production | Cut-off, U	5.1	m^3
MSW	Municipal solid waste {GLO}| treatment of municipal solid waste, unsanitary landfill, wet infiltration class (500mm) | Cut-off, U	0.15	kg

Supplementary Table 9 LCA inventory for 1.0 kg PVAc production

Items	Corresponding LCI	Amount	Unit
	PVAc		
PVAc	Products	1	kg
	Materials/fuels		
VAc	Vinyl acetate {GLO}| market for vinyl acetate | Cut-off, U	1.03	kg
Nitrogen	Nitrogen, liquid {RER}| Cut-off, U	0.00125	kg
Compressed air	compressed air, average installation, >30kW, 8 bar gauge, at supplynetwork {RER}| | Cut-off, U	1.73	m^3
Water	Tap water {GLO}| market group for tap water | Cut-off, U	2.48	kg
	Electricity/heat		
Electricity	Electricity, low voltage {GLO}| market group for electricity, low voltage | Cut-off, U	0.376	kWh
Steam	Steam, in chemical industry {GLO}| smelting and refining of nickel concentrate, 16% Ni | Cut-off, U	1.40	kg
Natural gas		0.885	MJ
	Emissions to air		
Heat	Heat waste | Cut-off, U	1.36	MJ

Supplementary Table 10 LCA inventory for 1.0 kg VAc production

Items	Corresponding LCI	Amount	Unit
	VAc		
VAc	Products	1	kg
	Materials/fuels		
Ethylene	Ethylene {RoW}| market for ethylene | Cut-off, U	0.334	kg
Acetic acid	Acetic acid, without water, in 98% solution state {GLO}| market for acetic acid, without water, in 98% solution state | Cut-off, U	0.705	kg
Oxygen	Oxygen, liquid {RoW}I market for oxygen, liquid | Cut-off, U	0.196	kg
Water	Tap water {GLO}| market group for tap water | Cut-off, U	2.48	kg
	Electricity/heat		
Electricity	Electricity, medium voltage {AU}| market for electricity, medium voltage I Cut-off, U	1.65	kWh
Natural gas	Heat, district or industrial, natural gas {RoW}I market for heat, district orindustrial, natural gas |Cut-off, U	1.856	MJ
	Emissions to air		
Acetic acid		0.00141	kg
Carbon dioxide		0.0623	kg
Ethylene		0.00067	kg
	Emissions to water		
BOD5	BOD5 (Bioloaical Oxygen Demand)	0.0696	kg
Acetic acid		0.000564	kg

Supplementary Table 11 The calculation results of GWP for 1.0 kg mycelium macerate based on IPCC 2021

Num	Damage category	GWP100
Unit		kg CO_2-eq
Total		7.63
CO_2	CO_2	0.784
Water	Water, decarbonised {GLO}| diethyl ether production | Cut-off, U	0.046
Malt extract	Wheat flour {RoW}| market for wheat flour | Cut-off, U	0.899
Glycerine	Glycerine {RoW}| market for glycerine | Cut-off, U	1.44
PVA	PVA	1.88
Electricity	Electricity, low voltage {GLO}| market group for electricity, low voltage | Cut-off, U	2.59

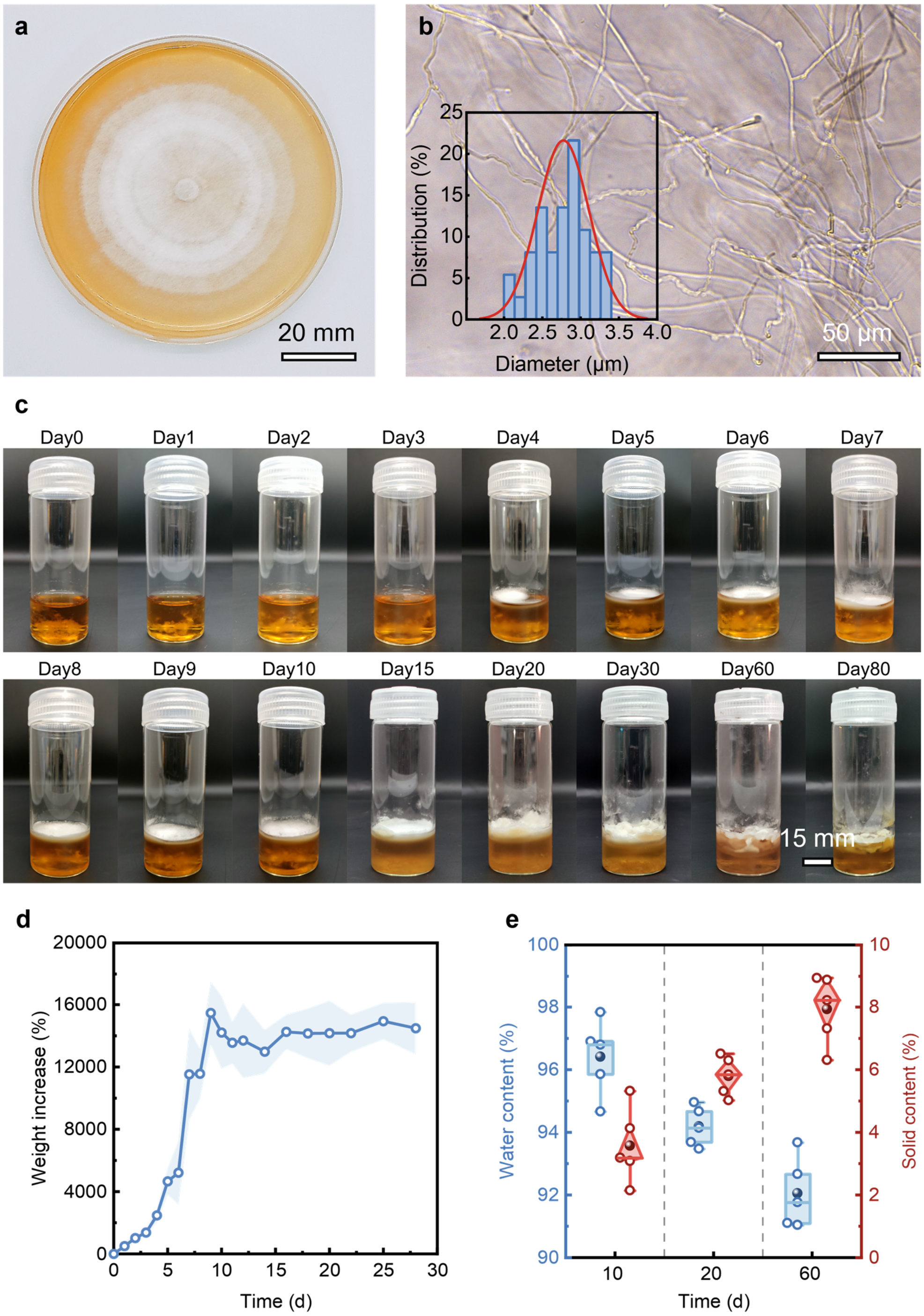

Supplementary Fig. 1 Growth features of *Trametes versicolor*. **a**, *Trametes versicolor* growing on MEB solid medium. **b**, Optical image of Trametes versicolor hypha cell. The inset is diameter distribution of Trametes versicolor hypha cell. **c**, Optical images recording the formation to aging process in 80 days of native *Trametes versicolor* mycelium membrane on the gas-liquid interface of MEB medium. **d**, Fungal biomass dry weight and growth curve of Trametes versicolor cultured in MEB liquid medium. Data are presented as mean values ± standard deviation (SD). **e**, Distribution of water content and solid content of native mycelium membrane cultured for 10, 20 and 60 days, n = 5. Box plot with the minima, maxima, centre, bounds of box and whiskers, and percentiles (25th, 75th) defined in the legend. The whiskers extend to the most extreme data points within 1.5×IQR, with values beyond this range plotted as outliers.

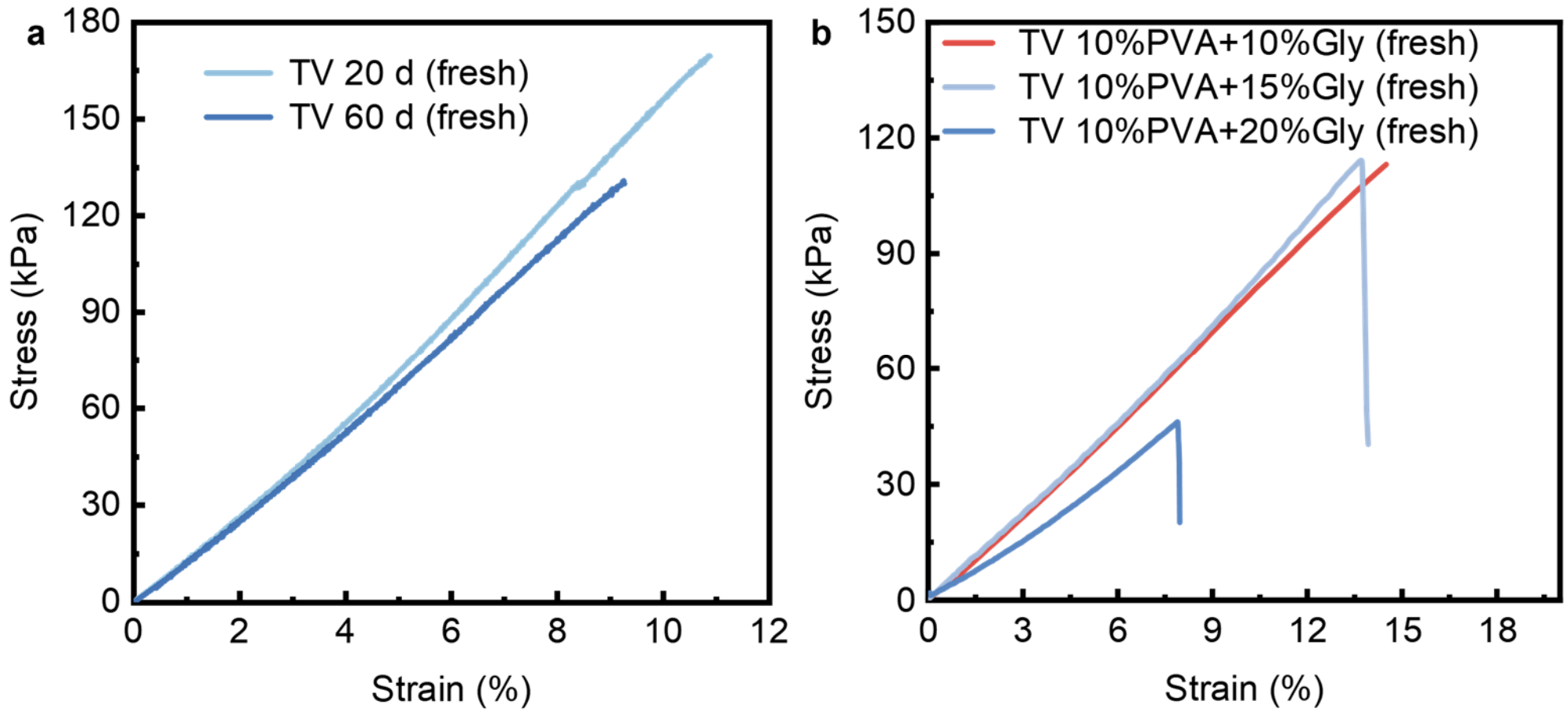

Supplementary Fig. 2 Mechanical properties of fresh mycelium materials. Tensile stress-strain curves of fresh native mycelium membrane (**a**) and TV@P-G cultured for 20 d (**b**).

A Trade-off for optimizing cultivation time

Native mycelium membrane samples were harvested at day 10, 20 and 60 for solid content measurement (Supplementary Fig. 1d) and tensile test (Supplementary Fig. 2a). Extending the cultivation time from 10 to 20 days significantly increased solid content (5.8%) and enhanced mechanical properties, as the 10-day samples were too fragile for proper clamping. While 60-day cultures achieved higher solid content (7.9%), they exhibited reduced tensile strength and elongation at break due to mycelium senescence.

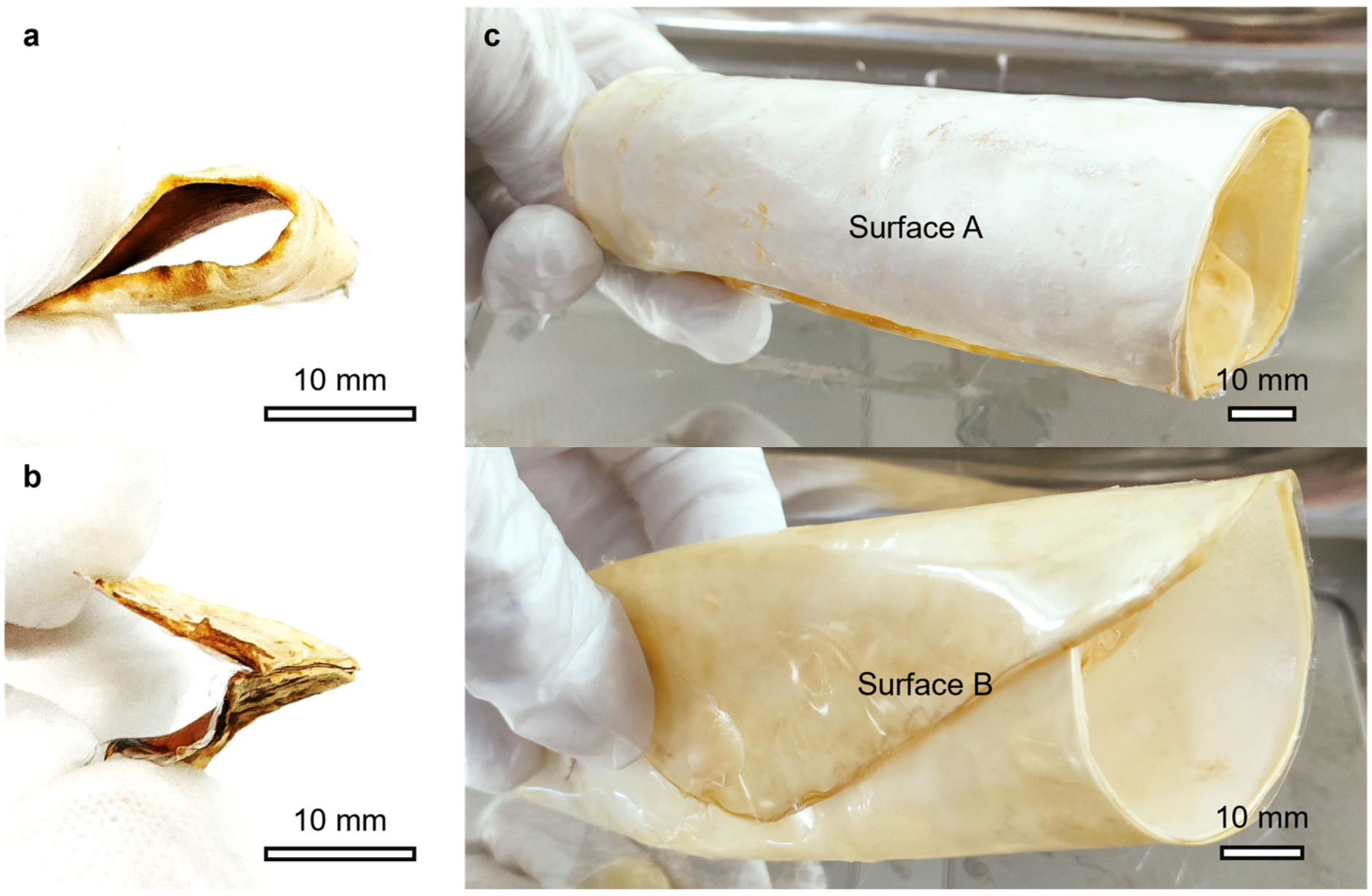

Supplementary Fig. 3 Optical images displaying flexibility of mycelium macerates. **a**, **b**, Bending TV 10%PVA+10%Gly (**a**) and TV 10%PVA (**b**) samples. **c**, Janus TV@P-G (10%PVA+10%Gly, 185×235 mm).

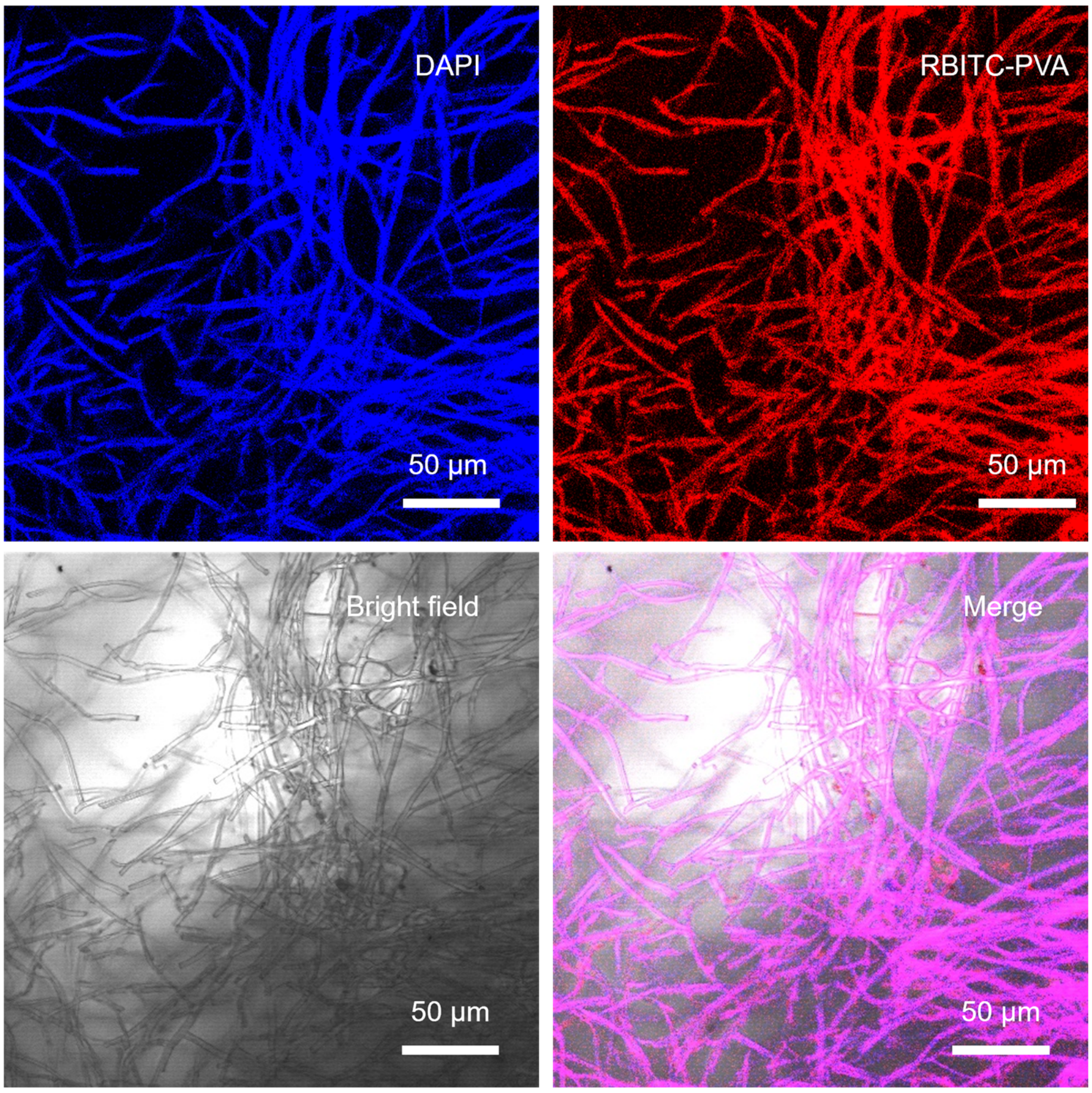

Supplementary Fig. 4 Confocal images of DAPI dyed TV@(RBITC-PVA)-Gly section. Shown is a representative histogram from three independent experiments (n = 3) with similar outcomes.

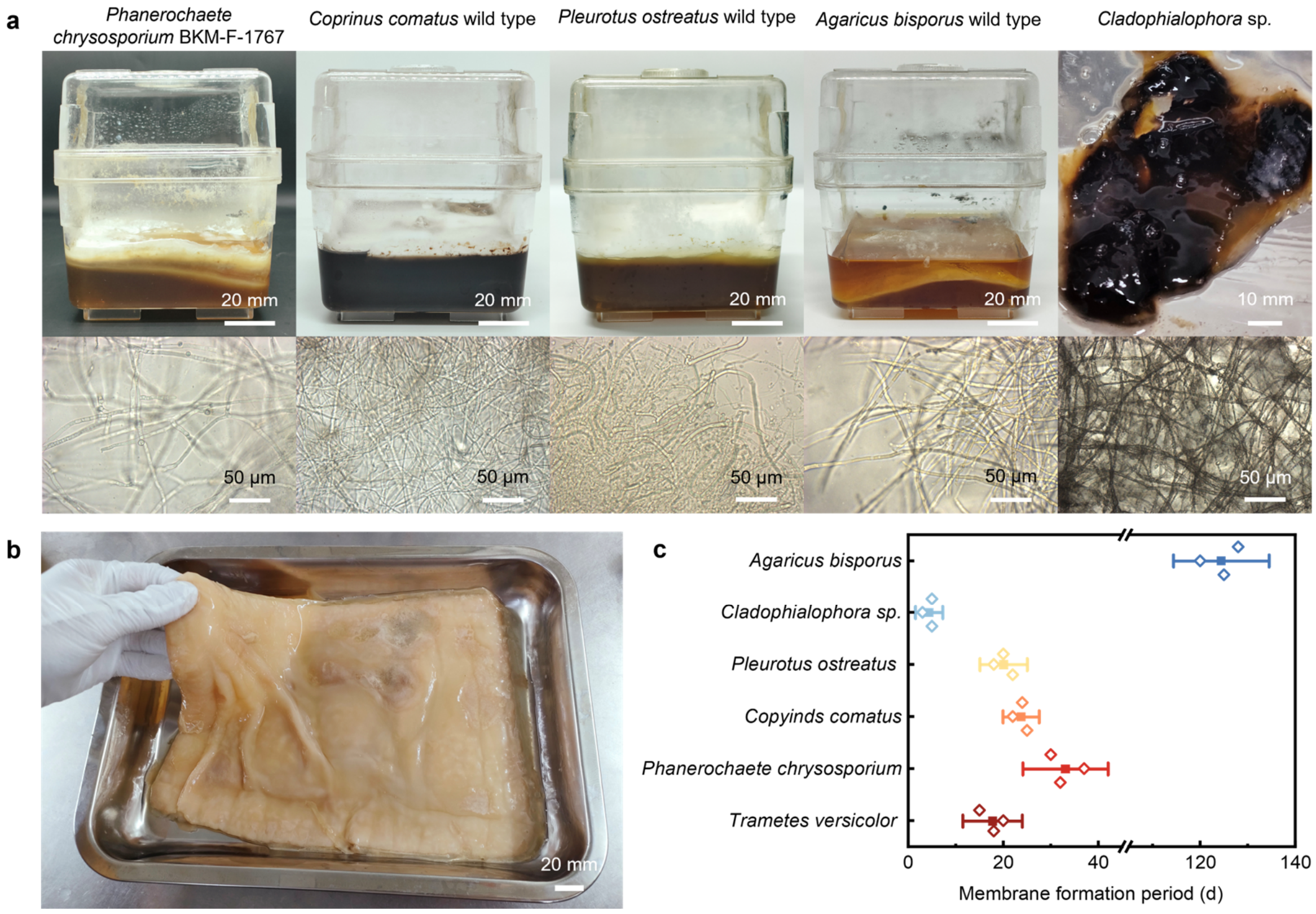

Supplementary Fig. 5 Mycelium membrane formation of various fungal species. **a**, Optical images of *Phanerochaete chrysosporium* BKM-F-1767, *Coprinus comatus* wild type, *Pleurotus ostreatus* wild type, *Agaricus bisporus* wild type and black yeast-like fungi（*Cladophialophora* sp.) membranes. **b**, Native mycelium membrane of *Phanerochaete chrysosporium* via liquid state fermentation in scale of 255×310 mm. **c**, Comparison of mycelium membrane formation period of different fungi species, n = 3. Data are expressed as mean ± standard deviation (SD).

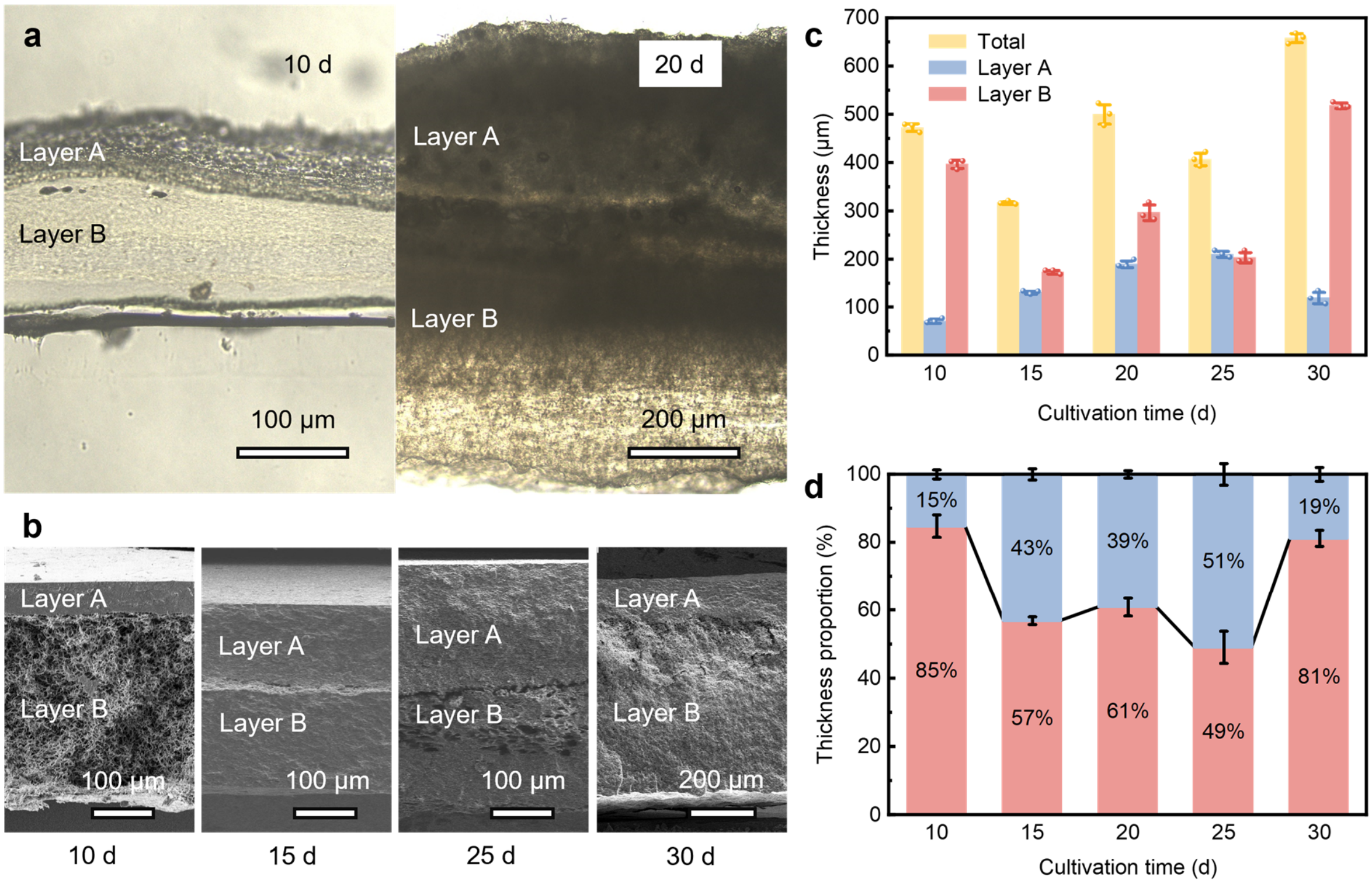

Supplementary Fig. 6 Correlation between Janus structure and cultivation time. **a**, Optical images of macerates sections cutured for 10 and 20 d. **b**, SEM images comparing cross sections of Janus TV@P-G cultured for 10-30 d. Shown is a representative histogram from three independent experiments (n = 3) with similar outcomes The sample cultured for 20 d refers to Fig.2c. **c**, **d**, Thickness changes (**c**) and proportion changes of layer A/B in total thickness (**d**), n = 10. Data are expressed as mean ± standard deviation (SD).

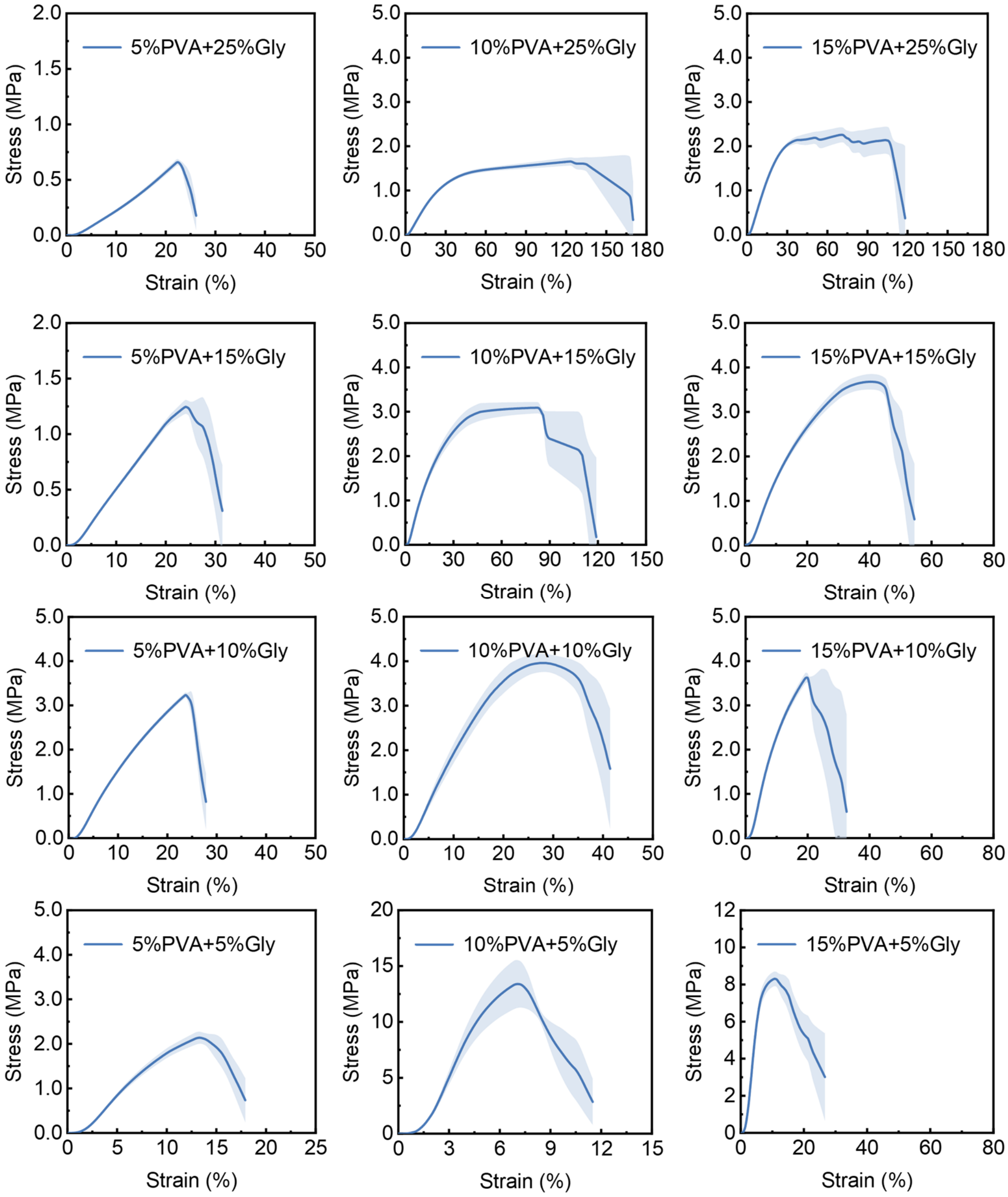

Supplementary Fig. 7 Tensile stress-strain curves of TV@P-G treated with 5-15% PVA and 5-25% glycerol, n = 5. Error bars (mean values ± SEM) were connected using B-spline basis function and visualized as a shadow effect around the data points.

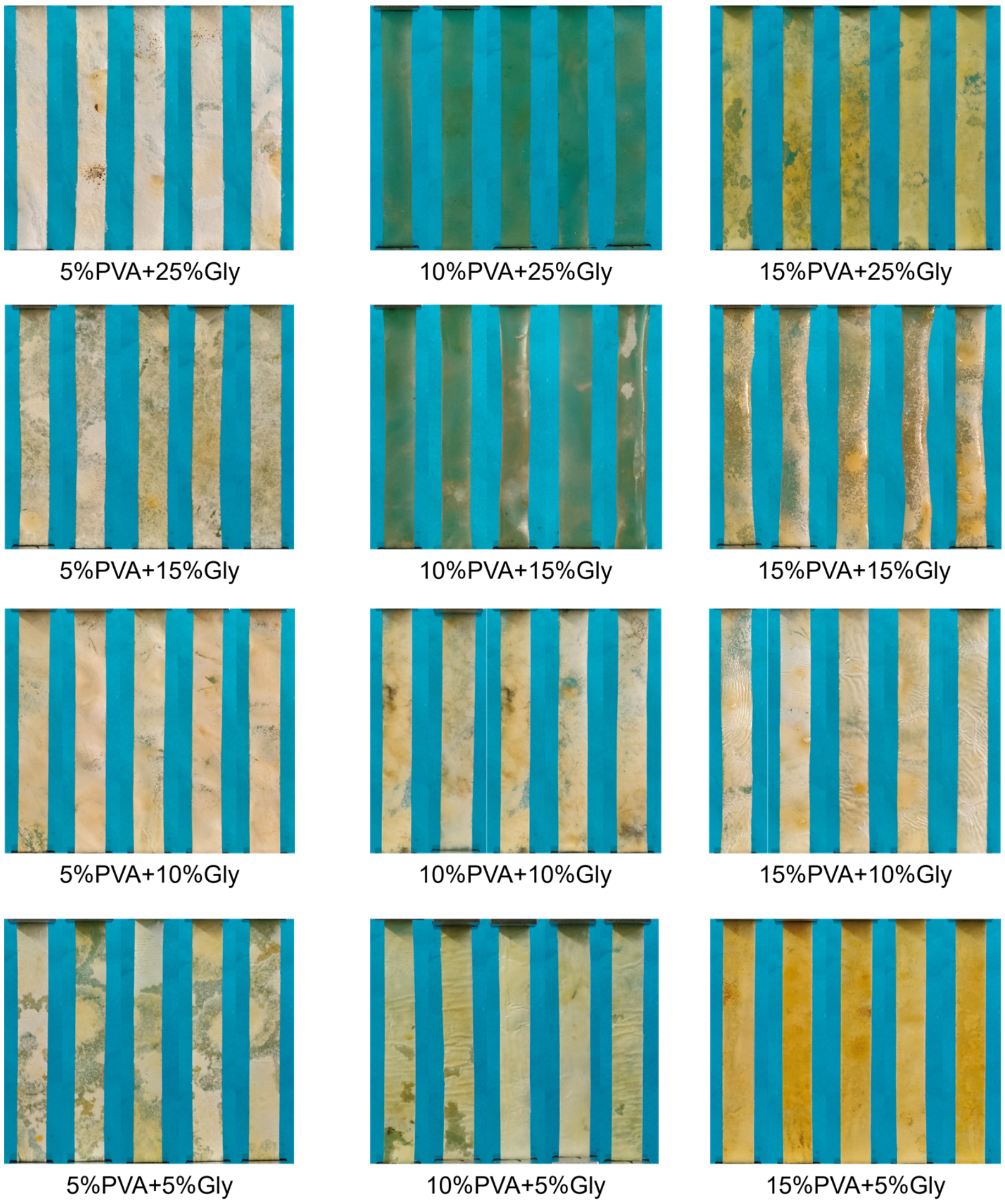

Supplementary Fig. 8 Optical images of mycelium macerate TV@P-G samples for tensile testing, n = 5. Sample dimensions (width 10 mm, initial grip separation 80 mm, total length 100 mm).

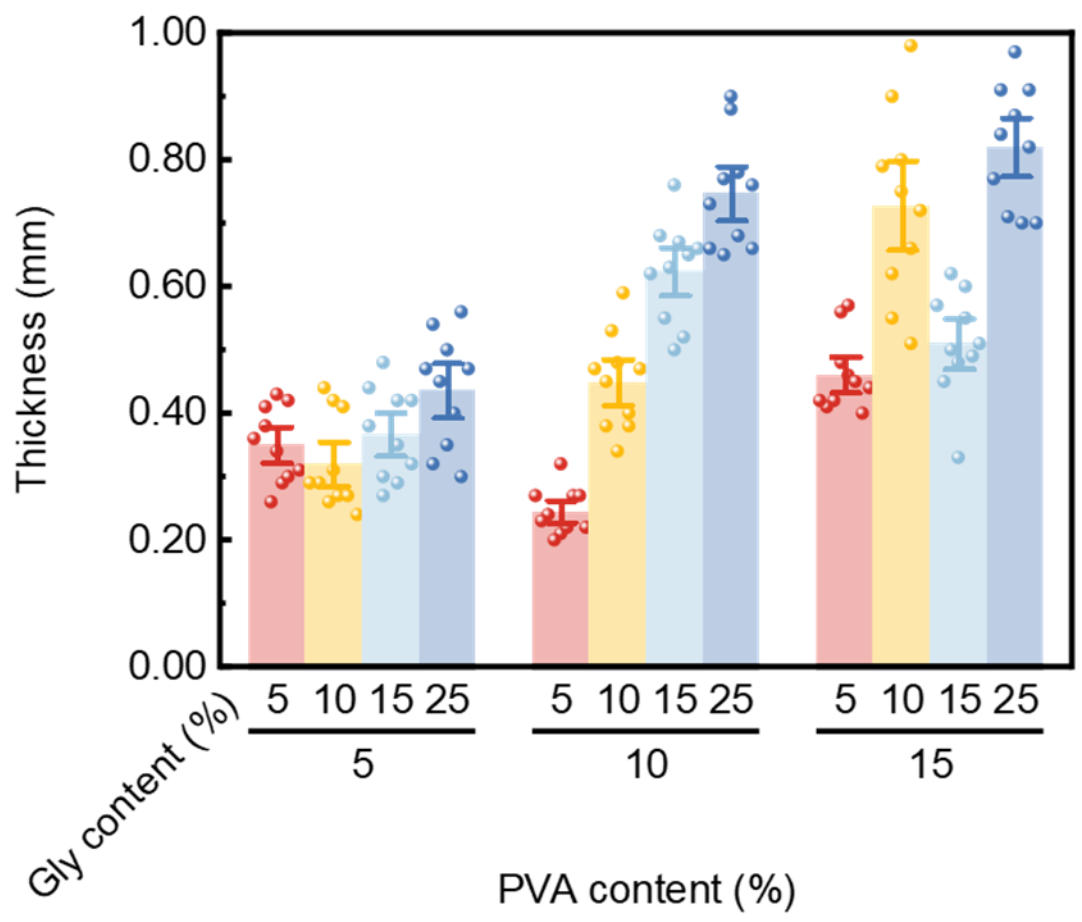

Supplementary Fig. 9 Thickness of TV@P-G macerates treated with different PVA and glycerol contents. Each group of experiments was repeated 10 times (n = 10). Data are expressed as mean ± standard deviation (SD).

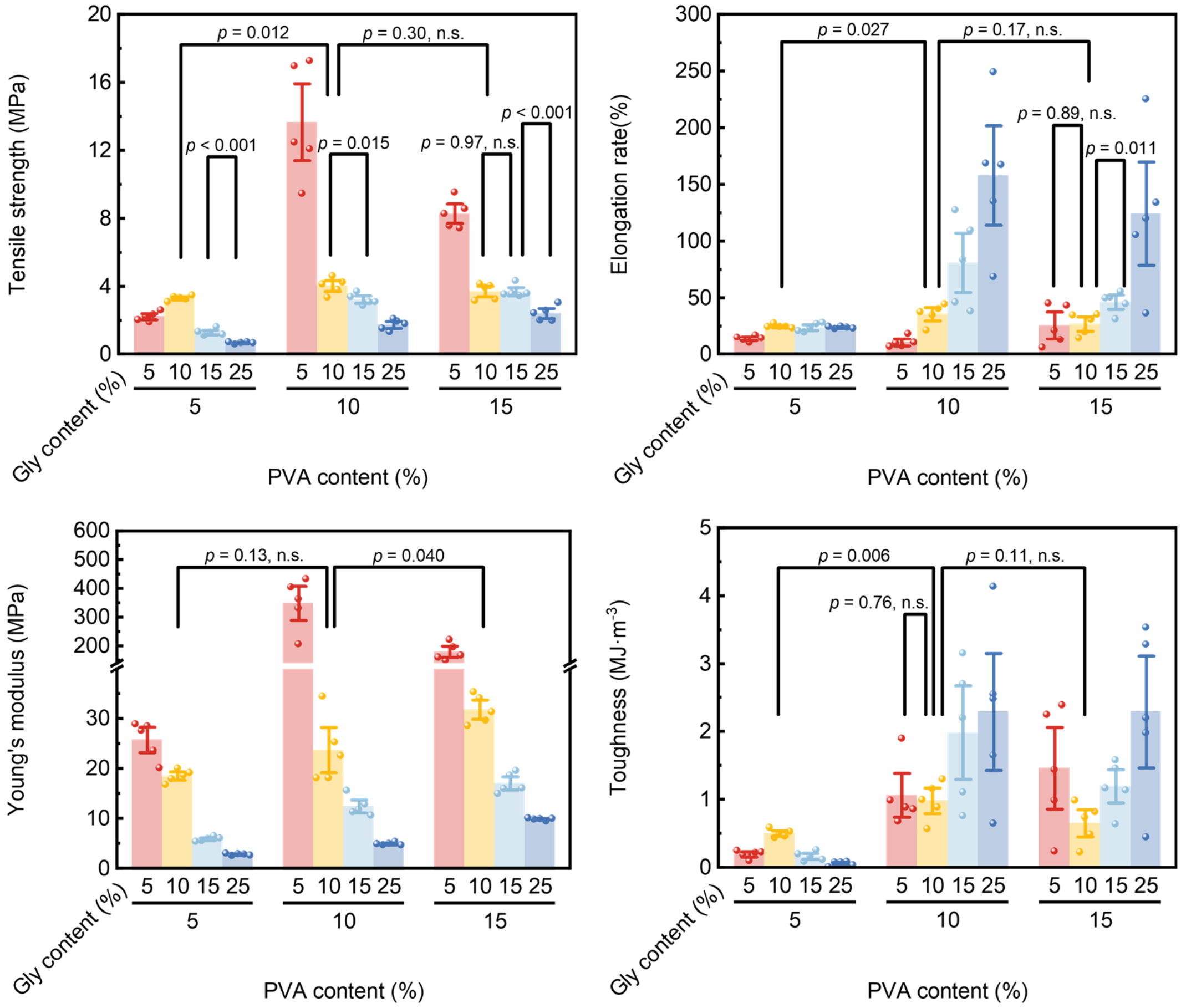

Supplementary Fig. 10 Statistically analyze the influence of different PVA and glycerol contents on the mechanical performance parameters (*TS*, *EG*, *E* and *T*) of TV@P-G macerate, and conduct screening by comprehensively considering the mechanical performance and material input. Each group of experiments was repeated 5 times (n = 5). Data are expressed as mean ± standard deviation (SD). A two-tailed t-test was used to compare similar data.

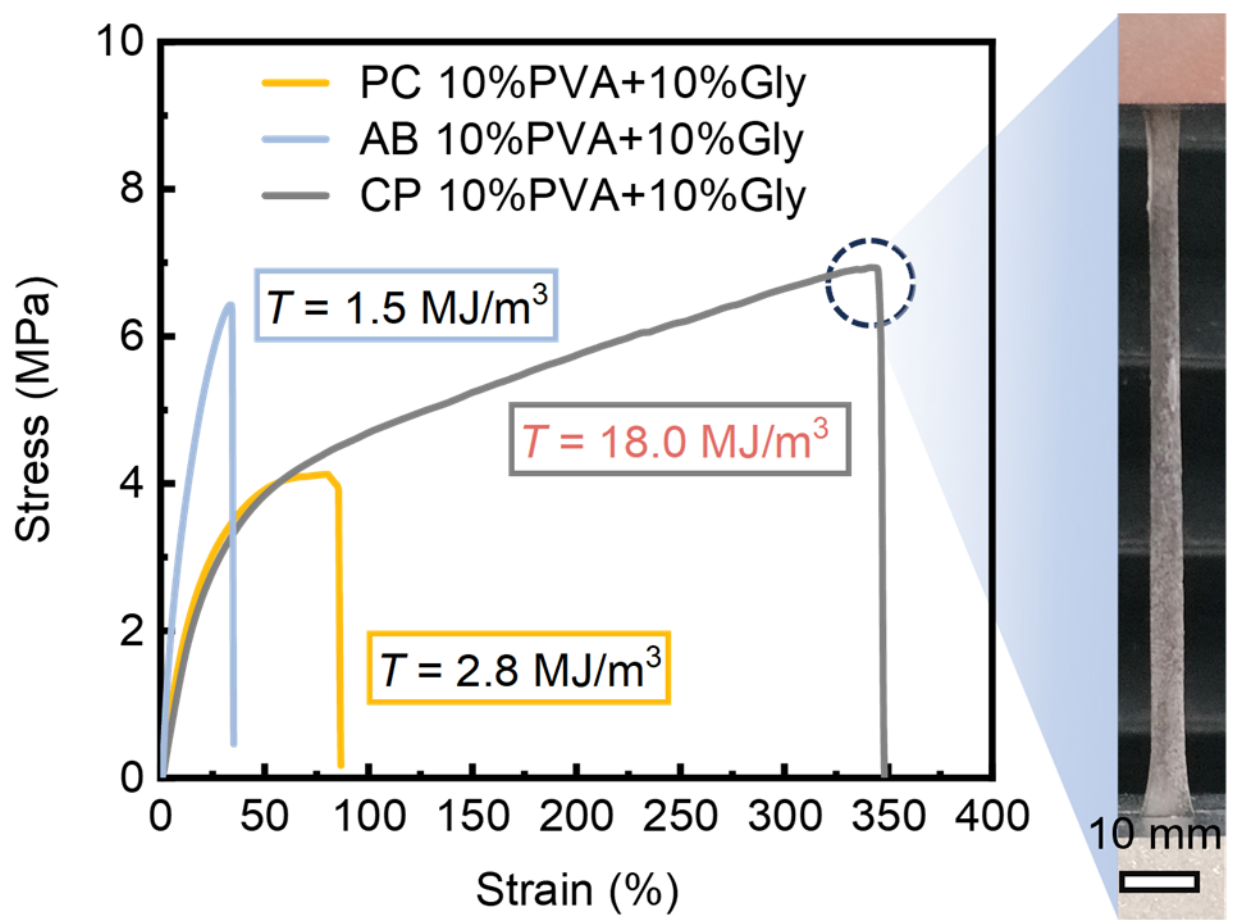

Supplementary Fig. 11 Tensile stress-strain curves of *Phanerochaete chrysosporium*, *Agaricus bisporus* and *Cladophialophora* sp. membrane treated with 10% PVA and 10% glycerol. The experiment was repeated independently three times (n = 3) with similar trends, and a representative experiment is shown.

Strengthening mechanism of PVA and plasticizing mechanism of glycerol

To elucidate the strengthening mechanism of PVA, the investigation on intermolecular interaction was carried out. In thermogravimetry (TG) tests (Supplementary Fig. 15a), weight reduction and thermal decomposition of mycelium biomass were delayed by PVA, owing to their intermolecular interaction. Comparing Fourier Transform infrared (FTIR) spectra of TV@PVA (Supplementary Fig. 15b), the signal at 1540 cm^{-1} reduced with PVA content increasing, corresponding to C-N stretching vibration, ν(C-N) and N-H bending vibration, δ(N-H) of chitin in hyphae cell wall, confirming the hydrogen bond formation between chitin and PVA. These observations suggest that PVA treatment induces hyphal densification, potentially through molecular interactions between PVA and hyphal biomacromolecules upon desiccation. Furthermore, 2D correlation spectra of TV@PVA (Supplementary Fig. 16) proved that PVA interacts with C=O of biomacromolecule (chitin) preferentially, recorded as 1653 cm^{-1} ν(C=O) → 3327 cm^{-1} ν(O-H) → 1540 cm^{-1} δ(N-H), ν(C-N) → 1434 cm^{-1} δ(O-H) → 1040 cm^{-1} ν(C-O-C).

Thermogravimetric (TG) analysis revealed that glycerol elevates the thermal decomposition temperature of the biomass (Supplementary Fig. 17). Fourier-transform infrared (FTIR) spectra of TV@Gly further showed a shift in the O–H stretching vibration (ν(O–H)) from 3360 cm^{-1} (polysaccharide-associated) to lower wavenumbers (Supplementary Fig. 18a), indicative of hydrogen-bond disruption between biomass macromolecules and glycerol interaction. Furthermore, 2D correlation spectra (Supplementary Fig. 18b, c) revealed that glycerol preferentially affects ν(O-H), and 3360 cm^{-1} ν(O-H, polysaccharide) → 3474 cm^{-1} ν(O-H, glycerol) → 1040 cm^{-1} ν(C-O-C) → 1653 cm-1 ν(C=O) → 1540 cm-1 δ(N-H), ν(C-N). Collectively, these results demonstrate glycerol's penetration into the mycelial matrix and its competitive hydrogen-bonding with polysaccharides, underpinning its plasticizing effect.

To further prove the mechanism of PVA and glycerol manipulation on mycelium, density functional theory (DFT) calculation was carried out. Interaction energies, E_{int} between PVA, glycerol and biomacromolecules (chitin and glucan) reflected that stronger interaction between glycerol and biomacromolecules ($|E_{int,\ glycerol\text{-}chitin} = -72.34\ kJ\cdot mol^{-1}|$, $|E_{int,\ glycerol\text{-}glucan} = -93.49\ kJ\cdot mol^{-1}| > |E_{int,\ chitin\text{-}chitin} = -37.79\ kJ\cdot mol^{-1}|$, $|E_{int,\ glucan\text{-}glucan} = -52.96\ kJ\cdot mol^{-1}|$) break the weak intramolecular force of chitin and glucan, respectively (Fig. 2e, f), while intramolecular force of glycerol, $E_{int,\ glycerol\text{-}glycerol}$ ($-39.19\ kJ\cdot mol^{-1}$) is weak. Therefore, as small molecule, glycerol promotes chains slide in mycelium elongation remarkably. Similarly, PVA forms close interaction with biomacromolecules ($E_{int,\ PVA\text{-}chitin} = -84.99\ kJ\cdot mol^{-1}$, $E_{int,\ PVA\text{-}glucan} = -59.85\ kJ\cdot mol^{-1}$) but strong intramolecular force ($E_{int,\ PVA\text{-}PVA} = -92.09\ kJ\cdot mol^{-1}$), thus PVA chains enhances the weak interaction between biomacromolecules, as non-covalent bonding agent (Supplementary Fig. 19).

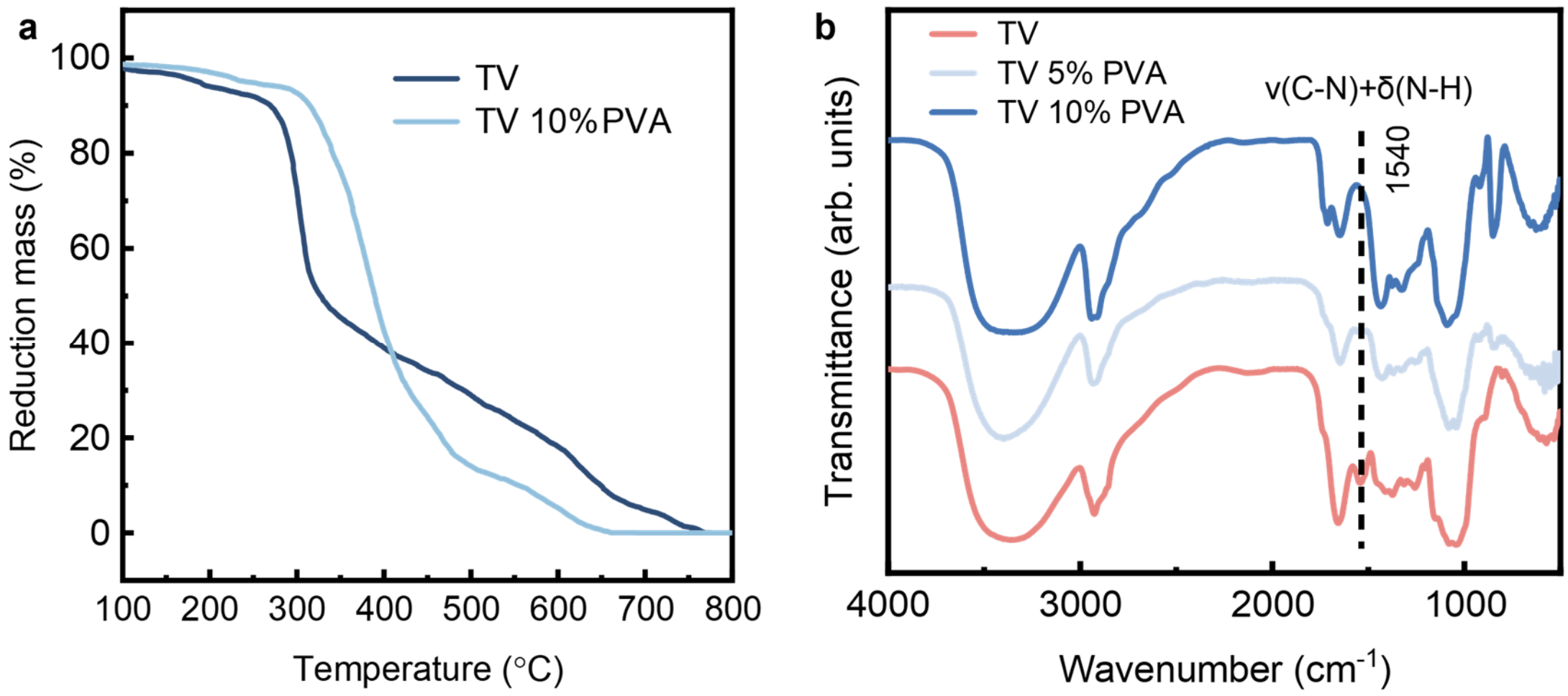

Supplementary Fig. 12 Characterization of interaction between fungal biomacromolecules and PVA by TGA and FTIR. **a**, TG curves TV@PVA. **b**, FTIR spectra of TV@PVA.

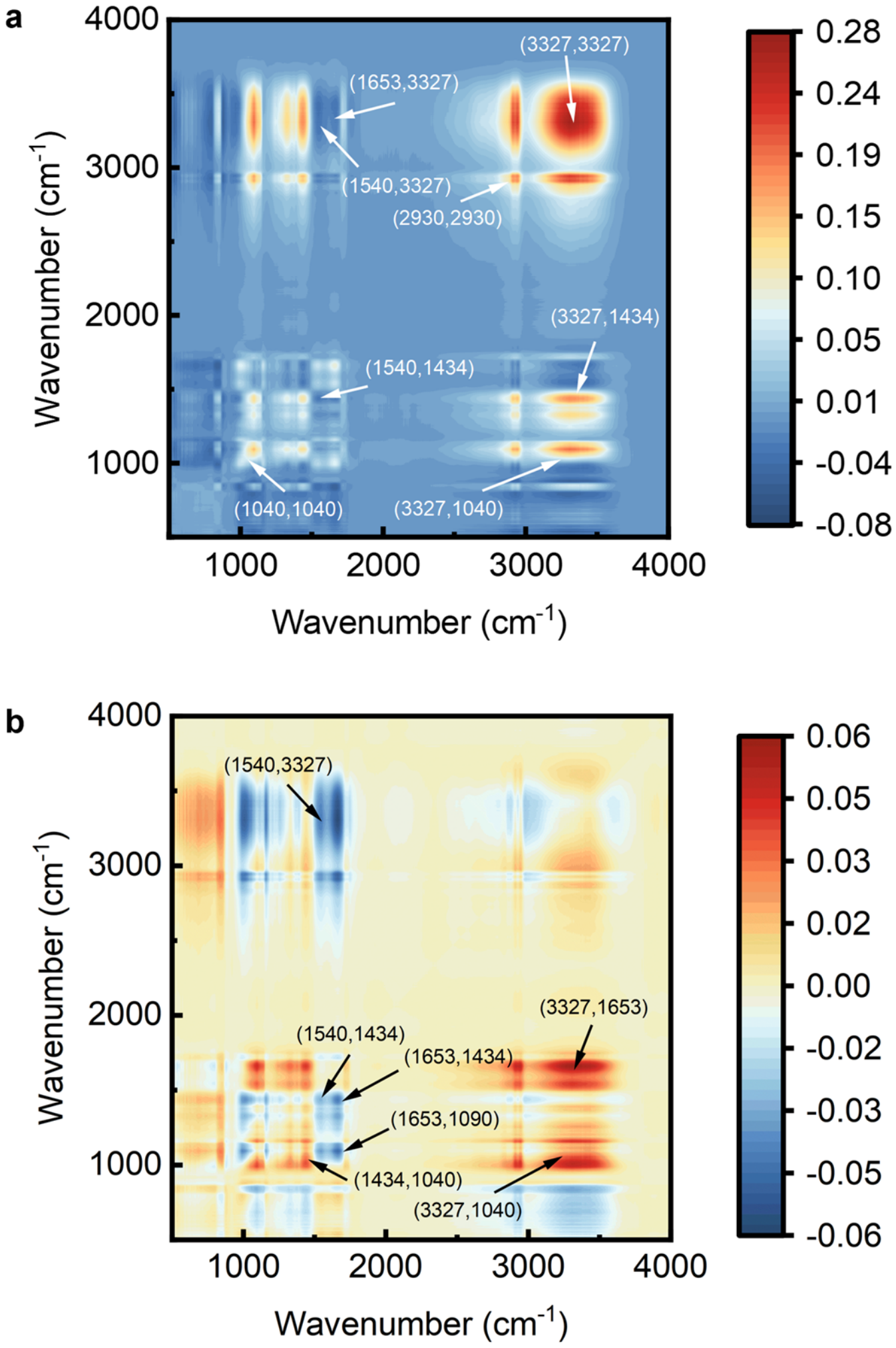

Supplementary Fig. 13 2D correlation infrared spectra of TV@PVA. **a**, 2D correlation synchronous spectra of TV@PVA. **b**, 2D asynchronous spectra of TV@PVA. PVA interacts with C=O of biomacromolecule (chitin) preferentially, recorded as 1653 cm-1 ν(C=O) → 3327 cm-1 ν(O-H) → 1540 cm-1 δ(N-H), ν(C-N) → 1434 cm-1 δ(O-H) → 1040 cm-1 ν(C-O-C).

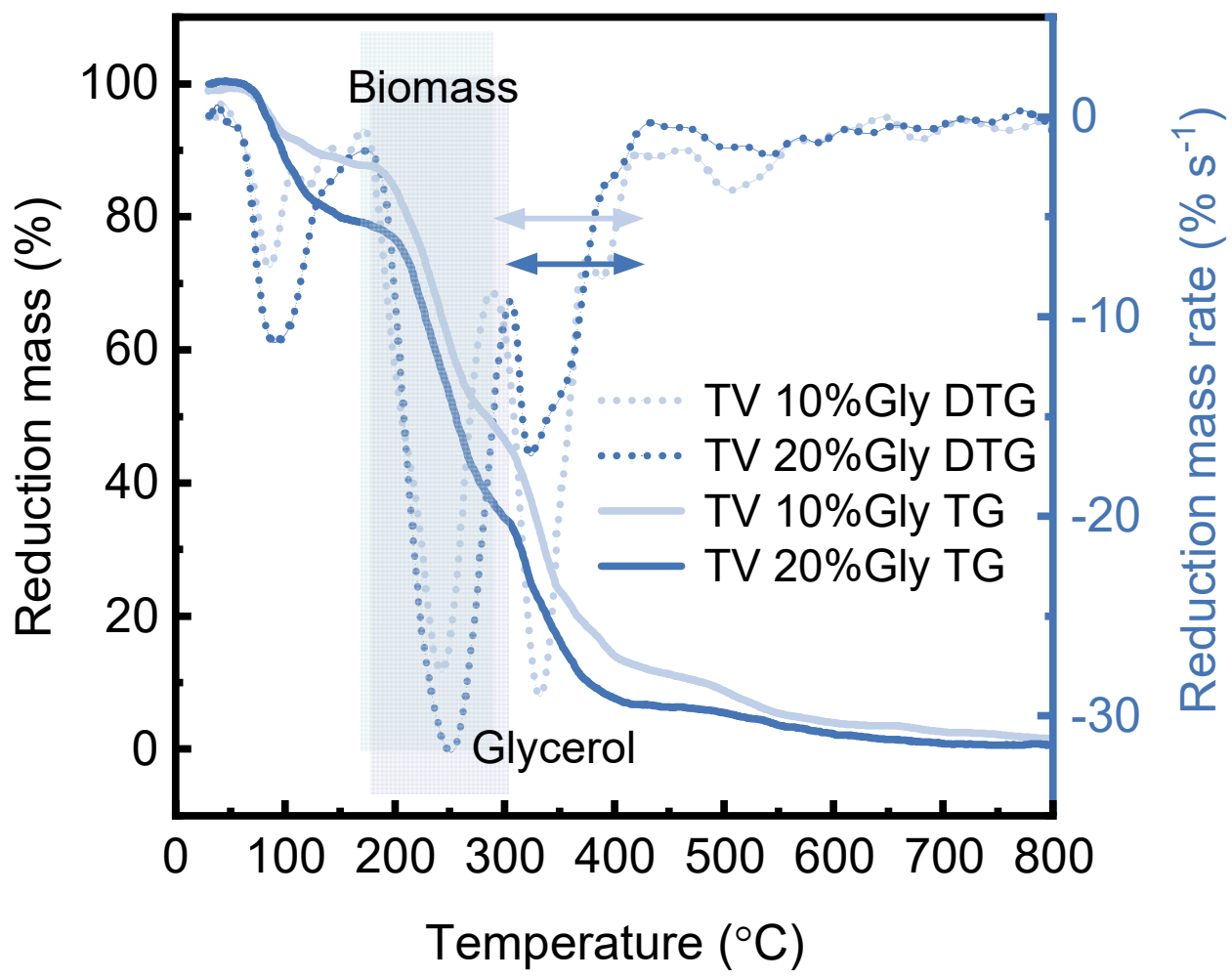

Supplementary Fig. 14 TG and DTG curves of TV@Gly. Decomposition temperature of TV@Gly increased 8.4℃ with glycerol treatment from 10% to 20%. Reduction mass rate equals to TG curves derivative multiplying heating rate 40 ℃/min.

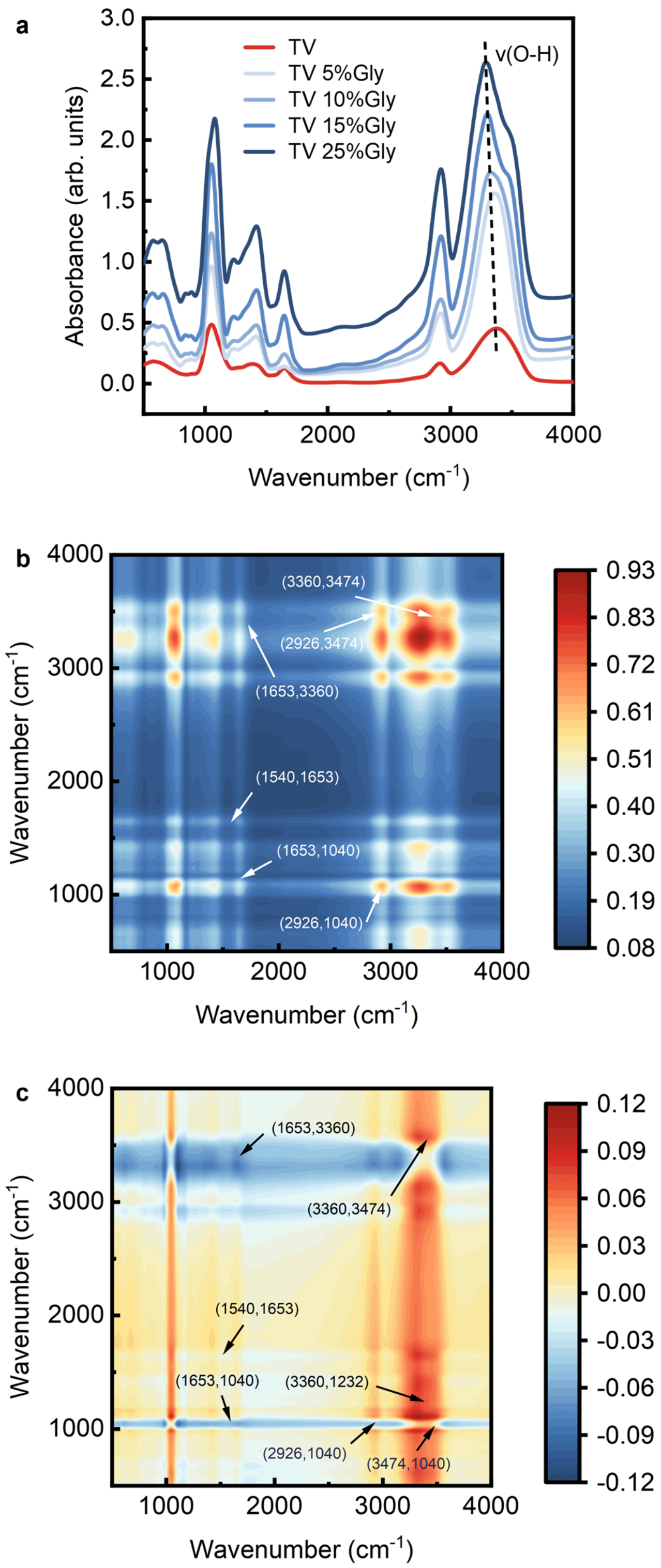

Supplementary Fig. 15 Infrared absorption spectra of TV@Gly. **a**, FTIR spectra of TV@Gly. **b**, **c** 2D correlation synchronous (**b**) and asynchronous (**c**) spectra of TV@Gly. Glycerol preferentially affects ν(O-H), and 3360 cm-1 ν(O-H, polysaccharide) → 3474 cm-1 ν(O-H, glycerol) → 1040 cm-1 ν(C-O-C) → 1653 cm-1 ν(C=O) → 1540 cm-1 δ(N-H), ν(C-N).

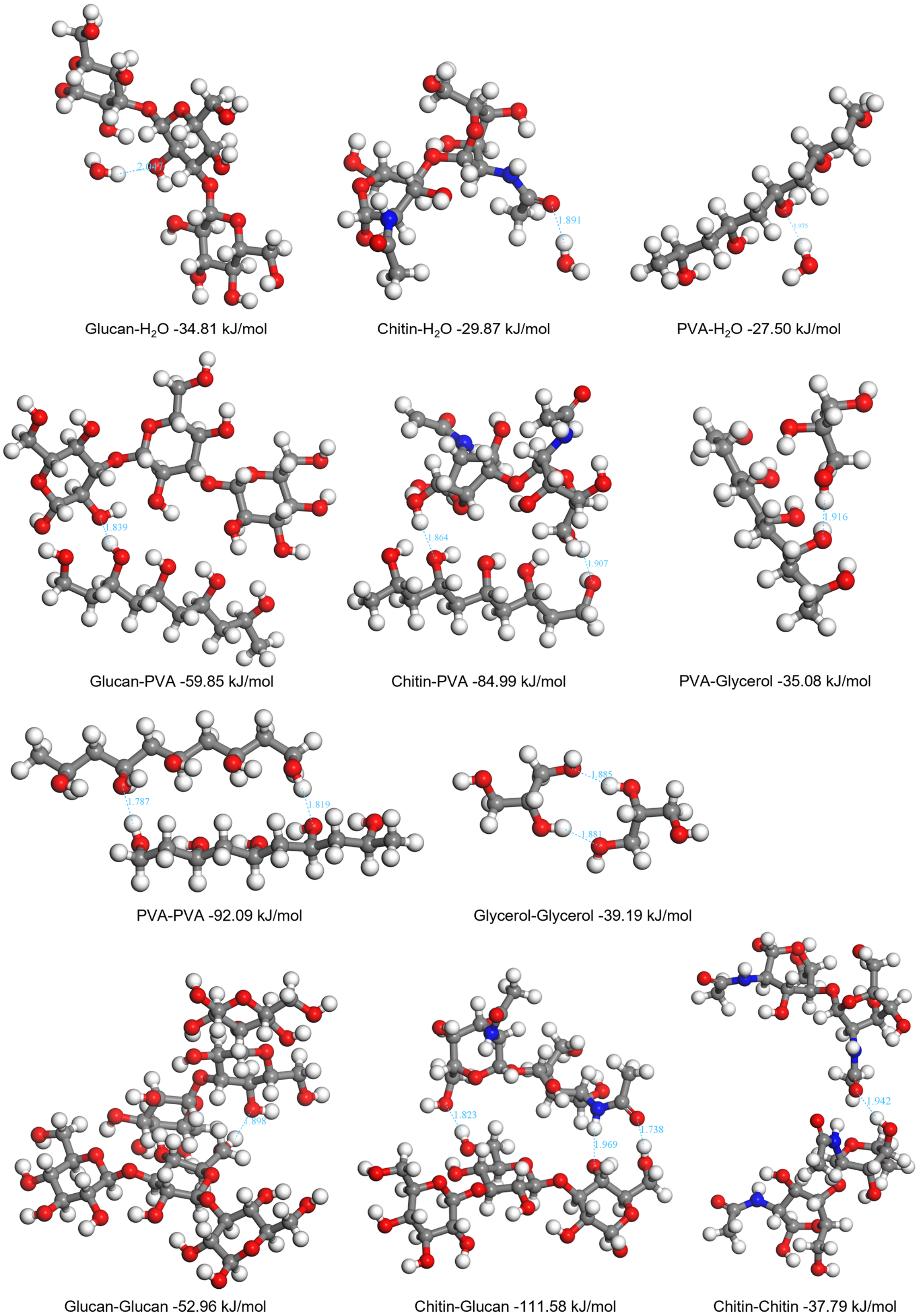

Supplementary Fig. 16 Intermolecular interactions and hydrogen bond between molecules in mycelium macerates from DFT calculation.

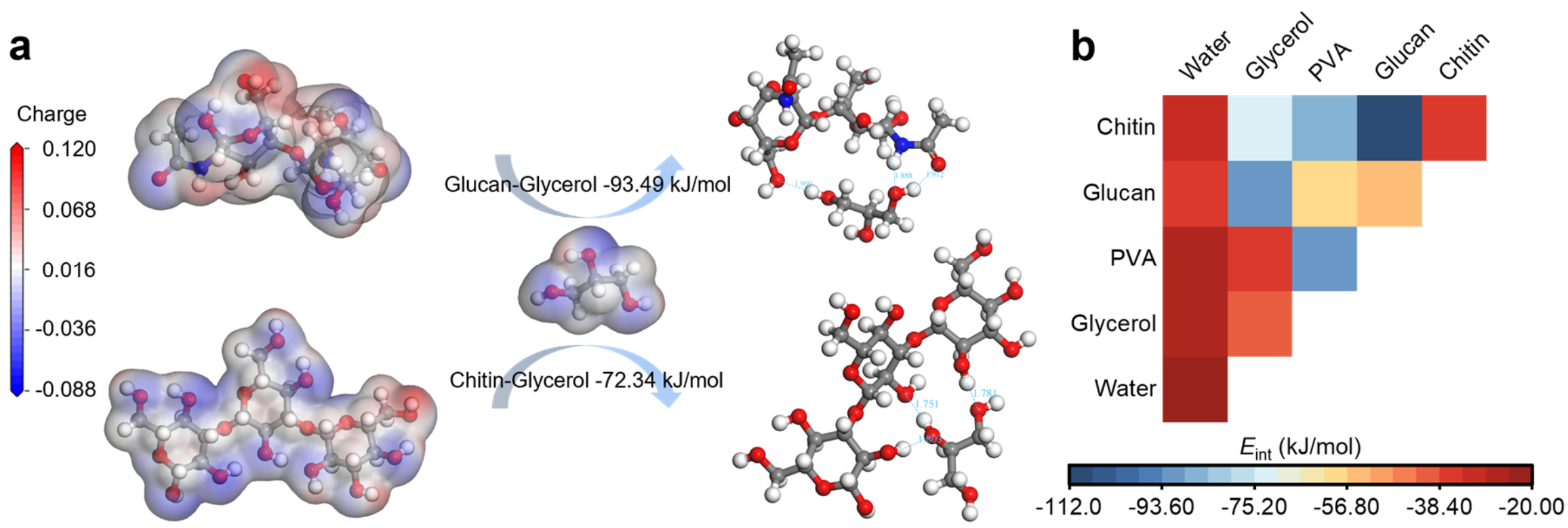

Supplementary Fig. 17 DFT calculation results for toughness modulation mechanism. **a**, Electrostatic potential (ESP) mapping of chitin, glucan and glycerol. Interactions between chitin, glucan, and glycerol, respectively, according to DFT optimization are illustrated. **b**, Comparison of DFT calculated intermolecular/intramolecular interactions energies in TV@P-G.

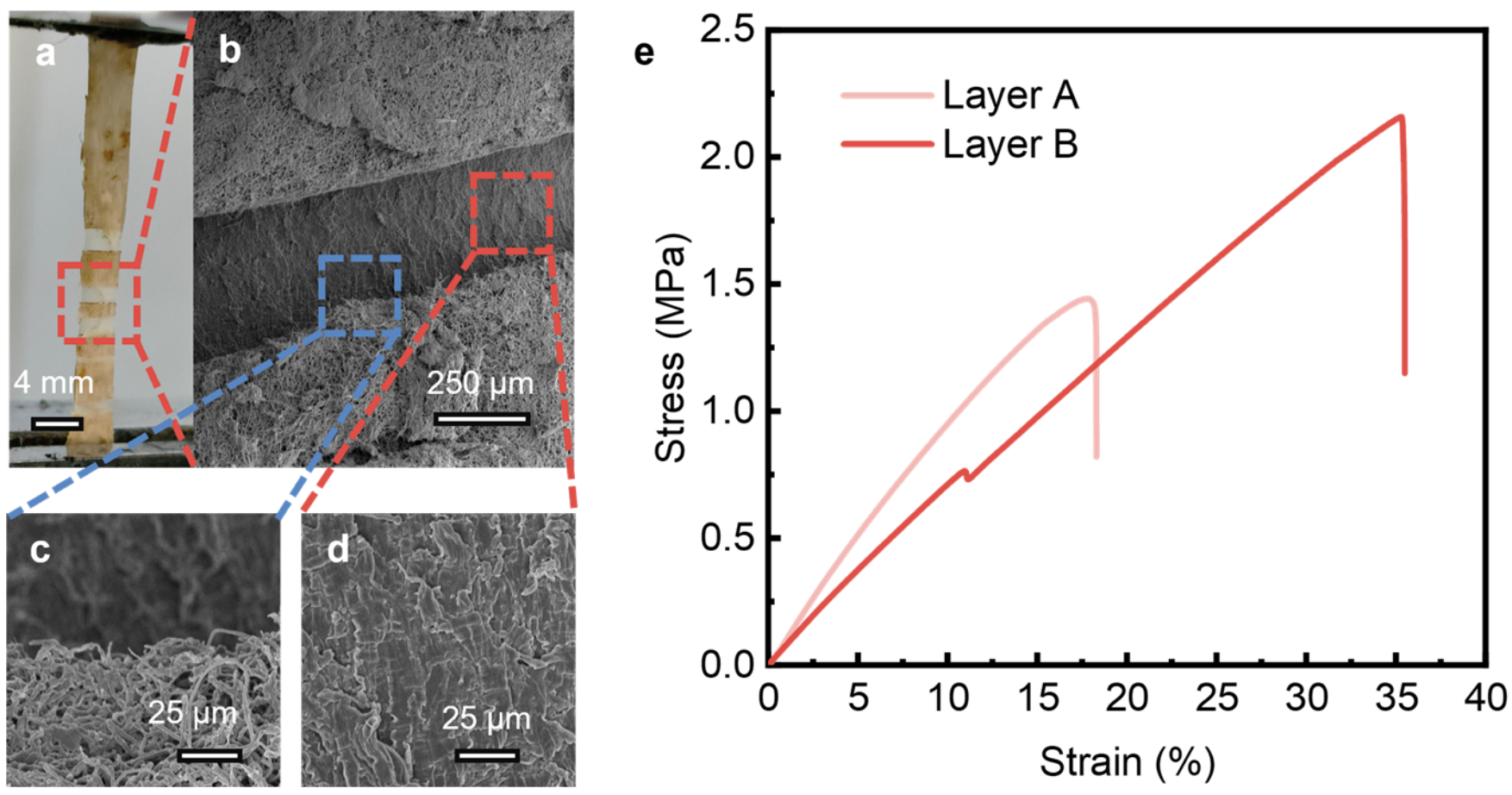

Supplementary Fig. 18 Mechanical asymmetry of mycelium macerate. **a**, Optical image demonstrates that layer A and B of TV 10%PVA+10%Gly specimen for tensile test broke separately. **b**, SEM image of non-simultaneous crack of partially broken TV 10%PVA+10%Gly specimen. **c**, **d**, Local amplified SEM image of **b**, displaying stretched layer A (**c**) and layer B (**d**). **e**, Tensile stress-strain curves of separated layer A and layer B.

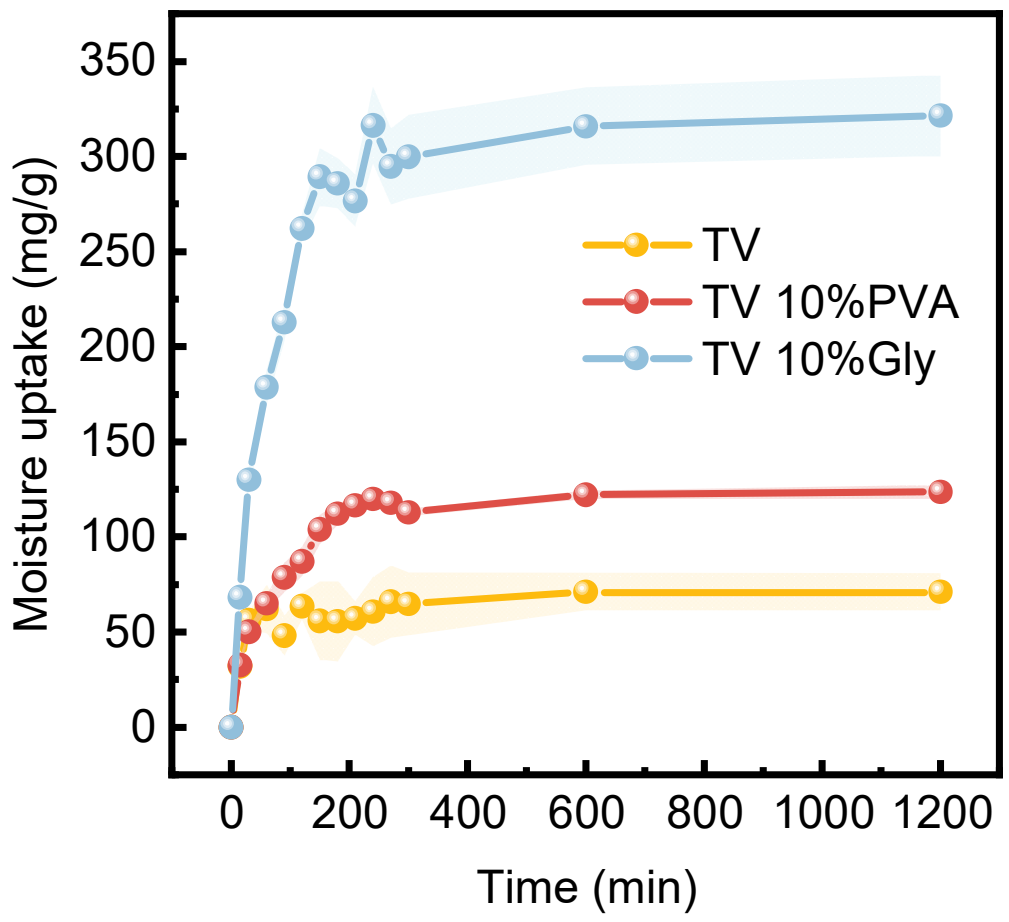

Supplementary Fig. 19 Sorption kinetics curves of control groups, TV, TV 10%PVA, TV 10%Gly, n = 3. Error bars (mean ± SD) were connected and visualized as a shadow effect around the data points.

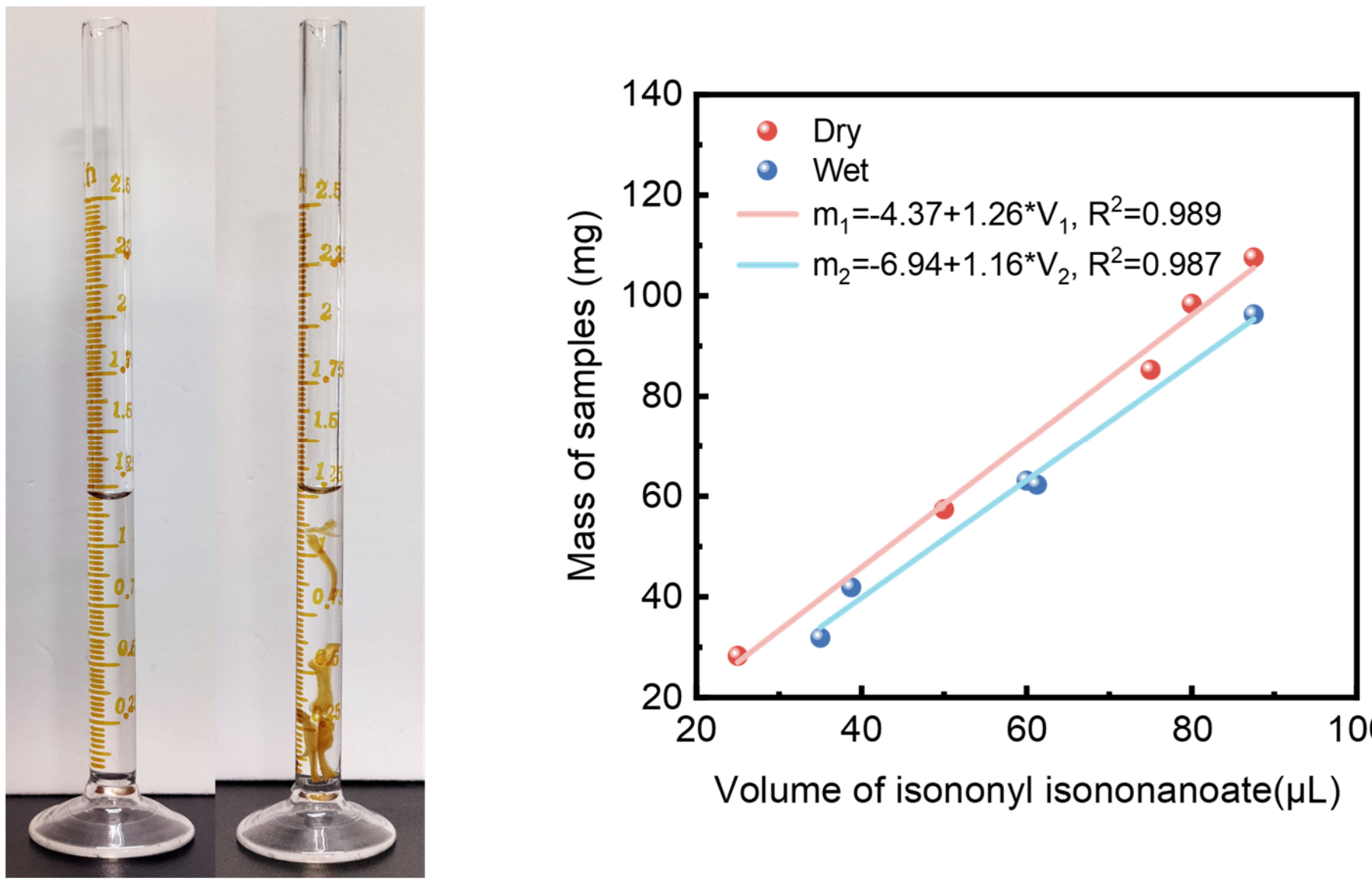

Supplementary Fig. 20 Archimedes method to test the density of dry (25℃, 20% RH) and wet (25℃, 85% RH) TV 10%PVA+10%Gly. Isononyl isononanoate was used to measure volume of membrane samples.

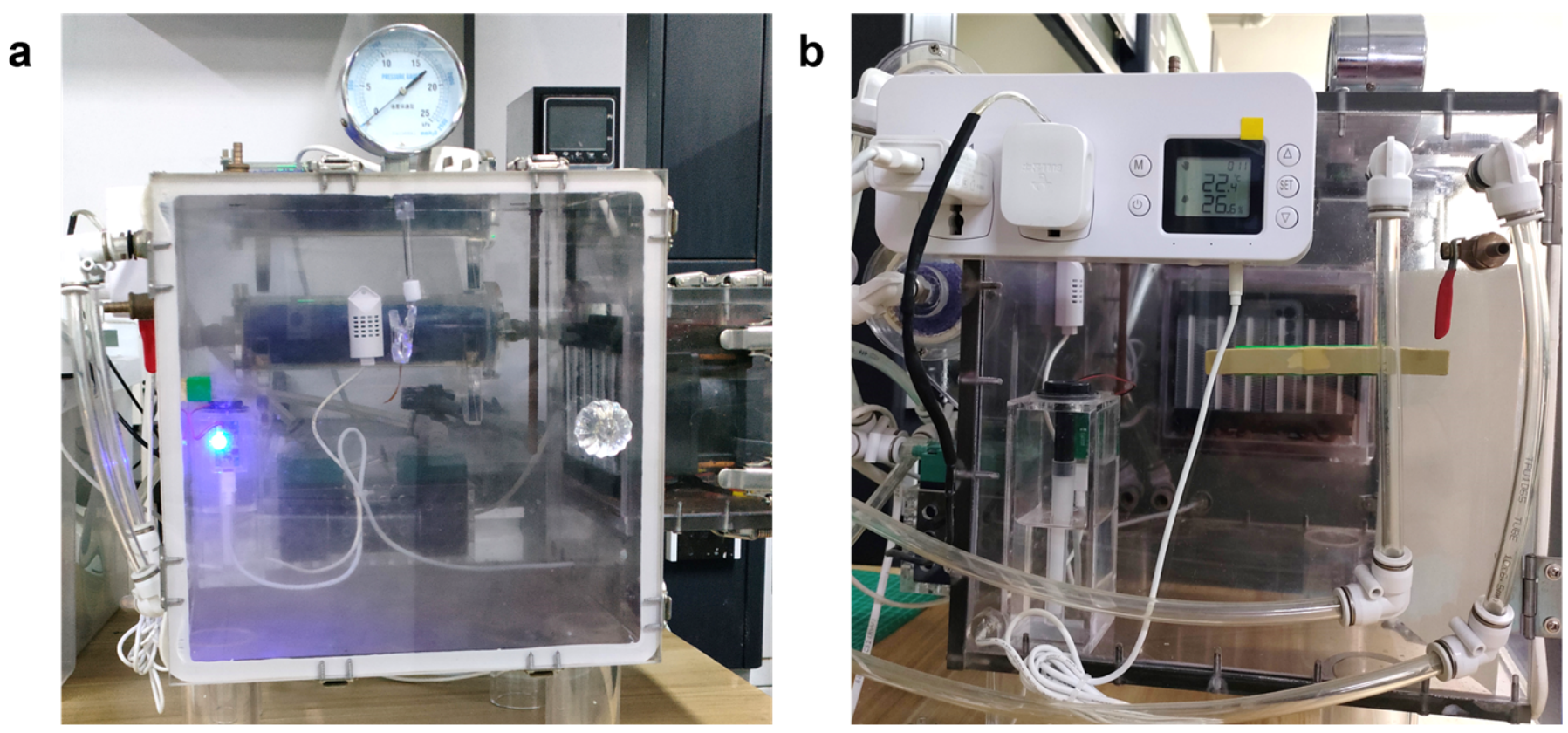

Supplementary Fig. 21 Optical images of the humidity chamber with inner humidity visualization, which was used to control the humidity and create a relatively homogeneous environment in this work. **a**, Front view. **b**, Left view with internal humidity shown.

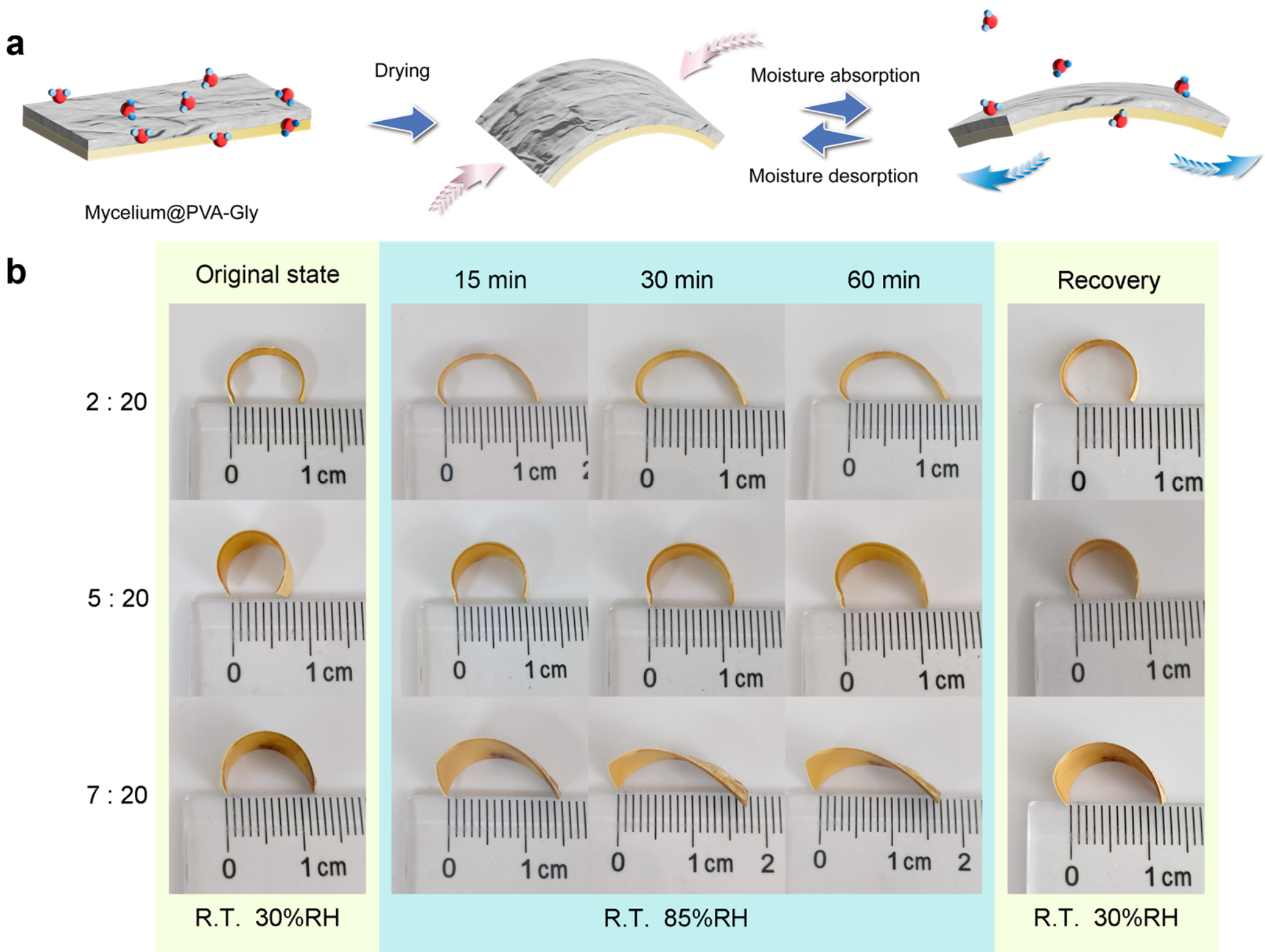

Supplementary Fig. 22 Untethered hygroscopic deformation of mycelium macerate. Schematic diagram (**a**) of the Janus structure causing deformation and optical images (**b**) showing hygroscopic deformation process of untethered Janus TV@P-G with different aspect ratios. The experiment was repeated independently three times (n = 3) with similar trends, and a representative experiment is shown.

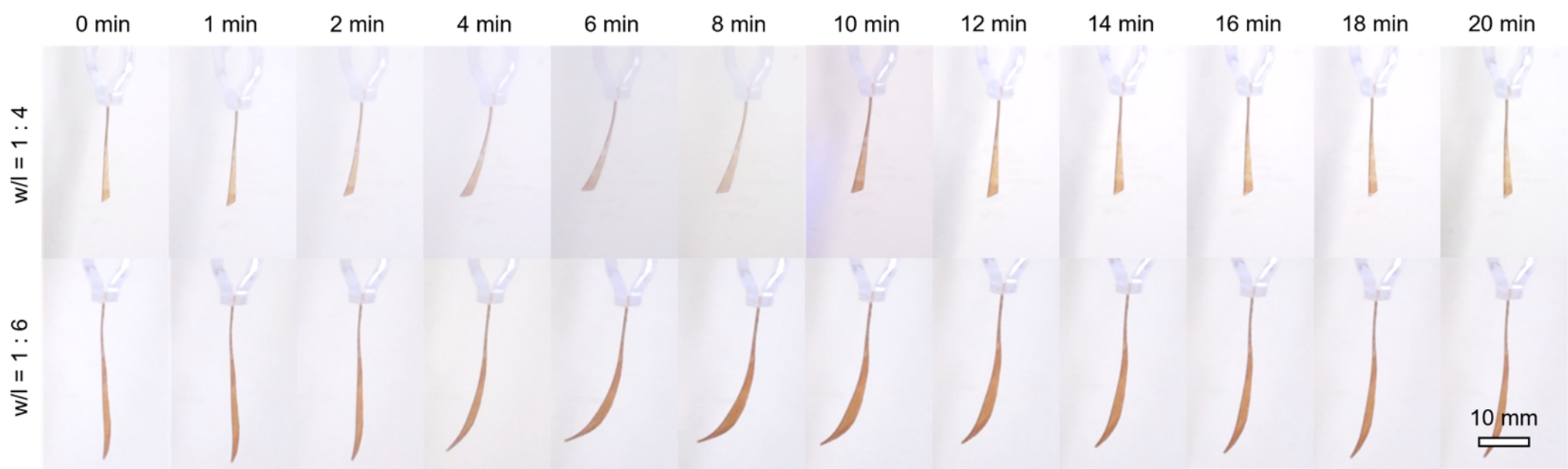

Supplementary Fig. 23 Optical images of TV@P-G specimen (5 × 20, 5 × 30 mm) with single fixed end deforming in hygroscopic process controlled in a humidity chamber, where humidity changed from 20% RH to 85% RH in 8 min, and decreased in the next 12 min. The experiment was repeated independently three times (n = 3) with similar trends, and a representative experiment is shown.

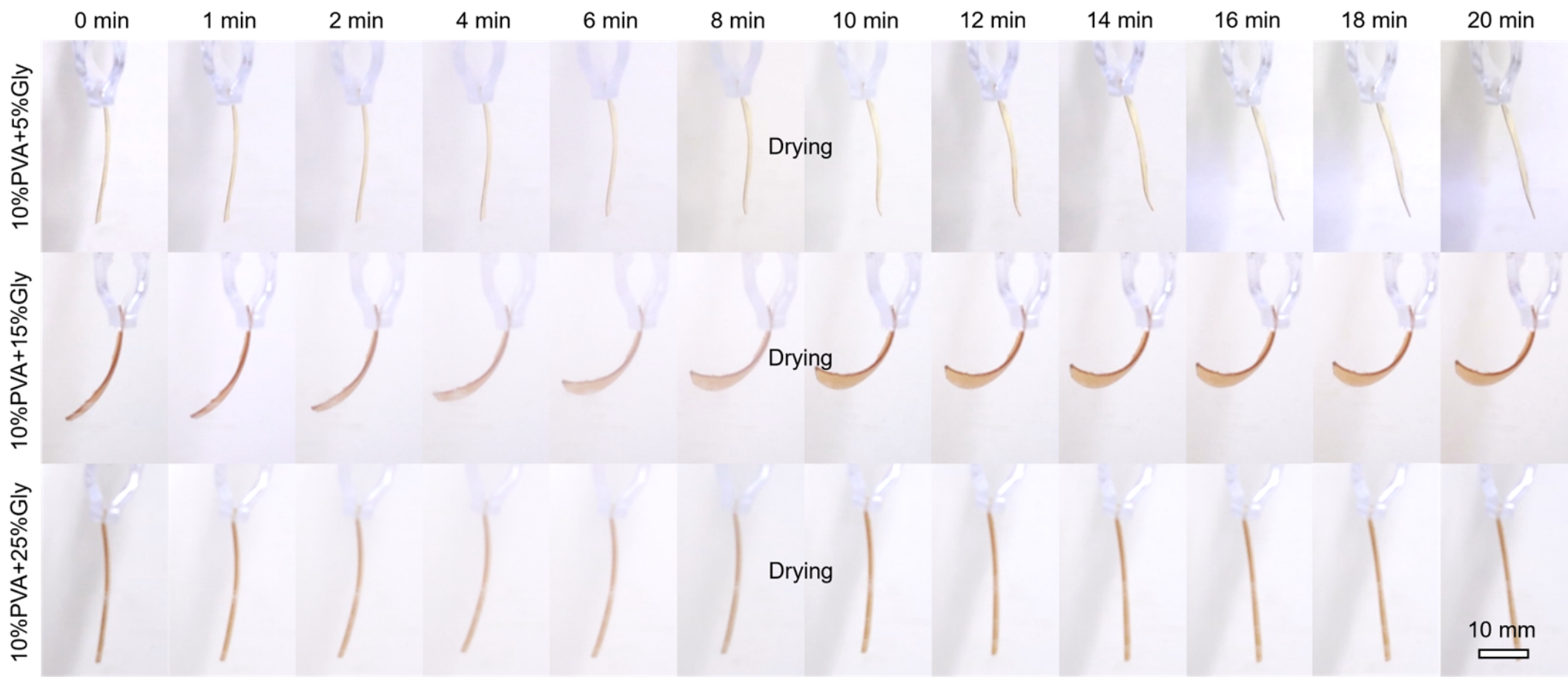

Supplementary Fig. 24 Optical images of various glycerol content treated TV@P-G specimen (5×25 mm) with single fixed end deforming in hygroscopic process controlled in a humidity chamber, where humidity changed from 20% RH to 85% RH in 8 min, and decreased in the next 12 min. The experiment was repeated independently three times (n = 3) with similar trends, and a representative experiment is shown.

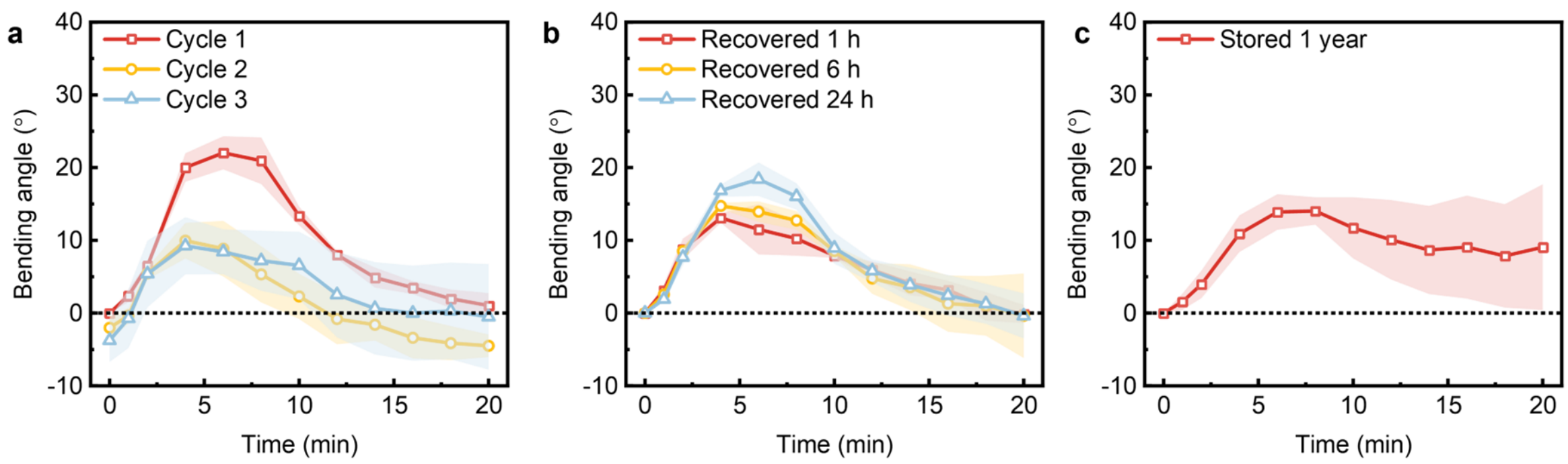

Supplementary Fig. 25 Repeatability and over-time performance of hygroscopic deformation. **a**, **b**, Cyclic bending angle changes of Janus TV@P-G (10%PVA+10%Gly) specimen (5 × 25 mm) in single fixed humidity response experiments, n = 3. Cycle 1 to 3 were continuous processes (**a**), while the "recovered cycles" were defined as the next cycle after 1-hour, 6-hour and 24-hour drying treatment to samples (**b**). **c**, Bending angle changes of Janus TV@P-G (10%PVA+10%Gly) specimen stored for 1 year (8℃, 60% RH), n = 3. Humidity was controlled with humidity chamber. Error bars (mean ± SD) were connected and visualized as a shadow effect around the data points.

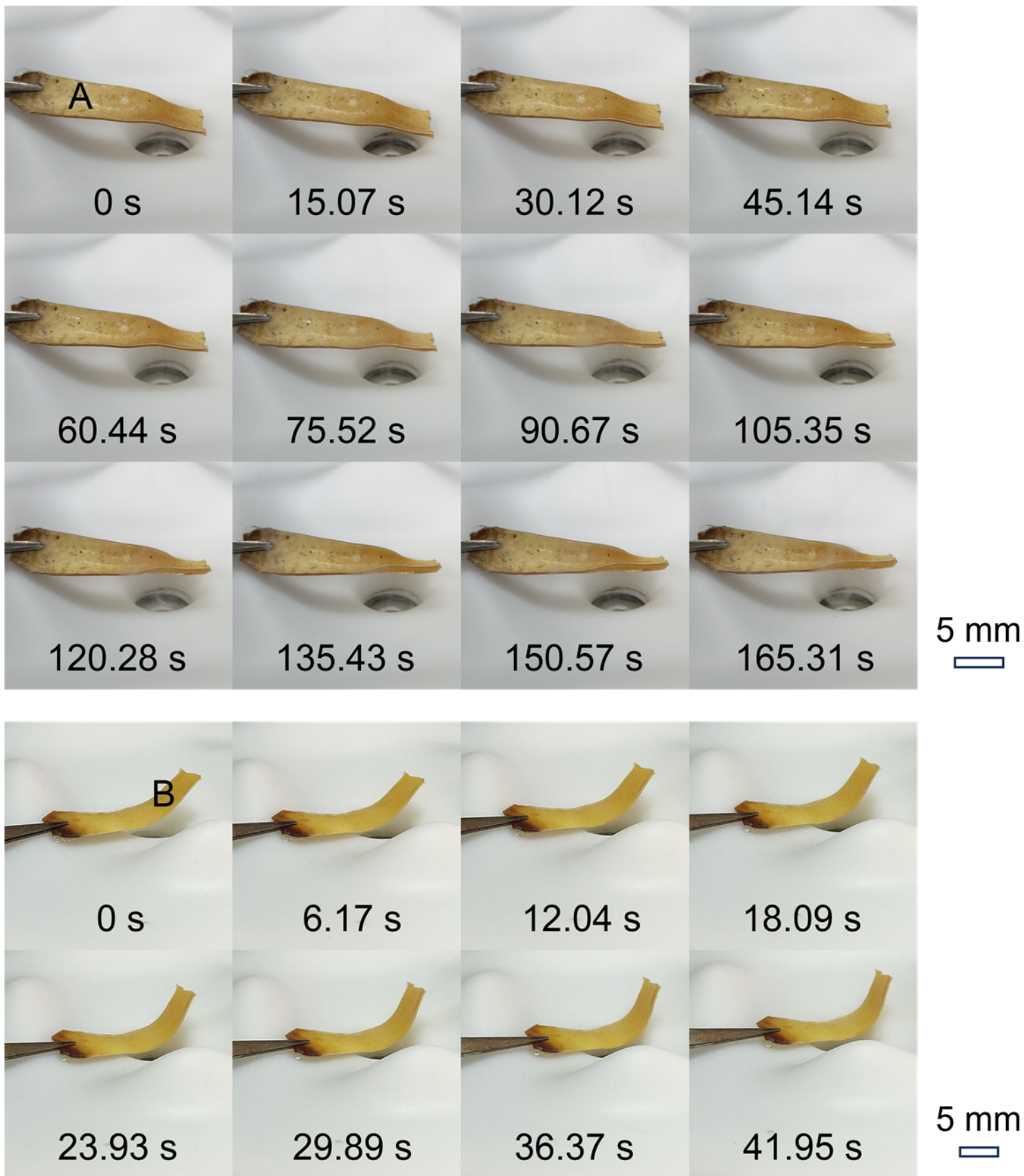

Supplementary Fig. 26 Hygroscopic deformation process of Janus TV@P-G with surface A or B towards the humidifier, respectively. The experiment was repeated independently three times (n = 3) with similar trends, and a representative experiment is shown.

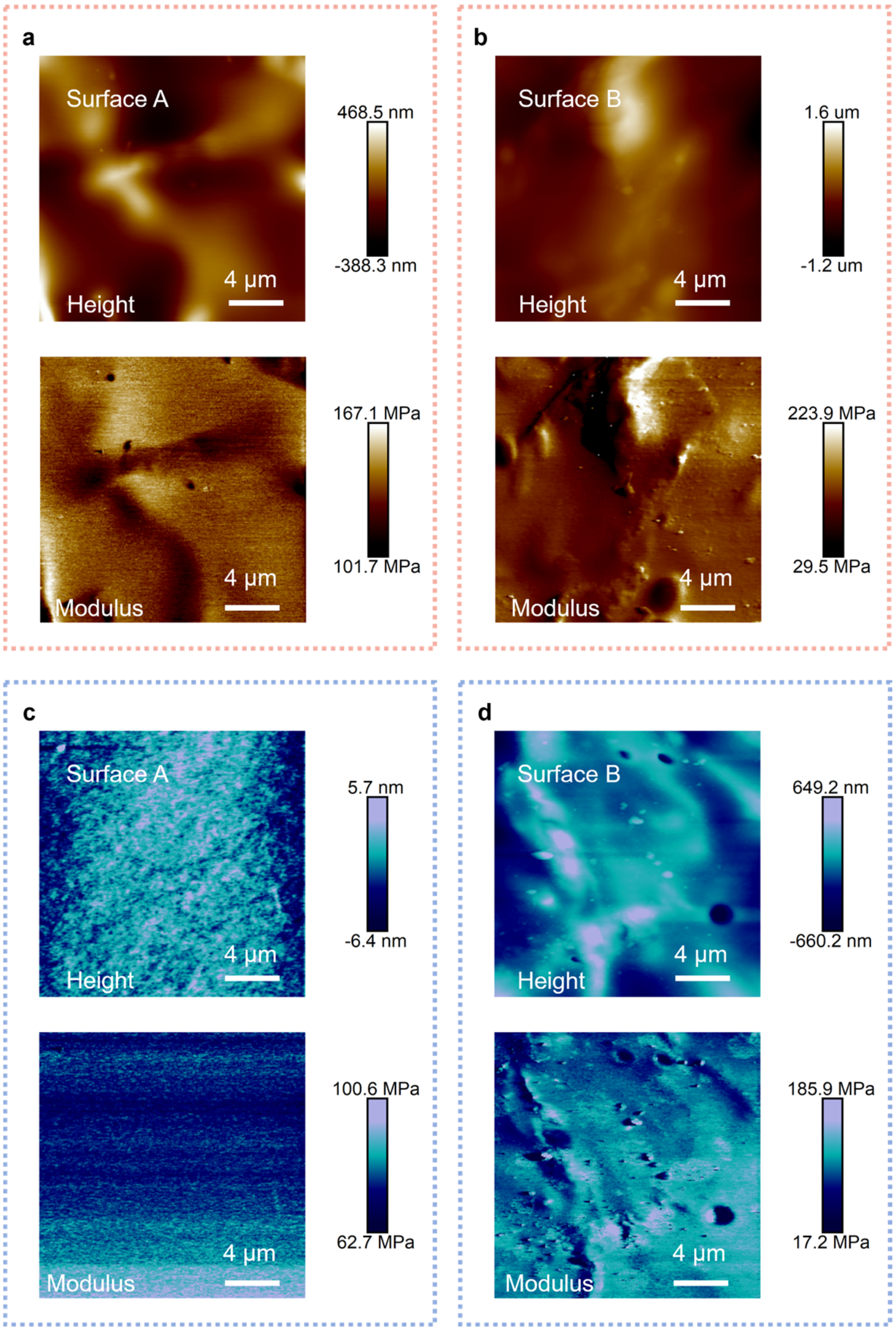

Supplementary Fig. 27 Asymmetry of mycelium macerate surfaces as observed by atomic force microscopy (AFM). AFM height and DMT modulus images of Janus TV 10%PVA+10%Gly in dry environment (R.T., 30%RH) and upon exposure to a wet environment (R.T., 85%RH), corresponding dry surface A (**a**), dry surface B (**b**), and wet surface A (**c**), wet surface B (**d**).

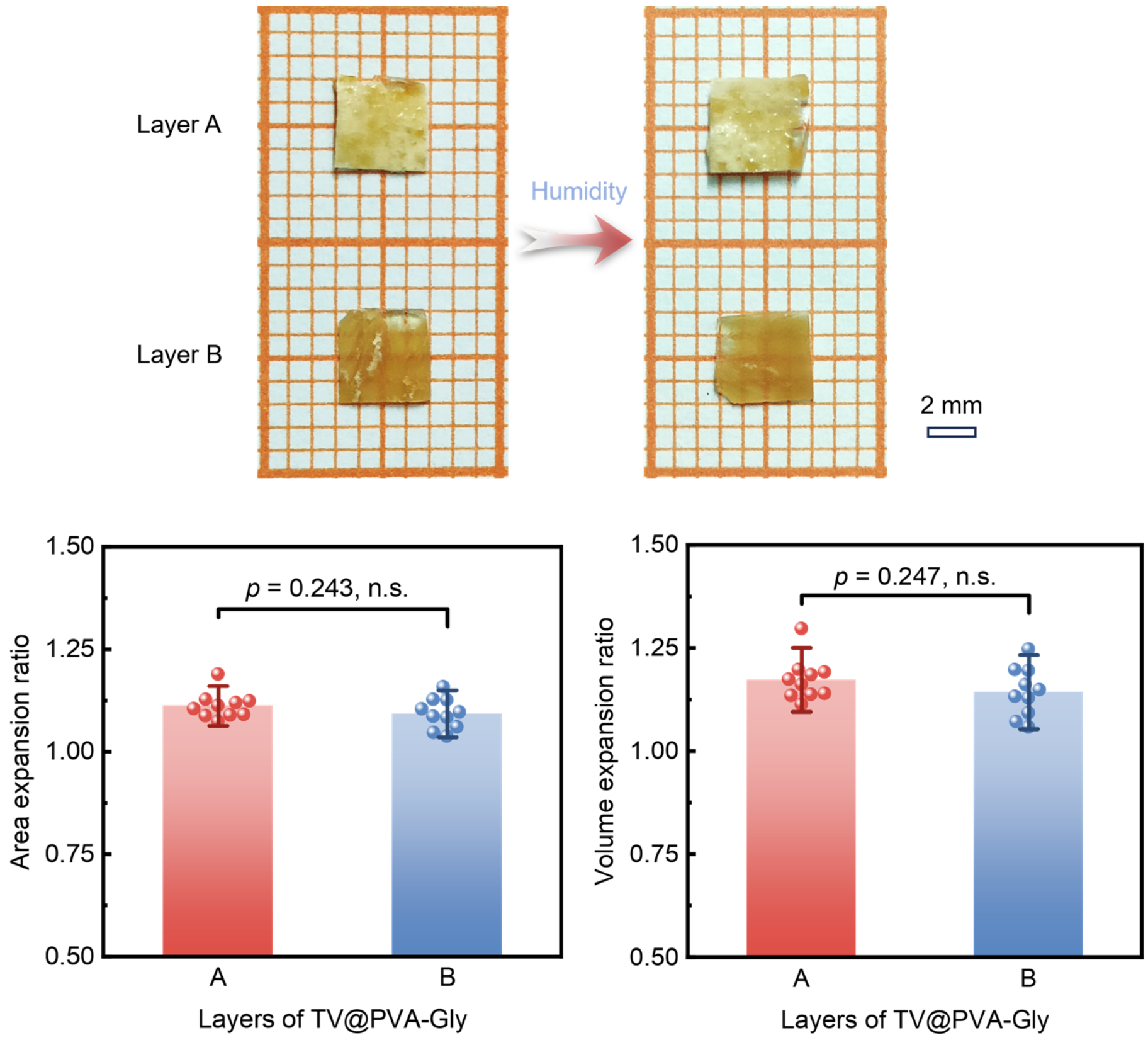

Supplementary Fig. 28 Statistics for comparison of expansion ratios of separated layer A and B using optical images of hygroscopic expansion, n = 10. Data are expressed as mean ± standard deviation (SD). A two-tailed t-test was used to compare similar data. For t-tests: Cohen's d was calculated as ($mean_1$ - $mean_2$) / pooled standard deviation. Difference in area expansion ratio: $p = 0.243$, Cohen' d = 0.57. Difference in volume expansion ratio: $p = 0.247$ Cohen' $d = 0.56$.

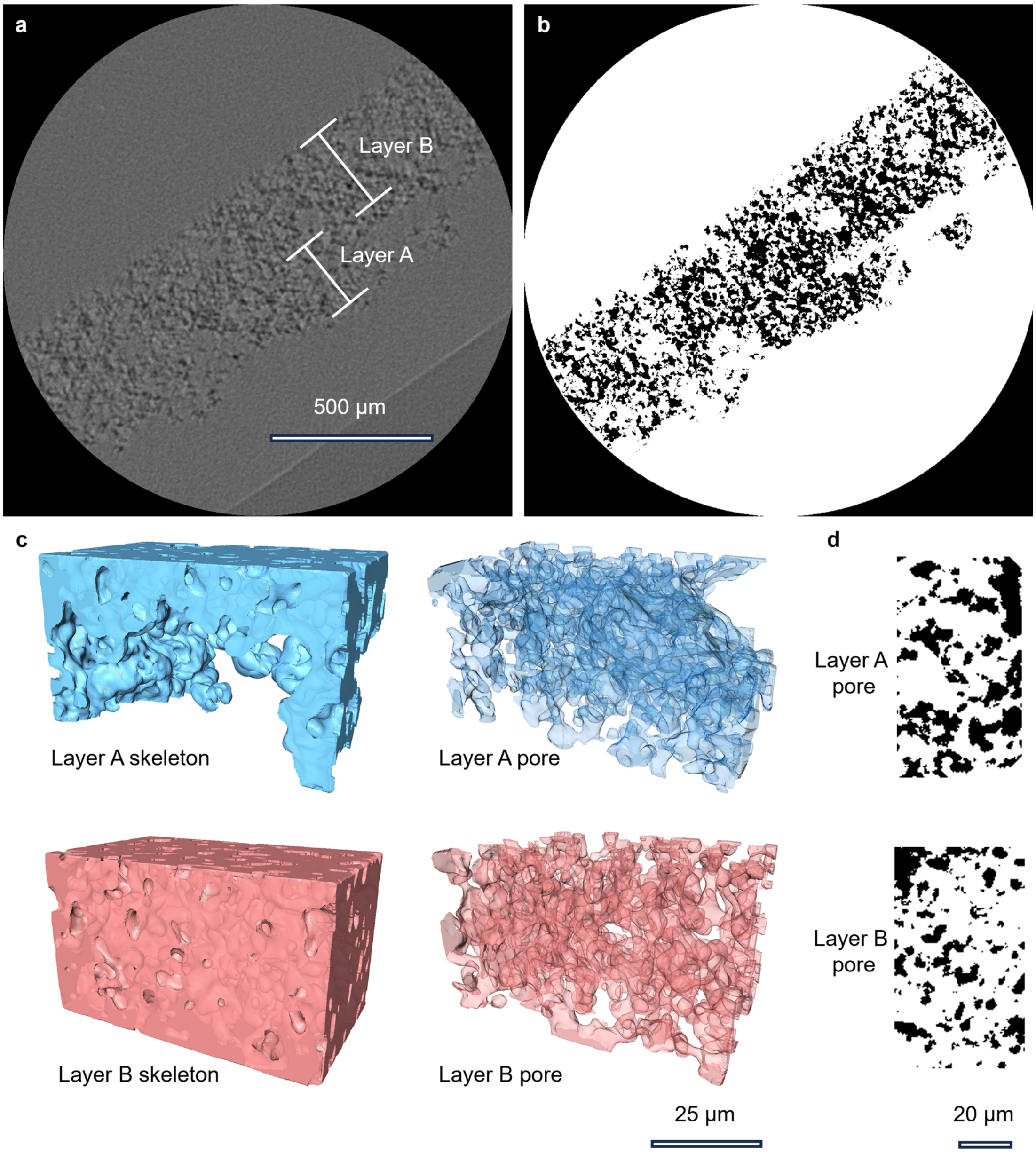

Supplementary Fig. 29 CT images of layer A and B in dry state. **a**, Original image of TV@P-G (R.T., 30%RH). **b**, Binarised image of TV@P-G. The black and white areas correspond to skeleton and pore respectively. **c**, Local magnified 3D CT images of skeleton and pore structure of separated layer A and B. **d**, Local magnified binarised images of separated layer A and B. The black areas correspond to pores.

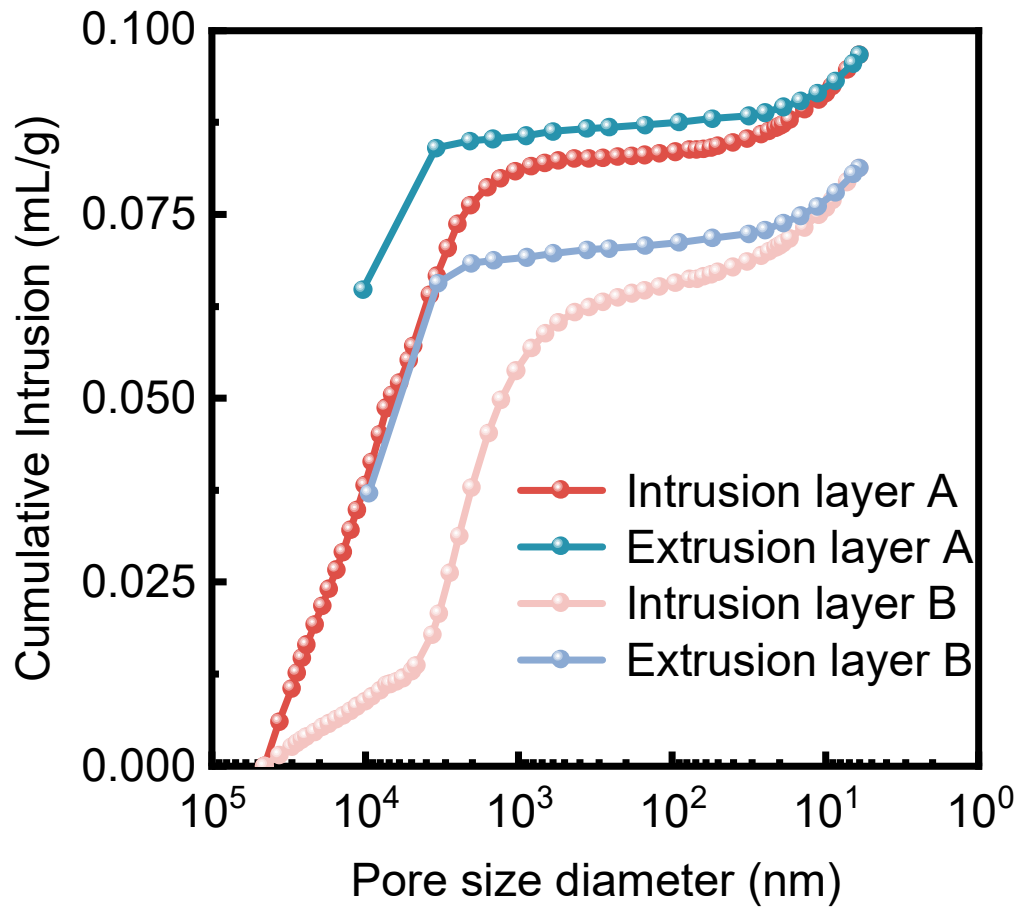

Supplementary Fig. 30 Cumulative intrusion and extrusion versus pore diameter for layer A and B.

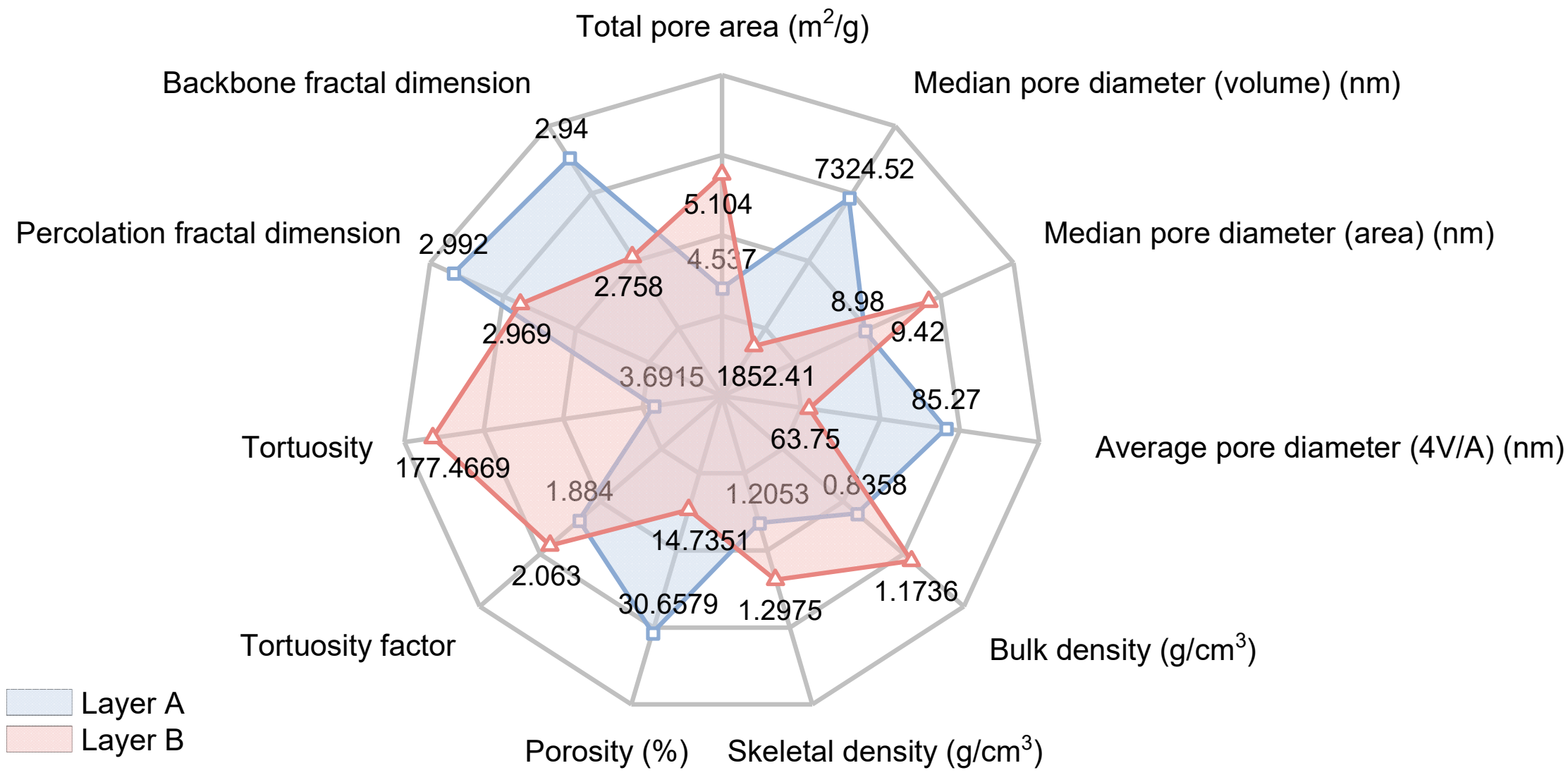

Supplementary Fig. 31 Comparison of pore structure parameters between layer A and B, measured with MIP.

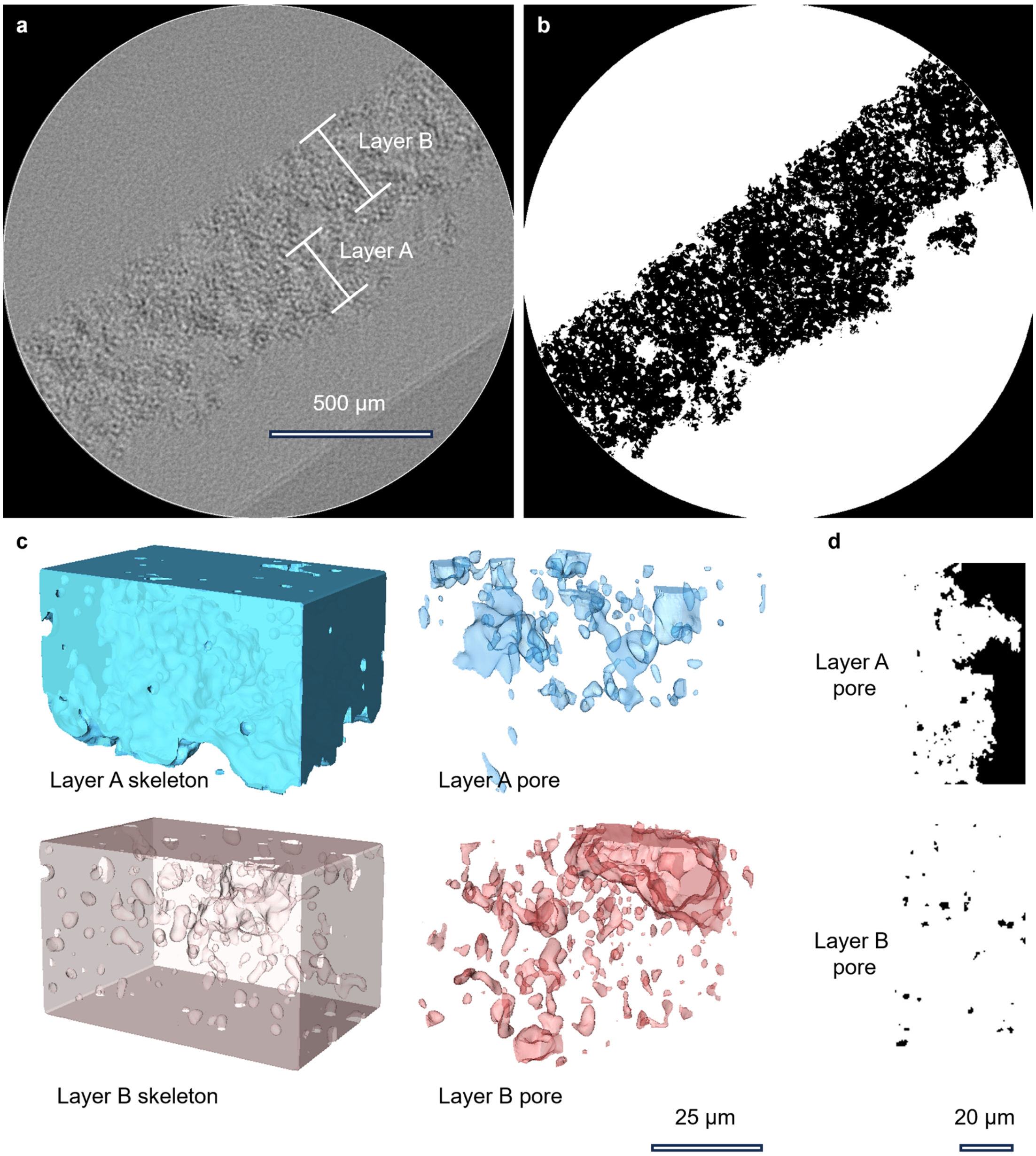

Supplementary Fig. 32 CT images of layer A and B in wet state. **a**, Original image of TV@P-G (R.T., 85%RH). **b**, Binarised image of TV@P-G. The black and white areas correspond to skeleton and pore respectively. **c**, Local magnified 3D CT images of skeleton and pore structure of separated layer A and B. **d**, Local magnified binarised images of separated layer A and B. The black areas correspond to pore.

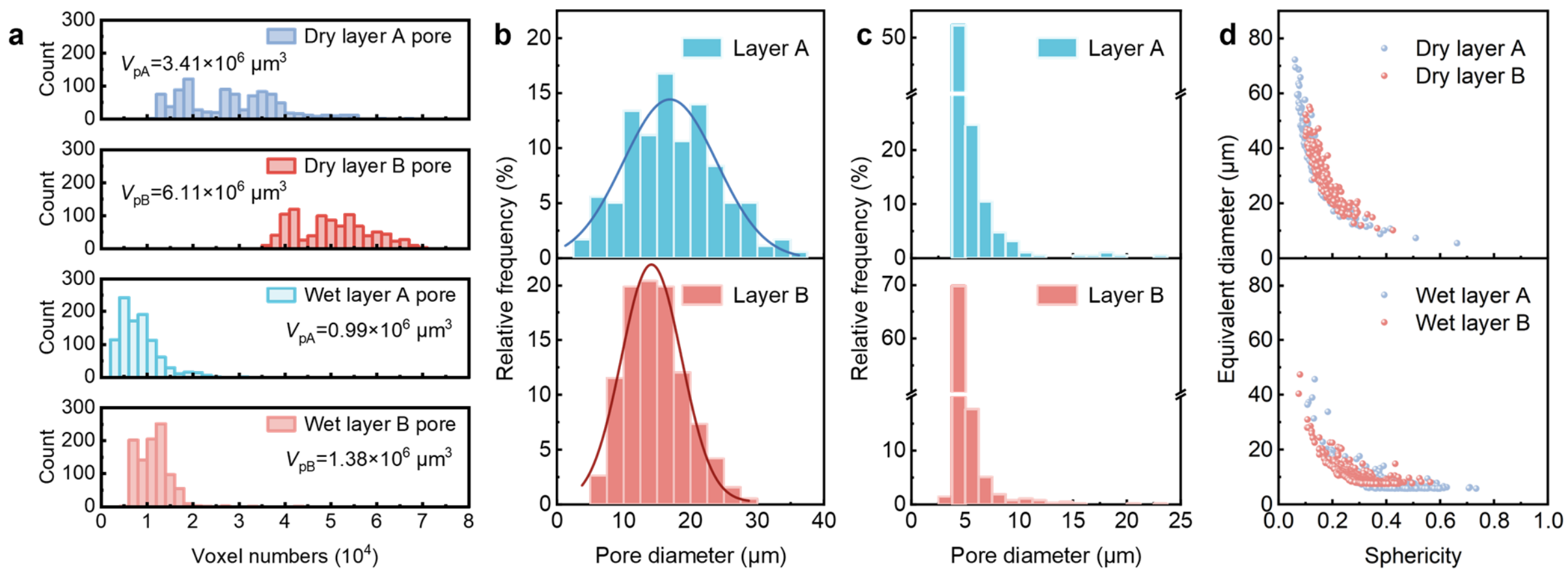

Supplementary Fig. 33 Analysis on pore structure transformation of Janus mycelium macerate from dry to wet state by micro-CT. **a**, Frame-wise statistics for voxel numbers of layer A and B pore structure in dry and wet states. **b**, **c**, Pore size distribution of layer A and B in dry (b) and wet (c) state. d, Pore diameter versus sphericity for layer A and B in dry and wet state.

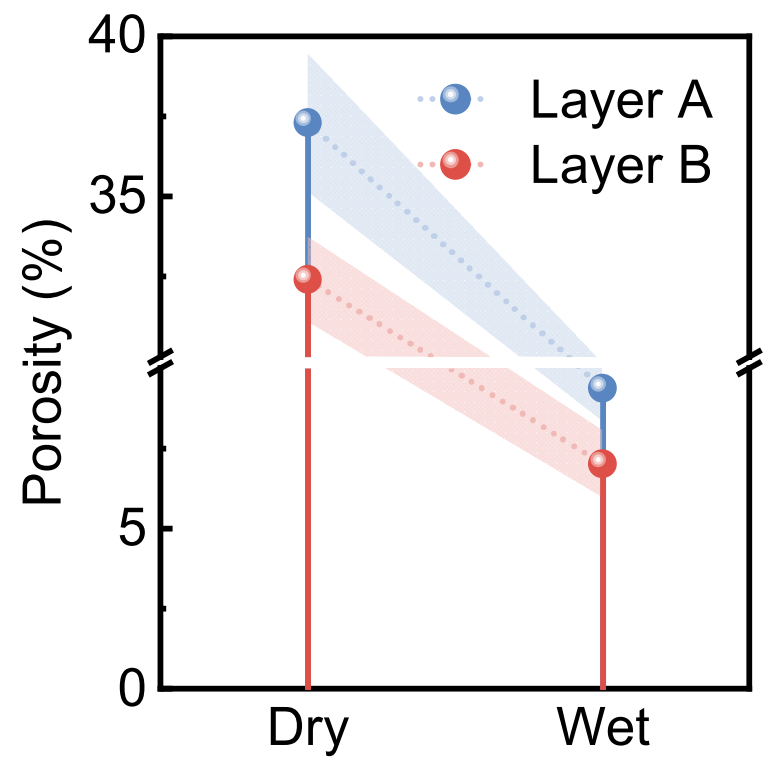

Supplementary Fig. 34 Porosity asymmetry of layer A and B from dry (R.T., 30%RH) to wet (R.T., 85%RH) state. Data are expressed as mean ± standard deviation (SD), n = 3.

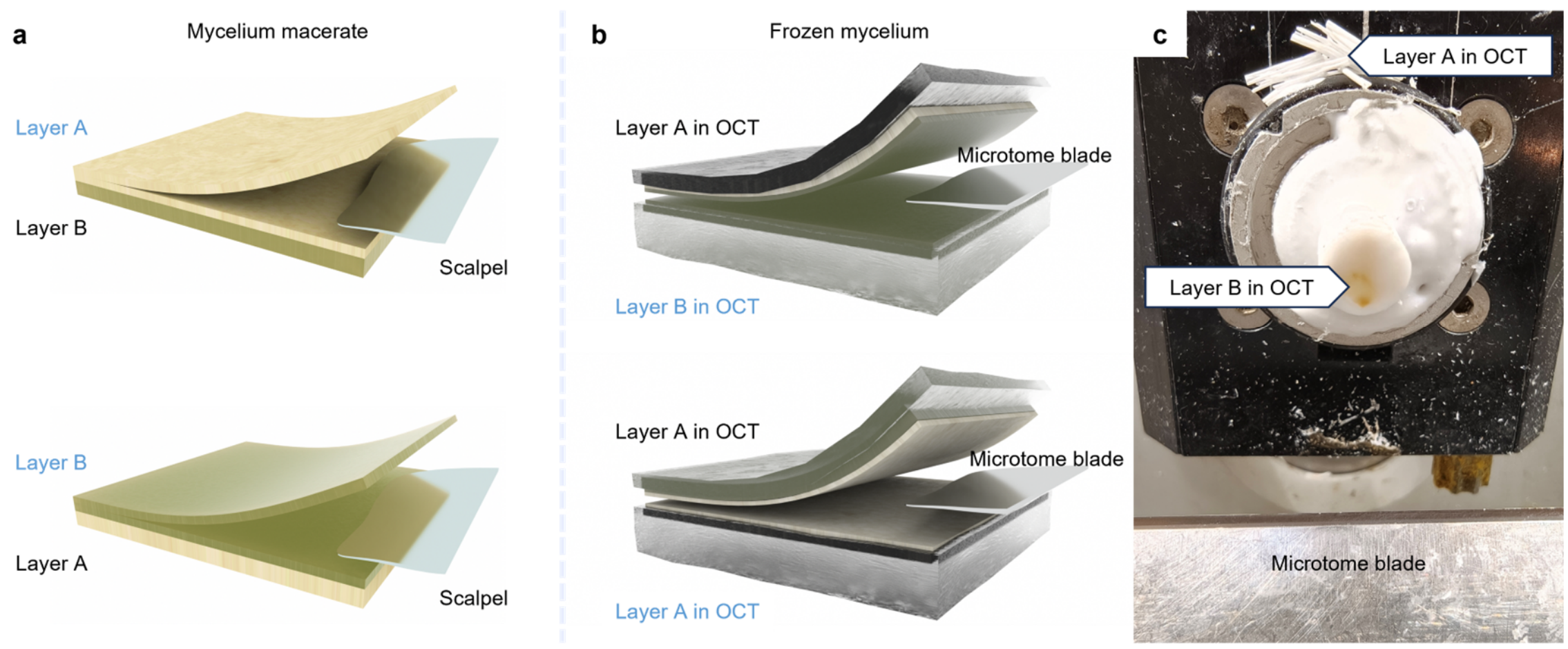

Supplementary Fig. 35 Layer separation methods. **a**, **b**, Schematic illustration for layer separation of mycelium macerate (**a**) and frozen (fresh) mycelium membrane (**b**), respectively. **c**, Optical image of peeling off layer A using a freezing microtome.

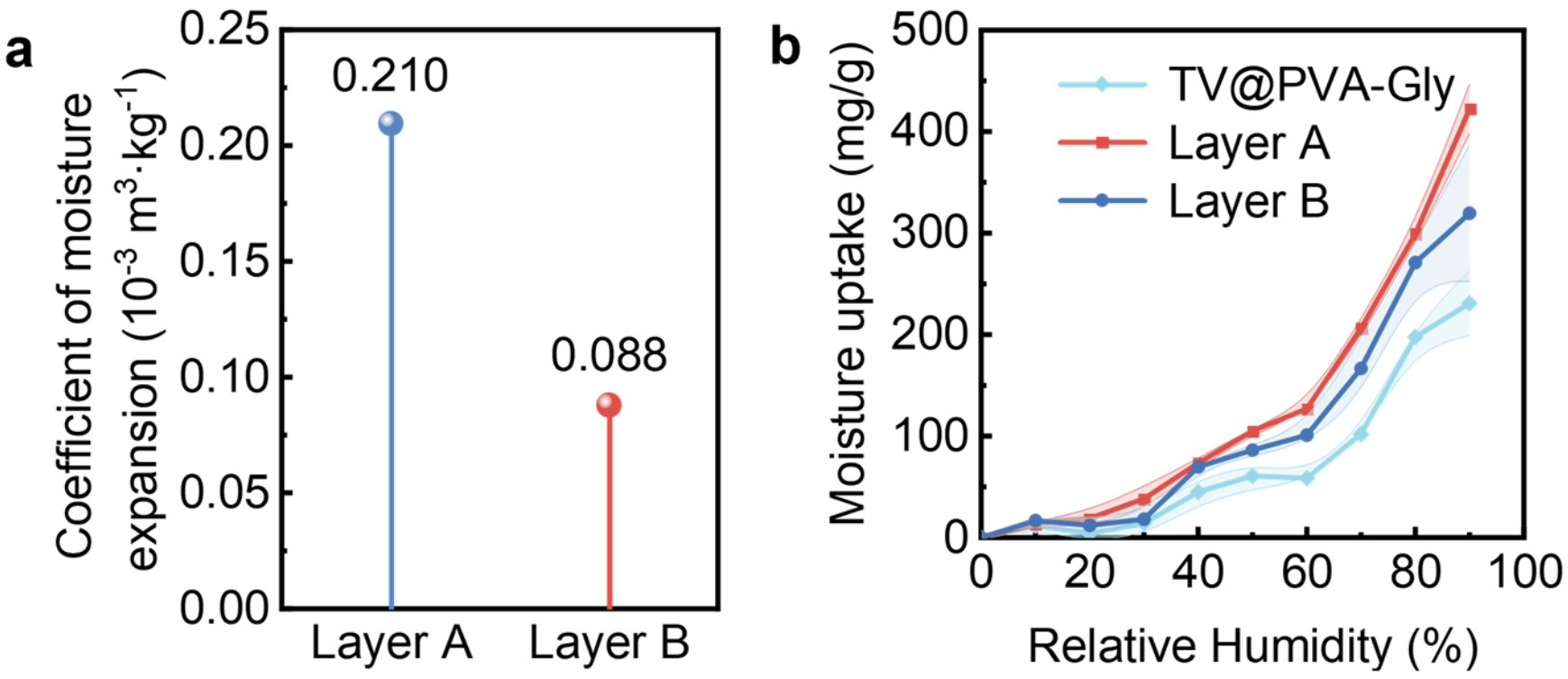

Supplementary Fig. 36 Differences of Janus structure in moisture uptake and coefficient of moisture expansion. **a**, Calculated coefficient of moisture expansion of layer A and B saturated moisture uptake upon exposure to the wet environment (R.T., 85%RH). **b**, DVS curves of TV@P-G, separated layer A and B, n = 3. Error bars (mean ± SD) were connected and visualized as a shadow effect around the data points.

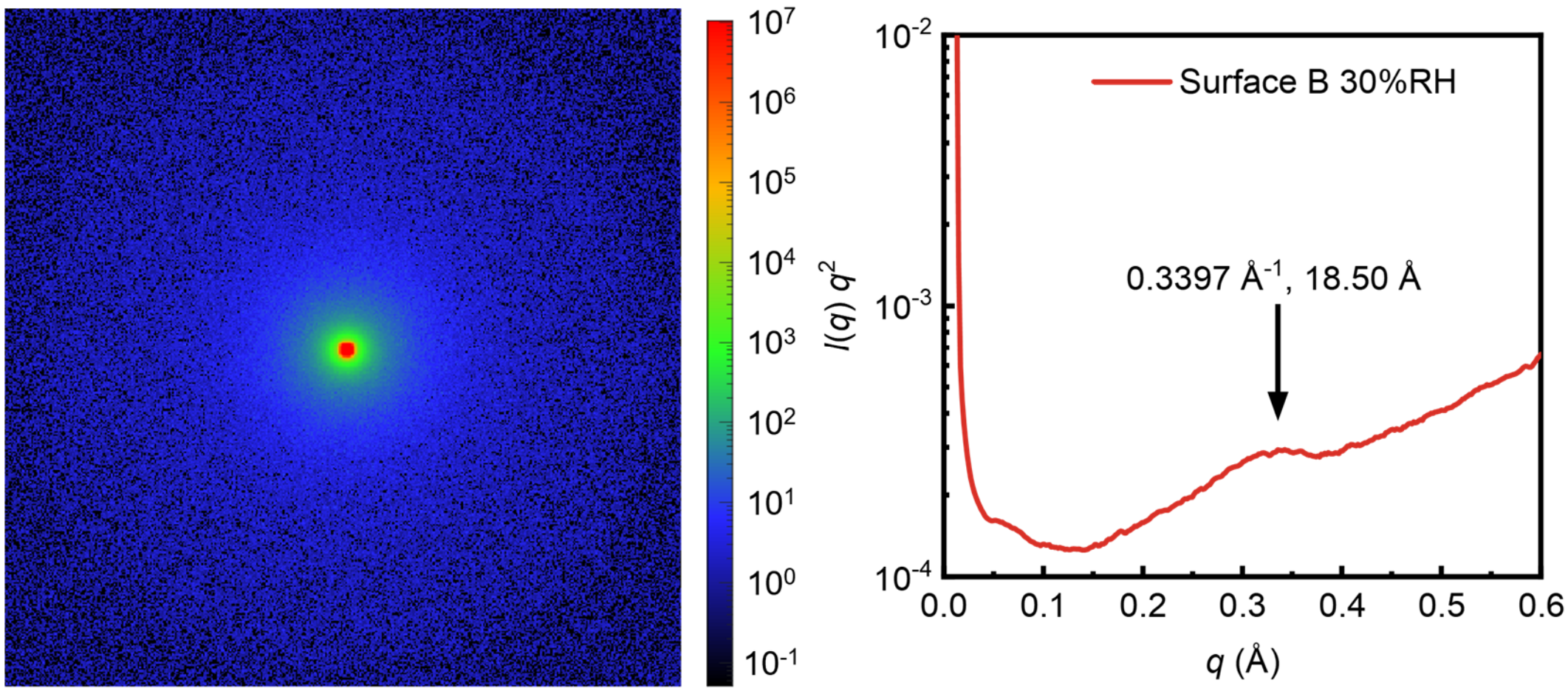

Supplementary Fig. 37 2D SAXS pattern and Lorentz-corrected 1D SAXS curve of surface B in the dry environment (R.T., 30%RH).

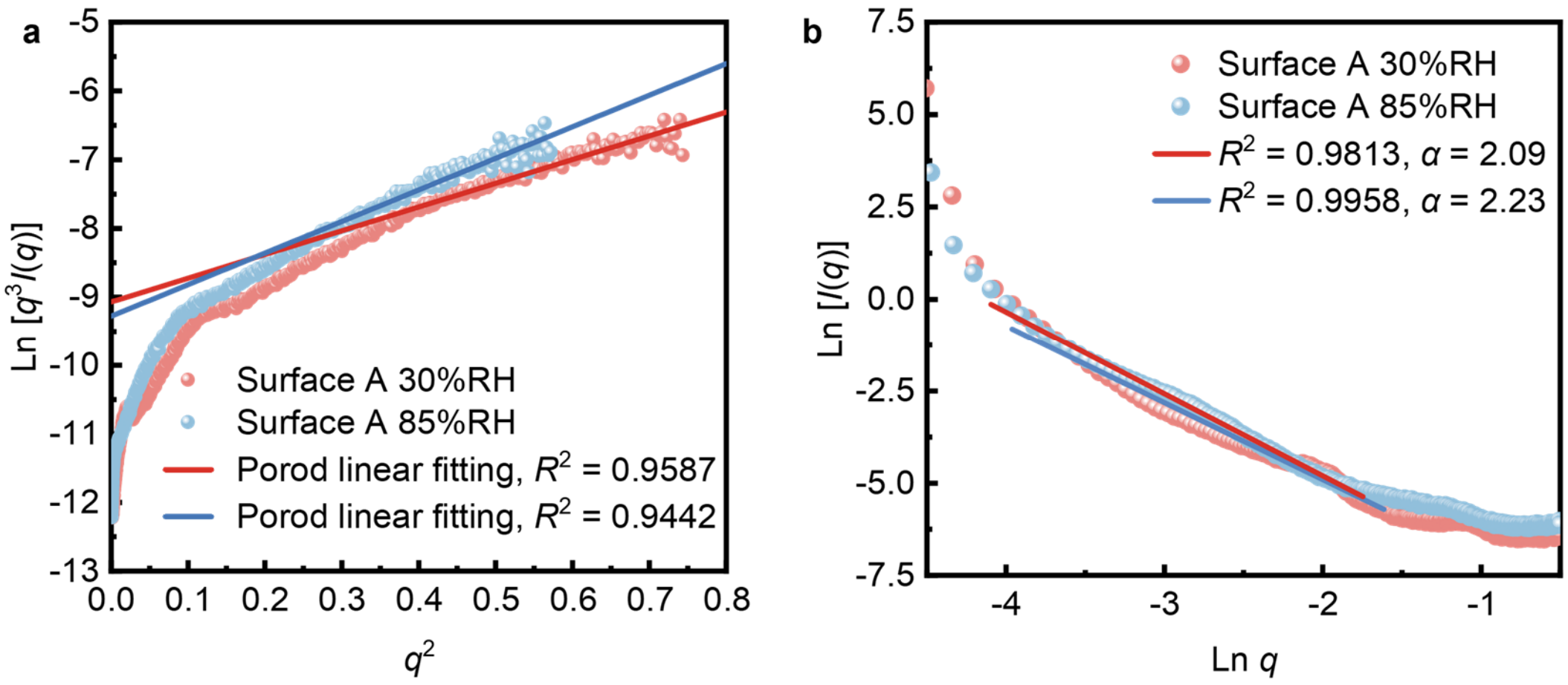

Supplementary Fig. 38 Humidity response of mycelium macerate revealed by SAXS analysis on surface A. **a**, Porod fitting of 1D SAXS data of surface A upon exposure to dry and wet environment. **b**, Fractal dimension analyzed from 1D SAXS data.

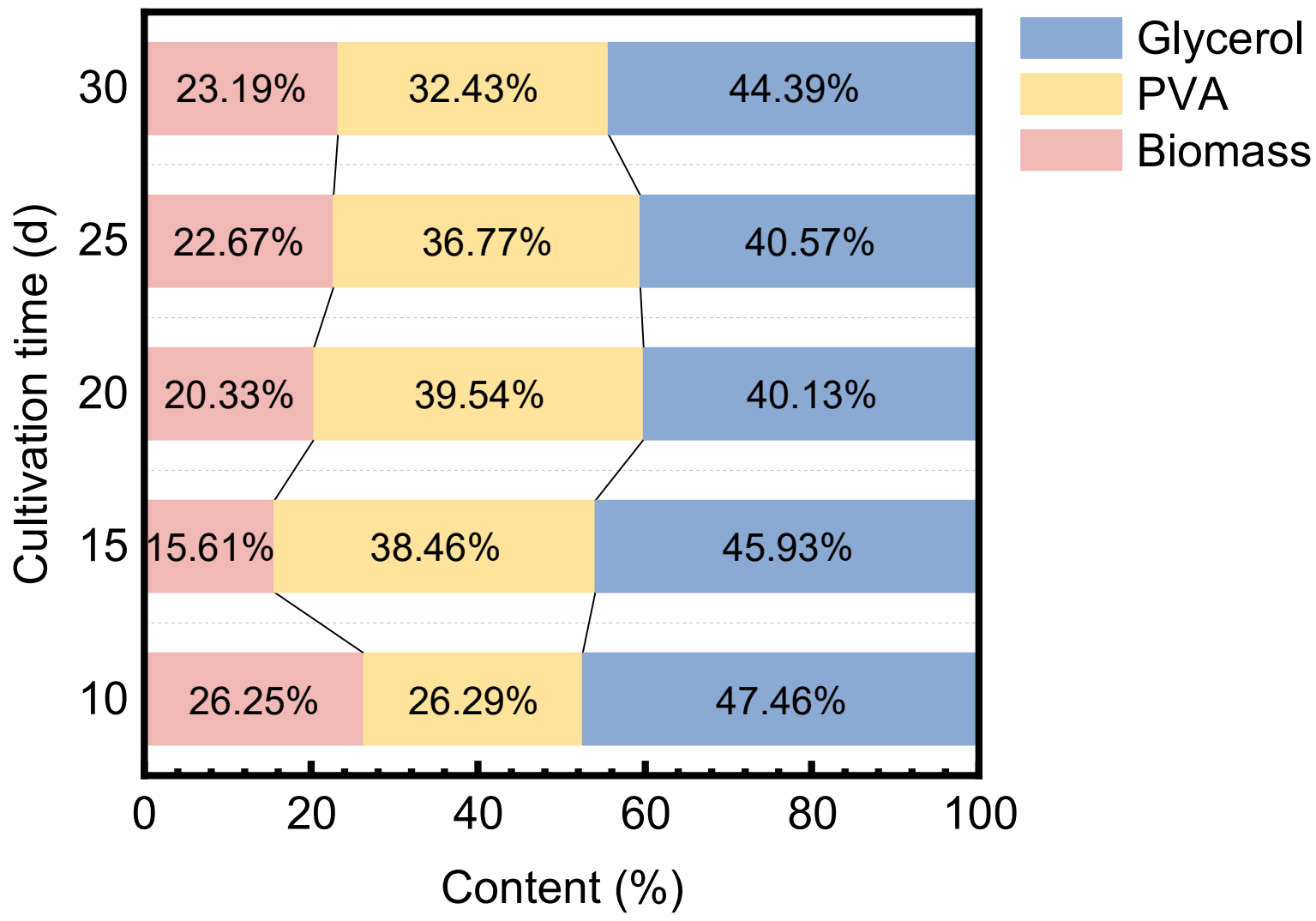

Supplementary Fig. 39 Proportion of mycelium biomass, PVA and glycerol in TV@P-G with different cultivation time, measured by temperature gradient dissolution, n = 3.

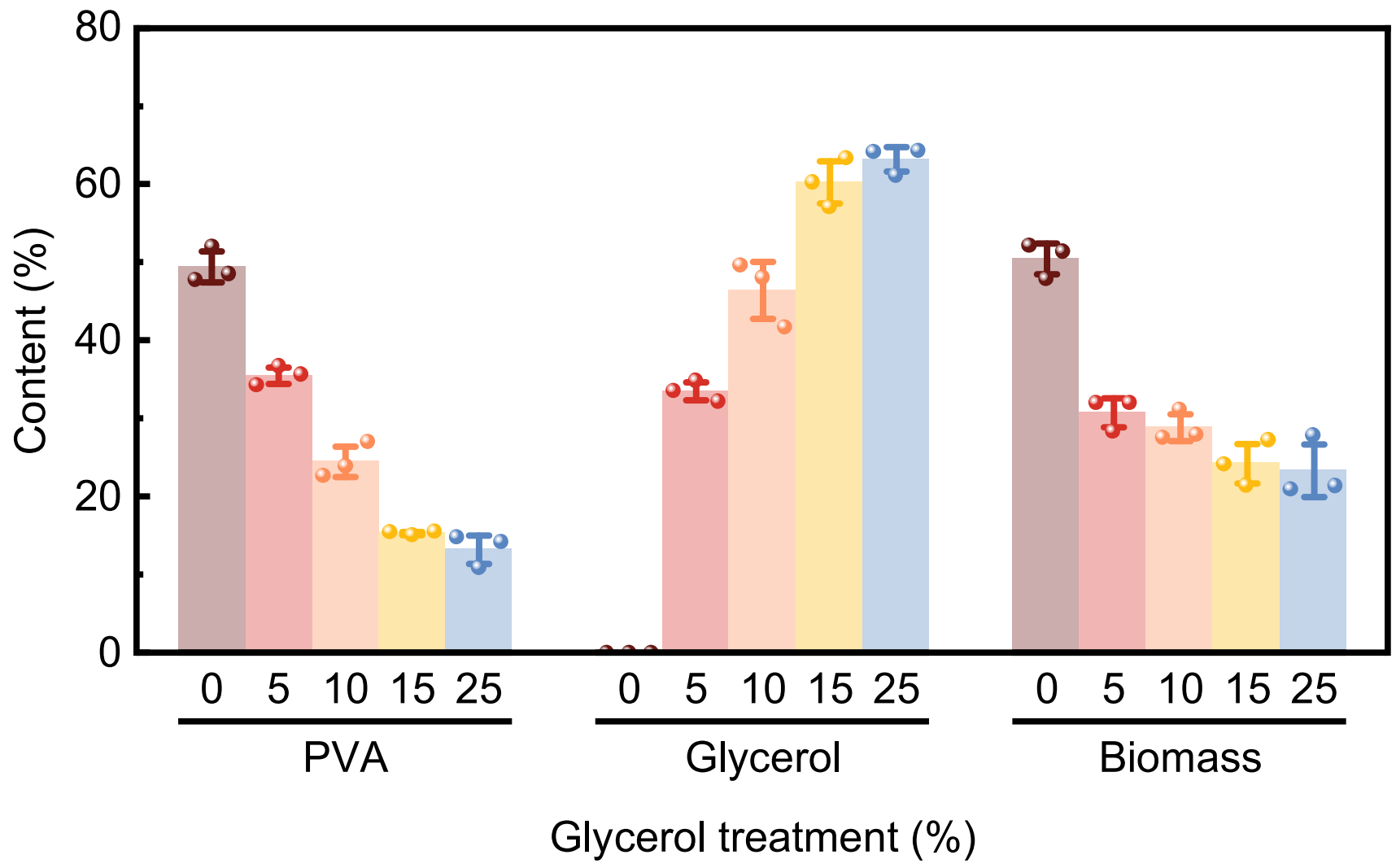

Supplementary Fig. 40 Percentage composition of PVA, Glycerol and biomass in TV@P-G fabricated through treatment with 0-25% glycerol, n = 3. Error bars represented mean ± SD.

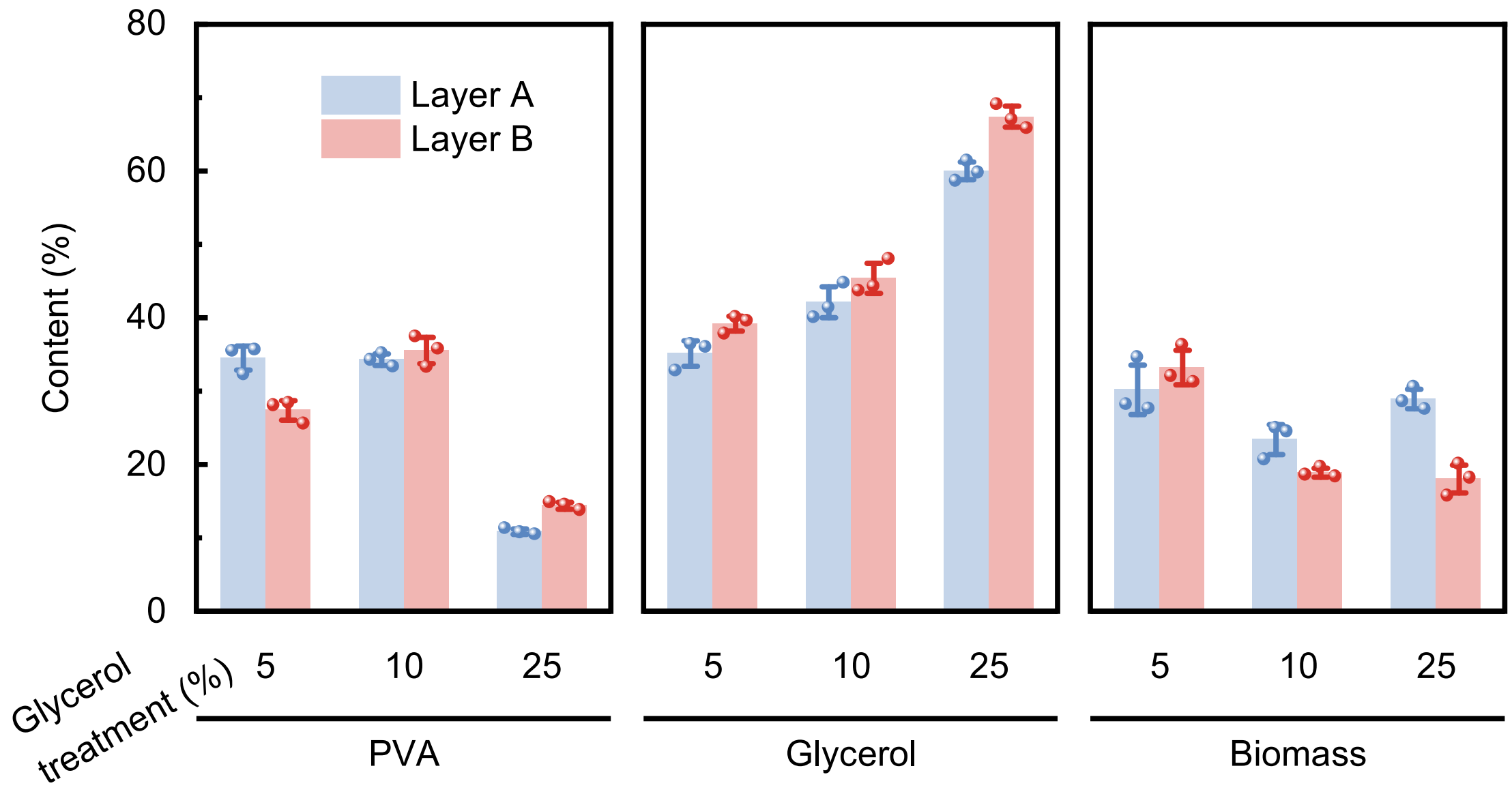

Supplementary Fig. 41 Percentage composition of PVA, Glycerol and biomass in layer A and B (dried) of TV@P-G fabricated through treatment with 5-25% glycerol, n = 3. Error bars represented mean ± SD.

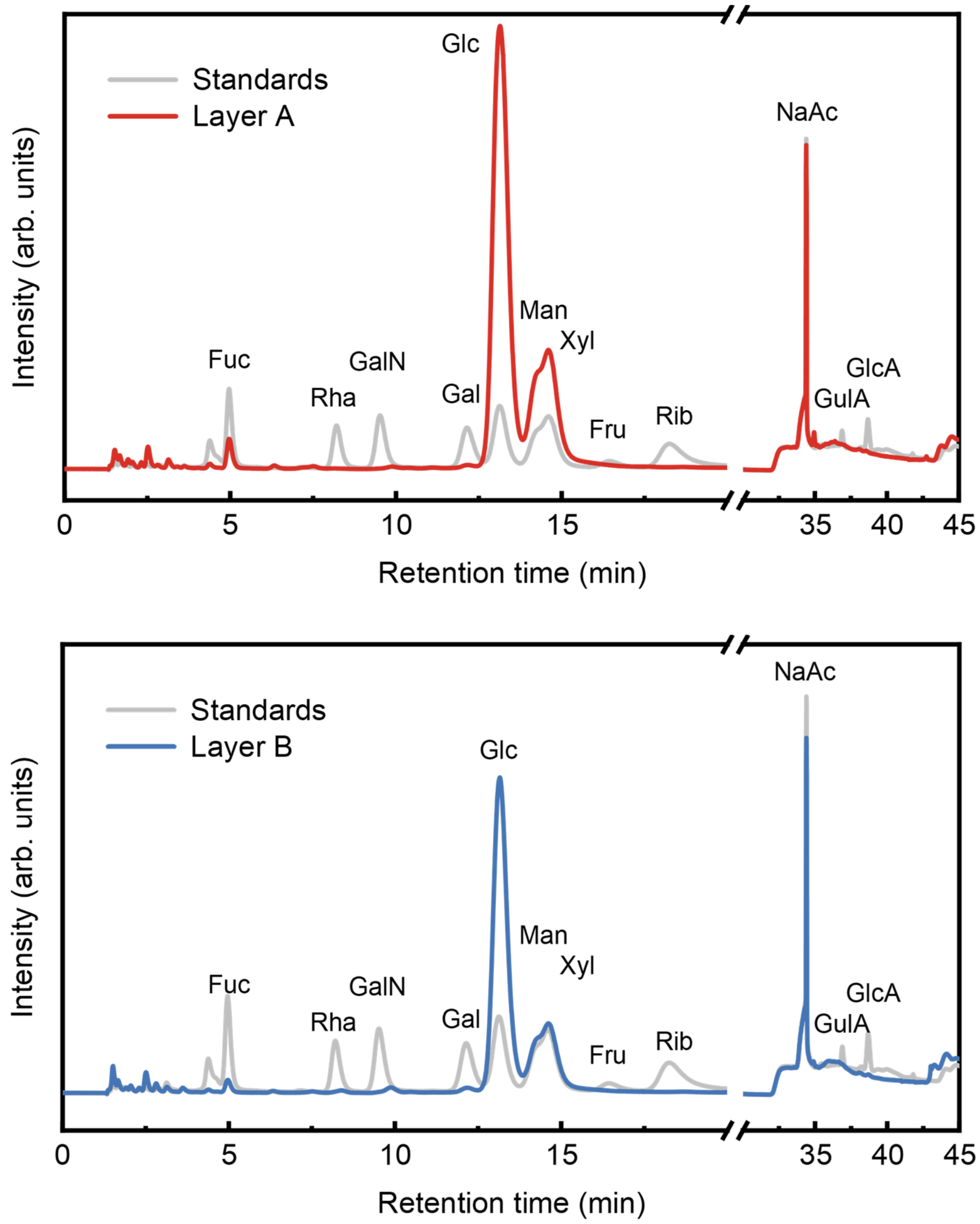

Supplementary Fig. 42 Ion Chromatogram of separated layer A and B from defatted and deproteinized mycelium membrane. N-acetyl glucosamine was not detected due to hydrolysis. Therefore, enzymolysis method was then employed to confirm chitin content in Janus structure.

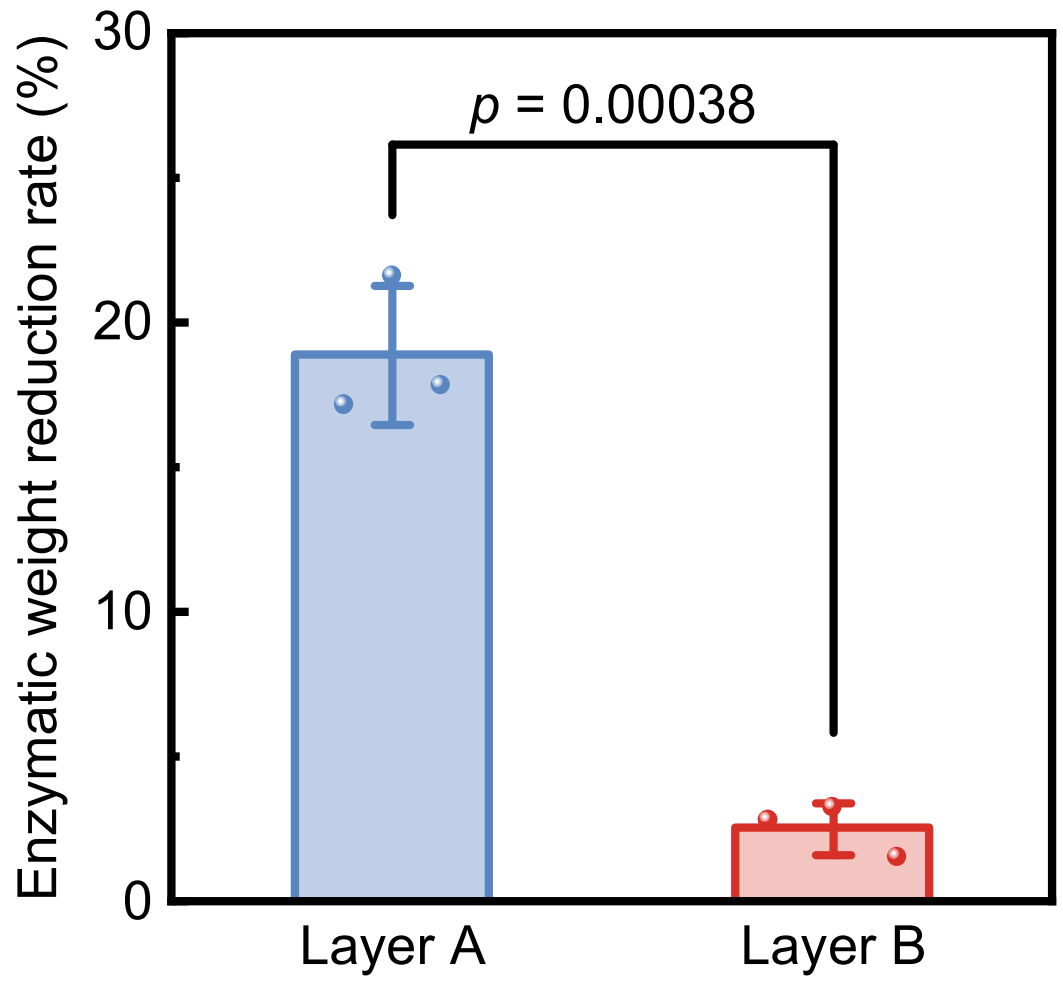

Supplementary Fig. 43 Weight reduction rates of separated layer A and B after dextranase treatment, n = 3. Error bars represented mean ± SD. Residual biomass was corresponding to chitin. It was noticed that the chitin content in native mycelium membrane was much higher than other polysaccharides, especially with glucan contents both in layer A and B much less than reported[15]. We hypothesized that during membrane mature and osmotic treatment, glucan in hyphae cell wall was mostly degraded, whereas chitin synthesis was upregulated.

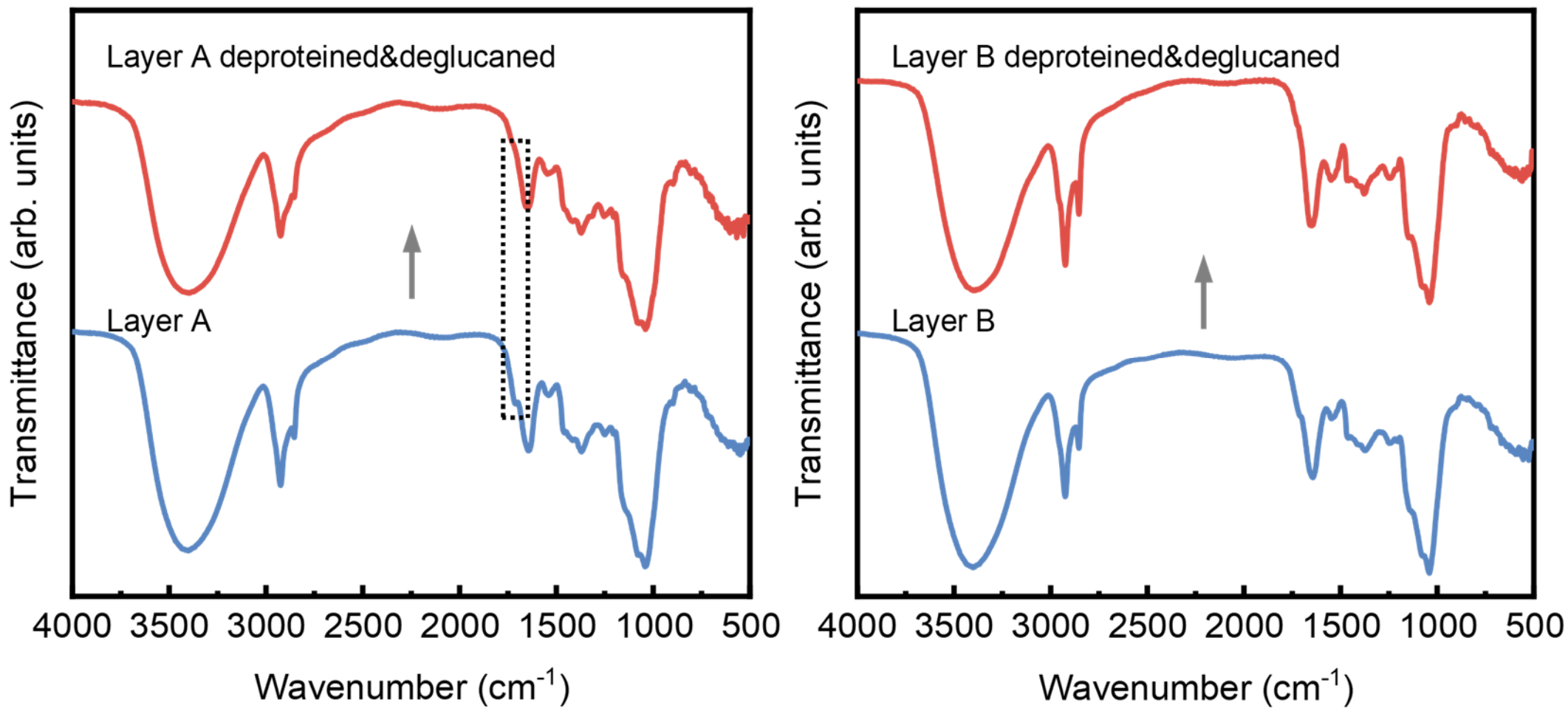

Supplementary Fig. 44 FTIR spectra of separated layer A and B after dextranase treatment, indicating glucan occupied much less than chitin in Trametes versicolor we used.

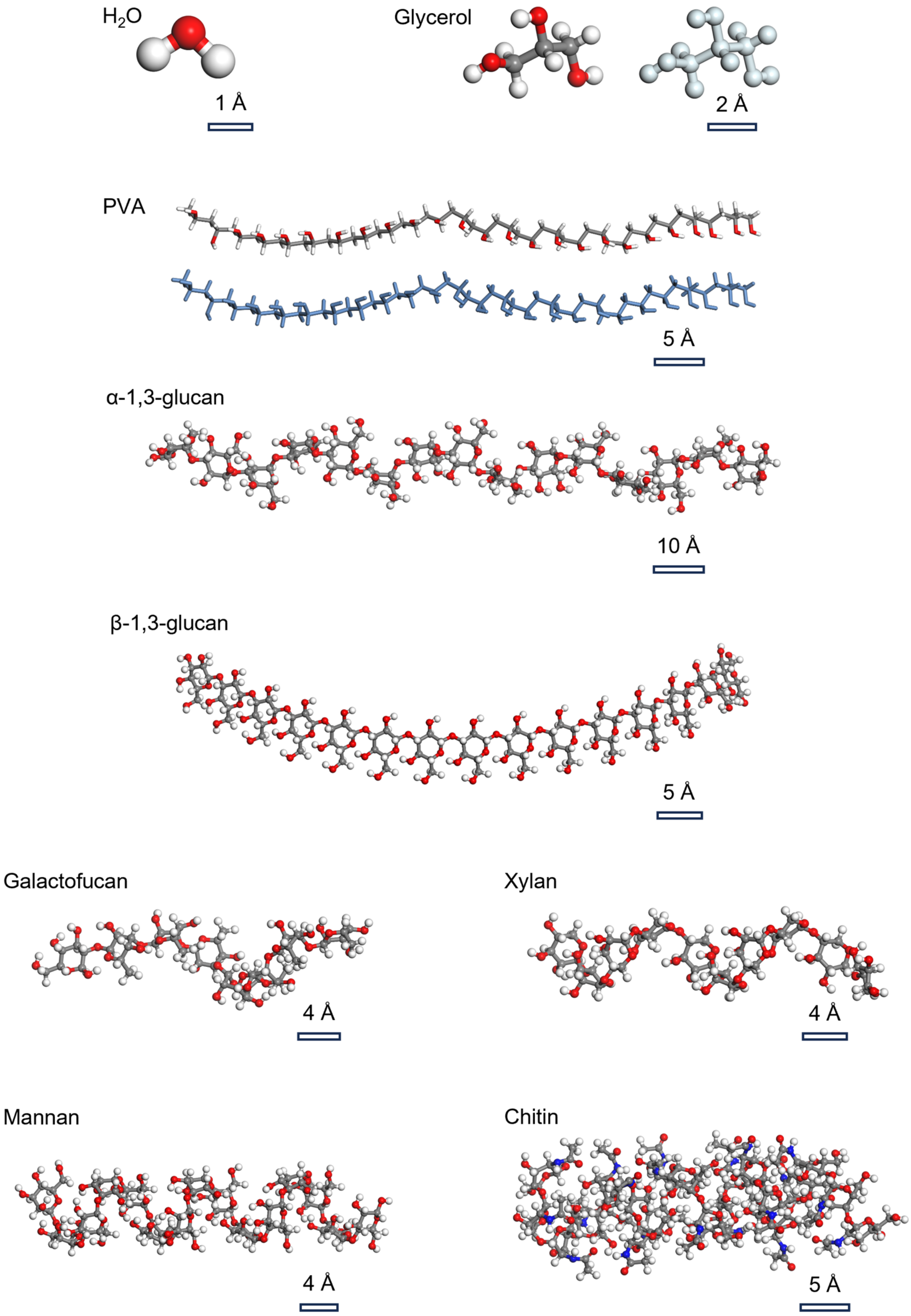

Supplementary Fig. 45 Geometrically optimized molecules as major components of Janus TV@P-G.

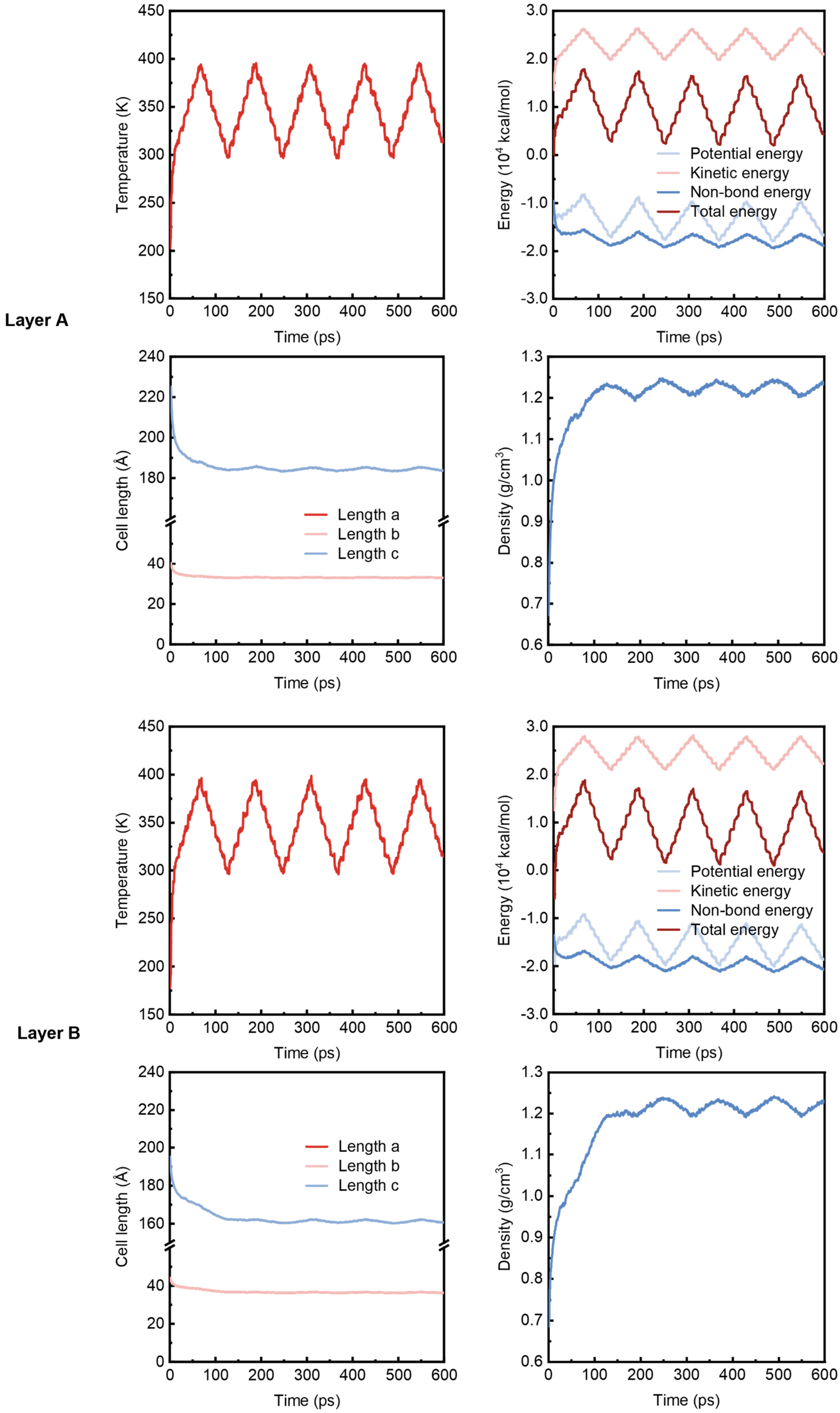

Supplementary Fig. 46 Temperature, energies, cell length and density equilibrium in MD simulation 6-cycled annealing process of dry layer A and B. Temperature was set from 298 K to 395 K and time step was 1 fs (length a = length b).

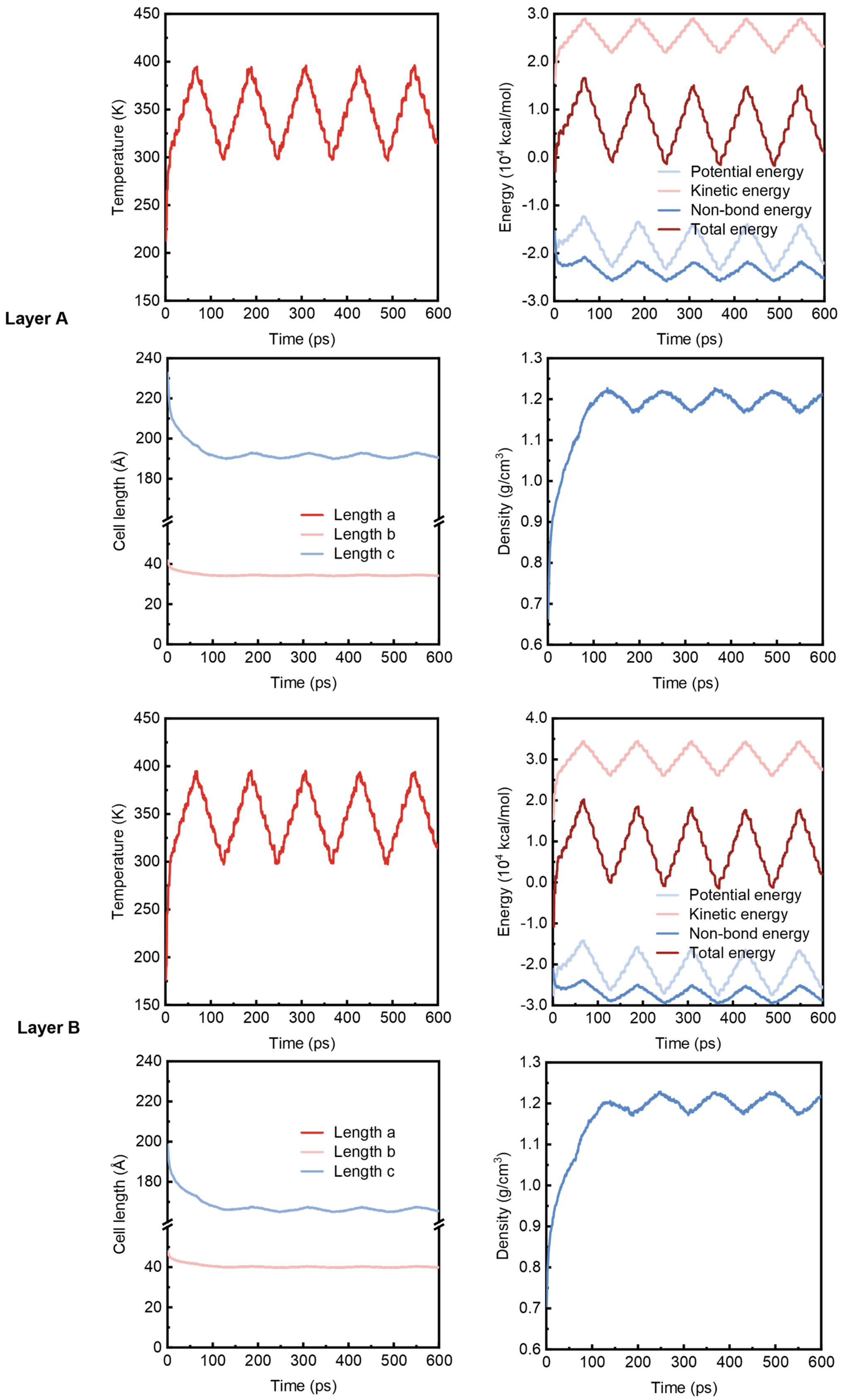

Supplementary Fig. 47 Temperature, energies, cell length and density equilibrium in MD simulation 6-cycled annealing process of wet layer A and B. Temperature was set from 298 K to 395 K and time step was 1 fs, (length a = length b).

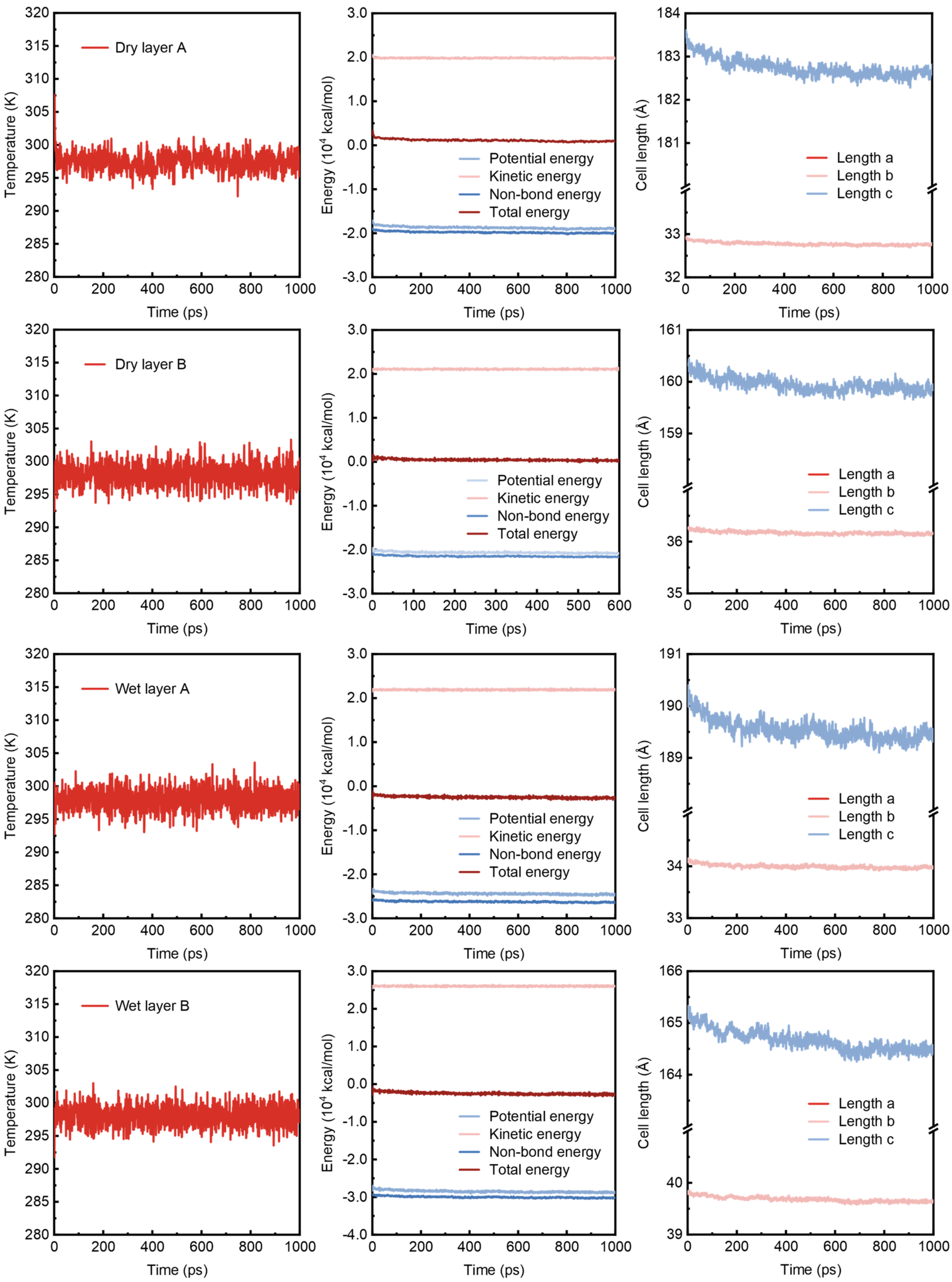

Supplementary Fig. 48 Temperature, energies and cell length equilibrium in MD simulation 1000 ps dynamics process of layer A and B in dry and wet state respectively. Temperature was set at 298 K and time step was 1 fs, (length a = length b).

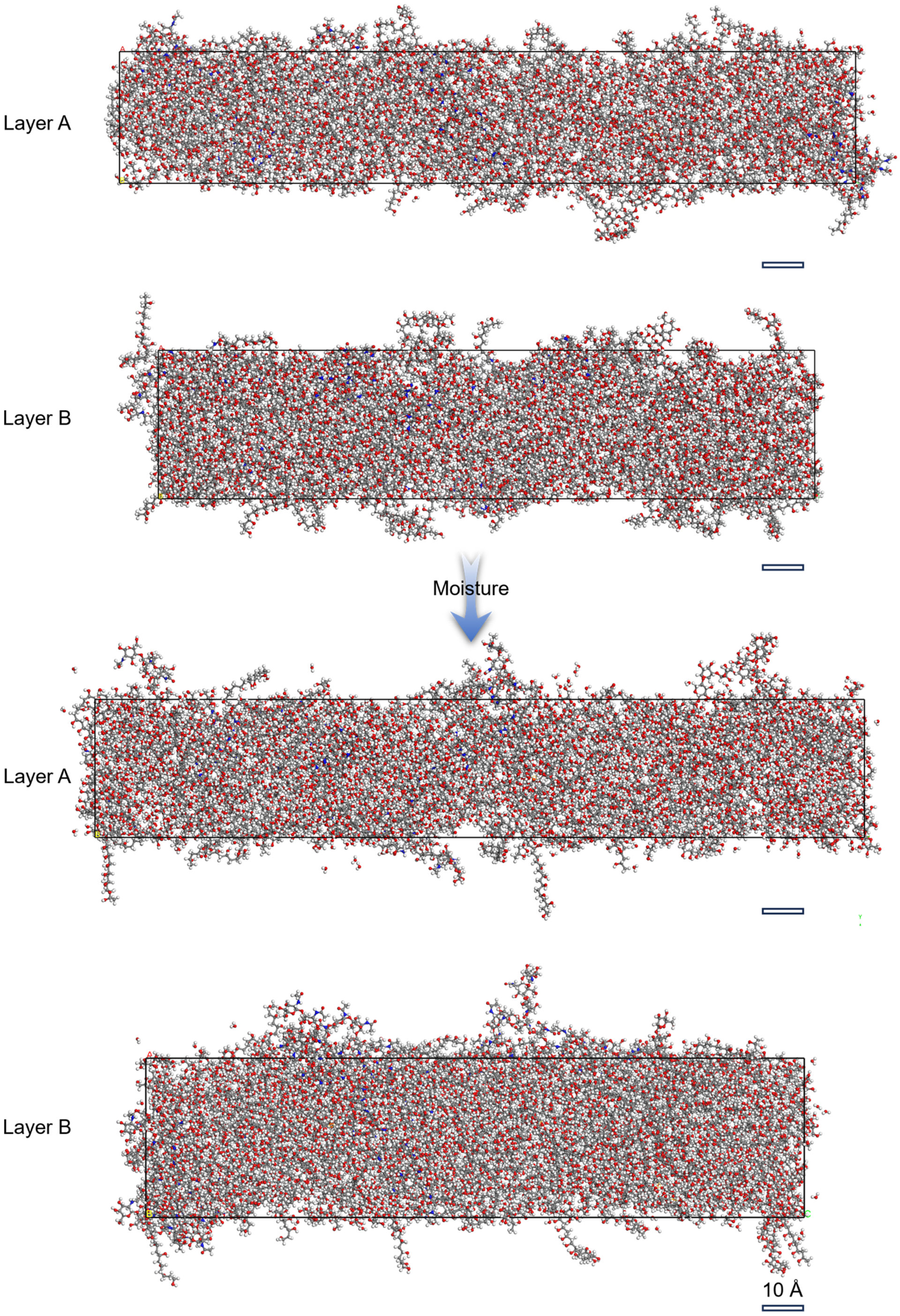

Supplementary Fig. 49 Amorphous cell of dry and wet layer A and B, respectively, after geometrically optimized, annealing and molecular dynamics process.

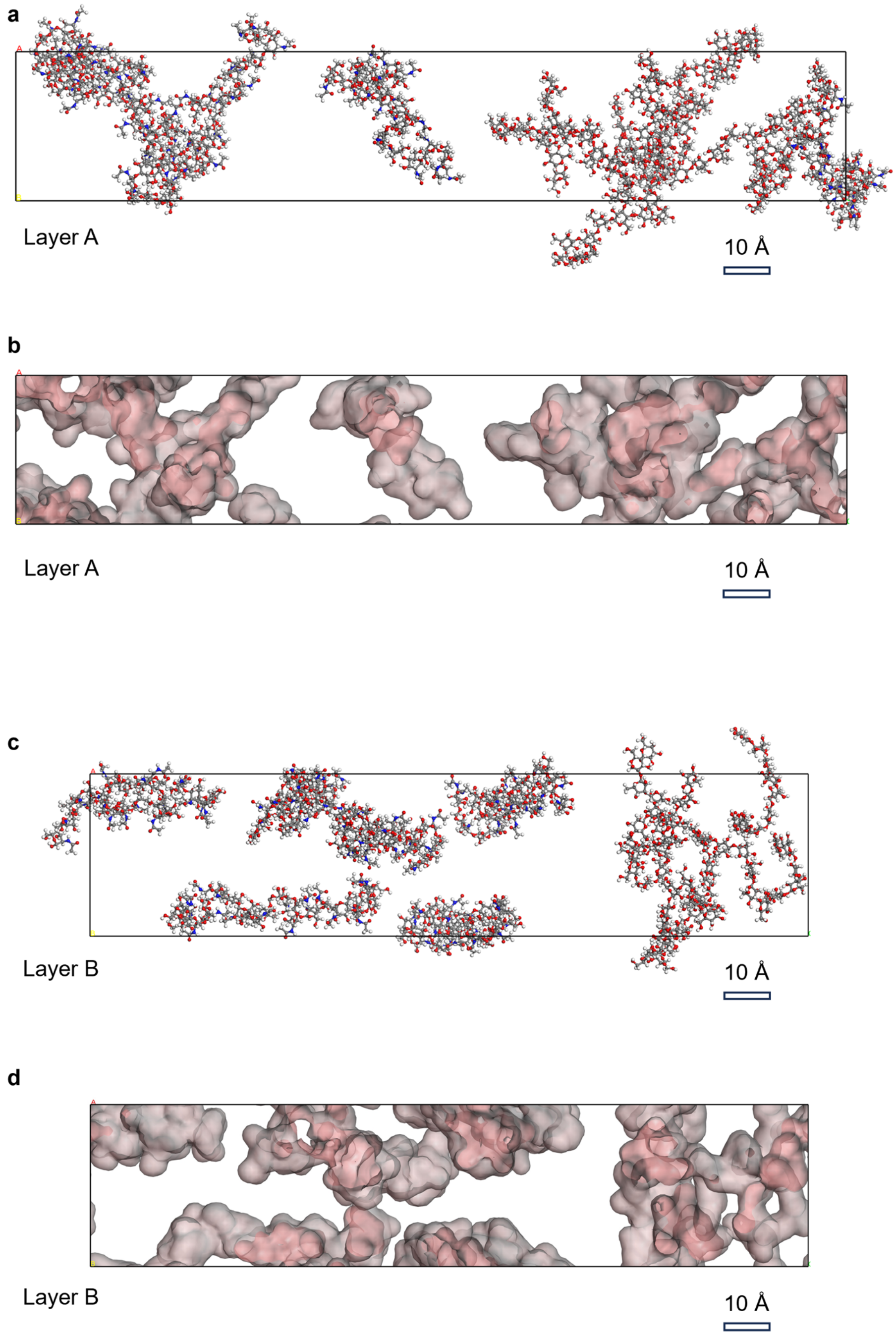

Supplementary Fig. 50 Simulated amorphous cells of dry layers A and B. **a**, **c**, Biomacromolecular structures. **b**, **d**, Connolly surfaces of the biomacromolecules, generated with a Connolly radius of 1.00 Å.

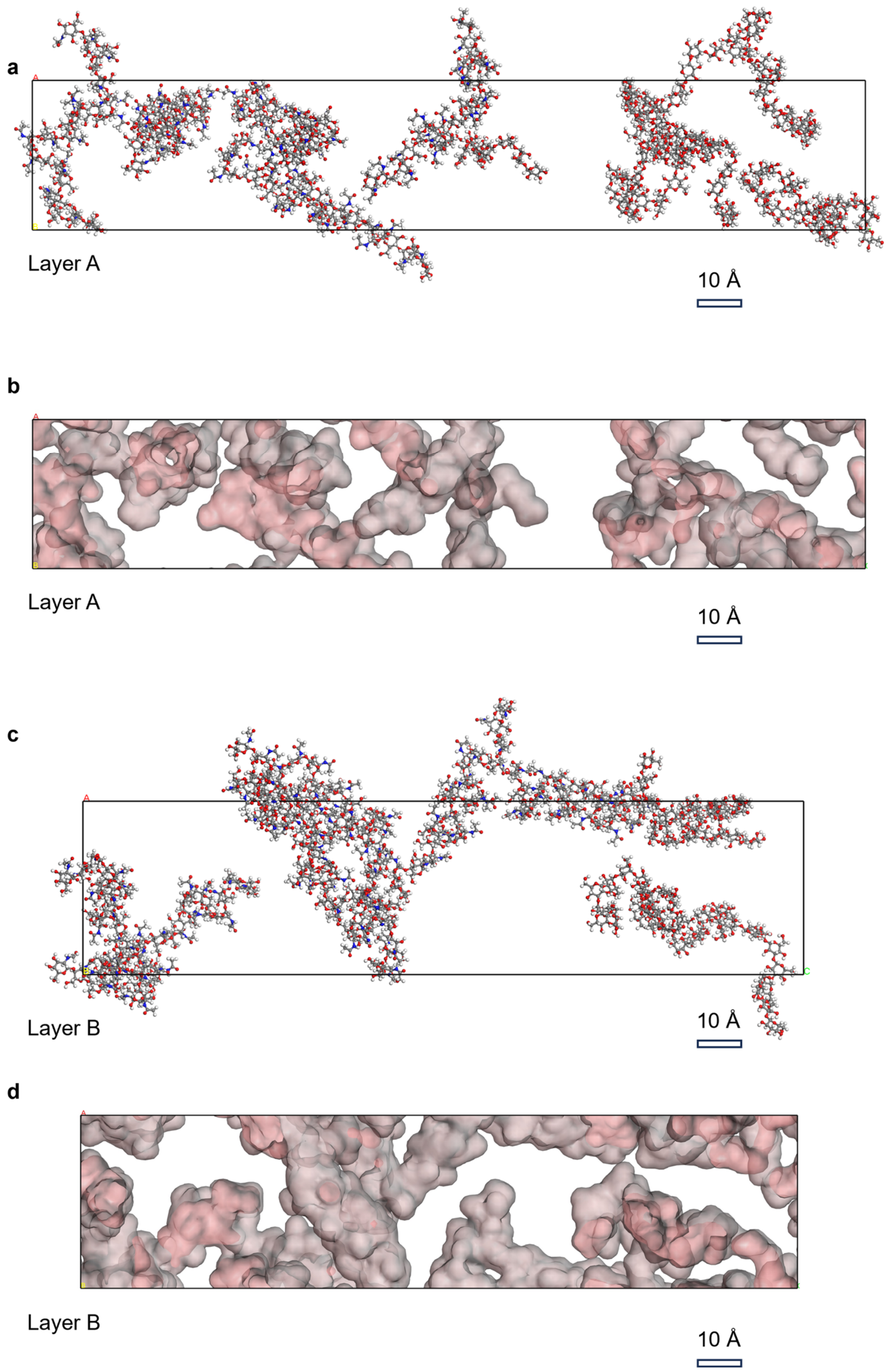

Supplementary Fig. 51 Simulated amorphous cells of hydrated layers A and B. **a**, **c**, Biomacromolecular structures. **b**, **d**, Connolly surfaces of the biomacromolecules, generated with a Connolly radius of 1.00 Å.

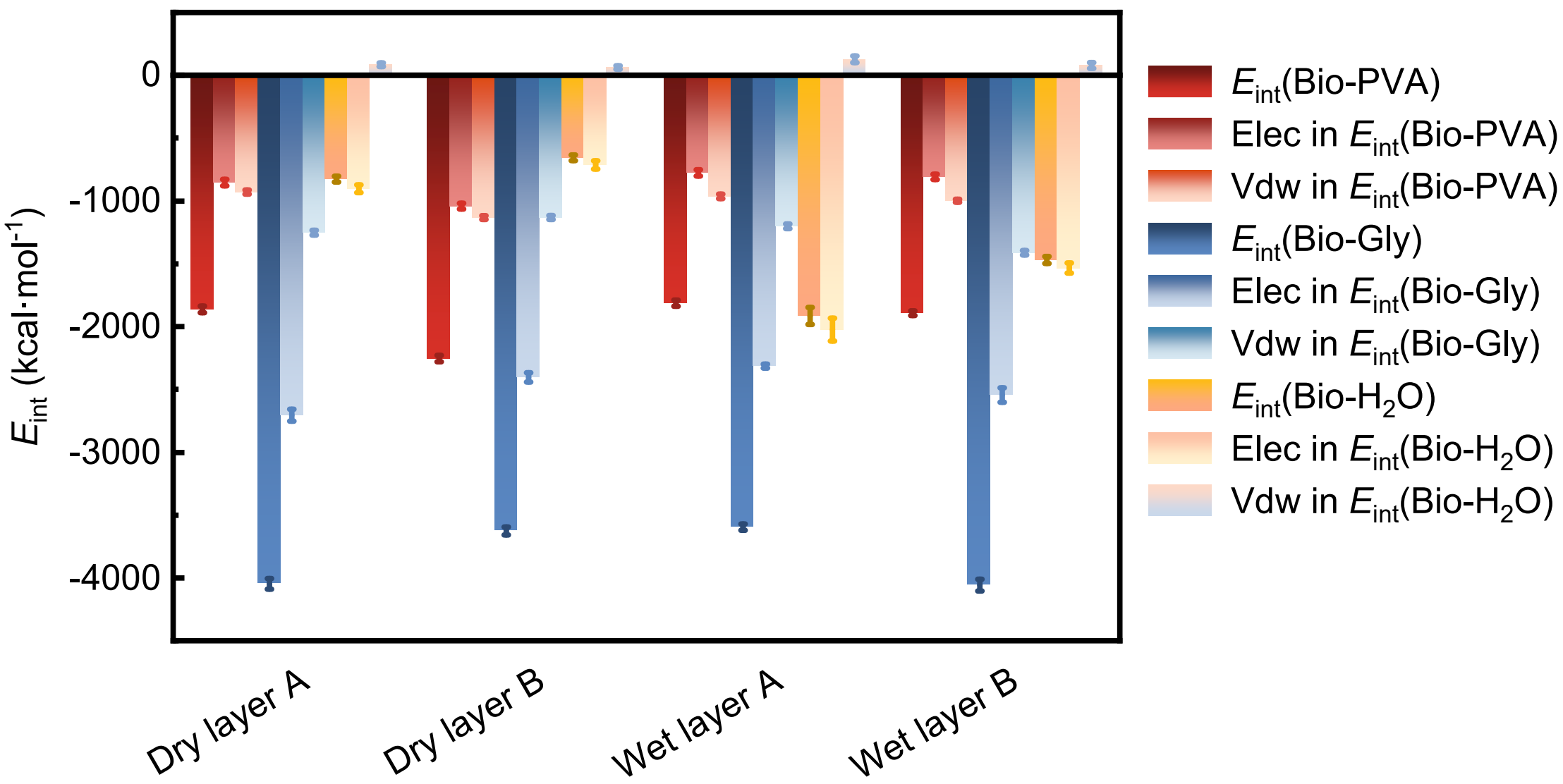

Supplementary Fig. 52 Interaction energy including electrostatic interaction and van der Waals force between biomass and PVA or glycerol in layer A and B from dry to wet environment.

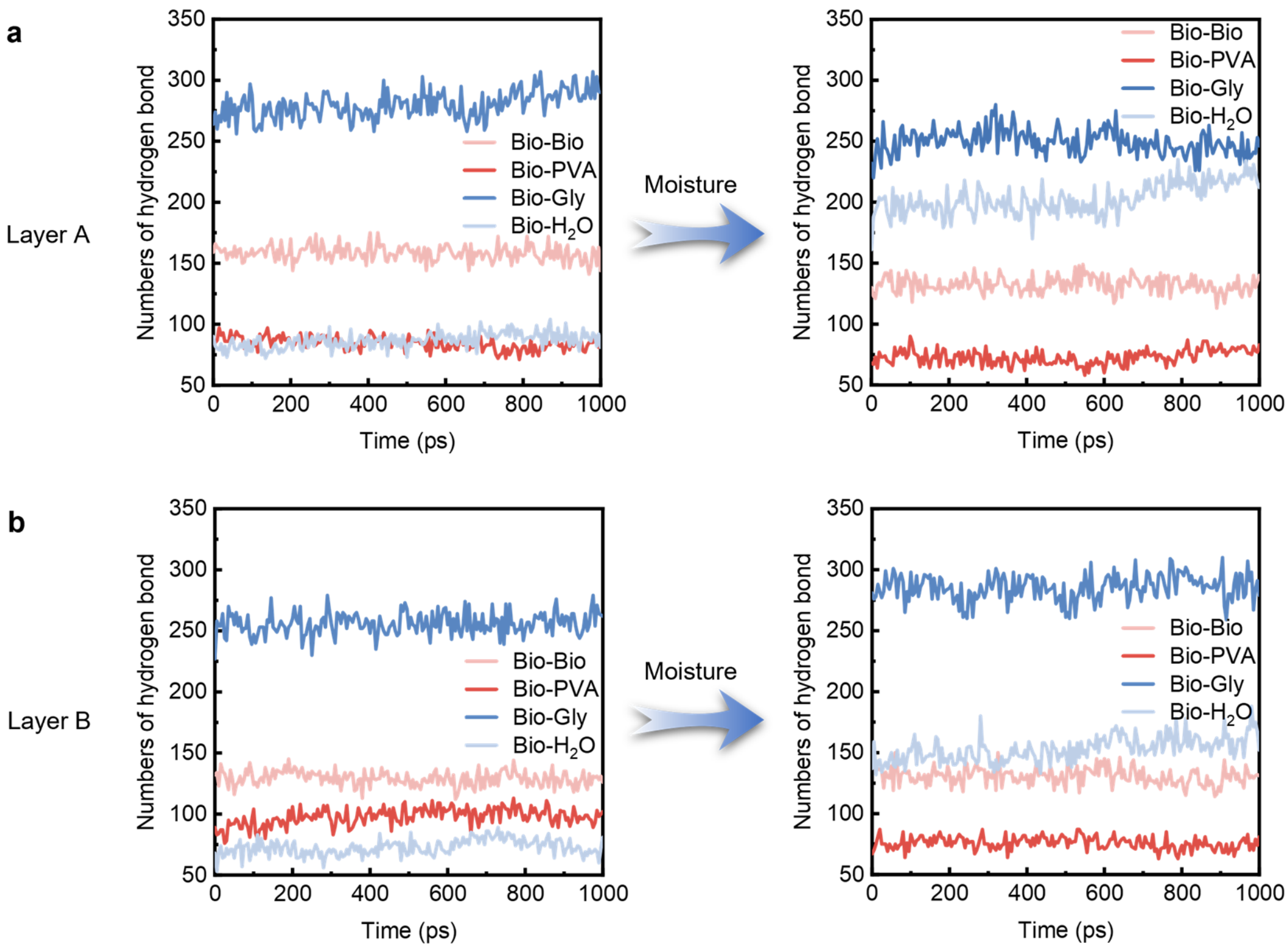

Supplementary Fig. 53 Number of hydrogen bond between biomass and PVA or glycerol in layer A and B from dry to wet environment.

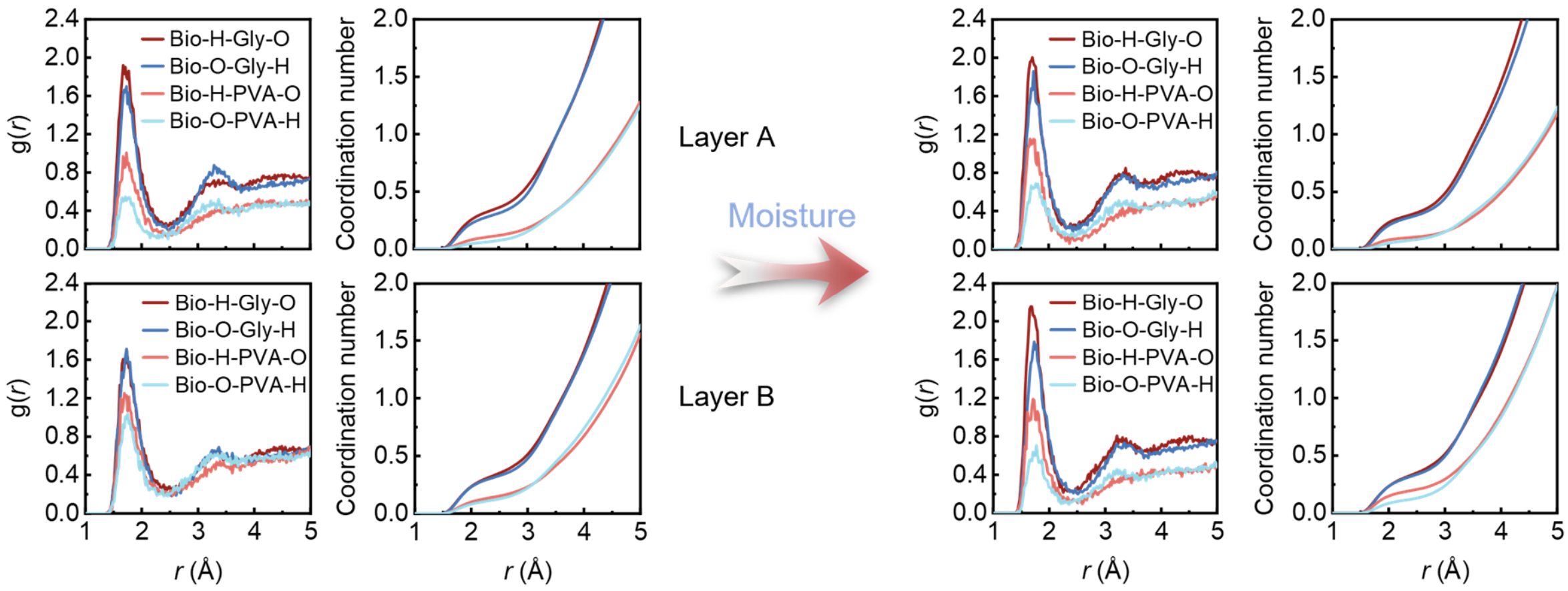

Supplementary Fig. 54 Influence of moisture on MD simulation radial distribution function and coordination number between biomacromolecules and PVA or glycerol in layer A and B.

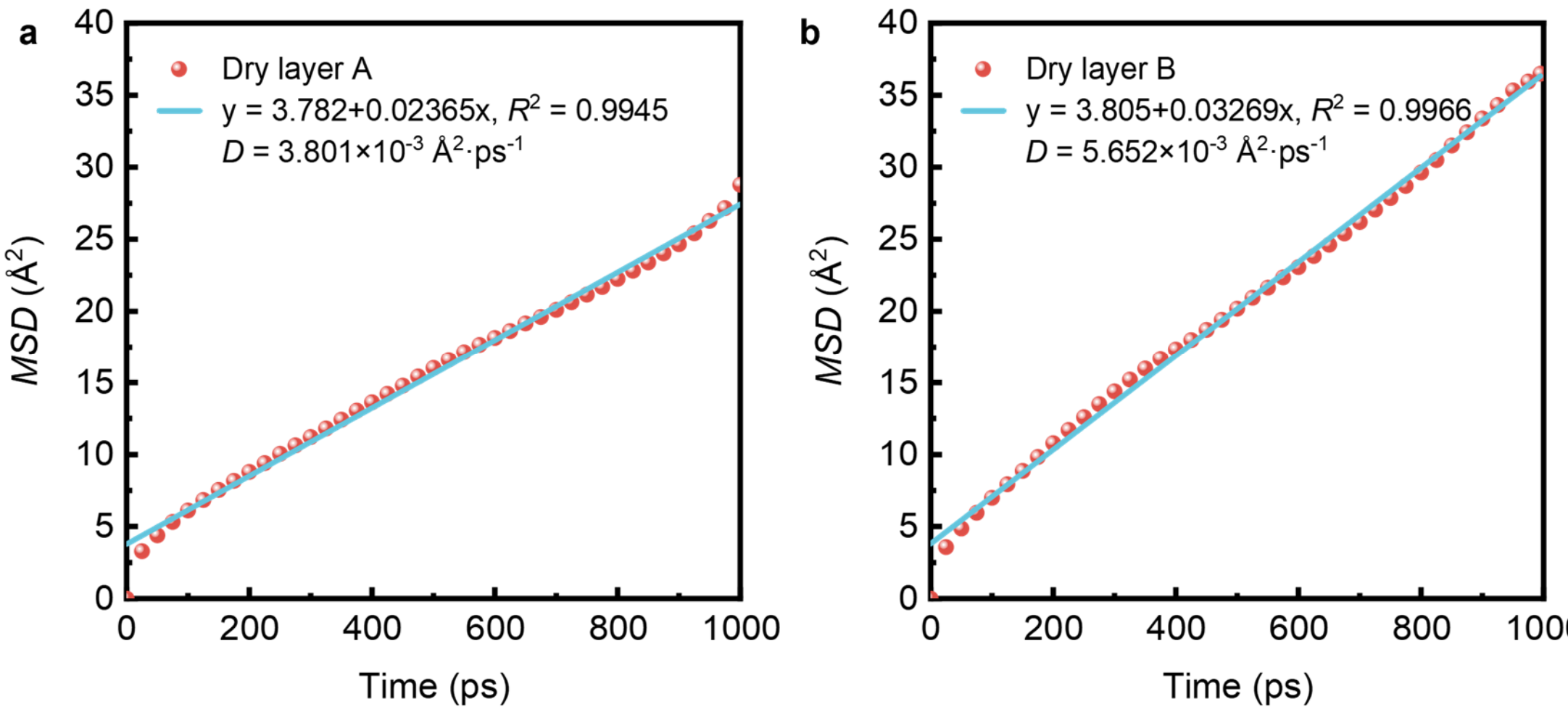

Supplementary Fig. 55 MD simulation mean square displacement (*MSD*) of water molecule in dry layer A and B, with diffusion coefficient (*D*) calculated according to slope.

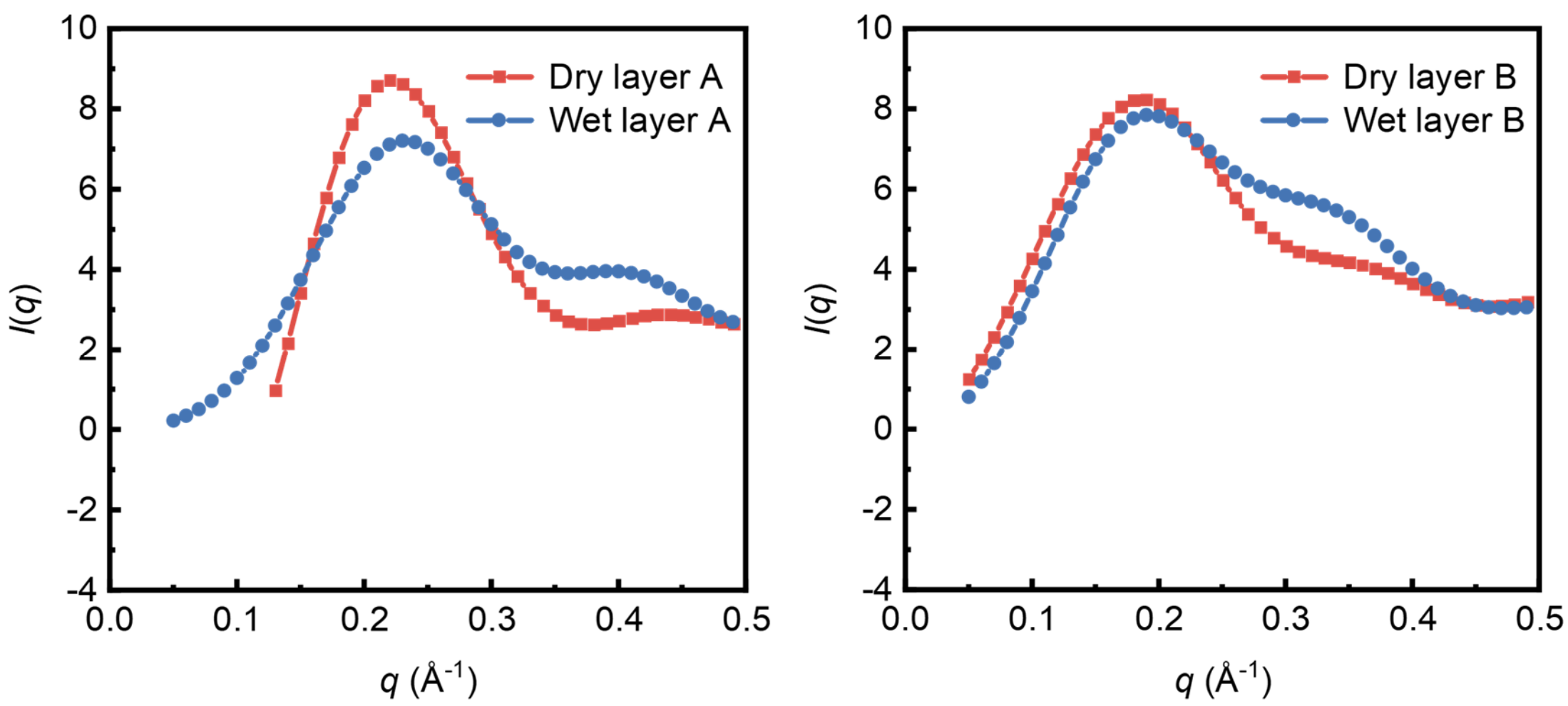

Supplementary Fig. 56 MD simulation X-ray scattering of dry and wet layer A and B

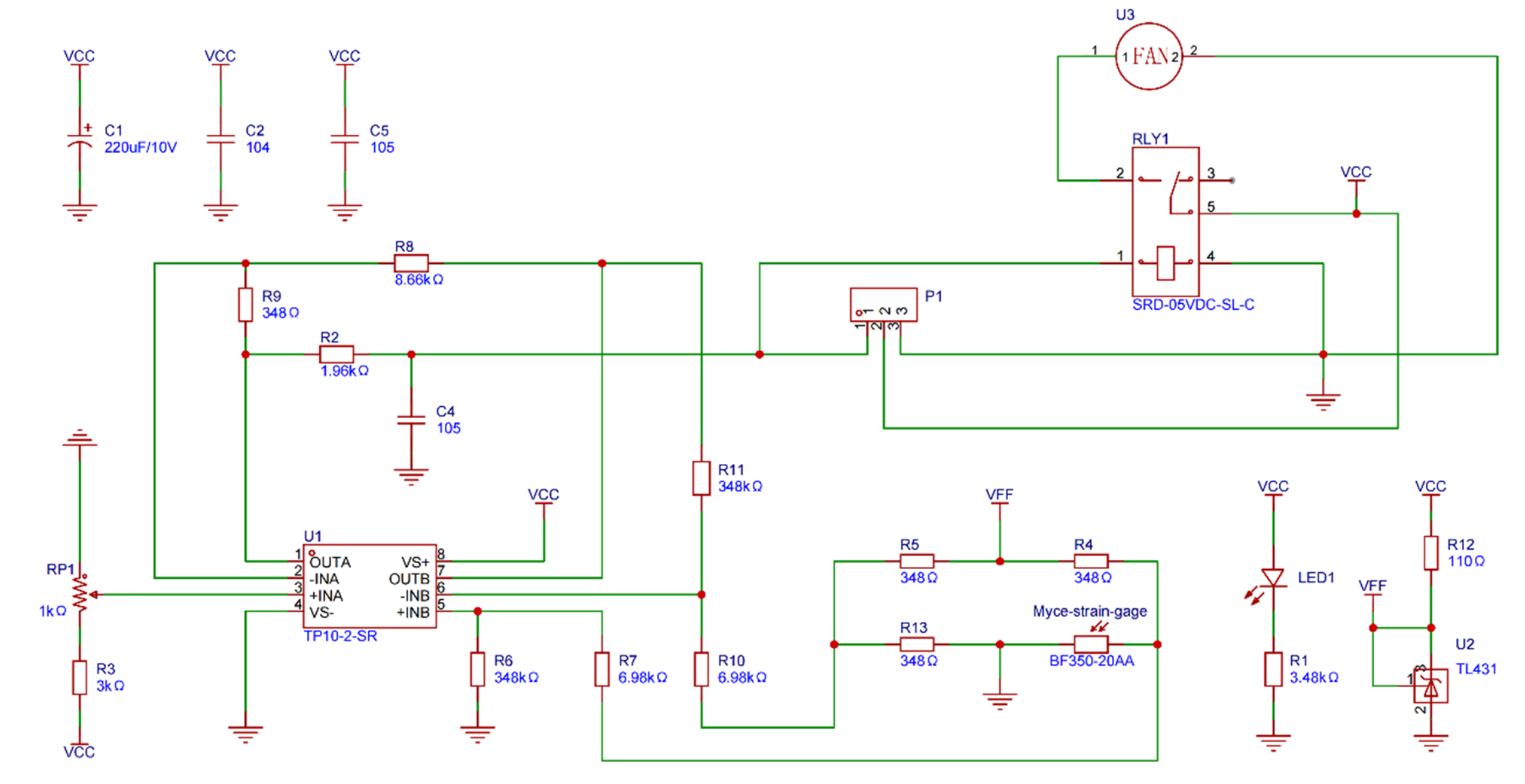

Supplementary Fig. 57 Circuit diagram to control the fan with Myce strain gage.

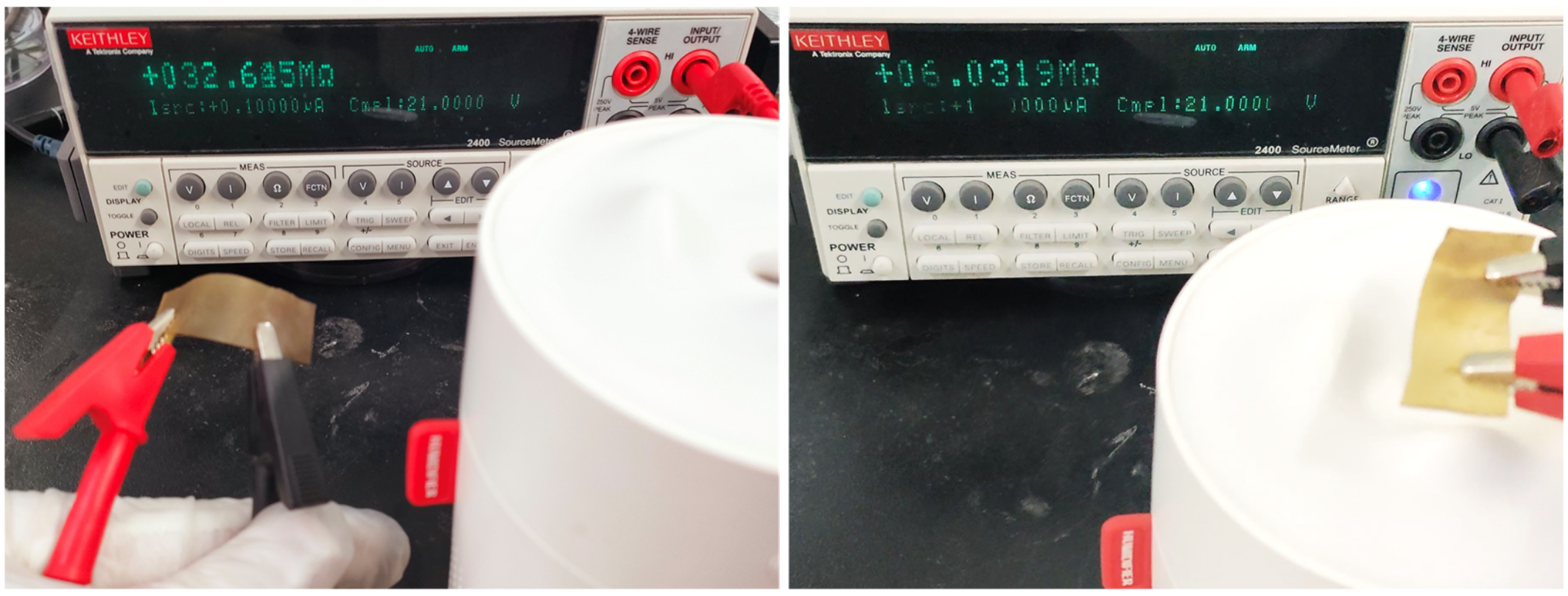

Supplementary Fig. 58 Optical images of resistance response during humidification of TV@P-G collected by R module of Keithley 2400 source meter unit (SMU).

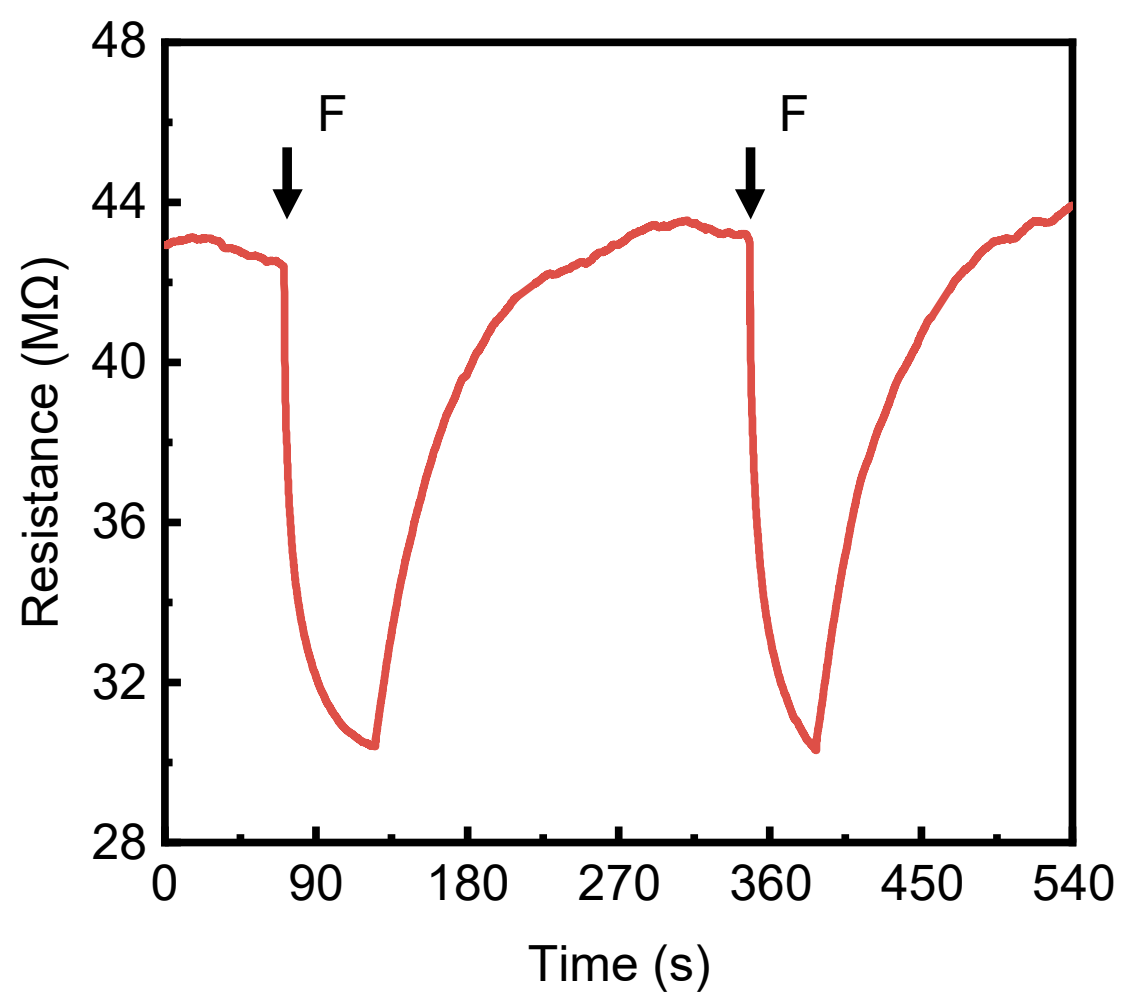

Supplementary Fig. 59 Control group of resistance response to force applied on TV@P-G collected by SMU.

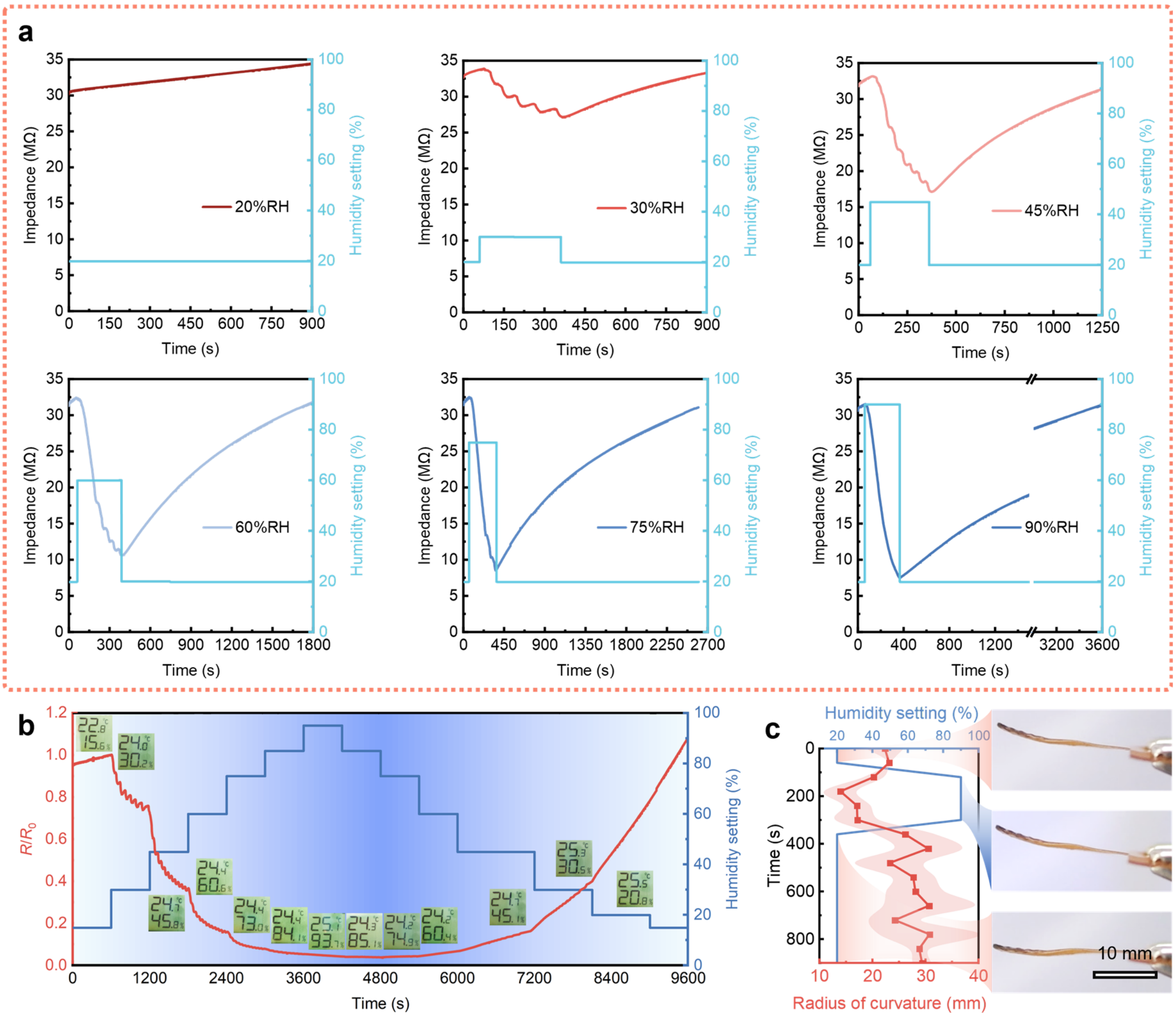

Supplementary Fig. 60 Mycelium macerate transduced humidity signal into electrical signal through resistance variation. **a**, Resistance response during humidification and recovery of TV@P-G in the constant humidity chamber. Humidity was set as 20, 30, 45, 60, 75 and 90% RH for modulating samples' response. **b**, Resistance response during continuous humidification and recovery of TV@P-G in the constant humidity chamber, with optical images of temperature and relative humidity recorded. **c**, Deformation applied on visualization of humidity changes from 20% RH to 90% RH, with resistance data collecting simultaneously. Shown is a representative histogram from three independent experiments (n = 3) with similar outcomes.

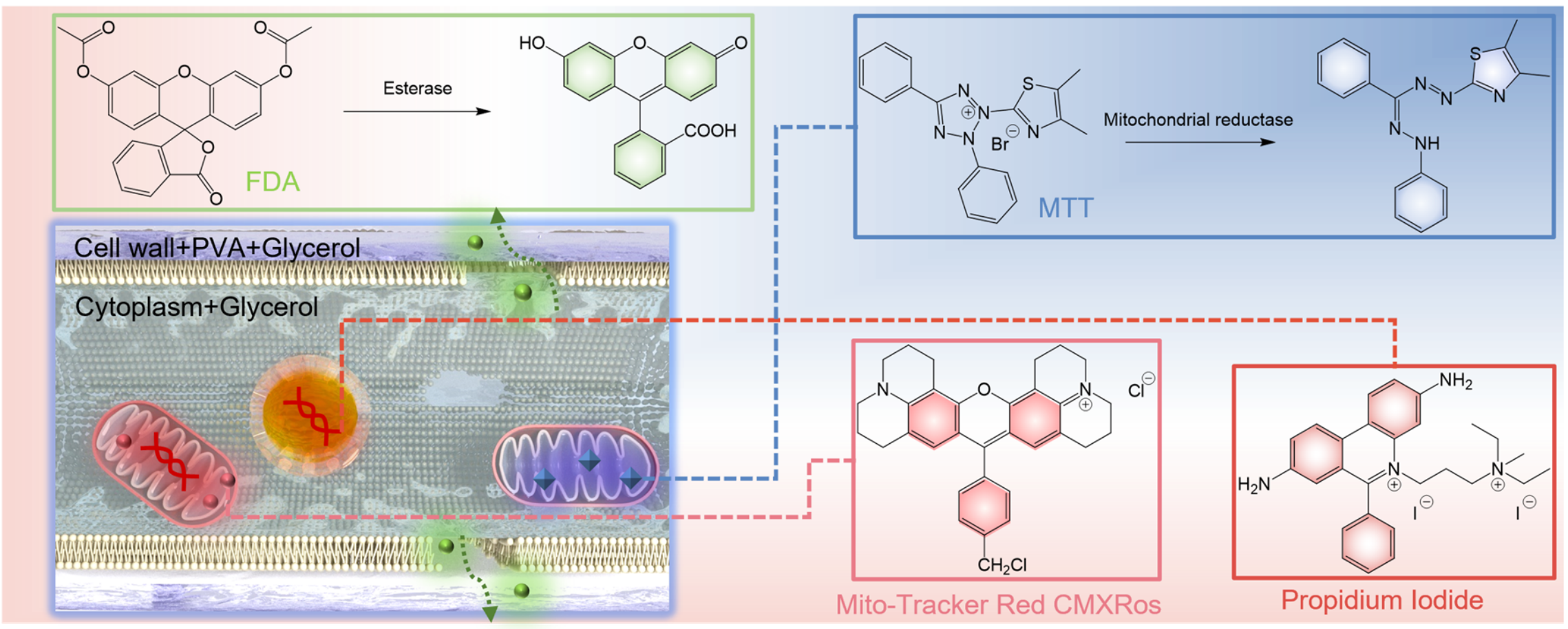

Supplementary Fig. 61 Schematic diagram of fluorescent stained TV@P-G section under desiccation state, indicates the localization of four kind of dyes, including FDA, PI, MTT and Mito-Tracker Red CMXRos in dormant Trametes versicolor hypha cell with damaged cell membrane and active mitochondria.

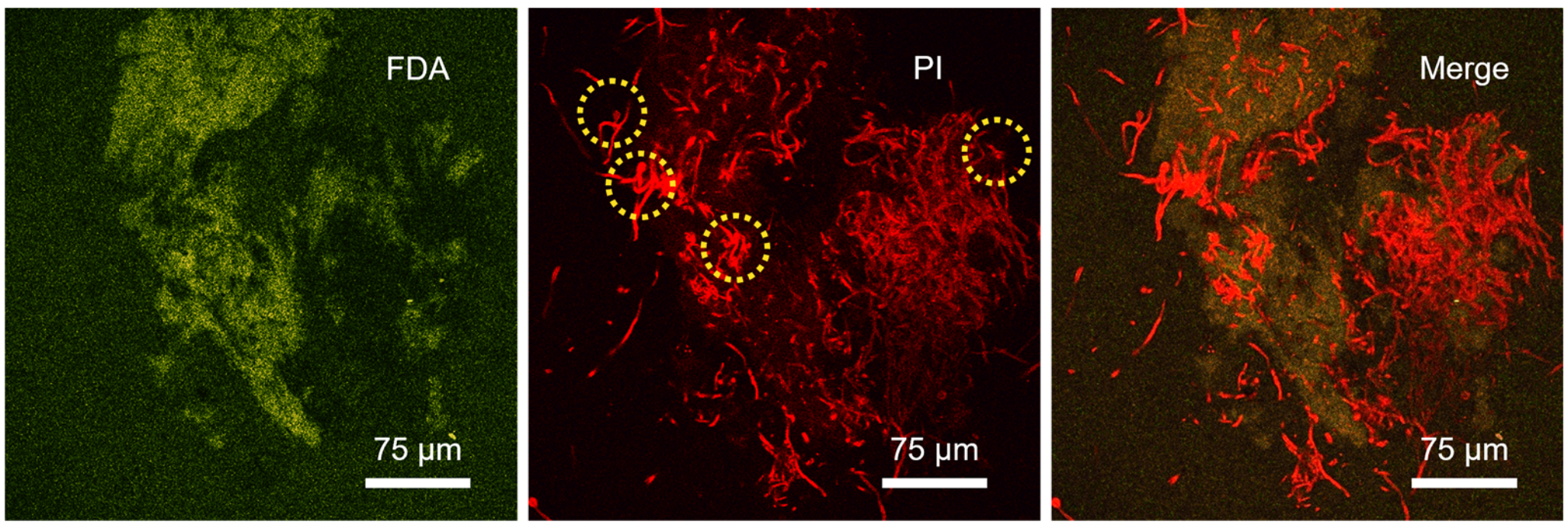

Supplementary Fig. 62 Confocal images of FDA-PI stained TV@P-G section. Circles represent spores. Extracellular fluorescein generation indicates enzyme activity of esterase and membrane damage. Colocation of PI and hyphae cells further supports that membrane damage occurred during desiccation. Data are representative of at least three independent biological replicates (n = 3) with similar results.

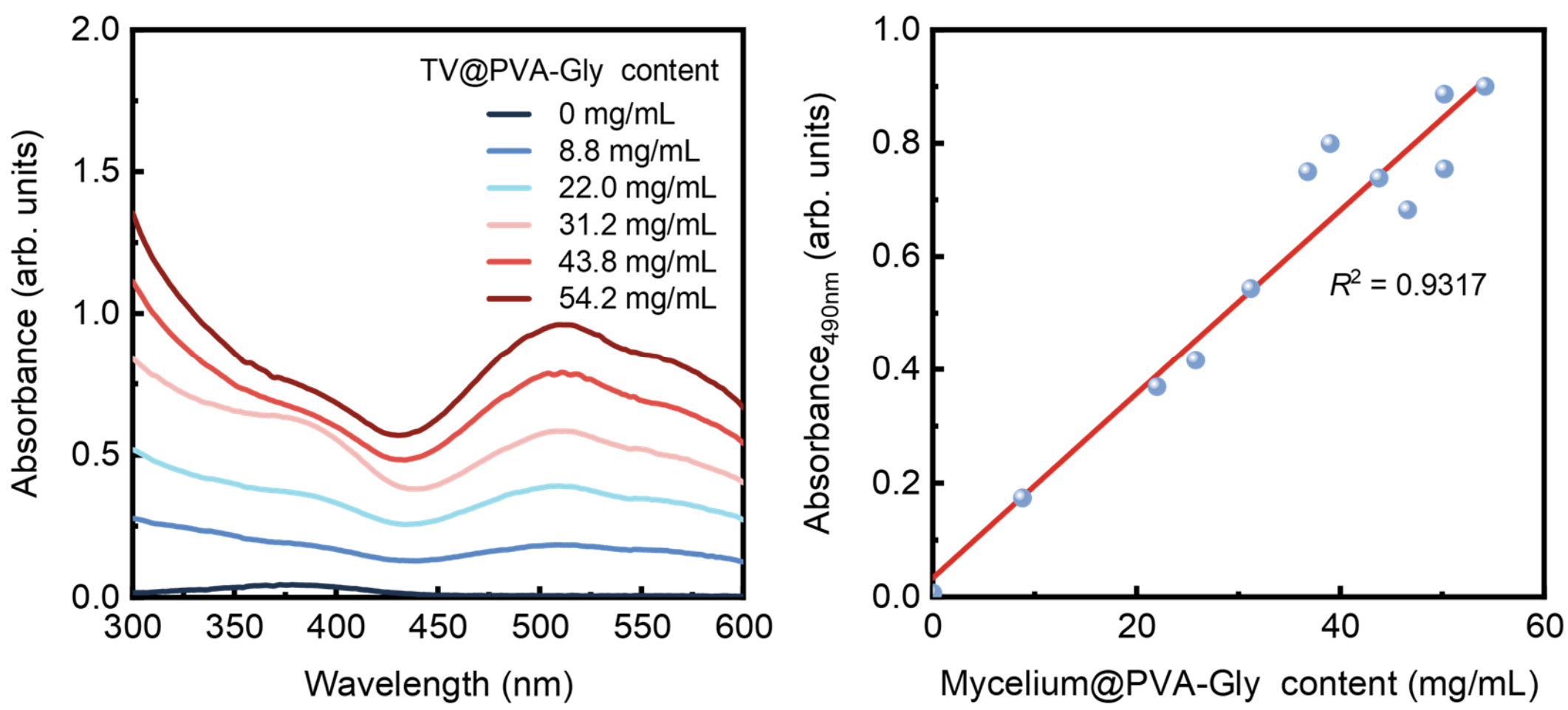

Supplementary Fig. 63 UV-vis spectra and standard curve of MTT formazan extracted with DMSO from MTT treated TV@P-G.

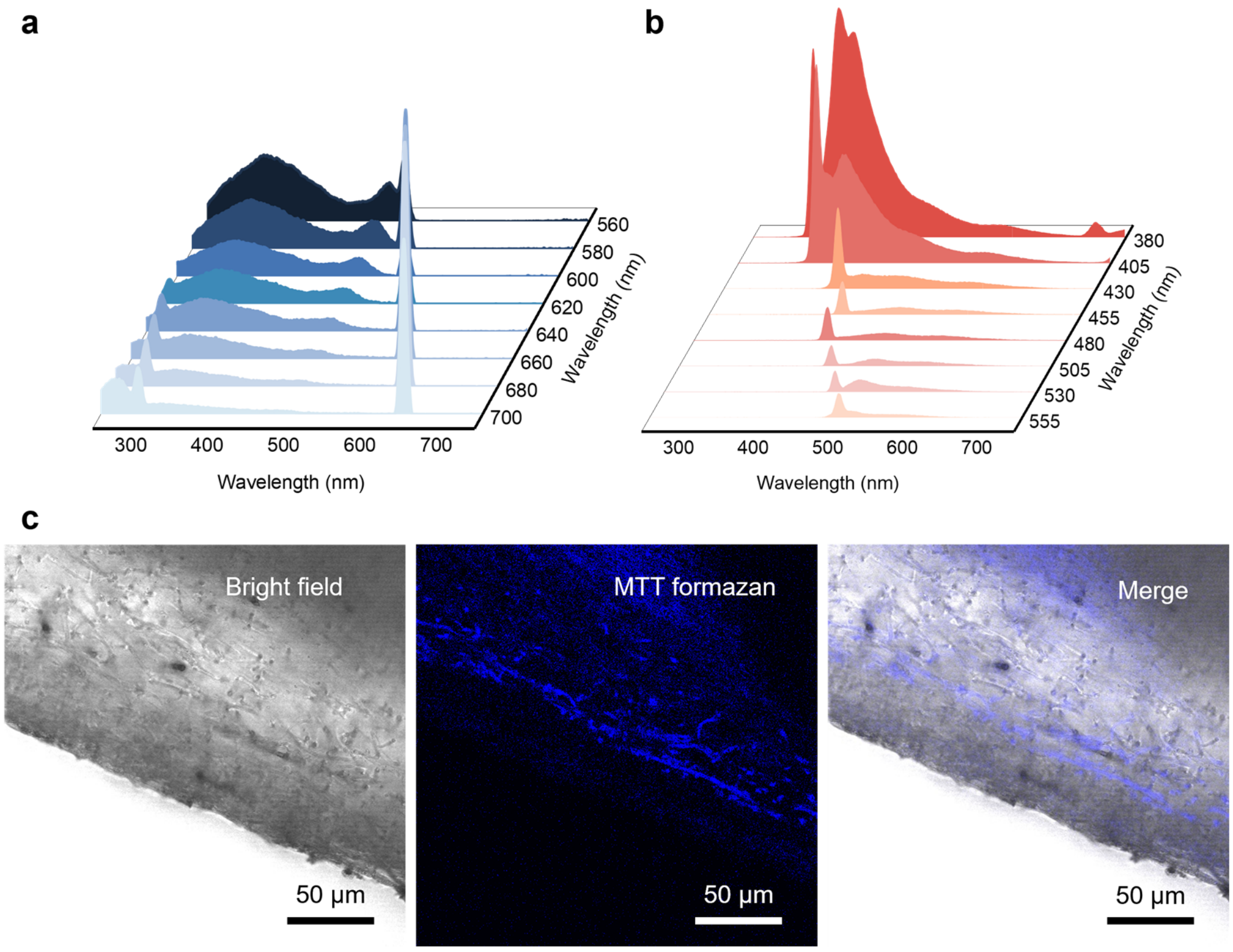

Supplementary Fig. 64 Characterization of MTT stained mycelium macerate. **a**, **b**, Fluorescence excitation (**a**) and emission (**b**) spectra of MTT formazan extracted with DMSO from MTT treated TV@P-G. **c**, Confocal images of MTT treated TV@P-G section. Data are representative of three independent biological replicates (n = 3) with similar results.

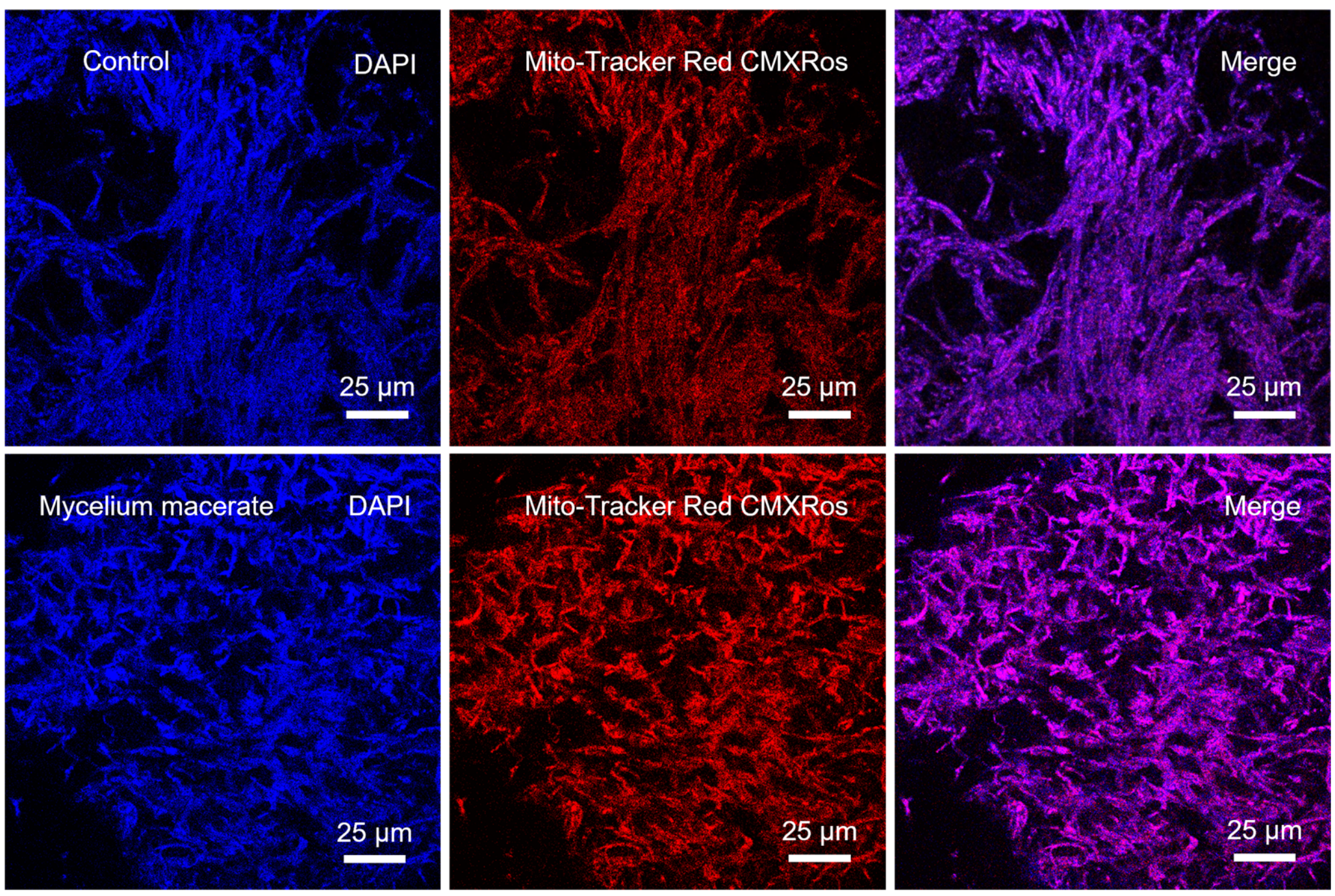

Supplementary Fig. 65 Confocal images and 3D reconstruction of DAPI and Mito-Tracker Red CMXRos stained living mycelium membrane control group and mycelium macerate section. The experiment was repeated independently three times (n = 3) with similar trends, and a representative experiment is shown.

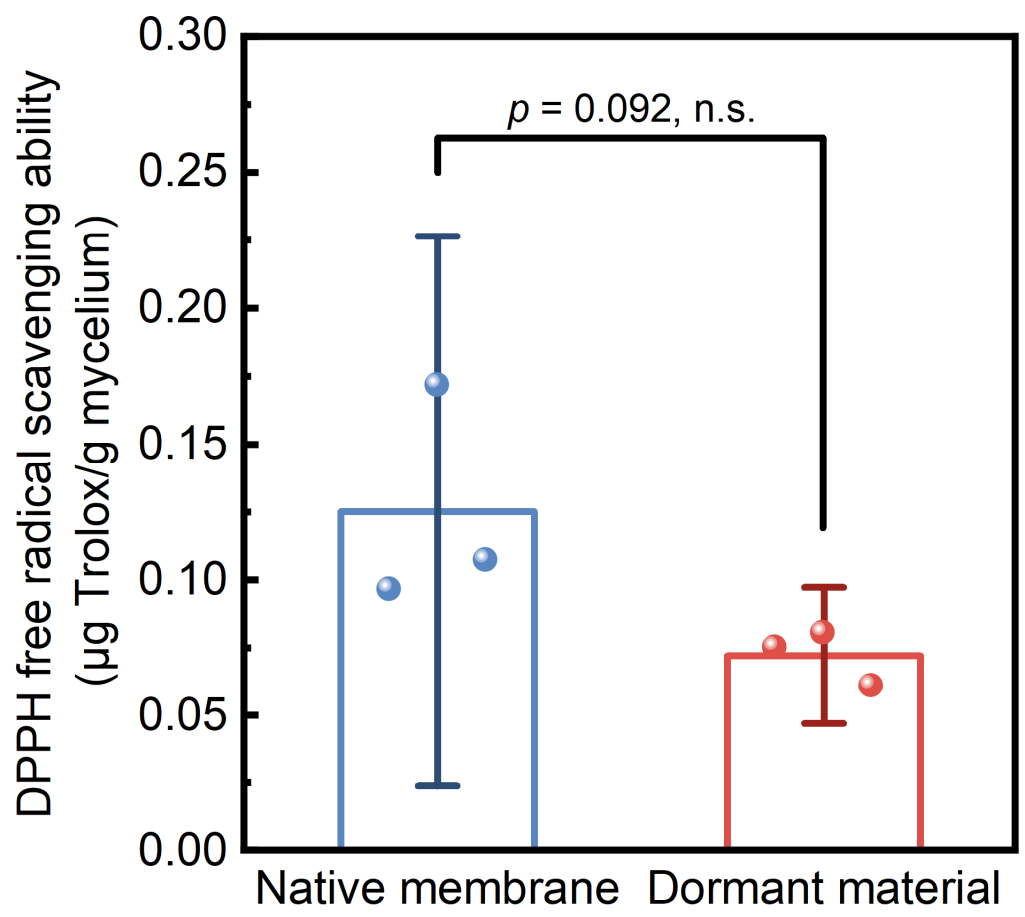

Supplementary Fig. 66 Determination of DPPH free radical clearance of native mycelium membrane and dormant macerate material, n = 3. Error bars represented mean ± SD.

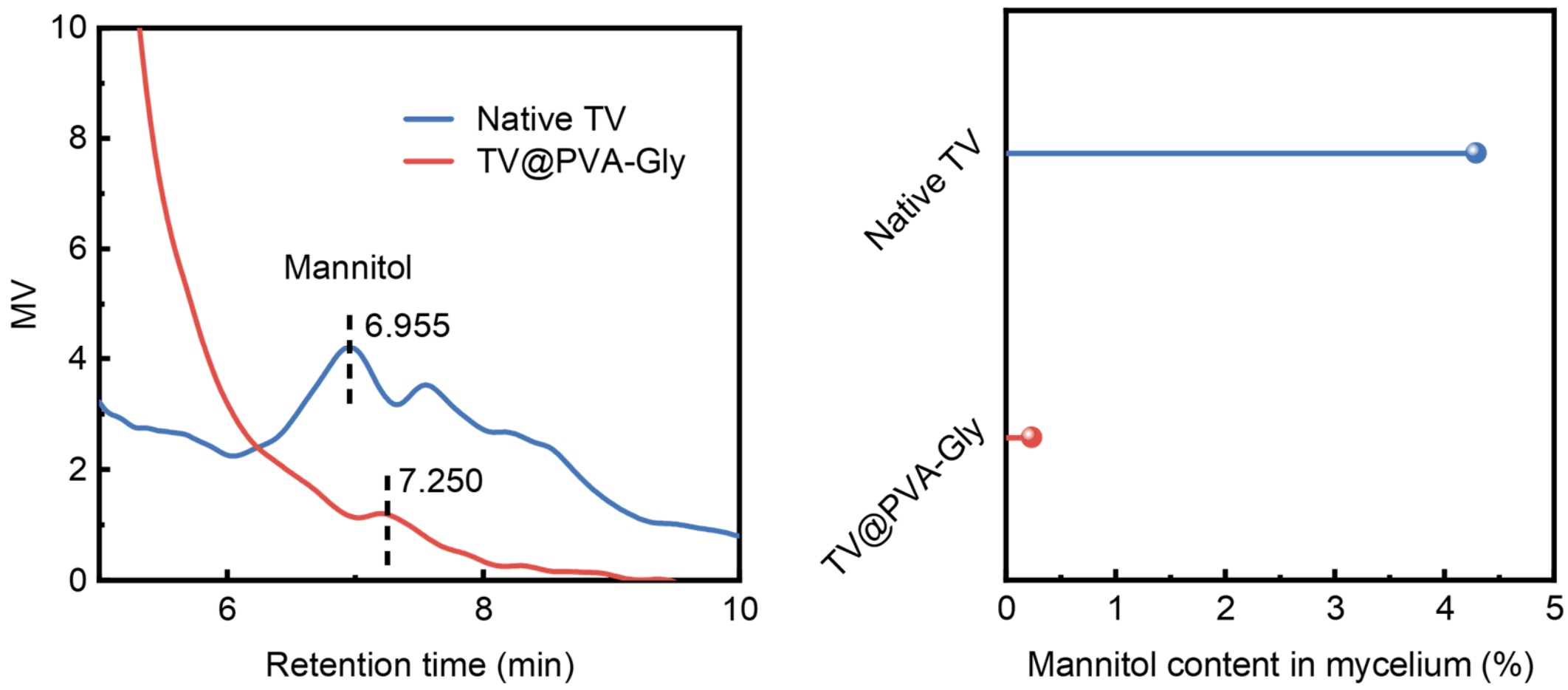

Supplementary Fig. 67 HPLC detection for mannitol content in native mycelium membrane and TV@P-G macerate.

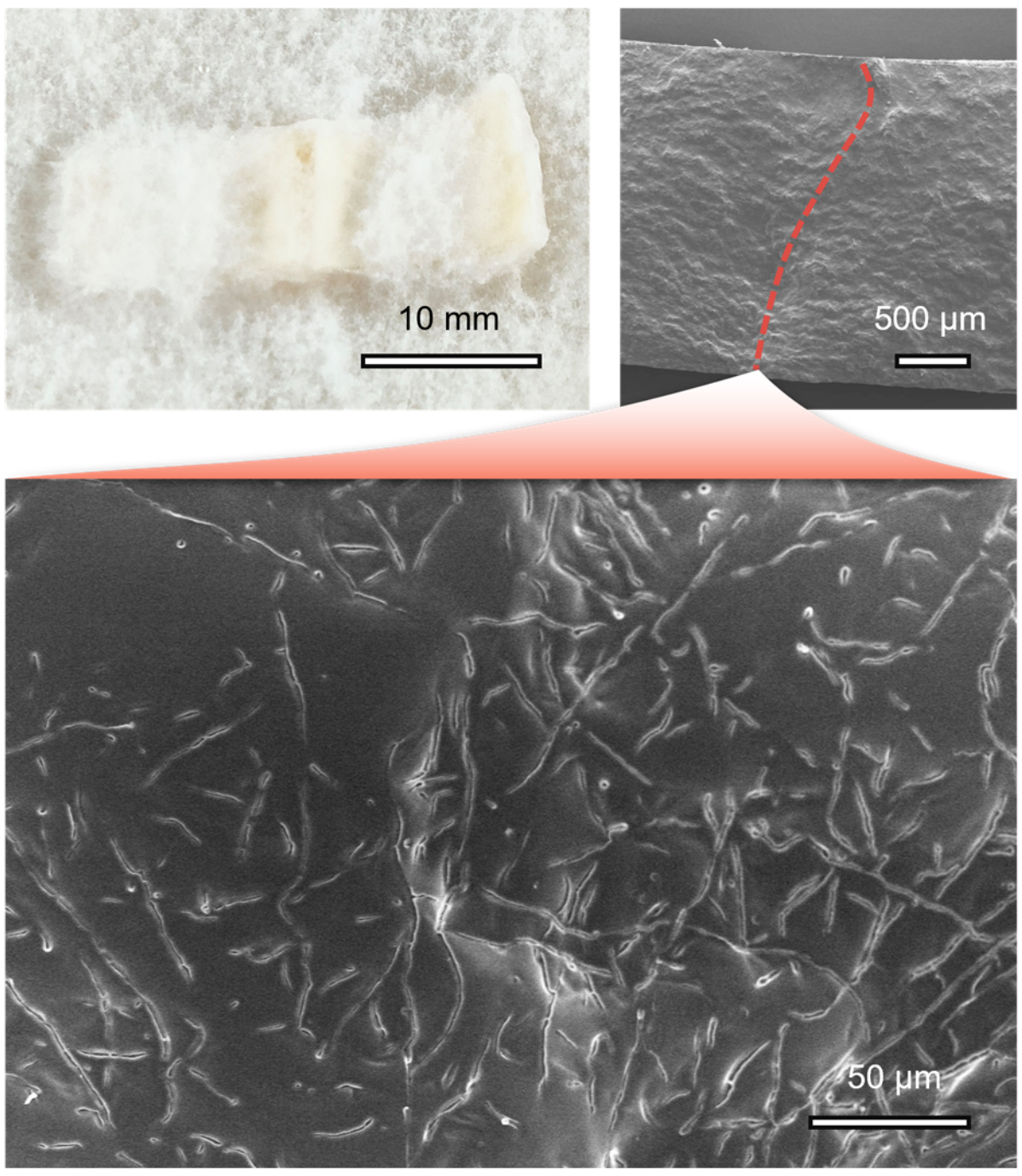

Supplementary Fig. 68 Optical and SEM images of self-healed TV@P-G on MEA solid medium for 1 week. Locally amplified SEM image of healed fracture shows that regenerative mycelium bonded and repaired the fracture. The experiment was repeated independently four times (n = 4) with similar trends, and a representative experiment is shown.

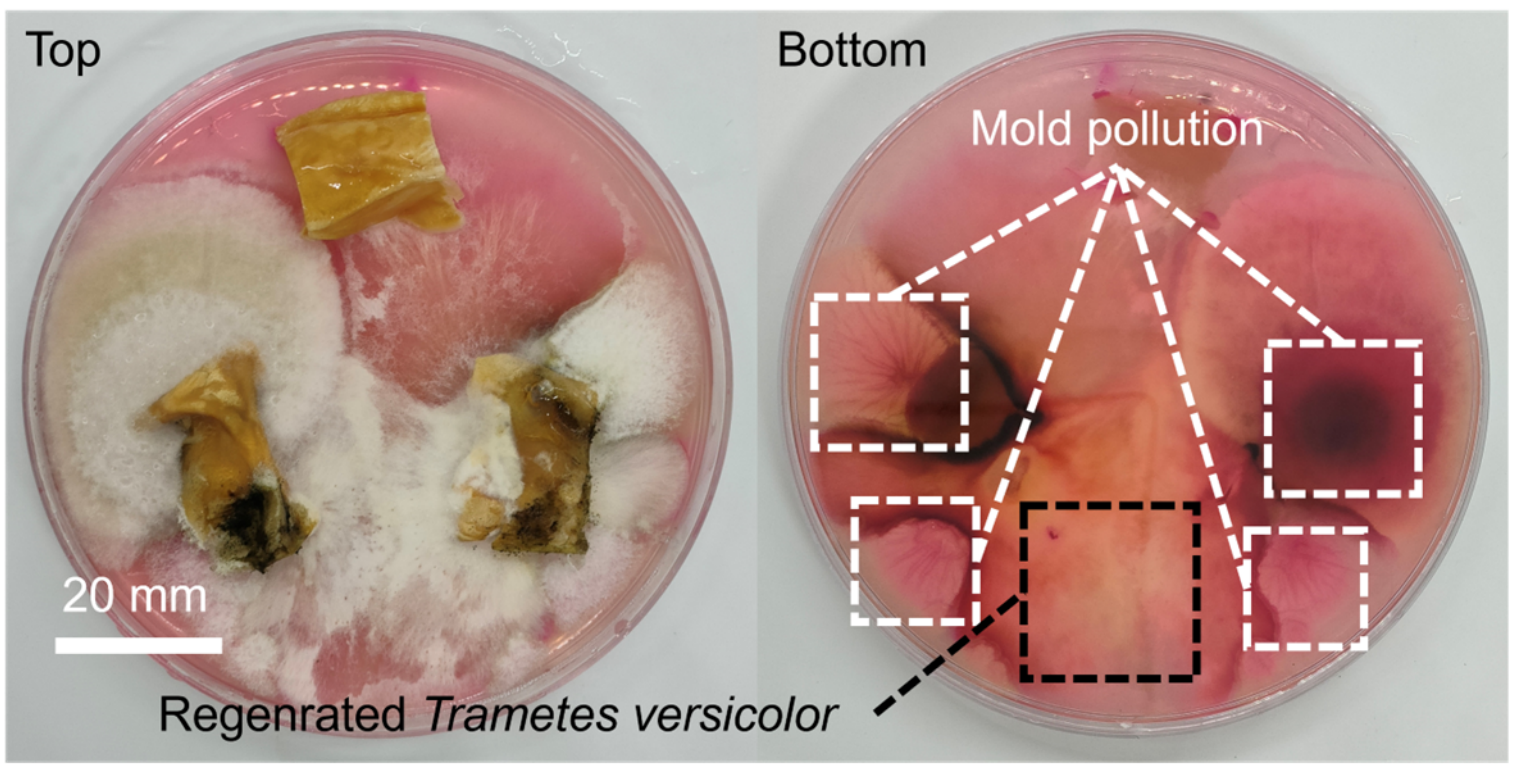

Supplementary Fig. 69 Optical images of dormant TV@P-G stored outside of sterility (25°C, 20%RH) for 1 year regeneration on rose Bengal medium at 25 °C for 10 d. The experiment was repeated independently three times (n = 3) with similar trends, and a representative experiment is shown.

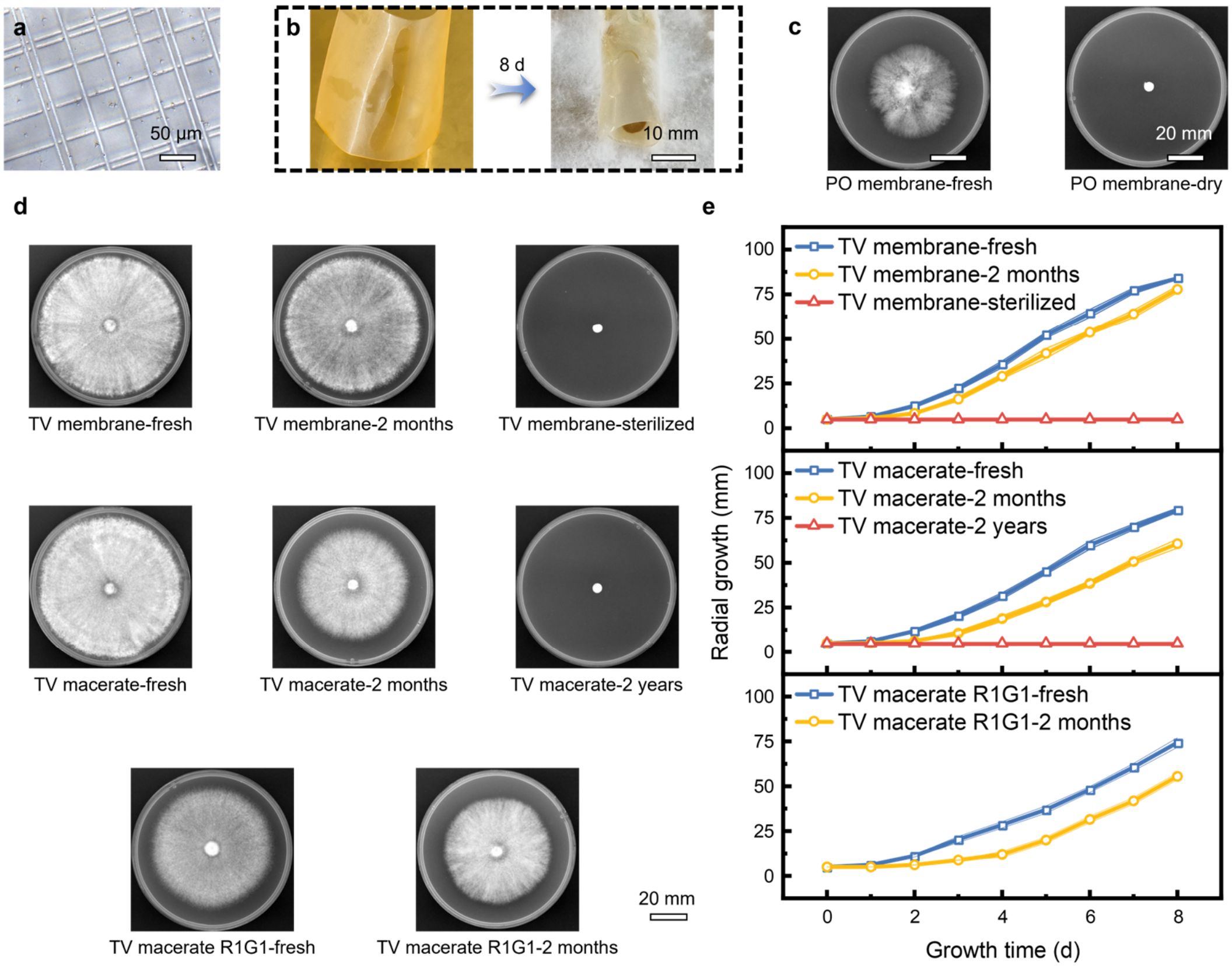

Supplementary Fig. 70 Regeneration of spores and hyphae. **a,** Optical images of TV spores (chlamydospores) on a hemacytometer. **b**, Regeneration of spores embedded in 10%PVA+10%Gly membrane (3×3 cm), which grew on PDA medium at 28 °C, 85% RH for 8 d. The experiment was repeated independently three times (n = 3) with similar trends, and a representative experiment is shown. **c**, Optical images of fresh and 1-month dry storage (25°C, 20% RH) PO mycelium membrane (d = 5 mm) regeneration, which grew on PDA medium at 28 °C, 85% RH for 8 d. **d**, Optical images of TV mycelium material (d = 5 mm) regeneration, which grew on PDA medium at 28 °C, 85% RH for 8 d. **e**, Radial growth curves of mycelium materials (d = 5 mm) on PDA medium at 28 °C, 85% RH for 8 d, n = 3. Error bars (mean ± SD) were connected and visualized as a shadow effect around the data points.

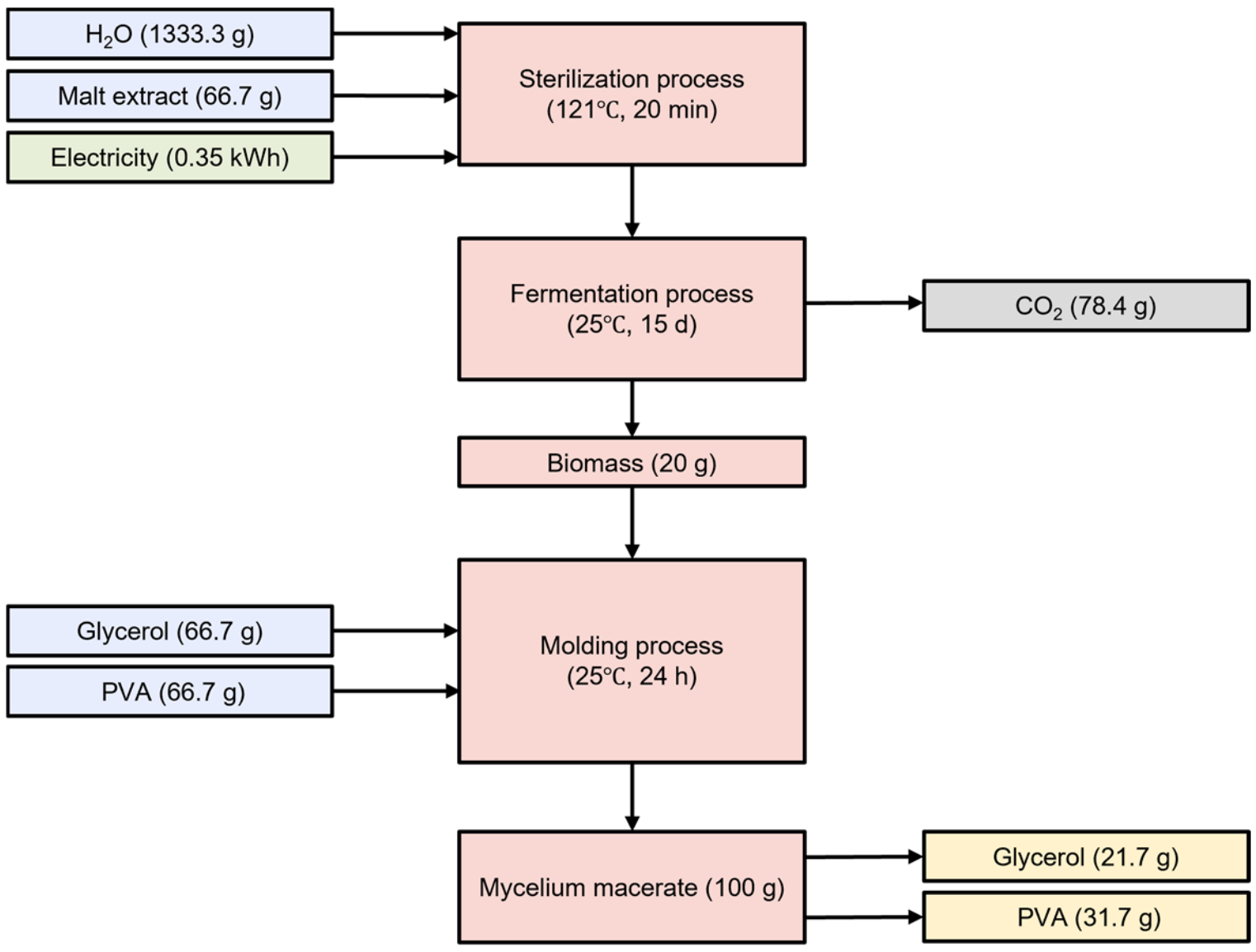

Supplementary Fig. 71 Flowchart of production stages for mycelium macerate (10%PVA+10%Gly). The quantities of raw materials and energy consumption in the flowchart are 10 times that of the experimental quantities.

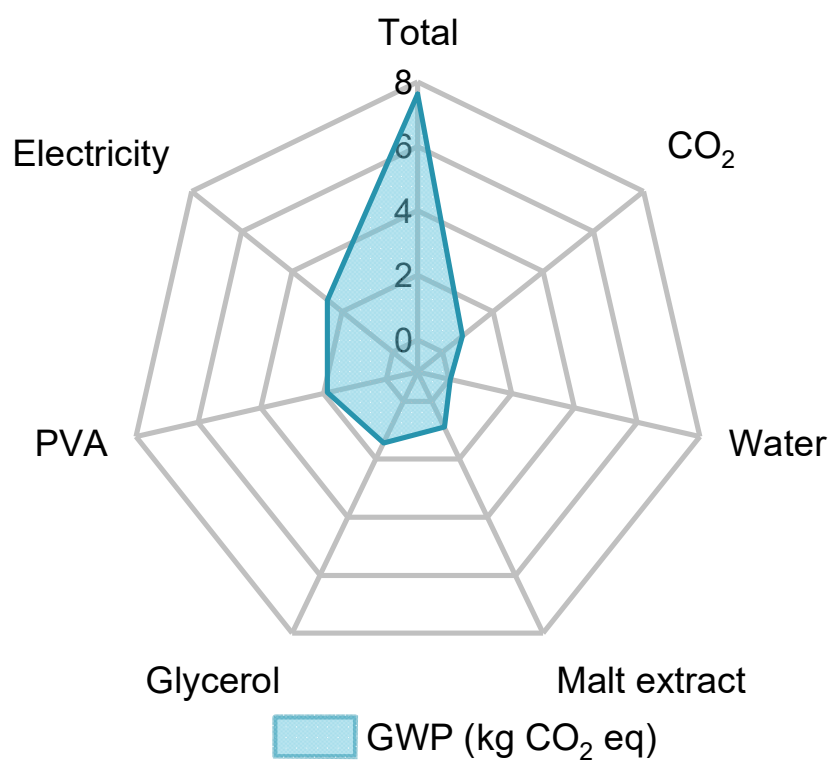

Supplementary Fig. 72 Contribution of different raw materials and energy consumption to GWP indicators of mycelium macerate (10%PVA+10%Gly).

Discussion of LCA results

According to the GWP assessment outcomes, the total GWP associated with producing 1 kg of the product is 7.63 kg CO_2-eq. Notably, electricity consumption emerges as the largest source of carbon emissions, contributing 2.59 kg CO_2-eq, which accounts for 33.9% of total emissions. This underscores that the carbon intensity of electricity utilized in the production process is a critical driving factor. Optimizing process parameters to reduce energy consumption or transitioning to renewable electricity sources represents the most effective strategy for mitigating the carbon footprint of this process. Second, the LCA-PVA process (as a raw material input) contributes 1.88 kg CO_2-eq, representing 24.6% of total emissions and ranking as the second-largest emission source. PVA production itself is characterized by high energy demand (requiring 1.07 kWh of electricity and 5 kg of steam for 1 kg PVA) and direct CO_2 emissions (2.5 kg). Thus, selecting PVA alternatives or suppliers with lower environmental impacts is pivotal for reducing the overall carbon

footprint. Third, glycerol, wheat flour, and direct CO_2 emissions from the process (784 g) contribute approximately 18.88%, 11.78%, and 10.28% of total emissions, respectively, while the environmental impact of water is negligible. More importantly, by taking advantage of the ability of fungi as natural decomectors, the culture medium can be replaced from high-carbon-footprint nutritional products to sugar-containing organic waste. For instance, in our laboratory, discarded mycelium was crushed and sterilized to produce culture medium for cultivating new mycelium, achieving the reuse of high-carbon-footprint materials and reducing the energy consumption in the preparation process of new batches of mycelium materials.

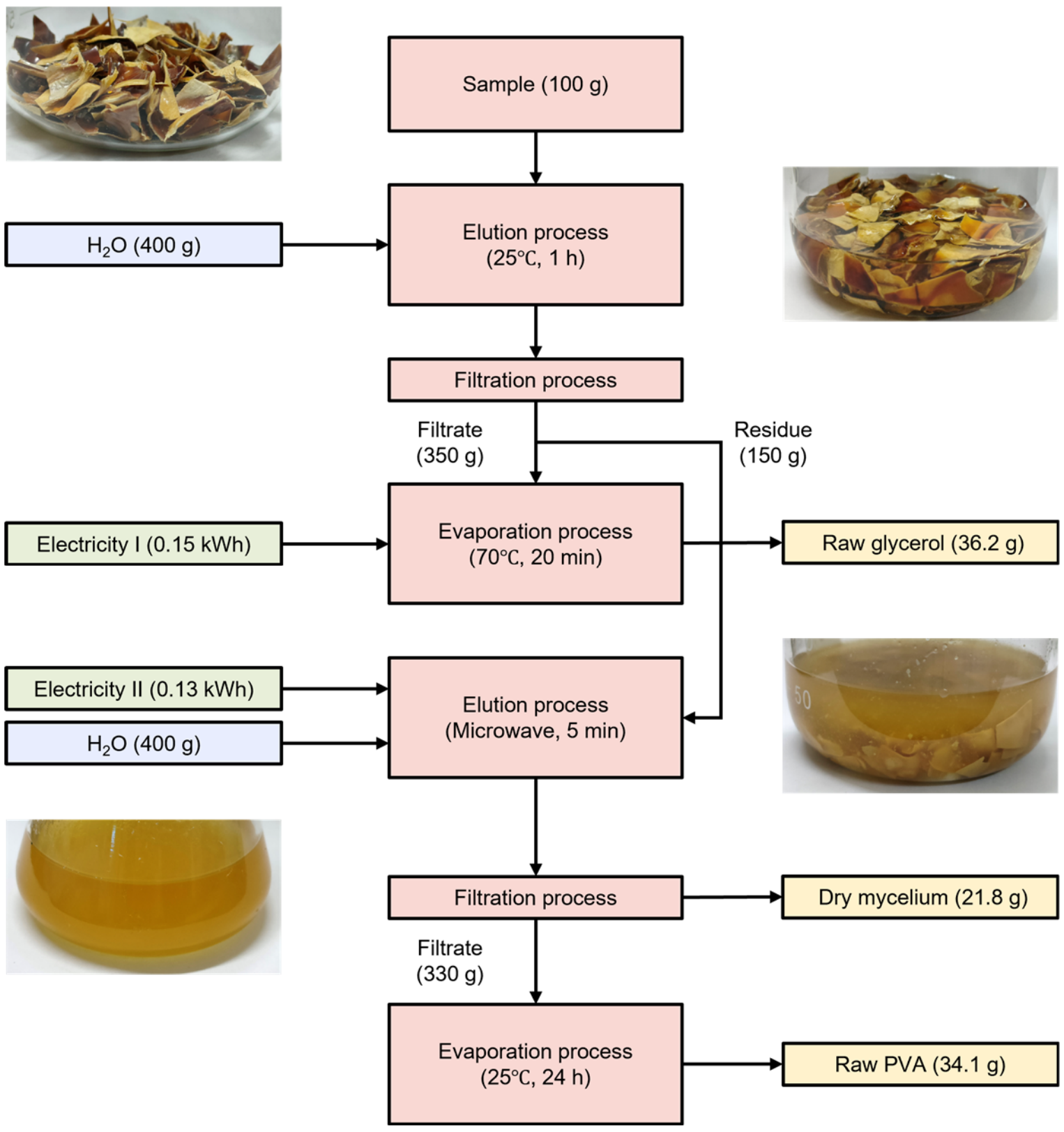

Supplementary Fig. 73 Flowchart of recycling stages for mycelium macerate (10%PVA+10%Gly). The quantities of materials and energy consumption in the flowchart are 10 times that of the experimental quantities.

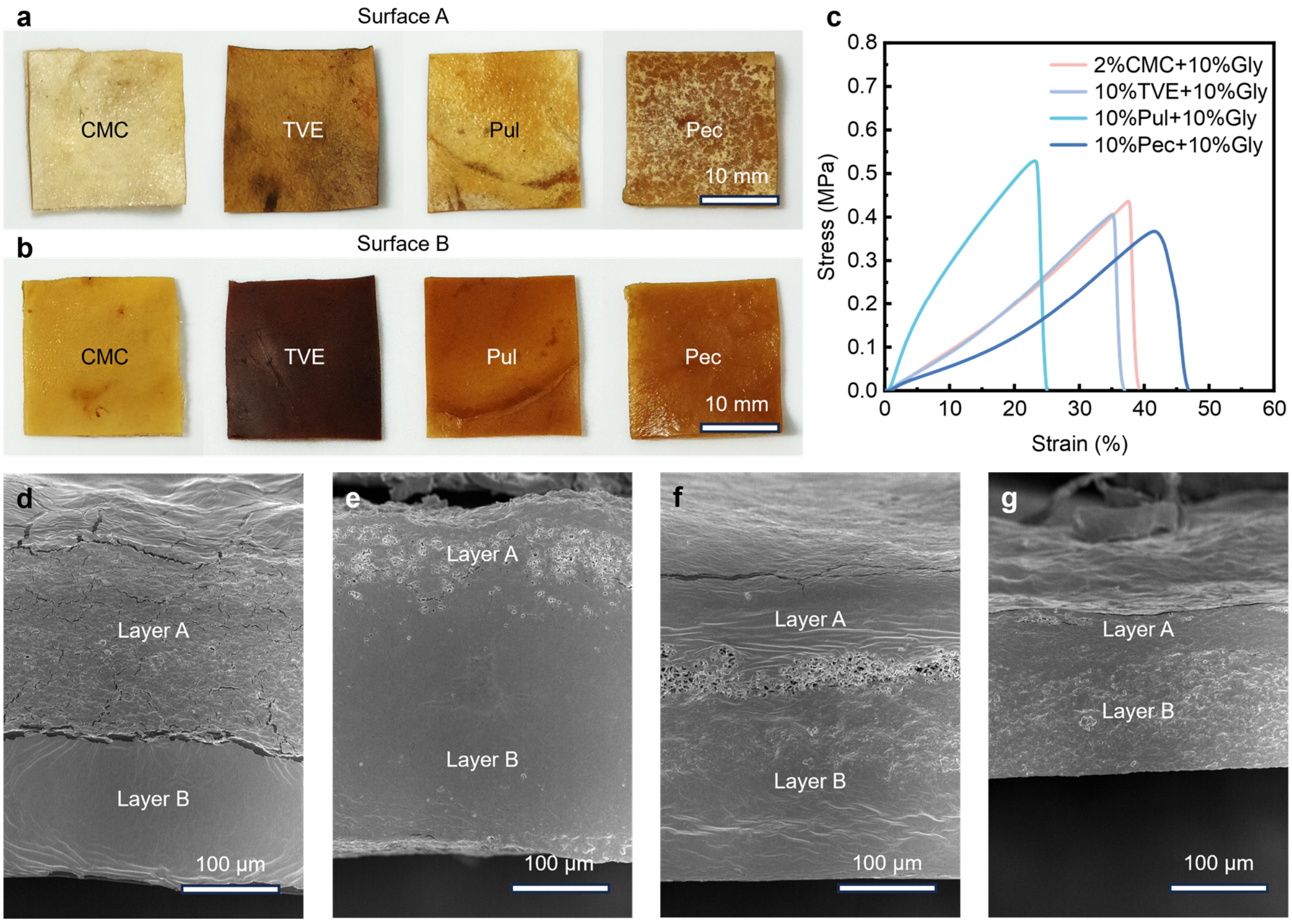

Supplementary Fig. 74 Mycelium macerates bonded with polysaccharides. **a**, **b**, Optical images of Janus mycelium macerates TV@PSac-G viewed from surface A (**a**) and B (**b**). **c**, Tensile stress-strain curves of *Trametes versicolor* membrane treated with polysaccharides, and 10% glycerol. CMC: Carboxymethyl cellulose; TVE: polysaccharides from Trametes; Pul: pullulan; Pec: pectin. **d**-**g**, SEM images of TV@PSac-G cross section. TV@CMC-G, (**d**); TV@TVE-G, (**e**); TV@Pul-G, (**f**); TV@Pec-G, (**g**). Data are representative of at least three independent replicates (n = 3) with similar results.

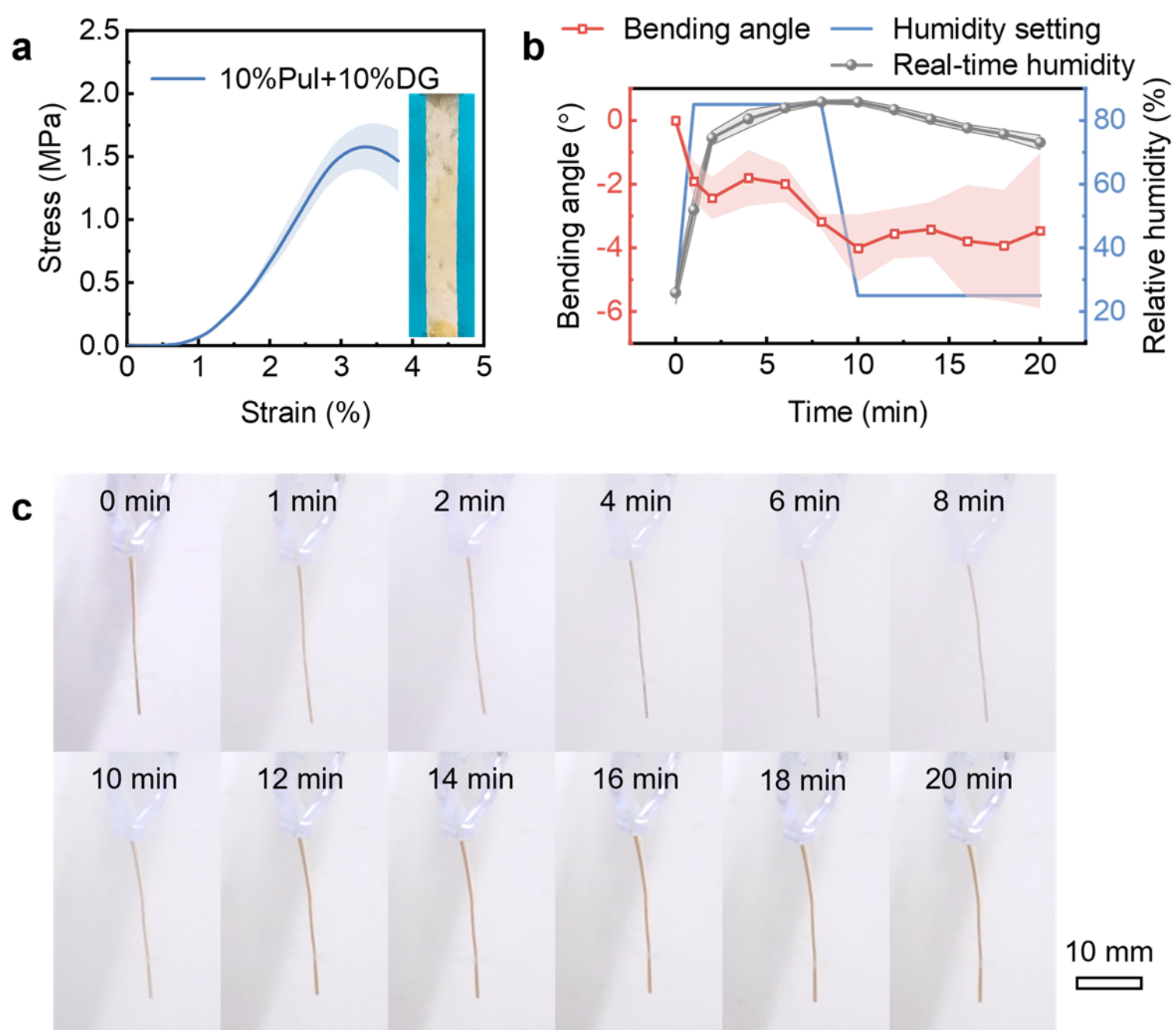

Supplementary Fig. 75 Mechanicl properties and hygroscopic deformation of all-biobased TV@Pul-DG macerate. **a**, Tensile curve and image of TV 10%Pul+10%DG specimen. Data are expressed as mean ± SEM, n = 3. **b**, Bending angle changes of TV@Pul-DG specimen (5 × 25 mm) in single fixed humidity response experiments, n = 3. Humidity was controlled with humidity chamber. Error bars (mean ± SD) were connected and visualized as a shadow effect around the data points. **c**, Optical images of TV@Pul-DG specimen (5×25 mm) with single fixed end deforming in hygroscopic process controlled in a humidity chamber, where humidity changes was set and recorded as (**b**).

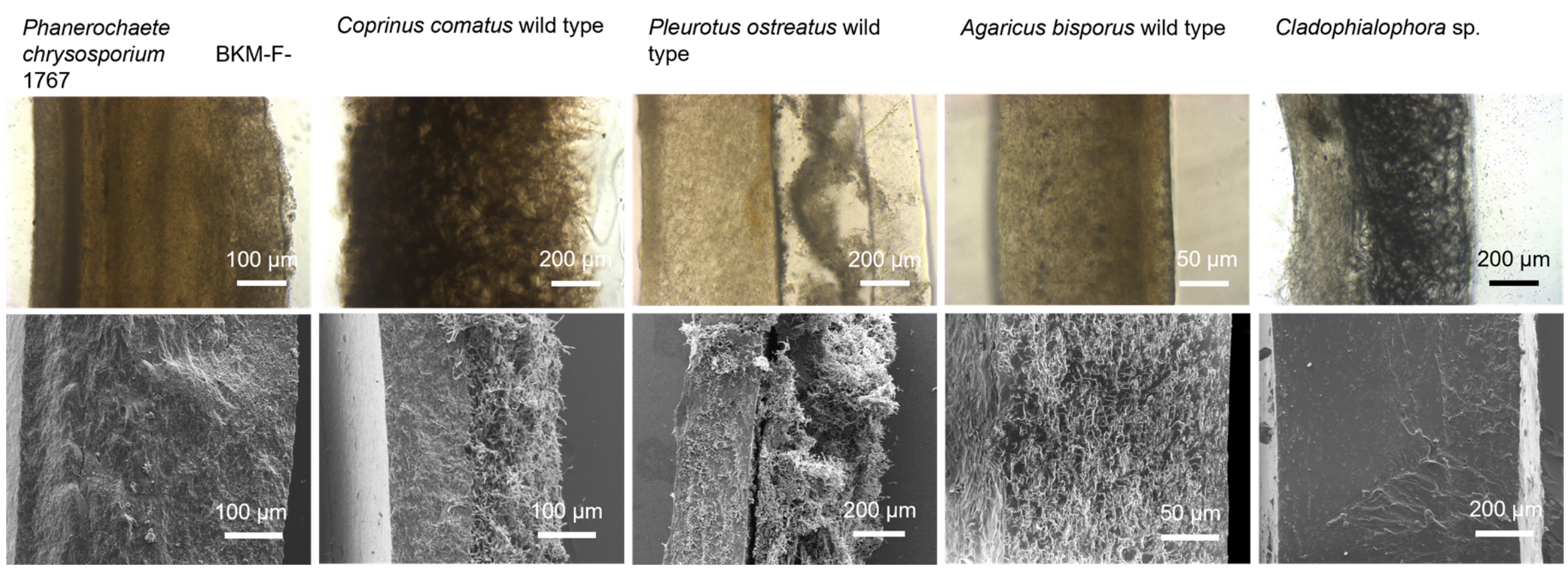

Supplementary Fig. 76 Optical and SEM images of cross section of PC@P-G, CC@P-G, PO@P-G, AB@P-G and CP@P-G macerates, of which layer A was set toward the right. Data are representative of at least three independent replicates (n = 3) with similar results.

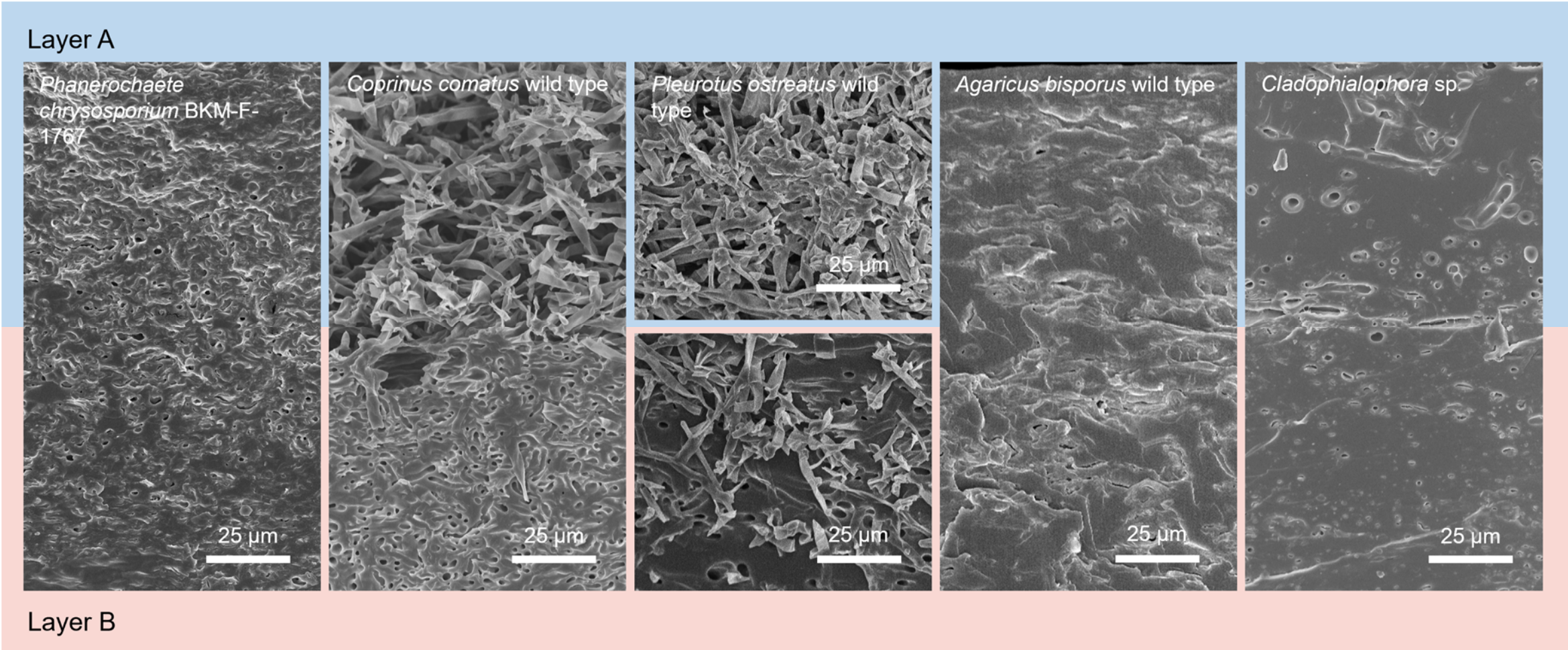

Supplementary Fig. 77 SEM images of magnified Janus structure of PC@P-G, CC@P-G, PO@P-G, AB@P-G and CP@P-G. CC@P-G and PO@P-G exhibited obvious Janus structure, while AB@P-G and CP@P-G shown Janus structure with inapparent morphology and porosity difference. Data are representative of at least three independent replicates (n = 3) with similar results.

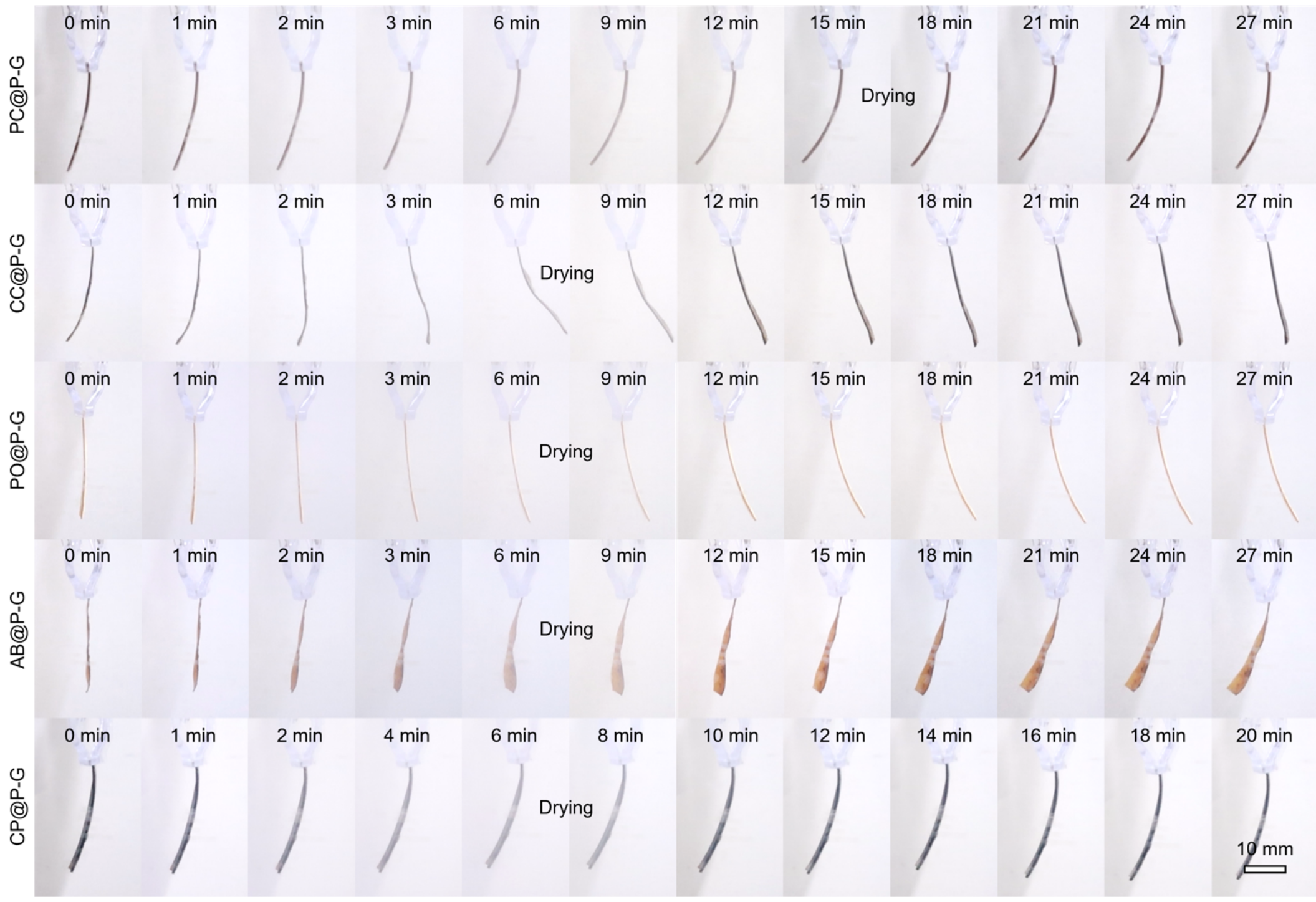

Supplementary Fig. 78 Optical images of PC@P-G, CC@P-G, PO@P-G, AB@P-G and CP@P-G with single fixed end deforming in hygroscopic process conducted with humidity chamber. Time marked in black and white respectively correspond to deformation and recovery process. The opposite curvature may involve multi-factor asymmetries between layers of different species, including density gradient differences, fractal dimension asymmetries, etc. n = 3 independent experiments.

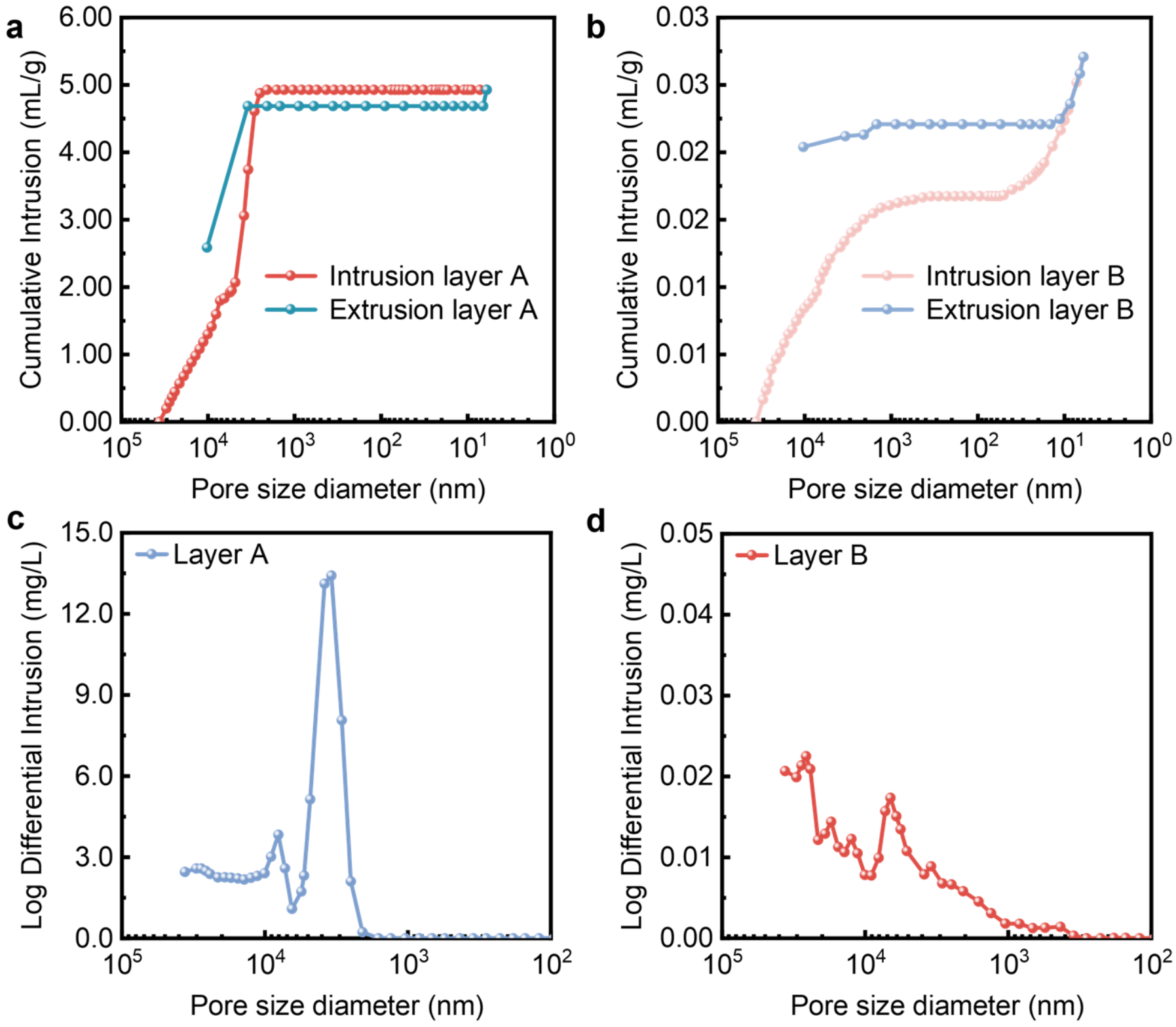

Supplementary Fig. 79 Mercury intrusion results of PO@P-G macerate at dry state. **a**, **b**, Cumulative intrusion and extrusion versus pore diameter for layer A (**a**) and B (**b**). c, d, Mercury intrusion pore size distribution curves of layer A (**c**) and B (**d**).

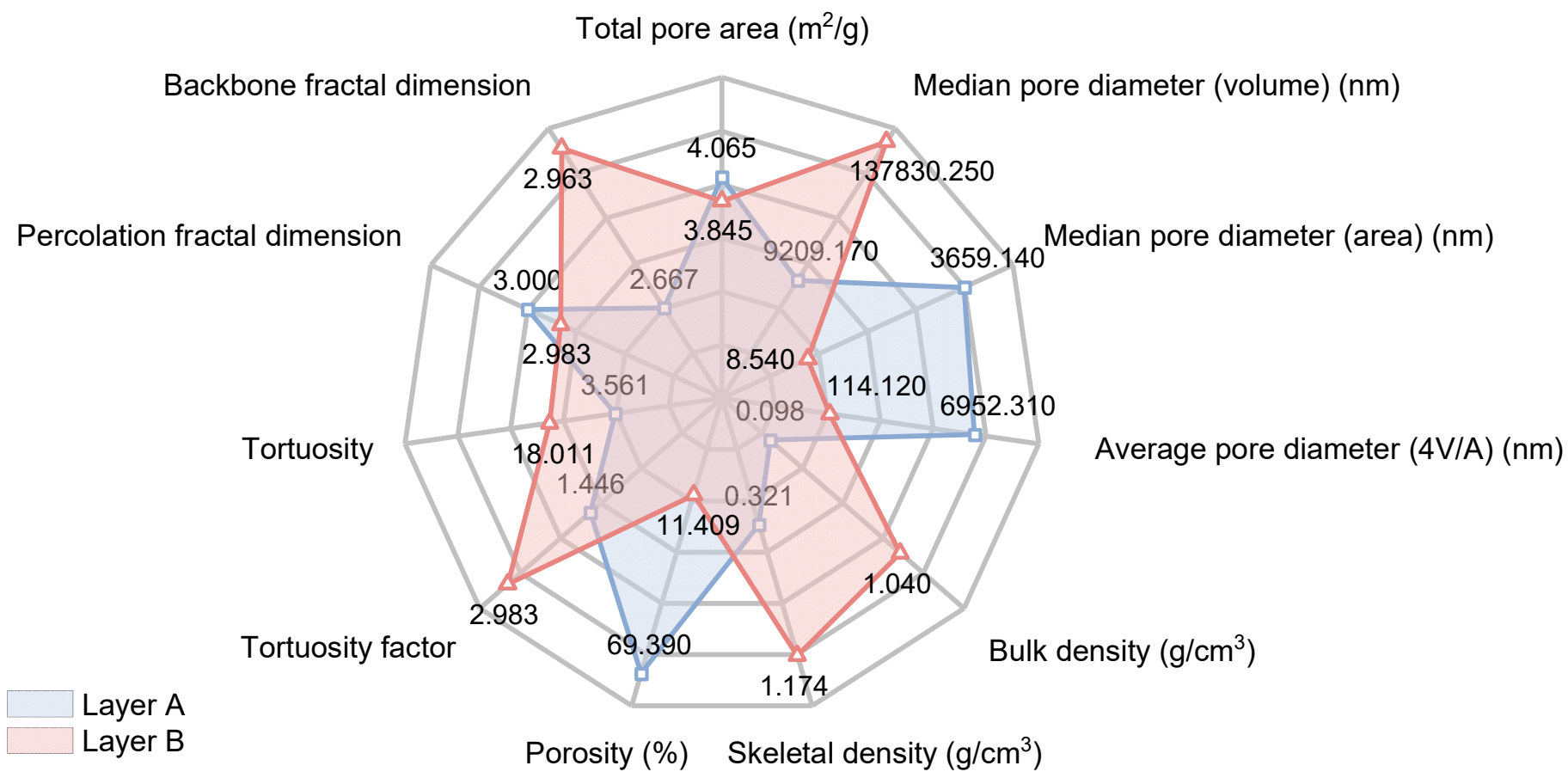

Supplementary Fig. 80 Comparison of pore structure parameters between layer A and B of PO@P-G macerate.

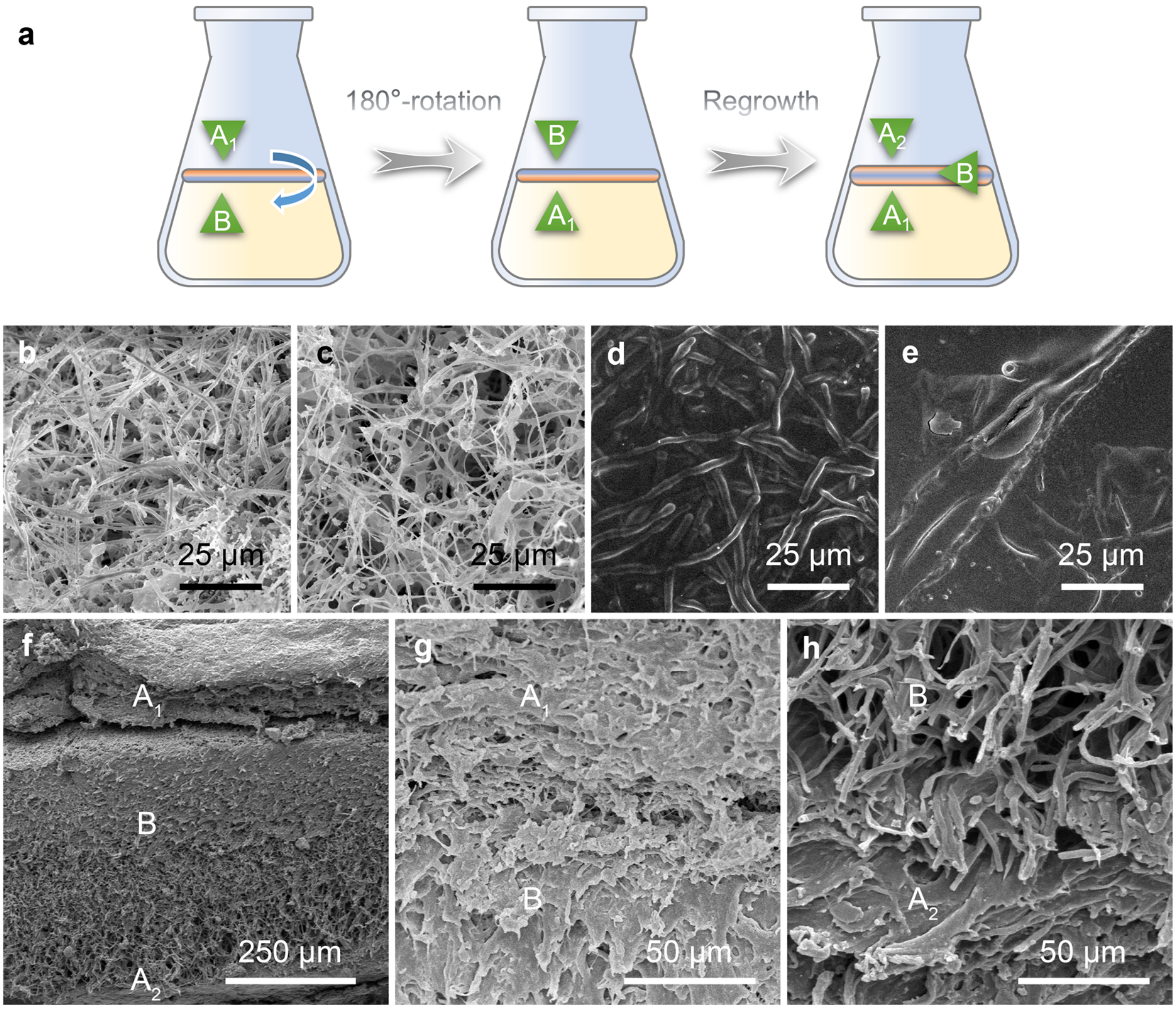

Supplementary Fig. 81 Sandwich-like A_1BA_2 architecture of TV@P-G macerate via turn-over growth. **a**, Schematic illustration of turn-over growth. **b**, **c**, SEM images of surface A_1 (**b**) and A_2 (**c**) morphology of mycelium membrane. **d**, **e**, SEM images of surface A_1 (**d**) and A_2 (**e**) morphology of mycelium macerate. **f**, SEM image of mycelium macerates' cross section. **g**, **h**, SEM images of amplified interface between A_1B (**g**) and BA_2 (**h**), respectively. Data are representative of at least three independent replicates (n = 3) with similar results.

Supplementary text of discussion

MIP testing was performed to explore the relationship between hygroscopic deformation and density gradients in different fungal species. Separated layer A and B of PO@P-G macerate showed remarkable differences in mercury intrusion and extrusion processes (Supplementary Fig. 79a, b), indicating that layer A of PO macerate displayed a much higher porosity than layer B. Pore diameter distribution (Supplementary Fig. 79c, d) showcased an inversed result that layer A compared with specie TV. We quantified pore structure parameters using AutoPore V 9600 software and compared these parameters in radar chart (Supplementary Fig. 80). With a much less porosity of 11.409%, layer B of specie PO showed much more bulk density and skeleton density than layer B. Density results from MIP showed that both species TV and PO share the same gradient orientation, density (layer A) < density (layer B). The opposite deformation remained unexplained. Notably, a reverse difference in backbone fractal dimension of specie PO, (a backbone fractal dimension 2.963 for layer B, 2.667 for layer A in specie PO; while 2.940 for layer A, 2.758 for layer B in specie TV), brought about a speculation that fractal dimension asymmetry programed deformation behaviors in biological Janus structures. In the case of similar percolation fractal dimensions, a higher backbone fractal dimension possibly correlates with enhanced hygroscopic responsiveness. Thus, even when density gradients are identical, the layer with higher backbone fractal dimension becomes the dominant active layer under external stimuli, dictating the bending direction. In specie TV, the layer A dominates; while in specie PO, layer B takes over resulting in opposite curvatures.

In application scenarios such as alternation of dry and wet, we also demonstrated such type of Janus mycelium macerates can perform bending and one-way walking (Supplementary Fig. 82). Then we are convinced that the slow action of macerates shows potential in smooth humidity regulation. Hygroscopic deformation of macerates can also collaborate in a cross-disciplinary manner with other cutting-edge technologies, including self-powered devices[16], and even software. Derived from mushroom, is ecofriendly and easy to fabrication, as a promising alternative for unsustainable devices. For the sake of environment, the bonding agent PVA and plasticizer glycerol we selected are both bio-degradable (Supplementary Fig. 83, 84). Beyond previously reported actuators[16, 17, 18], dormant type living material enables regeneration and subculturing, which are unique concepts of biology. Our study endows environmental responsive materials with livingness and sustainability of fungi, overcoming the material waste and recycling problem of conventional actuators with high price and complicated procedure. Waste can not only act like seeds to regenerate new mycelium membrane, but also be recycled as raw materials of culture medium. Newborn macerates perform better mechanical properties, of which the underlying mechanism remains clear.

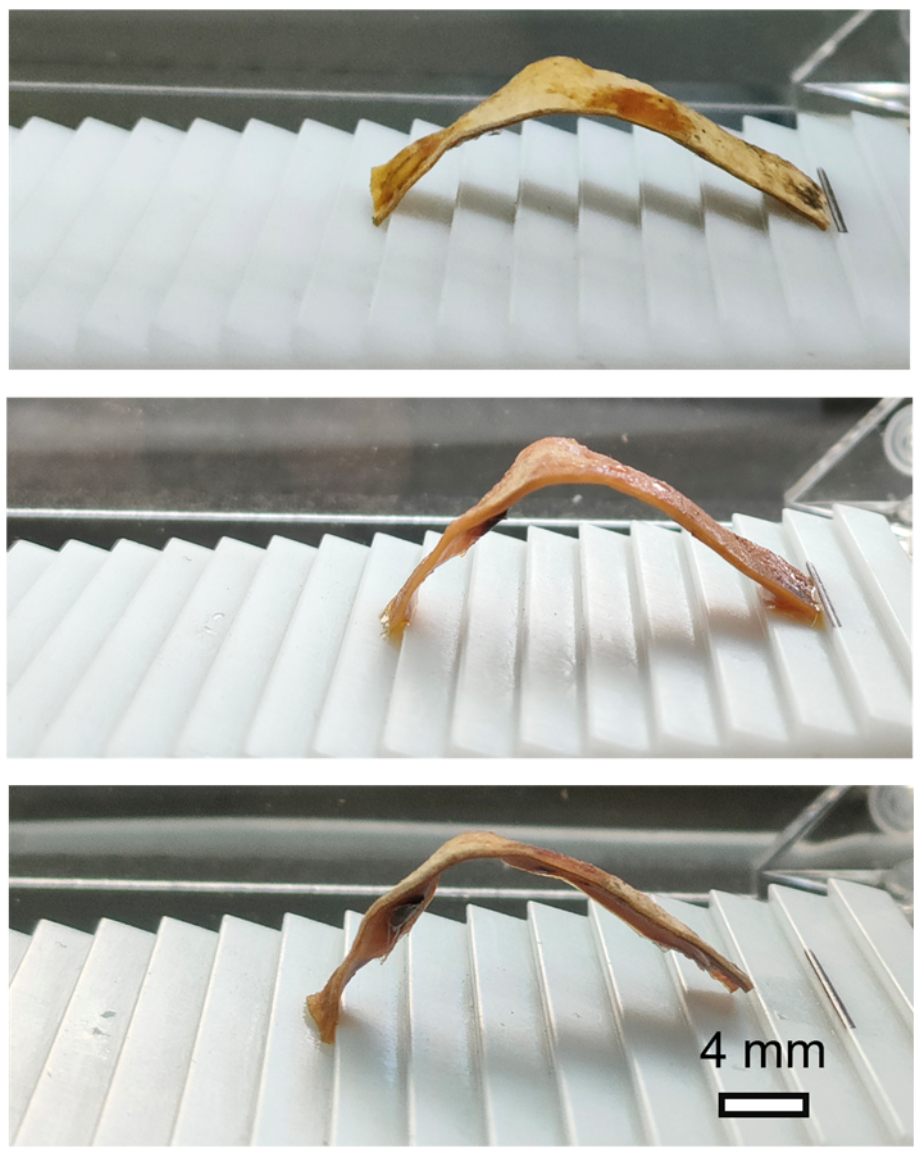

Supplementary Fig. 82 Locomotion of TV@P-G on a ratchet surface controlled with alternation of dry and wet.

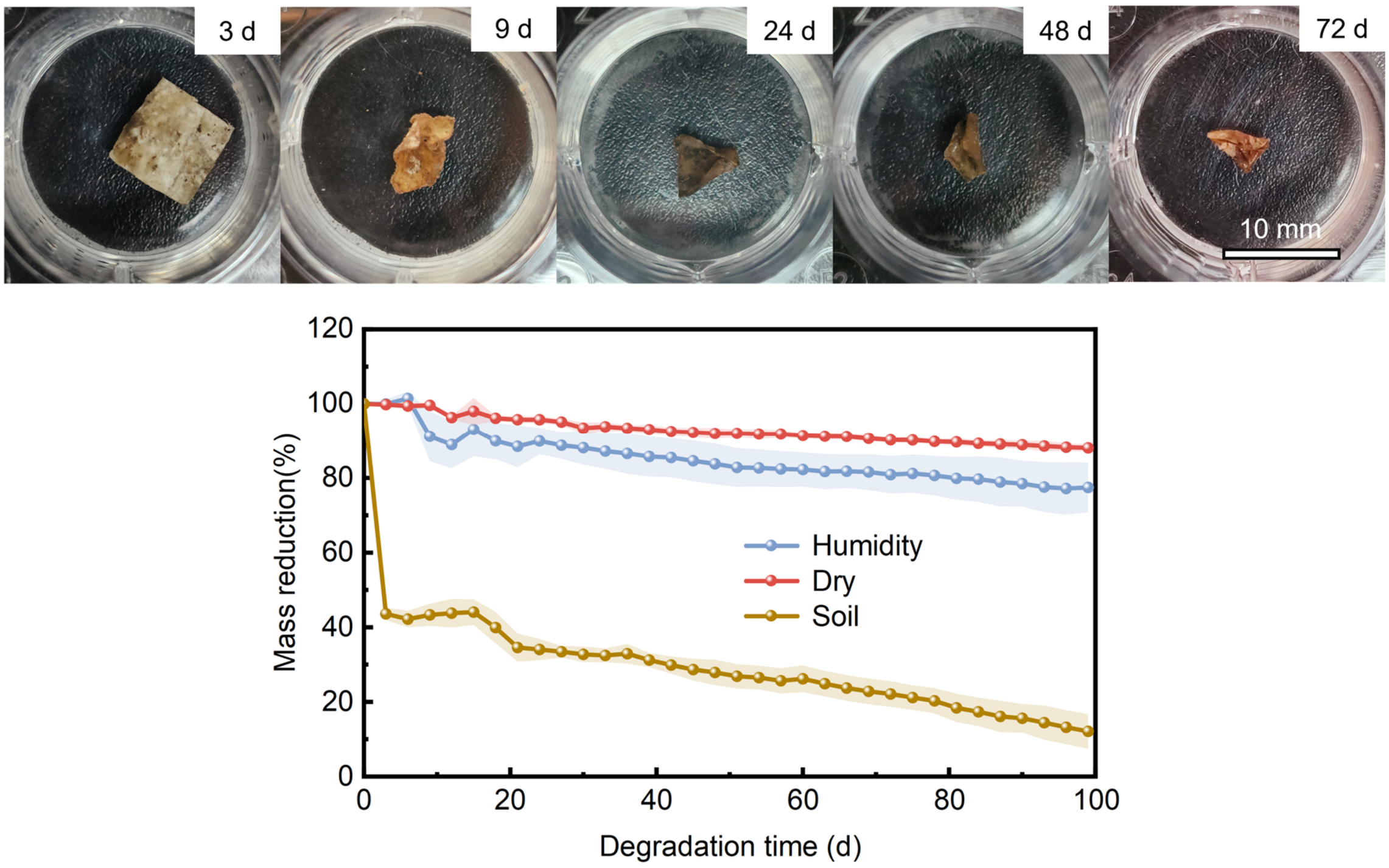

Supplementary Fig. 83 Soil degradation and weight reduction curve of TV@P-G, n = 3. After 100 days, mycelium macerates broke down into fragments until completely degraded impracticable to weigh. Error bars represented mean ± SD, were connected and visualized as a shadow effect around the data points.

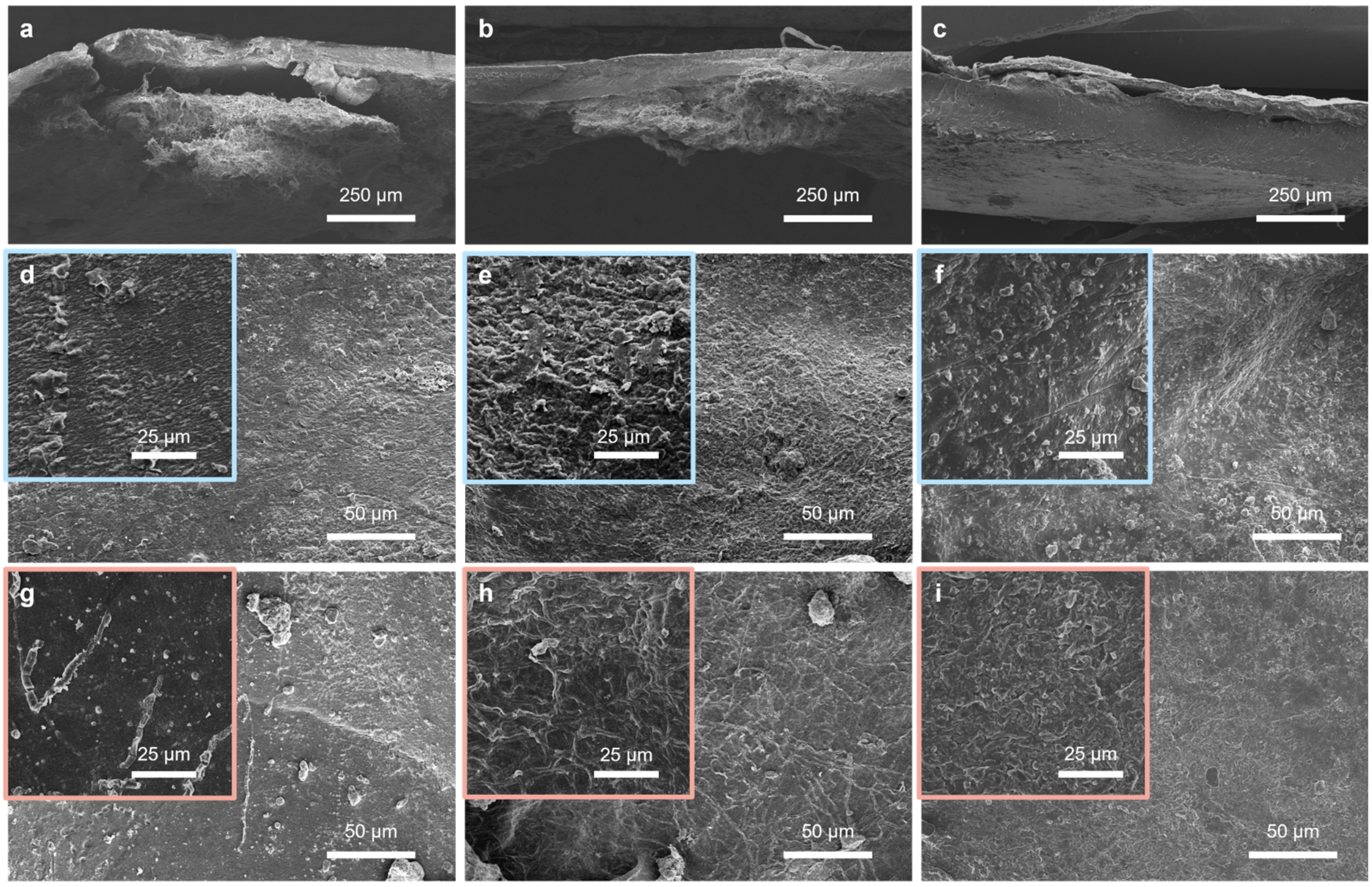

Supplementary Fig. 84 SEM images of degraded TV@P-G macerate. **a**, **d**, **g**, 3-day degradation; **b**, **e**, **h**, 24-day degradation; **e**, **f**, **i**, 72-day degradation **a**, **b**, **c**, Cross section. **d**, **e**, **f**, Surface A, **g**, **h**, **i**, Surface B.

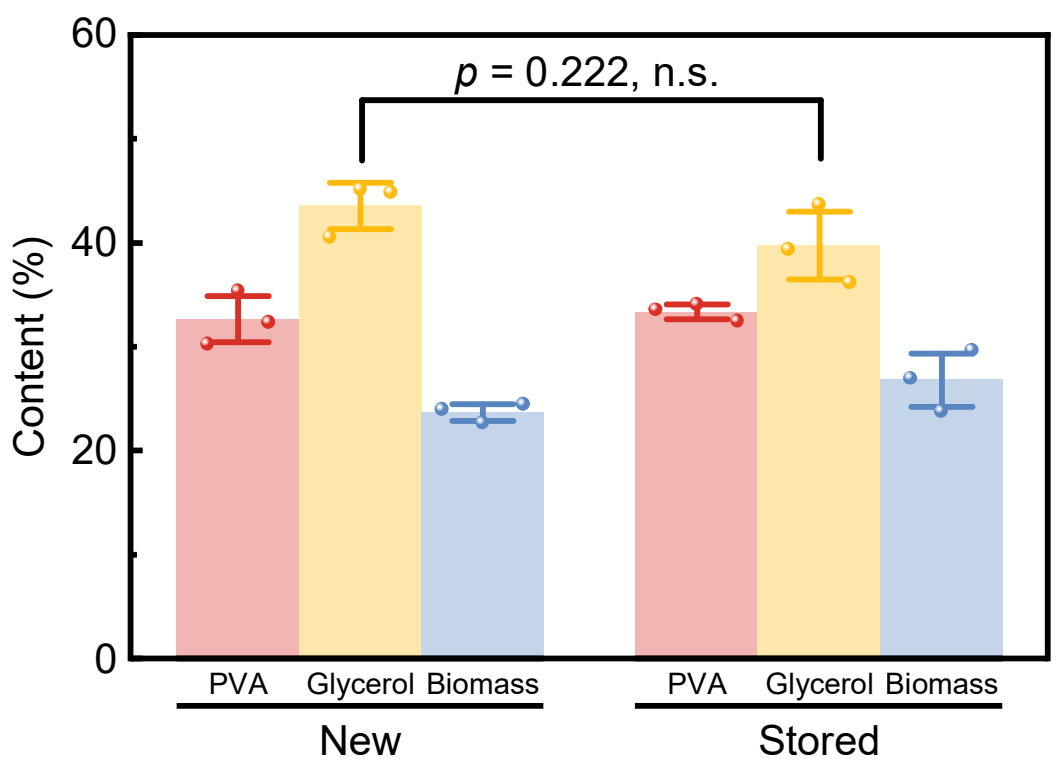

Supplementary Fig. 85 Percentage composition of PVA, glycerol and biomass in newly fabricated and 1-year stored TV@P-G macerates, n = 3. The significance of differences between the two groups was assessed using Student's t-test. For t-tests: Cohen's d was calculated as ($mean_1$ - $mean_2$) / pooled standard deviation. Difference in glycerol content: $p = 0.222$, Cohen' d = 0.45.

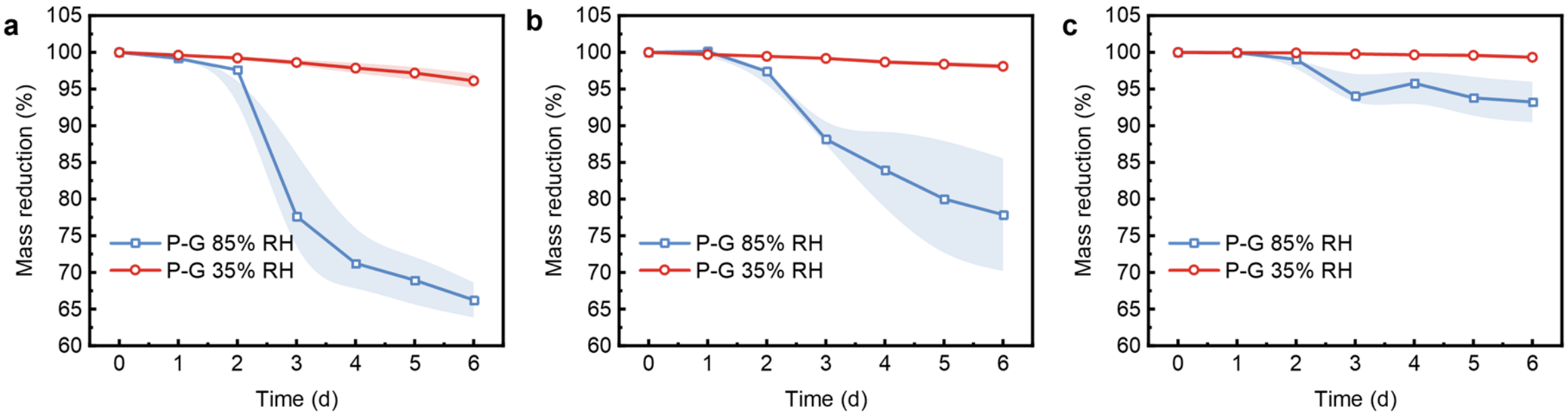

Supplementary Fig. 86 Migration of plasticizers. Mass reduction curves (means ± SD) of TV@P-G pressed by filter paper (**a**), PVA (**b**) and PTFE (**c**) exposed to 25℃, 35% and 85% RH, attributable to plasticizers migration, n = 3. Data are expressed as mean ± SD. Error bars were connected using B-spline basis function and visualized as a shadow effect around the data points.

Supplementary references

1. Danninger D, Pruckner R, Holzinger L, Koeppe R, Kaltenbrunner M. MycelioTronics: Fungal mycelium skin for sustainable electronics. *Science Advances* **8**, eadd7118 (2022).
2. Raman J, Kim D-S, Kim H-S, Oh D-S, Shin H-J. Mycofabrication of Mycelium-Based Leather from Brown-Rot Fungi. *Journal of Fungi* **8**, 317 (2022).
3. Shao G*, et al.* Green and sustainable biomaterials: Edible bioplastic films from mushroom mycelium. *Food Hydrocolloids* **146**, 109289 (2024).
4. Wijayarathna ERKB*, et al.* Fungal textile alternatives from bread waste with leather-like properties. *Resources, Conservation and Recycling* **179**, 106041 (2022).
5. Nguena-Dongue B-N, Amobonye A, Muniyasamy S, Pillai S. Mycelium-based leather-like material from Absidia koreana grown on agro-residues: Process optimisation, functionalisation, and material performance. *Sustainable Materials and Technologies* **48**, e01970 (2026).
6. Merisani AEF, Economou CN, Nanni G, Athanassiou A. Chemical conversion of Pleurotus ostreatus mycelium into biodegradable films with tailored properties. *ACS Applied Engineering Materials* **4**, 1802-1812 (2026).
7. Appels FVW, van den Brandhof JG, Dijksterhuis J, de Kort GW, Wösten HAB. Fungal mycelium classified in different material families based on glycerol treatment. *Communications Biology* **3**, 334 (2020).
8. d'Errico A, Land CA, Wissing M, Richrath RB, Wösten HAB. Polyglyceryl-4 caprate and polyglycerol-3 provide low mobility and enhance ductility in Schizophyllum commune mycelium materials. *Colloids and Surfaces A: Physicochemical and Engineering Aspects* **719**, 137055 (2025).
9. Elsacker E, Zhang M, Dade-Robertson M. Fungal Engineered Living Materials: The Viability of Pure Mycelium Materials with Self-Healing Functionalities. *Advanced Functional Materials* **33**, 2301875 (2023).
10. Bai L*, et al.* Hygrothermic Wood Actuated Robotic Hand. *Advanced Materials* **35**, 2211437 (2023).
11. Li K*, et al.* Engineered living materials grown from programmable Aspergillus niger mycelial pellets. *Materials Today Bio* **19**, 100545 (2023).
12. Wang H, Wang W, Tao J, Liu S, Hou X, He C. Anchoring mycelium on CNTs to make strong and smart self-regenerative composite materials. *Composites Part B: Engineering* **287**, 111861 (2024).
13. Sinha A*, et al.* Living Fiber Dispersions from Mycelium as a New Sustainable Platform for Advanced

Materials. *Advanced Materials* **37**, 2418464 (2025).

14. Petchsong P, Boonyuen N, Kwantong P, Nuankaew S, Chuaseeharonnachai C, Ummartyotin S. Development of mycelium-reinforced dialdehyde cellulose–polyvinyl alcohol composites for emerging high-performance sustainable materials. *Synthetic and Systems Biotechnology* **13**, 545-558 (2026).
15. Wang H*, et al.* Fabrication of Living Entangled Network Composites Enabled by Mycelium. *Advanced Science* **11**, 2309370 (2024).
16. Mao T*, et al.* Engineering Covalent Organic Frameworks with Polyethylene Glycol as Self-Sustained Humidity-Responsive Actuators. *Angewandte Chemie International Edition* **62**, e202216318 (2022).
17. Chen Z*, et al.* Dried bonito flakes-inspired moisture-responsive actuator with a gradient structure for smart devices. *Journal of Materials Science & Technology* **167**, 152-160 (2023).
18. Lin S*, et al.* Overcoming response-recovery kinetic mismatch in soft actuators via synergistic material design for multimodal moisture-responsive actuation. *Nano Energy* **140**, 110998 (2025).
