# Supplementary material for: Tailored living mycelium macerate for reproducible flexible actuators
Source: Nat Commun. 2026 Aug 12;17:9682. doi: 10.1038/s41467-026-76623-z (PMC13558639; doi:10.1038/s41467-026-76623-z)
Supplement: Supplementary file 2 [file 41467_2026_76623_MOESM7_ESM.pdf]

nature portfolio

Corresponding author(s): Xiaoman Liu and Xin Huang

Last updated by author(s): Jul 6, 2026



# Reporting Summary

Nature Portfolio wishes to improve the reproducibility of the work that we publish. This form provides structure for consistency and transparency in reporting. For further information on Nature Portfolio policies, see our Editorial Policies and the Editorial Policy Checklist.

## Statistics

For all statistical analyses, confirm that the following items are present in the figure legend, table legend, main text, or Methods section.

| n/a | Confirmed | |
|---|---|---|
| ☐ | ☒ | The exact sample size ($n$) for each experimental group/condition, given as a discrete number and unit of measurement |
| ☐ | ☒ | A statement on whether measurements were taken from distinct samples or whether the same sample was measured repeatedly |
| ☐ | ☒ | The statistical test(s) used AND whether they are one- or two-sided<br>*Only common tests should be described solely by name; describe more complex techniques in the Methods section.* |
| ☒ | ☐ | A description of all covariates tested |
| ☐ | ☒ | A description of any assumptions or corrections, such as tests of normality and adjustment for multiple comparisons |
| ☐ | ☒ | A full description of the statistical parameters including central tendency (e.g. means) or other basic estimates (e.g. regression coefficient) AND variation (e.g. standard deviation) or associated estimates of uncertainty (e.g. confidence intervals) |
| ☐ | ☒ | For null hypothesis testing, the test statistic (e.g. $F$, $t$, $r$) with confidence intervals, effect sizes, degrees of freedom and $P$ value noted<br>*Give P values as exact values whenever suitable.* |
| ☒ | ☐ | For Bayesian analysis, information on the choice of priors and Markov chain Monte Carlo settings |
| ☒ | ☐ | For hierarchical and complex designs, identification of the appropriate level for tests and full reporting of outcomes |
| ☐ | ☒ | Estimates of effect sizes (e.g. Cohen's $d$, Pearson's $r$), indicating how they were calculated |

*Our web collection on statistics for biologists contains articles on many of the points above.*

## Software and code

Policy information about availability of computer code

| | |
|---|---|
| Data collection | Our optical images of material sections were observed with an inverted microscope (Leica DMI8). Confocal images were collected using a laser confocal microscope (Leica TCS SP8) and 3D reconstruction was conducted using software LAS X. Scanning electron microscopy (SEM) images were collected using Hitachi SU8010. Fourier Transform infrared spectra were collected using a Fourier transform infrared spectrometer (PerkinElmer Spectrum Two). Thermogravimetry curves of mycelium samples were measured with a thermogravimetric analyzer (HENVEN HCT-). Height and DMT modulus images were scanned with an atomic force microscope (Bruker Dimension® Icon™). Small angle X-ray scattering (SAXS) was carried out using a SAXS instrument (Xenocs Xuess3.0). MicroActive AutoPore V 9600 was employed to carry out mercury intrusion tests. X-ray microscopy (ZEISS Xradia 515 Versa) was employed to conduct micro-CT. Ion chromatogram was performed using a ThermoFisher ICS5000 ion chromatograph. A Keithley 2400 source meter unit (SMU) was employed to collect real-time resistance values of mycelium materials. |
| Data analysis | Analysis of all optical and SEM images was performed using Image-Pro Plus 6.0. Atomic force microscope data was analyzed with NanoScope Analysis 1.5 software. Data processing of Micro-CT was conducted using Avizo. |

For manuscripts utilizing custom algorithms or software that are central to the research but not yet described in published literature, software must be made available to editors and reviewers. We strongly encourage code deposition in a community repository (e.g. GitHub). See the Nature Portfolio guidelines for submitting code & software for further information.

# Data

Policy information about availability of data

All manuscripts must include a data availability statement. This statement should provide the following information, where applicable:

- Accession codes, unique identifiers, or web links for publicly available datasets
- A description of any restrictions on data availability
- For clinical datasets or third party data, please ensure that the statement adheres to our policy

All experimental data supporting the findings of the study are available within the article and its Supplementary Information. Source data are provided with this paper. All data are available from the corresponding author upon request. Optimized atomic coordinates and molecular dynamics trajectories are provided as a supplementary data file.

# Research involving human participants, their data, or biological material

Policy information about studies with human participants or human data. See also policy information about sex, gender (identity/presentation), and sexual orientation and race, ethnicity and racism.

| | |
|---|---|
| Reporting on sex and gender | This study did not involve human participants. Therefore, no data related to sex, gender, identity/presentation, sexual orientation were collected or analyzed. |
| Reporting on race, ethnicity, or other socially relevant groupings | This study did not involve human participants. Therefore, no data related to race, ethnicity, or racism were collected or analyzed. |
| Population characteristics | Not applicable in this study. This study did not involve any human participants or human-derived samples. |
| Recruitment | Recruitment is not applicable as there were no human subjects. |
| Ethics oversight | Ethics approval was not required because the study did not involve human participants, human data, or human tissues. |

Note that full information on the approval of the study protocol must also be provided in the manuscript.

# Field-specific reporting

Please select the one below that is the best fit for your research. If you are not sure, read the appropriate sections before making your selection.

☒ Life sciences ☐ Behavioural & social sciences ☐ Ecological, evolutionary & environmental sciences

For a reference copy of the document with all sections, see nature.com/documents/nr-reporting-summary-flat.pdf

# Life sciences study design

All studies must disclose on these points even when the disclosure is negative.

| | |
|---|---|
| Sample size | Due to constraints associated with long-term cultivation and sample homogeneity, all specimens for tensile test were uniformly fabricated into rectangular specimen with a width of 10 mm, a total length of 100 mm, and initial grip separation of 80 mm. Sample thickness varied depending on the fungal species and cultivation duration, n = 5. Thickness measurements, obtained using a vernier caliper, ranged from 0.20 to 0.80 mm. Samples for hygroscopic deformation experiment were 5 × 20, 5 × 25 and 5 × 30 mm, n = 3. Samples for plasticizer migration testing were prepared in diameter of 10 mm, n = 3. Samples for livingness quantifying on PDA medium were normalized in diameter of 5 mm, n = 3. For n = 3: This was applied to experiments with relatively short culture cycles (e.g., 5-10 days of submerged fermentation]) and straightforward sample processing (e.g., direct mycelial biomass collection). In these cases, the intra-group variance was consistently low in our preliminary tests, making three independent biological replicates sufficient to detect major biological trends while minimizing resource waste. This number is the minimal standard for parametric statistical tests (e.g., Student's t-test) in microbiological assays.<br>For n = 5: This was chosen for experiments involving moderate handling complexity or slightly longer incubation periods (e.g., tensile tests of mycelium macerates), where batch-to-batch variability in fungal growth rates is moderately higher. Increasing replicates to 5 enhances the robustness of the mean estimation without overburdening the experimental workflow.<br>Samples were allocated based on experimental group design (e.g., control vs. treatment) using synchronized strain passages. While individual containers were not randomized, all covariates (passage number, seeding density, culture duration, and reagent lots) were strictly controlled and matched across all groups within each independent biological repeat. Since the primary biological variability arises from inter-batch culture differences rather than intra-plate positional effects, our allocation strategy ensured that each independent preparation (each "n") contained the full set of groups. The varying n (3 -10) reflects the logistic feasibility of preparation, but for all cases, covariates were balanced, and results were validated across independently prepared batches. |
| Data exclusions | Mycelium samples treated with 0-5% glycerol were excluded because of brittleness. While the samples treated with 0-5% PVA were too soft and thus excluded. In the course of the experiment, these samples were observed to be exceptionally rigid, rendering them unable to be bent or transformed into dumbbell shapes for further processing. |

| | |
|---|---|
| Replication | Each experiment was carried out at least 3 independent replications and was described in the figure captain with similar results. There were 5 parallel experiments for each group of tensile tests and 3 parallel experiments for each group of humidity response tests. |
| Randomization | To ensure biological safety, we selected mushroom fungi species (macrofungi): standard strain, Phanerochaete chrysosporium BKM-F-1767 (PC), and wild type Trametes versicolor (TV),Coprinus comatus (CC), Pleurotus ostreatus (PO), Agaricus bisporus (AB), as the mycelium source due to their well-documented cultivability. Furthermore, black yeast-like fungi (Cladophialophora sp., CP) was also utilized to obtain Janus mycelium macerate samples. These fungi species were observed with various growth period, membrane thickness and deformation behaviors. |
| Blinding | The investigators were blinded to group allocation during data collection and/or analysis. |

# Reporting for specific materials, systems and methods

We require information from authors about some types of materials, experimental systems and methods used in many studies. Here, indicate whether each material, system or method listed is relevant to your study. If you are not sure if a list item applies to your research, read the appropriate section before selecting a response.

## Materials & experimental systems

| n/a | Involved in the study |
|---|---|
| ☒ | ☐ Antibodies |
| ☐ | ☒ Eukaryotic cell lines |
| ☒ | ☐ Palaeontology and archaeology |
| ☒ | ☐ Animals and other organisms |
| ☒ | ☐ Clinical data |
| ☒ | ☐ Dual use research of concern |
| ☒ | ☐ Plants |

## Methods

| n/a | Involved in the study |
|---|---|
| ☒ | ☐ ChIP-seq |
| ☒ | ☐ Flow cytometry |
| ☒ | ☐ MRI-based neuroimaging |

## Eukaryotic cell lines

Policy information about cell lines and Sex and Gender in Research

| | |
|---|---|
| Cell line source(s) | Trametes versicolor wild type and Phanerochaete chrysosporium BKM-F-1767 were purchased from Shanghai Microbiological Culture Collection Co.,Ltd. Wild type Coprinus comatus (CC), Pleurotus ostreatus (PO), Agaricus bisporus (AB) were purchased from Zhou Yulin Edible Fungus Research institute, Hongshan District, Wuhan. Cladophialophora sp. was separated from wild Trametes versicolor in Harbin Institute of Technology. |
| Authentication | None of the cell lines used were authenticated. |
| Mycoplasma contamination | The cell lines were not tested for mycoplasma contamination. |
| Commonly misidentified lines (See ICLAC register) | Not applicable. The study uses non-animal eukaryotic cells (fungal cells). No commonly misidentified cell lines. |

## Plants

| | |
|---|---|
| Seed stocks | Not applicable in this study. All cell used in this study are derived from fungi. |
| Novel plant genotypes | Not applicable in this study. All cell used in this study are derived from fungi. |
| Authentication | Not applicable in this study. All cell used in this study are derived from fungi. |
