# Supplementary material for: Tailored living mycelium macerate for reproducible flexible actuators
Source: Nat Commun. 2026 Aug 12;17:9682. doi: 10.1038/s41467-026-76623-z (PMC13558639; doi:10.1038/s41467-026-76623-z)
Supplement: Supplementary file 3 [file 41467_2026_76623_MOESM8_ESM.pdf]

nature portfolio

Peer Review File

Tailored Living Mycelium Macerate for Reproducible Flexible Actuators

Corresponding Author: Professor Xin Huang

This file contains all reviewer reports in order by version, followed by all author rebuttals in order by version.

Version 0:

Reviewer comments:

Reviewer #1

(Remarks to the Author)
The manuscript describes an interesting type of actuators that incorporate living mycelium materials, which provides good performance including mechanical properties and programmable responsiveness. This was done by treating Mycelium Macerate with a PVA and glycerol network, which overcomes the brittleness of dried biomass. Various investigations were conducted, including micro-CT, SAXS, MD simulations. Applications as sensors were demonstrated. The key mechanism behinds the hygroscopic deformation was the “Janus” structure of the mycelium. Lastly, the author demonstrated the material’s “dormant living” nature, which I found particularly interesting. The material can be stored and regenerated after one year with self-healing capabilities and recyclability.
The manuscript is well-written, the data is solid, and the findings are of broad interest to the fields. I support the publication of the paper after addressing some of the minor issues listed as following,
1. In Fig. 4 and 5 (and Fig. S45-S51), the authors use MD simulations to explain drives for deformation. The manuscript mentions simplifying the polymer chains (chitin, glucan, etc.) into fragments for calculation. While simplification is necessary for computational feasibility, the fungal cell wall is a complex, cross-linked matrix. Could the authors discuss or provide justification on how well these simplified fragments represent the actual physical entanglement and steric hindrance present in the real system?
2. Figs. S71–S77 presents an interesting finding: different fungal species exhibit different, even opposite, deformation directions. This is a significant result that supports the "tailorable" claim in the title but is discussed somewhat briefly in the main text. I suggest expanding the Discussion section to elaborate on the biological or structural reasons for these differences. Is it driven by inverted density gradients of aerial vs. nutritive hyphae in different species?
3. In title, “reproducible” was used. How do materials made from different batches of fungal differ in actuation performance and mechanical properties?
4. For flexibility and moisture absorption, the material relies on PVA and glycerol which are both water-soluble/hydrophilic, and the actuation mechanism relies on high humidity. Is there a risk of glycerol leaching or migration over repeated cycles of high-humidity exposure or long-term storage? Did the authors observe any degradation in mechanical properties or actuation amplitude over cycling tests?
5. What is the more detailed mechanism of regeneration? Is it primarily driven by the re-activation of the vegetative hyphae or the germination of spores embedded in the matrix? Is there a way to quantify the “livingness” of the material?
6. Fig. 2d, the unit of toughness should be “MJ/m3”. In the paper, values like “66.13~349.42% elongation” and “1.28~18.04 MJ/m-3” were reported. The number of significant figures should be reduced, as the current level of precision appears to exceed the practical accuracy of the experimental measurements.

Reviewer #2

(Remarks to the Author)
The authors present a modified mycelium material that exhibits directed motion due to a two-layer structure that responds differently to changes in humidity. Their work is accompanied by a comprehensive analysis of the material structure, the interaction of PVA and glycerin with the material and its various layers, and several demonstrations of its use in sensors and actuators. The idea is interesting, but the article lacks scientific novelty, appropriate statistics and objective analysis, as well as adequate demonstrations. Furthermore, and most importantly, the state of the manuscript does not allow for proper

review, as the language/writing style is chaotic, characterized by missing words, exaggerated language, unnecessarily complex/complicated words and sentence structures, and unsatisfactory reasoning in the conclusions. The latter is mainly due to the large number of different experiments and the resulting findings as well as missing statistics.

Another issue is the lack of statistical analysis with an appropriate amount of samples for certain experiments. In order to make reliable statements, it must first be demonstrated that the material can be produced with sufficiently low deviations in terms of mechanical properties and structural composition. The growth of mycelium materials can exhibit quite large deviations in this respect, especially within different batches that are cultivated at different times. Thorough investigations include analyzing the variations within the same material, within the same batch, and those produced at different times. While this may seem quite extensive, it is necessary due to the complexity of the materials and the fact that this is not a "simple synthesis" but an organism that grows on its own. Only then are arguments based on just one or a few samples viable.

In my opinion, this article does not meet the high standards of this journal and needs to be completely revised before another submission to ensure compliance with scientific standards in both writing and experiments.

I suggest that the authors revise the entire manuscript in relation to two aspects:
1. Use appropriate language, avoid exaggerations, and provide arguments for each conclusion and claim.
2. Reduce the number of experiments and include only those that are informative for this work and provide reliable results. Do not include poorly designed experiments, such as the use of the humidifier when accurate relative humidity values are required, the sample preparation in Fig. S19a (separating the layers with a scalpel cannot be done reliably), and the entire setup in Fig. 2c (misleading description and conclusion).

While it is not feasible to address all issues and discrepancies, many of which are likely due to language barriers, I will outline the key points below that the authors should consider:

1. The claim that low elongation rates are a limitation of mycelium materials is insufficient. As with all other materials, the mechanical property requirements are application-specific, and low elongation rates are no disadvantage in many instances.
2. Terms such as "tailored" and "programmable" suggest that the material can be adapted for specific bending angles in response to specific environmental conditions. This is not demonstrated, and I strongly doubt that it is possible given the likely varations in mechanical properties.
3. There is hardly a common thread throughout the entire manuscript, especially given the presentation of all the experiments, and references to figures/panels are not in the correct order.
4. Each experiment requires descriptions with all relevant information so that others can replicate it, including sample geometries, statistics, environmental conditions, etc.
5. Experiments involving humidity appear to be rather inaccurate, as the humidified air is only a small jet from the device. I cannot imagine how a sensor nearby can measure the exact value here. For all experiments requiring an accurate value for relative humidity, use a chamber.
6. The contour maps in Fig. 2 are very detailed, but are based on only a few measurements in Fig. S9. How is all the missing information generated, or did the authors forget to include a data set? This is particularly interesting because the "best" result is not included in the measurements.
7. Demonstrations such as those in Fig. 2c are not meaningful in terms of the weight mentioned. The result can be easily manipulated because it is a poor experimental design. In general, the demonstrations are rather superficial and in the case of the sensors do not seem feasible. I suggest focusing on the bending for actuation and expanding the characterization (repeatability, performance over time, ...) and design here, as this has the greatest potential.
8. Layer separation in general seems hard to do without damage. Address why your methods are reliable in this regard as it is not obvious.

Reviewer #3

(Remarks to the Author)
Nie and co-workers report the development of a mechanically robust, humidity-responsive, and biologically viable "living mycelium macerate" based on liquid-state fermented fungal membranes engineered through PVA–glycerol maceration. By exploiting the intrinsic Janus architecture of aerial and nutritive hyphal layers, the authors achieve asymmetric hygromechanical behavior that enables programmable actuation and humidity-to-electrical signal transduction. The work further demonstrates long-term dormancy, regeneration, and inheritance of structural and functional properties across multiple growth generations.

The study integrates multiscale characterization (SEM, AFM, micro-CT, SAXS, MIP), computational modeling (DFT and MD), mechanical testing, and in vitro assays to establish a comprehensive structure–property–function relationship. The reported elongation and toughness values are among the highest for flexible mycelium-based materials, and the concept of a regenerative, inheritable "dormant living actuator" is novel and conceptually impactful.

Overall, the manuscript is ambitious, interdisciplinary, and largely well executed. Therefore, I recommend the publication of this work in Nature Communications, provided the following comments are addressed:

1. The main concern is about the reproducibility of mechanical properties. The authors have repeatedly claimed "record-

breaking" mechanical performance among flexible mycelium materials. However, limited information about the mechanical tests have been provided, especially the sample sizes (n) and error bars that should be consistently emphasized in all mechanical and hygroscopic performance plots. The reproducibility of the performance is crucial for this kind of un-conventional living materials.

2. Although the development is based on living materials, PVA, a non-degradable, non-biobased polymer has been used as the main network enhancer to improve the mechanical properties. The authors may clarify the end-of-life scenario for PVA-containing macerates in real-world deployment, and consider potential bio-based or biodegradable alternatives to PVA and their expected impact on mechanical and hygroscopic performance. In addition, it is not surprising to see the introduction of strong polymer chains bringing hydrogen bonding can improve the mechanical properties of the pristine materials.

3. The demonstrated application for actuation and sensing operate on timescales of minutes, which limits applicability for many real-time sensing or robotic control scenarios. Any comments on this?

Version 1:

Reviewer comments:

Reviewer #1

(Remarks to the Author)
My previous concerns have been fully resolved by the authors. I agree to the acceptance of the paper.

Reviewer #2

(Remarks to the Author)
The authors have, in my opinion, addressed all the comments raised by the reviewers and fully sustained all the claims in this manuscript. They added and revised several experiments to now ensure reliable and reproducible results. Furthermore, thanks to the new additions to the text, the manuscript reads now better and it will be easier for the reader to follow the experiments.

Reviewer #3

(Remarks to the Author)
The authors have made reasonable effort to address my comments. I have no further quesitons and recommend the publication of this work.

Open Access This Peer Review File is licensed under a Creative Commons Attribution 4.0 International License, which permits use, sharing, adaptation, distribution and reproduction in any medium or format, as long as you give appropriate credit to the original author(s) and the source, provide a link to the Creative Commons license, and indicate if changes were made.

In cases where reviewers are anonymous, credit should be given to 'Anonymous Referee' and the source.
The images or other third party material in this Peer Review File are included in the article's Creative Commons license, unless indicated otherwise in a credit line to the material. If material is not included in the article's Creative Commons license and your intended use is not permitted by statutory regulation or exceeds the permitted use, you will need to obtain permission directly from the copyright holder.
To view a copy of this license, visit https://creativecommons.org/licenses/by/4.0/

Response to the reviewers' comments (Reviewer#1)

Comments to the Author:

The manuscript describes an interesting type of actuators that incorporate living mycelium materials, which provides good performance including mechanical properties and programmable responsiveness. This was done by treating Mycelium Macerate with a PVA and glycerol network, which overcomes the brittleness of dried biomass. Various investigations were conducted, including micro-CT, SAXS, MD simulations. Applications as sensors were demonstrated. The key mechanism behinds the hygroscopic deformation was the "Janus" structure of the mycelium. Lastly, the author demonstrated the material's "dormant living" nature, which I found particularly interesting. The material can be stored and regenerated after one year with self-healing capabilities and recyclability. The manuscript is well-written, the data is solid, and the findings are of broad interest to the fields. I support the publication of the paper after addressing some of the minor issues listed as following,

Response: We thank Reviewer#1 for the careful review and constructive comments on our manuscript. We greatly appreciate the time and effort you have dedicated to providing insightful feedback, which has helped us improve the quality of our work.

We have carefully considered all the comments and have revised the manuscript accordingly. Below, we respond to each point raised by the reviewer point-by-point. All changes in the manuscript have been highlighted in yellow for your convenience.

1. In Fig. 4 and 5 (and Fig. S45-S51), the authors use MD simulations to explain drives for deformation. The manuscript mentions simplifying the polymer chains (chitin, glucan, etc.) into fragments for calculation. While simplification is necessary for computational feasibility, the fungal cell wall is a complex, cross-linked matrix. Could the authors discuss or provide justification on how well these simplified fragments represent the actual physical entanglement and steric hindrance present in the real system?

Response: Thank you for your professional and thoughtful comment. Your concern regarding the simplified model of fungal cell wall is an excellent and highly pertinent question that gets to the heart of a major challenge in MD simulations of biological structure: balancing detail with computational capacity. In the fungal cell wall, chitin, glucan and other polysaccharide are distributed in hierarchical architecture. Our model is not intended as a complete replica but rather a defined, minimal model designed to study the interaction between biomacromolecules and water on average, which contributes to revealing the physical principle of hygroscopic deformation. While large-scale topological entanglements are undoubtedly present in vivo, capturing them explicitly would require simulations at a much larger spatiotemporal scale that are currently computationally prohibitive.

Modification: According to your professional suggestions and our experiment results, we have added an explanation regarding the MD simulations in the main text, page 10, lines 227-280.

"…It is important to note that this model represents a simplified view of the cell wall. The calculated interaction energies reflect the local chemical environment, but do not account for the long-range physical constraints imposed by the global network architecture."

2. Figs. S71–S77 presents an interesting finding: different fungal species exhibit different, even opposite, deformation directions. This is a significant result that supports the "tailorable" claim in the title but is discussed somewhat briefly in the main text. I suggest expanding the Discussion section to elaborate on the biological or structural reasons for these differences. Is it driven by inverted density gradients of aerial vs. nutritive hyphae in different species?

Response: Thank you for your professional suggestions. And we agree with the reviewers' assumption that mycelium materials formed by different fungal species varied in density gradients between aerial (layer A) and nutritive (layer B) hyphae, which resulted in various hygroscopic deformation behaviors. As suggested, we have expanded the Discussion section to elaborate on the biological and structural reasons for these differences. Furthermore, to verify this assumption, we have added further characterization and discussion on the oppositely deformed PO@P-G macerate. Combined with our new experiment results of repeating hygroscopic deformation in the humidity chamber suggested by the reviewer#2, mercury intrusion porosimetry (MIP) test for separated layer A and B of PO@P-G macerate was carried out.

Interestingly, we found that the Janus density gradient orientation is actually the same between the two species, TV and PO, both with higher density on layer B and lower on layer A. However, specie PO exhibits a significantly larger interlayer density difference than specie TV. This result demonstrates that the density gradient is not the sole factor determining the direction of hygroscopic deformation. Instead, we now propose that the magnitude of the density difference may lead to qualitatively different mechanical responses, for example, different swelling/contraction ratios. Furthermore, the finding that backbone fractal dimension of layer B was higher than that of layer A in specie PO offered a new explanation, which evidences reversed relationship in fractal dimension between species TV (Fig. S31) and PO.

Modification: According to your professional suggestions, we have added the following sentences in the Discussion section (page 16, lines 471-474) to conclude these results.

"…The opposite deformation direction across species is a key support for the 'tailorable' claim, yet previously underexplored. We interpret these differences primarily as a result of species-specific Janus structural asymmetry (Fig. S79 and 80, Supplementary text of discussion)."

Cumulative intrusion and extrusion curves of layer A and B, as well as pore size distribution curves were added as new Fig. S79 in the revised manuscript. In addition, we compared pore structure parameters of layer A and B in new Fig. S80 and analyzed MIP data in Supplementary Information, Supplementary text of discussion (page 70, lines 820-836).

"…MIP testing was performed to explore the relationship between hygroscopic deformation and density gradients in different fungal species. Separated layer A and B of PO@P-G macerate showed remarkable differences in mercury intrusion and extrusion processes (new Fig. S79a, b), indicating that layer A of PO macerate displayed a much higher porosity than layer B. Pore diameter distribution (new Fig. S79c, d) showcased an inversed result that layer A compared with specie TV. We quantified pore structure parameters using AutoPore V 9600 software and compared these parameters in radar chart (new Fig. S80). With a much less porosity of 11.409%, layer B of specie PO showed much more bulk density and skeleton density than layer B. Density results from MIP showed that both species TV and PO share the same gradient orientation, density (layer A) < density (layer B). The opposite deformation remained unexplained. Notably, a reverse difference in backbone fractal dimension of specie PO, (a backbone fractal dimension 2.963 for layer B, 2.667 for layer A in specie PO; while 2.940 for layer A, 2.758 for layer B in specie TV), brought about a speculation that fractal dimension asymmetry programed deformation behaviors in biological Janus structures. In the case of similar percolation fractal dimensions, a higher backbone fractal dimension possibly

correlates with enhanced hygroscopic responsiveness. Thus, even when density gradients are identical, the layer with higher backbone fractal dimension becomes the dominant active layer under external stimuli, dictating the bending direction. In specie TV, the layer A dominates; while in specie PO, layer B takes over resulting in opposite curvatures."

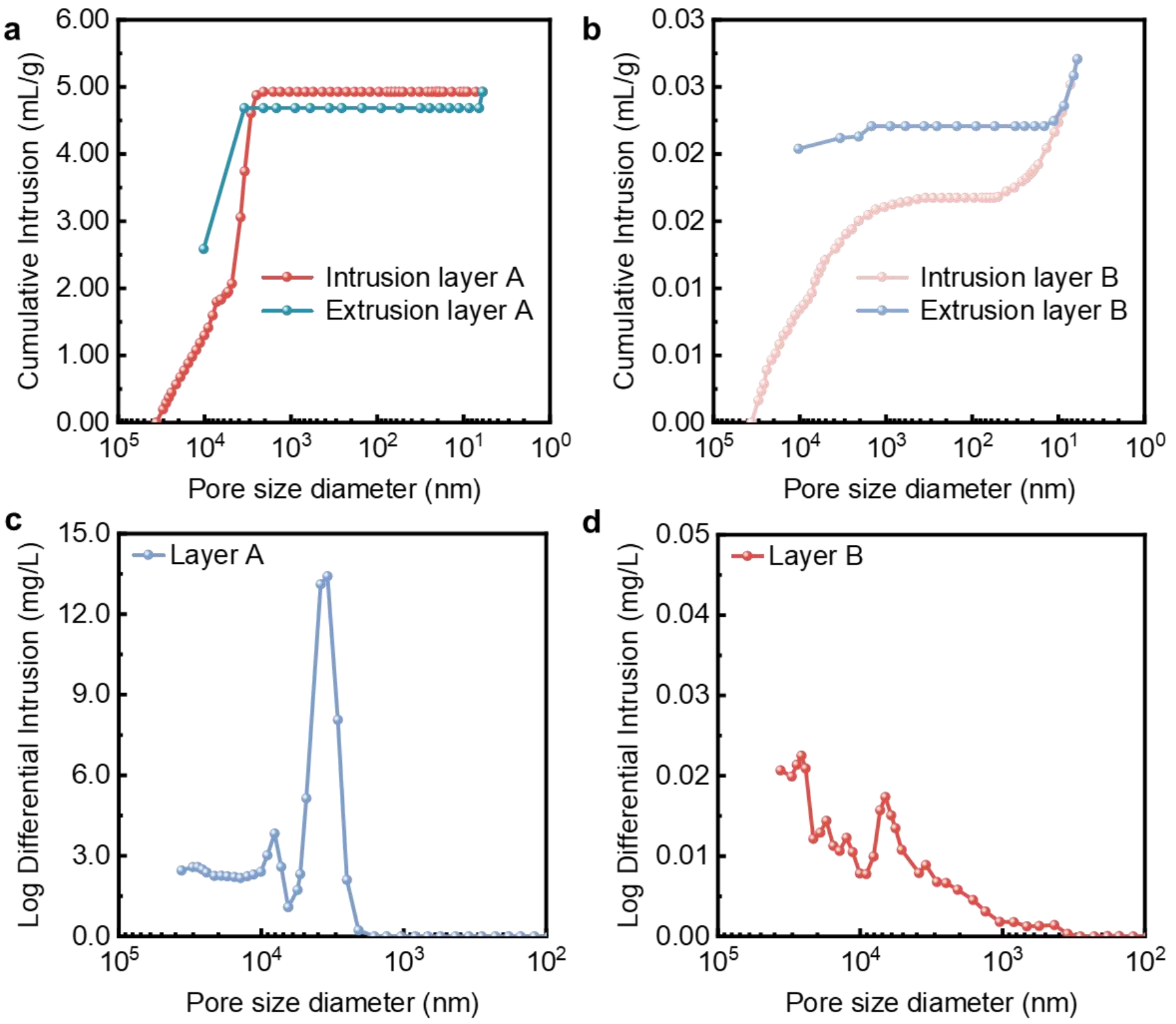

New Fig. S79 Mercury intrusion results of PO@P-G macerate at dry state. **a**, **b**, Cumulative intrusion and extrusion versus pore diameter for layer A (**a**) and B (**b**). c, d, Mercury intrusion pore size distribution curves of layer A (**c**) and B (**d**).

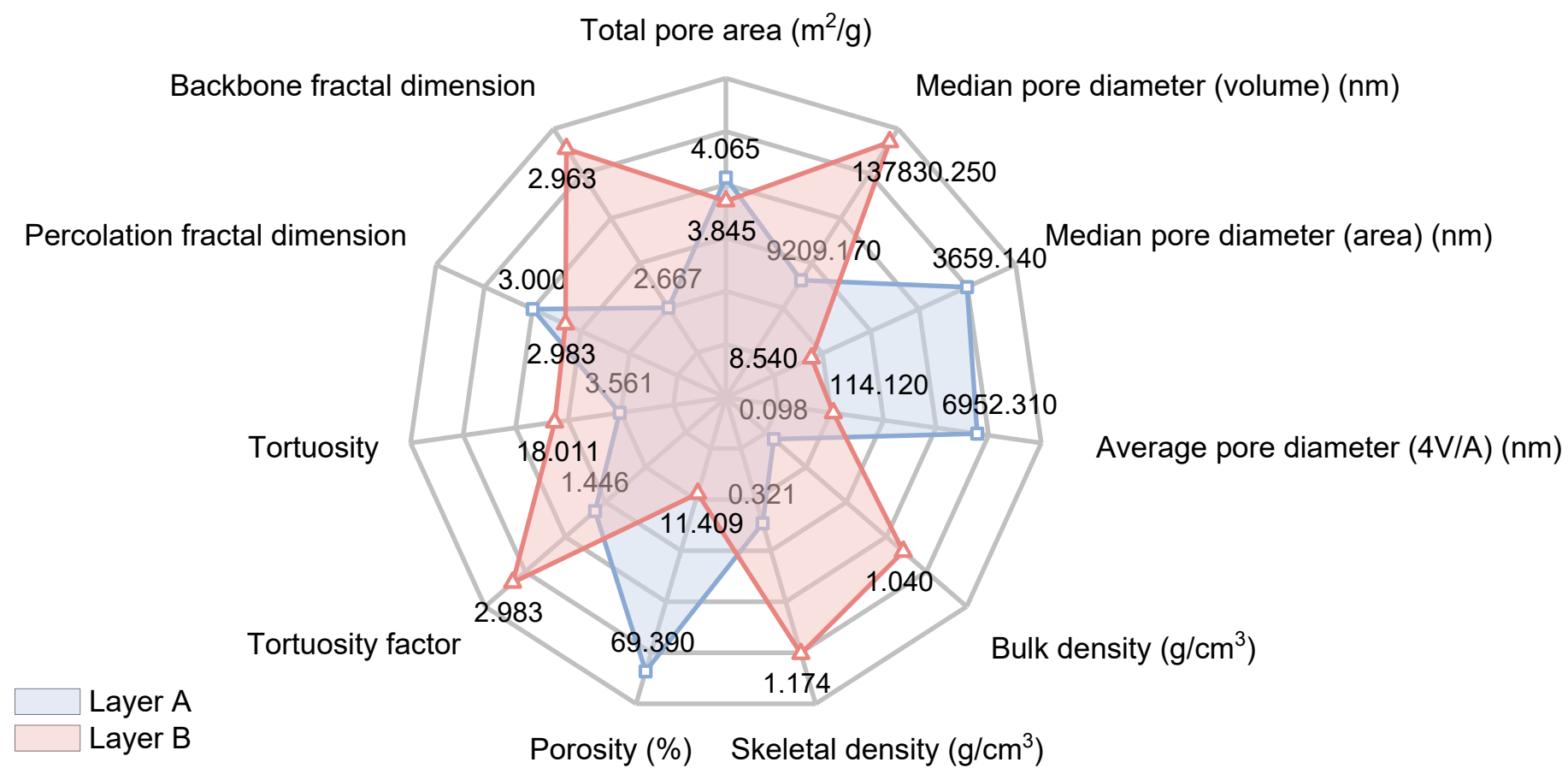

New Fig. S80 Comparison of pore structure parameters between layer A and B of PO@P-G macerate.

3. In title, "reproducible" was used. How do materials made from different batches of fungal differ in actuation performance and mechanical properties?

Response: Many thanks for the instructive comment. Our research mentioned "reproducible" derived from livingness of mycelium materials. Specifically, the mycelium macerate could be recycled as "seed" to reproduce the next generation of mycelium membrane, of which process was demonstrated in Fig. 5d. We agree that simply using the word "reproducible" in the title just

considering the regeneration of fungi. It is insufficient to lack demonstration on batch-to-batch consistency.

In fact, we have prepared materials from five regenerated batches of TV and characterized their mechanical properties in our original manuscript, as shown in Fig. 5d. There is a comparison of the mechanical properties of different generations of materials. The results showed that as the batches of living materials increasing, the mechanical property underwent a self-renewal process from high strength to high strain. Accordingly, we have kept the word "reproducible" in the title but clarified the batch-to-batch performance in the text.

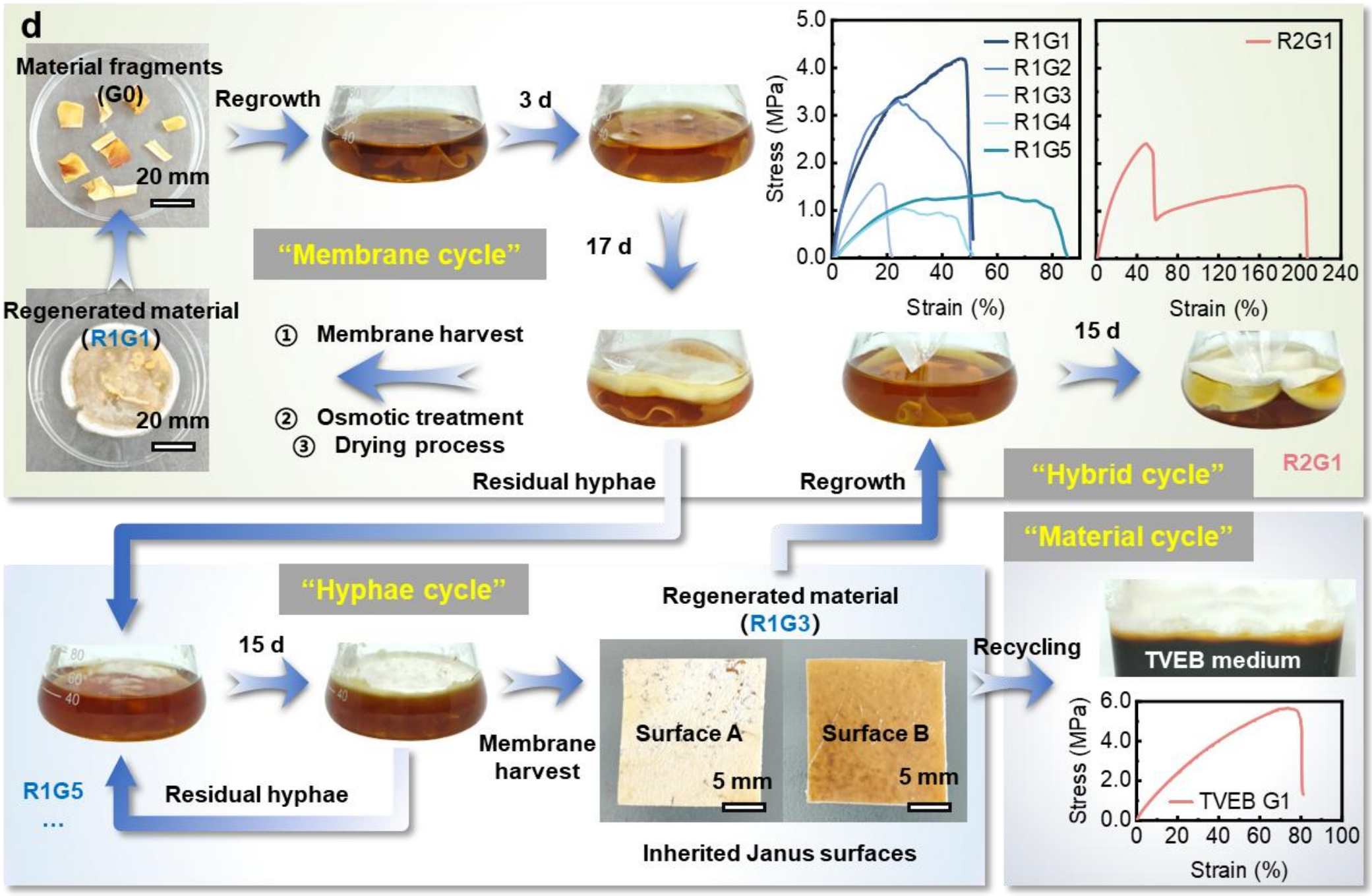

Fig.5d Four regeneration pathways of mycelium macerates. Membrane cycle: regrowth process of TV@P-G fragment in MEB liquid medium. New mycelium membrane regrew and matured on gas-liquid interface. Hyphae cycle: residual hyphae regrow and form mycelium membrane repeatedly. Hybrid cycle: regeneration from regrew mycelium macerates through membrane cycle and hyphae cycle. Material cycle: recycling mycelium macerates into culture medium to generate new mycelium membrane. Insets: stress-strain curves of regrowth mycelium macerates through different regeneration pathways.

4. For flexibility and moisture absorption, the material relies on PVA and glycerol which are both water-soluble/hydrophilic, and the actuation mechanism relies on high humidity. Is there a risk of glycerol leaching or migration over repeated cycles of high-humidity exposure or long-term storage? Did the authors observe any degradation in mechanical properties or actuation amplitude over cycling tests?

Response: We sincerely thank you for your careful check and comments. First, we compared glycerol content of new samples and those have been stored for 1 year, which is displayed as new Fig. S85. There was almost no glycerol leaching for samples stored at room temperature, 25% RH. Moreover, after drying process, mycelium material samples stored at 8°C, 60% RH also showed a close glycerol content compared with new samples. However, since we had not stored the samples at high humidity conditions (> 80% RH) in the past year, the results of long-term storage of samples at high humidity are not available. We speculate that long-term storage under high humidity

conditions (> 80% RH) would lead to plasticizers leaching.

Next, the leaching of glycerol when exposure to cyclic humidification and dehumidification was quantified in new Fig S86. The small molecule plasticizer glycerol (molecular weight of 92 g·mol^{-1}) is prone to migration and moisture absorption, which is a major drawback in the application of biobased materials with plastic modification. We followed d'Errico's method in [Polyglyceryl-4 caprate and polyglycerol-3 provide low mobility and enhance ductility in *Schizophyllum commune* mycelium materials. *Colloids and Surfaces A: Physicochemical and Engineering Aspects* **719**, (2025).] to determine the migration percentage of glycerol at cyclic high-humidity (85%) exposure. As the reviewer concerned, high humidity promoted migration of water-soluble glycerol. Besides paper, we also set PVA as adsorbent material to confirm that diffusion of plasticizer molecules in PVA matrix was restricted, compared to biomacromolecule-based (polysaccharide) matrix. In order to reduce the loss of plasticizer in the application scenario, hydrophobic materials could be used to contact these plasticizer-treated macerates directly. Pressed by polytetrafluoroethylene (PTFE) plates, TV@P-G lost 6.76% of total mass in 6-day exposed to 25℃, 85% RH ~ 35 %RH cycles, equivalent to 15.57% of initial glycerol content. In brief, using hydrophobic materials to contact with mycelium macerates and selecting higher molecular weight plasticizers is expected to reduce the leaching of plasticizers in a high-humidity environment. With a larger molecular size, polyglycerol could replace glycerol as the plasticizer of mycelium materials in our future study, potentially reducing the migration rate of plasticizer in macerates. We supposed that polyglycerol would enhance migration resistance, which is further related to molecular polarity and physical entanglement.

On the other hand, we supplemented three consecutive cycles of humidity response experiments using a humidity chamber (85% RH), as well as control groups whose humidity response bending recovered after re-drying the samples exposed to a dry environment (25% RH). After undergoing a series of continuous humidification-dehumidification cycles, the maximum bending angle of mycelium macerate decreased to 50%. And after 1-hour and 24-hour drying process, the maximum bending angle can recover to 59% and 83%, respectively.

Modification: We added the comparison result on glycerol percentage, as well as PVA and biomass percentage between newly fabricated and 1-year stored TV@P-G macerates as new Fig. S85.

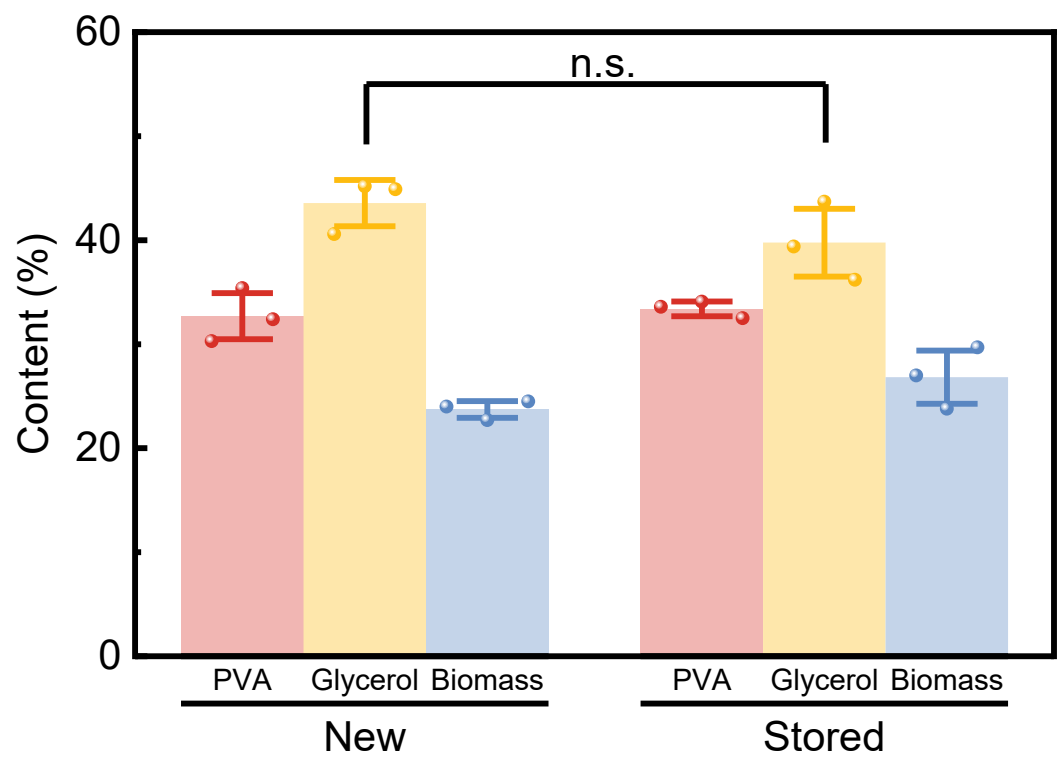

New Fig. S85 Percentage composition of PVA, glycerol and biomass in newly fabricated and 1-year stored TV@P-G macerates, n = 3. The significance of differences between the two groups was assessed using Student's t-test. For t-tests: Cohen's d was calculated as ($mean_1$ - $mean_2$) / pooled standard deviation. Difference in glycerol content: p = 0.222, Cohen' d = 0.45.

Plasticizer migration experiment was carried out, which method and results were summarized in Supplementary Information (page 17 lines 468-475), and new Fig. S86, respectively.

"Plasticizer migration measurements

Plasticizer diffusion was measured following ISO 177:2016 with modifications. Samples were prepared with diameter of 10 mm. For each cyclic humidity condition (85% RH for 12 h to 35% RH for 12 h and 35% RH for 24 h) at room temperature, three parallel experiments were conducted. Each sample was placed between two layers of 2-mm-thick contact materials and clamped firmly: filter paper, PVA sheets, or polytetrafluoroethylene (PTFE) sheets. The PVA sheets were prepared by pouring and drying with 10%PVA solution. Mass loss was measured each 24 hours by weighing the sample. Three independent replicates were performed for each combination of humidity and contact material."

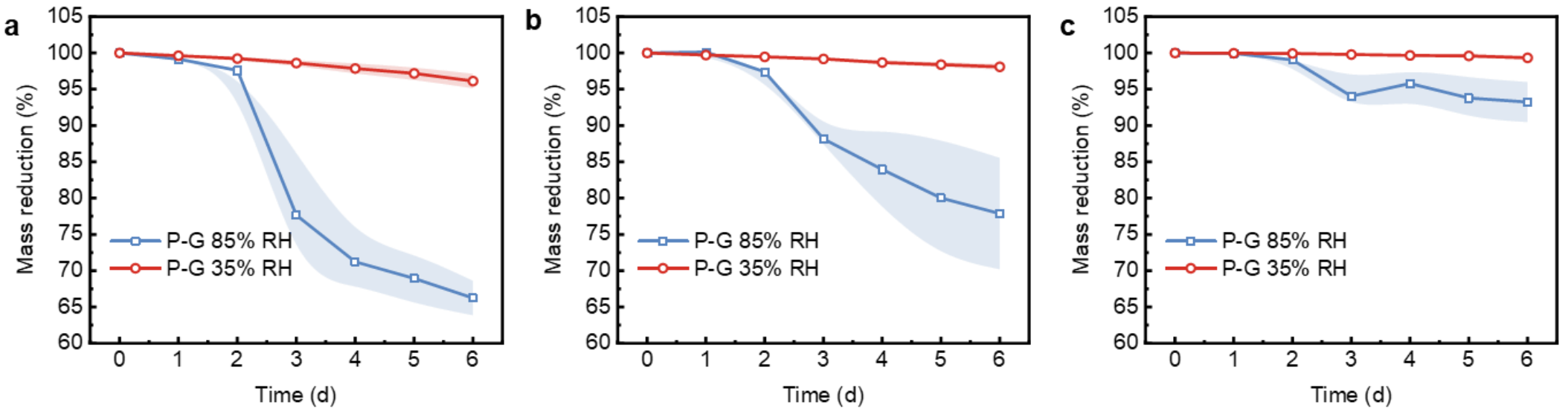

New Fig. S86 Mass reduction curves of TV@P-G pressed by filter paper (**a**) PVA (**b**) and PTFE (**c**) exposed to 25°C, 35% and 85% RH, attributable to plasticizers migration, n = 3. Error bars (mean ± SD) were connected using B-spline basis function and visualized as a shadow effect around the data points.

Furthermore, to verify the cyclic stability of hygroscopic deformation, we conducted repeated actuation and recovery experiments, of which results were added in new Fig. S25.

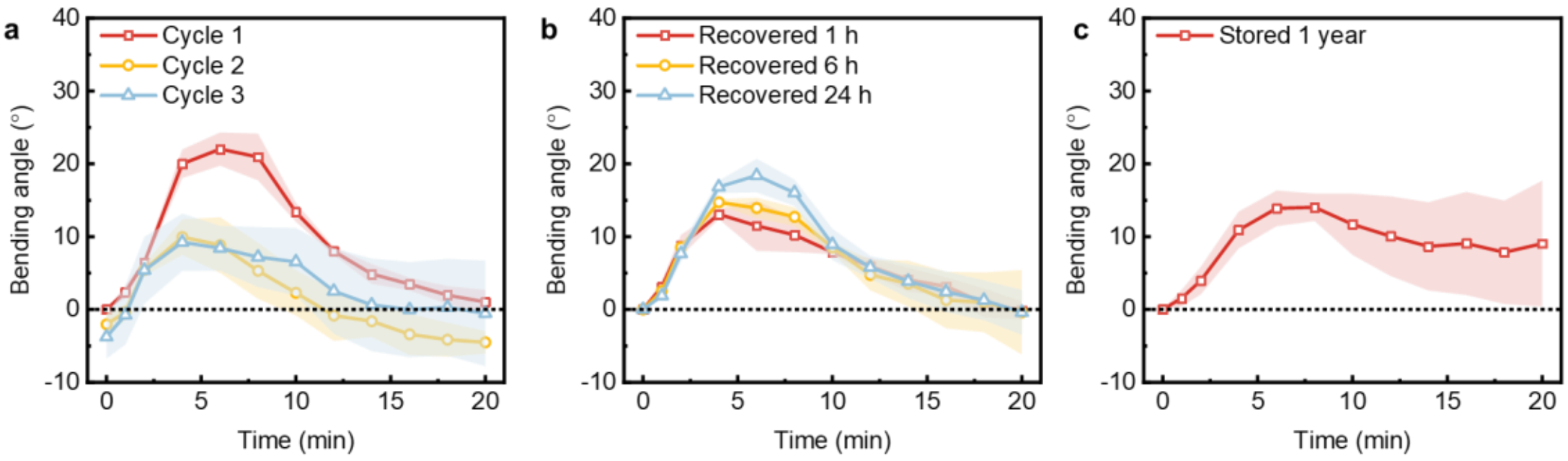

New Fig. S25 Repeatability and over-time performance of hygroscopic deformation. **a**, **b**, Cyclic bending angle changes of Janus TV@P-G (10%PVA+10%Gly) specimen (5 × 25 mm) in single fixed humidity response experiments, n = 3. Humidity was controlled with humidity chamber. Cycle 1 to 3 were continuous processes (a), while the "recovered cycles" were defined as the next cycle after 1-hour, 6-hour and 24-hour drying treatment to samples (b). **c**, Bending angle changes of Janus TV@P-G (10%PVA+10%Gly) specimen stored for 1 year (8°C, 60% RH), n = 3. Humidity was controlled with humidity chamber. Error bars (mean ± SD) were connected and visualized as a shadow effect around the data points.

5. What is the more detailed mechanism of regeneration? Is it primarily driven by the re-activation of the vegetative hyphae or the germination of spores embedded in the matrix? Is there a way to quantify the "livingness" of the material?

Response: We sincerely thank you for your professional comments. We have explained and supplemented the hyphae and chlamydospore might be both revival agent for mycelium macerate regeneration. Given that the fungi species *Trametes versicolor* (TV) we cultivated could generate occasional chlamydospores after membrane formation, we collected TV chlamydospores (new Fig. S70a) to verify their livingness in desiccation. Similar with mycelium macerate material, TV chlamydospores were dispersed in 10% PVA and 10% glycerol solution and embedded PVA-glycerol membrane through drying process. Undergoing 1-month storage (8°C, 60% RH), PVA-glycerol membrane encapsulating chlamydospores was transported onto potato dextrose agar (PDA) medium. Shown as new Fig. S70b, regrowth of hyphae occurred after 3-day cultivation, indicating that spores (chlamydospores) played a significant role in mycelium macerate regeneration. However, it was tough to obtain pure hyphae without spores, due to the connection between spores and hyphae. Inspired by Elsacker's work (*Advanced Functional Materials, 2023,* **33**, 2301875), we chose *Pleurotus ostreatus* (PO) as the control group, because this species rarely generates chlamydospores. It has been proven that PO cannot even be regenerated after short-term dry storage shown in new Fig. S70c. Therefore, we propose that spores play a major role in regeneration of mycelium materials. Furthermore, we have also attempted to quantify the "livingness" of mycelium material. In new Fig. S70d, mycelium membrane or macerate were cut into a normalized size, with a diameter of 5 mm. Average radial growth rate on PDA medium at 25°C, 85%RH could reflect the livingness of mycelium material. We further explore the influence of cyclic generation and process on macerate livingness. Long periods of dormancy or subculture will reduce the livingness of mycelium materials.

Modification: According to your professional question, a clarification of "livingness" was added into Section, *Organism-like sustainability derived from livingness*, the revised main text on pages 13-14, lines 409-412 as follows. To quantify the livingness of our live materials, we evaluated it by the growth rate of material samples in the same diameter on the surface of solid medium, meaning the average growth radius of the same time. Radial growth curves were plotted as new Fig. S70e, where growth rate of dry macerate was slower than fresh mycelium membrane, due to the regeneration period of dormant spores. Additionally, livingness of regenerated macerate was also compared.

"…This regeneration process was mainly achieved by dormant spores, and the livingness of mycelium macerates could be quantified and compared through the radial growth rate (Fig. S70). To maintain the livingness of spores in macerates, the dry storage period should be limited within 1 year."

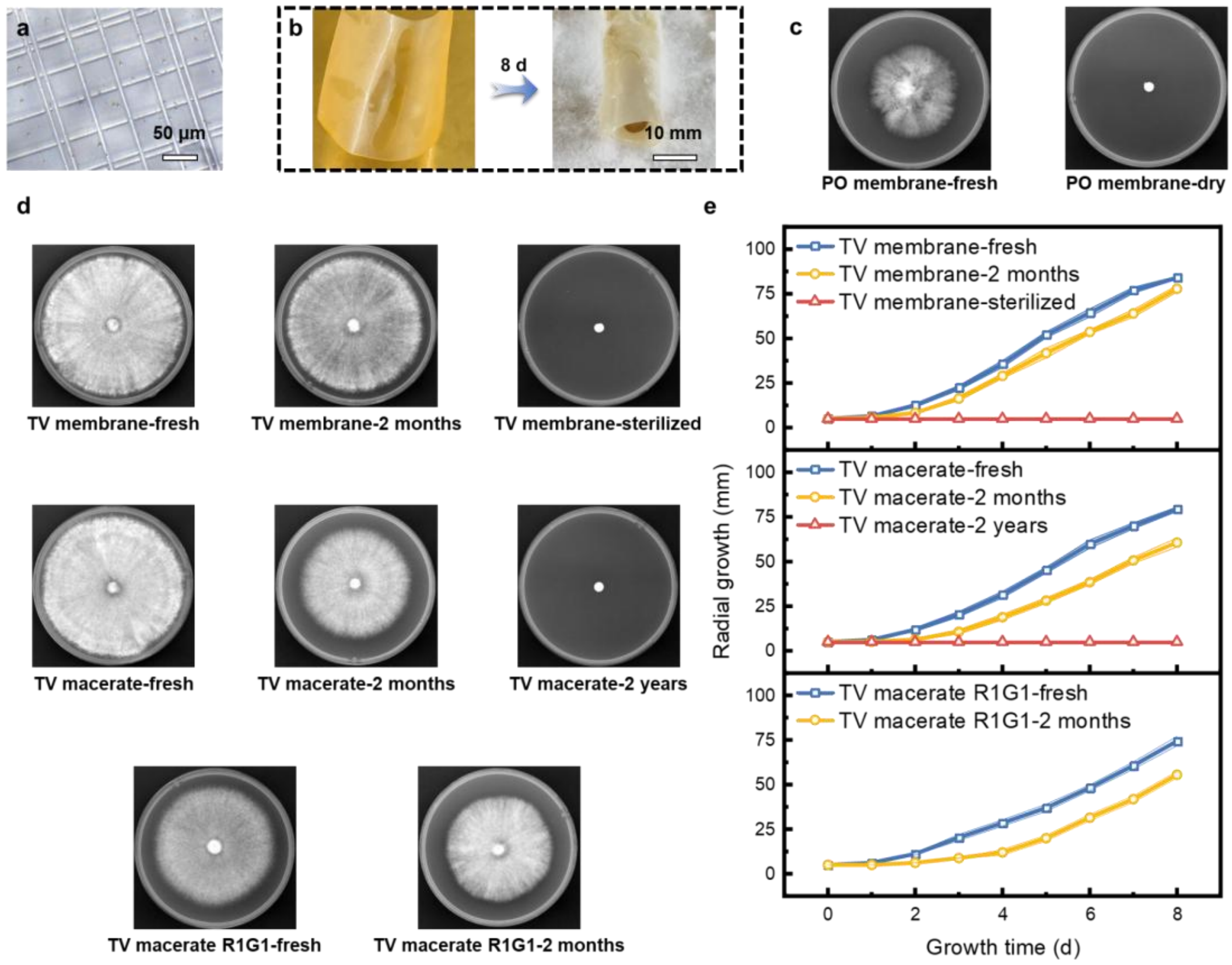

New Fig. S70 a, Optical images of TV spores (chlamydospores) on a hemacytometer. **b**, Regeneration of spores embedded in 10%PVA+10%Gly membrane (3×3 cm), which grew on PDA medium at 28 °C, 85% RH for 8 d. **c**, Optical images of fresh and 1-month dry storage (25 °C, 20% RH) PO mycelium membrane (d = 5 mm) regeneration, which grew on PDA medium at 28 °C, 85% RH for 8 d. **d**, Optical images of TV mycelium material (d = 5 mm) regeneration, which grew on PDA medium at 28 °C, 85% RH for 8 d. **e**, Radial growth curves of mycelium materials (d = 5 mm) on PDA medium at 28 °C, 85% RH for 8 d, n = 3. Error bars (mean ± SD) were connected and visualized as a shadow effect around the data points.

6. Fig. 2d, the unit of toughness should be "MJ/m3". In the paper, values like "66.13~349.42% elongation" and "1.28~18.04 MJ/m3" were reported. The number of significant figures should be reduced, as the current level of precision appears to exceed the practical accuracy of the experimental measurements.

Response: We thank the reviewer for this important observation. We fully agree that the original manuscript reported an excessive number of significant figures. In the revised manuscript, we have evaluated all reported numerical data based on the following three criteria:

1) ASTM D882-18: Standard test method for tensile properties of thin plastic sheeting, which specifies the recommended reporting precision for elongation (± 1%).

2) Instrument sensor accuracy: The force sensor accuracy is ± 0.5% of the full scale (± 0.1 N for 20 N and ± 0.5 N for 100 N), and the displacement sensor accuracy is ± 0.1 mm.

3) Significant figure rules: For multiplication/division (e.g., tensile strength = load / cross-sectional area), the result is reported to the least number of significant figures among the measured quantities.

Modification: The modified accuracy was summarized in new Table S1. Following this systematic review, we have reduced the significant figures in all affected tables (Table S2 and S3), figures (Fig. S11), and the corresponding text. Changes are highlighted in the revised manuscript.

New Table S1 Precision limitations for each measured parameter

Parameter	Accuracy	Standard
Width, length	± 0.01 mm	Accuracy of vernier caliper
Tensile strength	± 0.1 MPa	Significant figure rules
Young's modulus	± 0.1 MPa	Significant figure rules
Elongation rate	± 1%	ASTM D882-18
Toughness	± 0.1 MJ/m^3	Significant figure rules
Bending angle	± 0.2°	Image pixel resolution

Response to the reviewers' comments (Reviewer#2)

Comments to the Author:

The authors present a modified mycelium material that exhibits directed motion due to a two-layer structure that responds differently to changes in humidity. Their work is accompanied by a comprehensive analysis of the material structure, the interaction of PVA and glycerin with the material and its various layers, and several demonstrations of its use in sensors and actuators. The idea is interesting, but the article lacks scientific novelty, appropriate statistics and objective analysis, as well as adequate demonstrations. Furthermore, and most importantly, the state of the manuscript does not allow for proper review, as the language/writing style is chaotic, characterized by missing words, exaggerated language, unnecessarily complex/complicated words and sentence structures, and unsatisfactory reasoning in the conclusions. The latter is mainly due to the large number of different experiments and the resulting findings as well as missing statistics.

Another issue is the lack of statistical analysis with an appropriate amount of samples for certain experiments. In order to make reliable statements, it must first be demonstrated that the material can be produced with sufficiently low deviations in terms of mechanical properties and structural composition. The growth of mycelium materials can exhibit quite large deviations in this respect, especially within different batches that are cultivated at different times. Thorough investigations include analyzing the variations within the same material, within the same batch, and those produced at different times. While this may seem quite extensive, it is necessary due to the complexity of the materials and the fact that this is not a "simple synthesis" but an organism that grows on its own. Only then are arguments based on just one or a few samples viable.

In my opinion, this article does not meet the high standards of this journal and needs to be completely revised before another submission to ensure compliance with scientific standards in both writing and experiments.

I suggest that the authors revise the entire manuscript in relation to two aspects:

1. Use appropriate language, avoid exaggerations, and provide arguments for each conclusion and claim.

2. Reduce the number of experiments and include only those that are informative for this work and provide reliable results. Do not include poorly designed experiments, such as the use of the humidifier when accurate relative humidity values are required, the sample preparation in Fig. S19a (separating the layers with a scalpel cannot be done reliably), and the entire setup in Fig. 2c (misleading description and conclusion).

Response: We thank Reviewer#2 for the assessment of our manuscript, valuable suggestions as well as the careful checking. In the revised manuscript, we have polished the language and refined the main text by moving some data to the Supporting Information. Meanwhile, as suggested, we have removed most experiments using the humidifier and replaced them with more accurate relative humidity control using a humidity chamber. We have also deleted previous Fig. 2c. Furthermore, the unreliable layer separation method for mycelium membrane has been replaced with a more precise instrument. In the following, we address each point systematically. All corresponding changes have been highlighted in yellow in the revised manuscript.

While it is not feasible to address all issues and discrepancies, many of which are likely due to language barriers, I will outline the key points below that the authors should consider:

1. The claim that low elongation rates are a limitation of mycelium materials is insufficient. As with all other materials, the mechanical property requirements are application-specific, and low elongation rates are no disadvantage in many instances.

Response: We thank the reviewer for this insightful comment. We agree that the statement "low elongation rates are a limitation" was overly absolute and lacked context. As the reviewer correctly points out, mechanical property requirements are highly dependent on the specific application; low elongation is not a disadvantage per se, but only a limitation in contexts where ductility is required. We have revised the manuscript accordingly to reflect this nuance.

Modification: According to your professional suggestions, we have now refined the introduction to specify that low elongation constitutes a drawback only in applications demanding high plasticity or energy absorption, whereas it may be acceptable or even desirable in fields prioritizing stiffness and dimensional stability. This opinion has been added in the abstract (page 1, lines 5-6) and main text (page 2, lines 37-43).

Abstract: "The developed materials demonstrate wide-range adjustable tensile performance…"

Main text: "However, despite these pioneering advances, existing mycelium-based materials remain limited by tunability of mechanical properties. Given the target application in rigid structural supports, where mechanical robustness and shape retention are prioritized over plastic deformation, this level of elongation is sufficient and does not pose a limitation. Conversely, for potential future applications requiring high flexibility, this property would need to be addressed. On-demand customization remains a critical challenge for widespread adoption."

2. Terms such as "tailored" and "programmable" suggest that the material can be adapted for specific bending angles in response to specific environmental conditions. This is not demonstrated, and I strongly doubt that it is possible given the likely varations in mechanical properties.

Response: We are grateful for the reviewer's critical and constructive comments. In our original submission, we did use terms like "tailored" and "programmable" without sufficient experimental support. Your criticism was valid.

In our manuscript, we had demonstrated that the bending angle changed reversibly with environmental humidity. This confirms that the material can adapt to specific environmental conditions, not just a static bending angle. In revision, we have performed three new sets of experiments to directly address your concerns:

1) Geometric and compositional tuning: We systematically varied width-length ratio (new Fig. 2b and c) and macerate composition, showing that the bending angle can be predictably adjusted over a range of $(-19.0 \pm 10.3)°$ to $(43.2 \pm 10.7)°$ (new Fig. 2d).

2) Cycling stability: Although over 3 long-period continuous humidification - dehumidification cycles, the bending angle shows 50% variation, our materials still recovered the deformation amplitude through drying process. It indicated that the influence of humidity on mechanical properties could weaken response deformation macerate material but this effect is reversible.

3) We have also discussed the remaining limitations regarding batch-to-batch mechanical variability.

Modification: Based on these new data, we now use "tailored" and "programmable" with clear qualification. Specifically, the material is programmable in terms of its equilibrium bending angle as a function of humidity, not in the sense of arbitrary reconfiguration. All changes in revised manuscript (page 6, lines 183-186) have been highlighted in yellow.

"In this section, we demonstrate a humidity-responsive material whose equilibrium bending angle can be programmed by tailoring geometric parameters and composition. The angle varies reversibly with relative humidity, and a library of bending angles, (-19.0 ± 10.3)° to (43.2 ± 10.7)° was achieved through structural design."

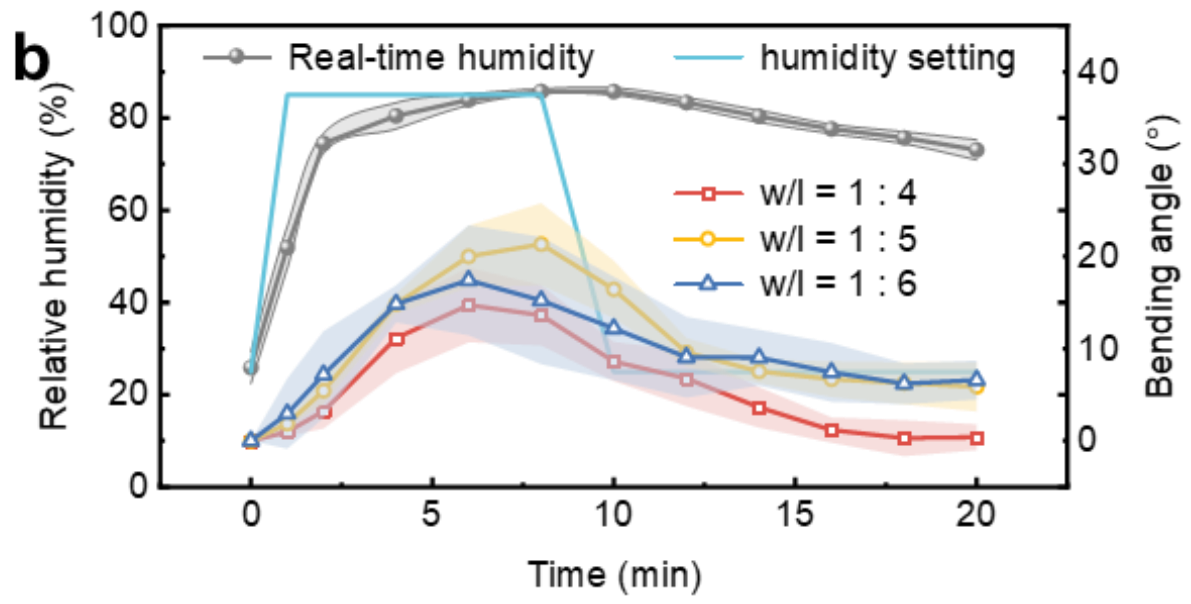

New Fig. 2b, Bending angle changes of Janus TV@P-G (10%PVA+10%Gly) specimen with different width to length ratios (w/l) in single fixed humidity response experiments, n=3. Humidity was controlled with humidity chamber.

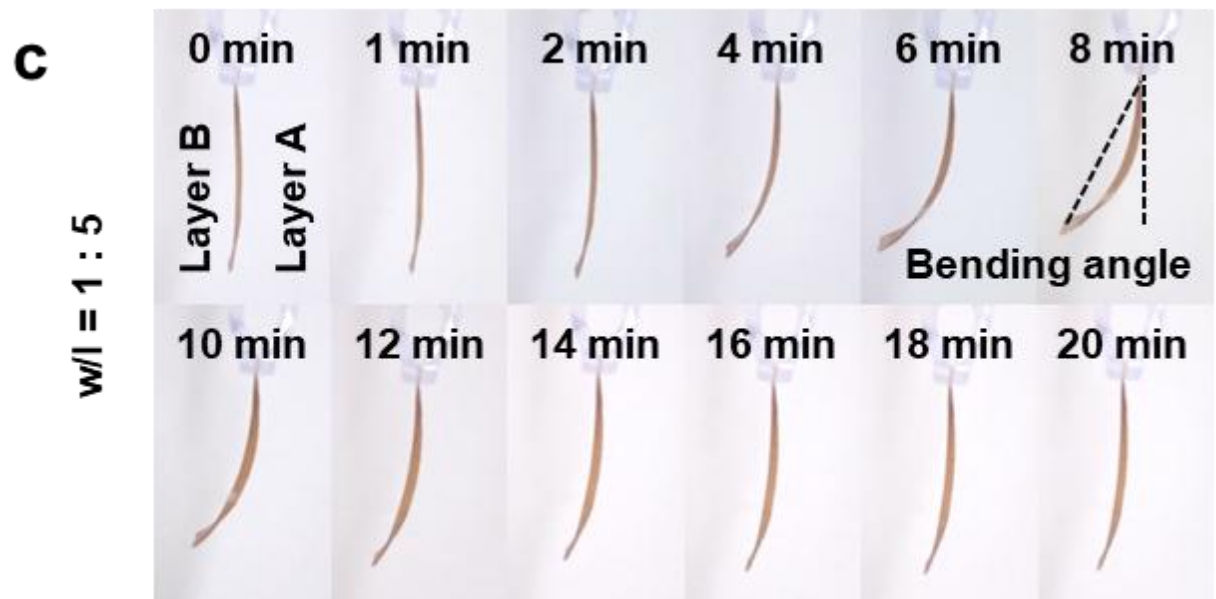

New Fig. 2c, Optical images of TV@P-G specimen (5 × 25 mm) with single fixed end deforming in hygroscopic process controlled in a humidity chamber, where humidity changed from 20% RH to 85% RH in 8 min, and decreased in the next 12 min.

3. There is hardly a common thread throughout the entire manuscript, especially given the presentation of all the experiments, and references to figures/panels are not in the correct order.

Response: Many thanks for pointing out these critical issues. To address these concerns, we have summarized a flow diagram (new Fig. R1) that outlines the sequential steps of our investigation, and we have substantially revised the manuscript accordingly. Specifically, the introduction has been shortened by removing repetitive sentences. We have also restructured Section 1 to improve readability. In addition, we have carefully reviewed the entire manuscript to eliminate redundant expressions and wordiness. Overall, we have reduced the manuscript length by about 300 words while preserving all technical content.

Modification:

In order to adjust the writing sequence, we identified our thread and presented it using a fishbone diagram (new Fig. R1).

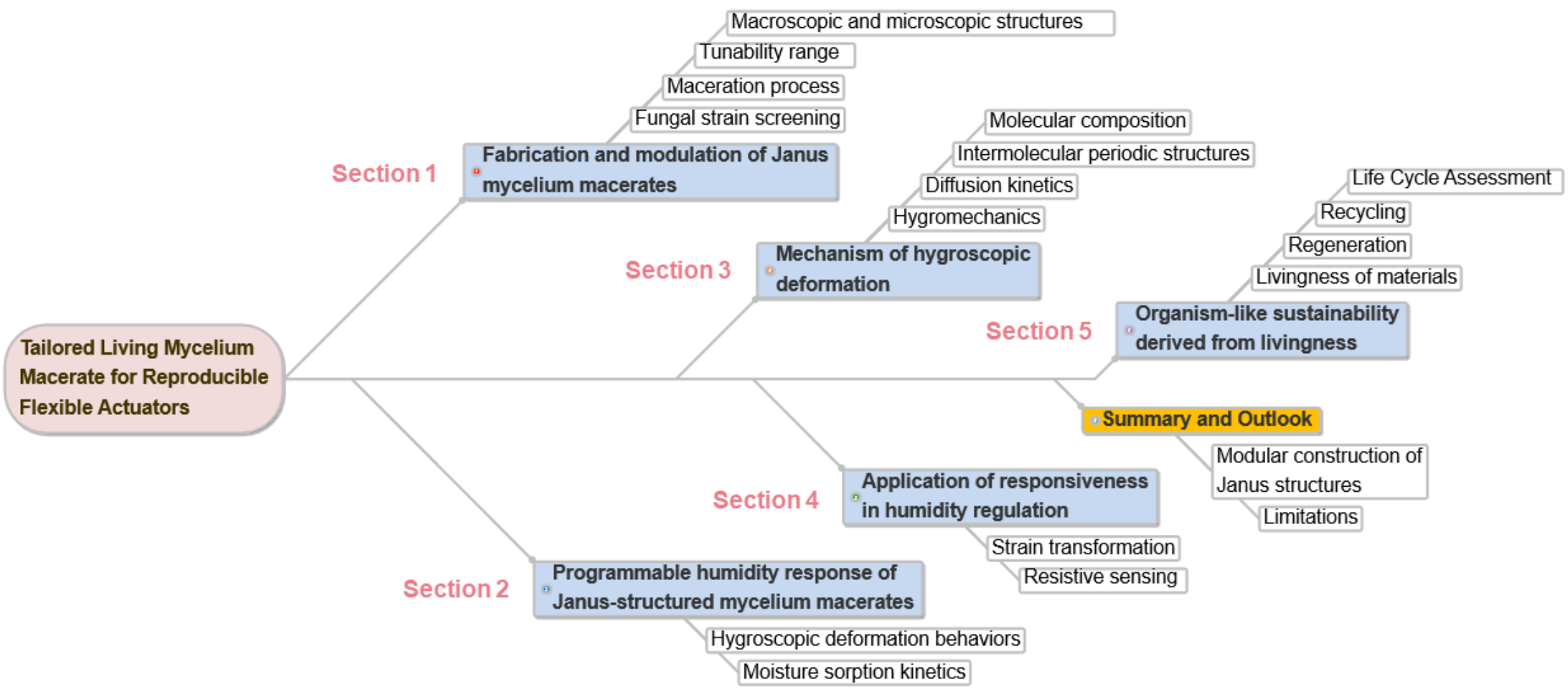

New Fig. R1 Fishbone diagram summarizing the logical flow of the study. The head of the fish defines the central question: How to adjust the mechanical properties and environmental (humidity) responsiveness of mycelium materials on demand, while maintaining the properties of fungal dormancy recovery and nature of the subculture? The spine follows the order of investigation: 1) fabrication and modulation, 2) humidity responsiveness, 3) deformation mechanism, 4) concept presentation of application, 5) livingness and sustainability, 6) summary and outlook.

We have restructured and shortened the last paragraph of Introduction, page 2. lines 59-66.

"To overcome the inherent brittleness of dry mycelium, we applied a PVA network and glycerol to enhance flexibility and moisture absorption, establishing the concept of "macerate" that preserves cell viability and the Janus structure. By maceration engineering that manipulated the inherent physical property of polysaccharide-dominated fungal networks, we achieved programmable hygroscopic deformation through engineered structural asymmetry. Our findings reveal that the Janus-type deformation arises from molecular-level heterogeneity in both hygromechanics and entropy-driven reorganization."

In Section 1, redundant descriptions of experimental procedures have been removed. And we have added a one-sentence summary for cultivation period optimization, page 3, lines 88-90.

"Taking solid content (Fig. S1e), mechanical property in fresh state (Fig. S2a) and cultivation period into comprehensive consideration, we selected 20 days as the optimal cultivation period for mushroom specie TV."

Previous schematic drawing of mycelium macerate fabrication in Fig. 1 has been merged as revised Fig. 1a.

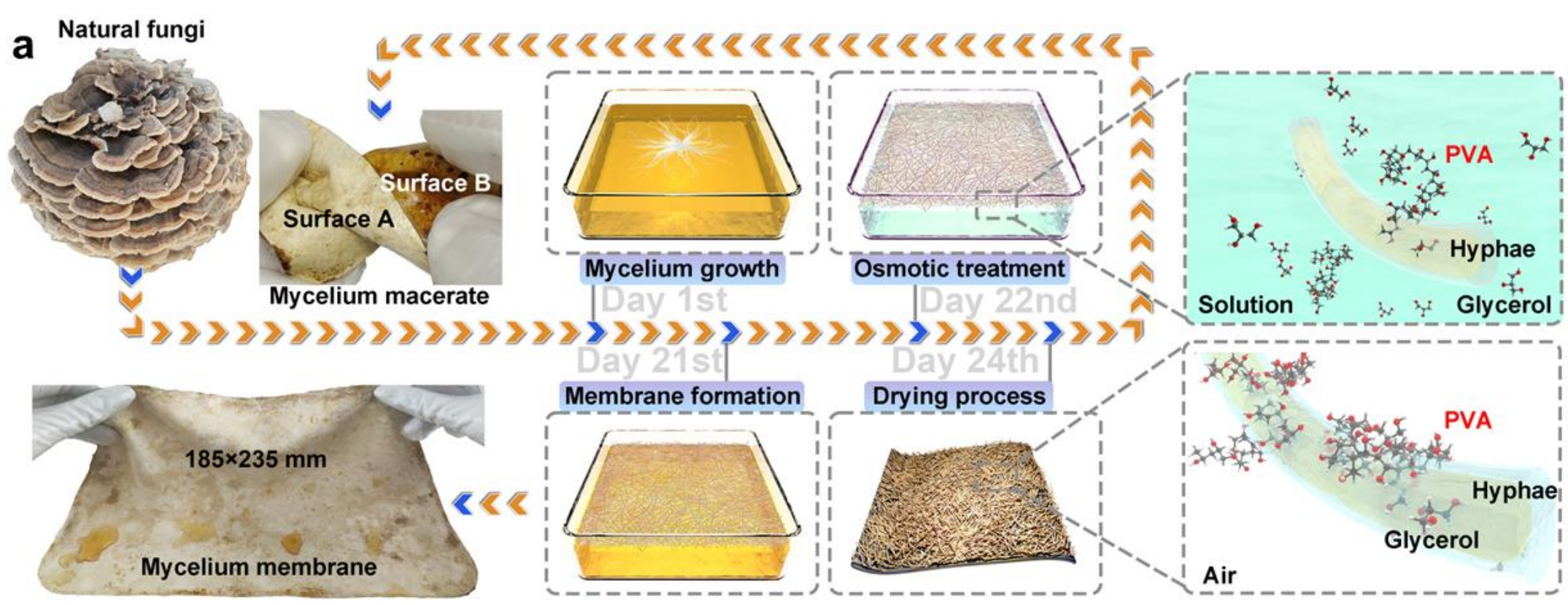

Revised Fig. 1a Schematic drawing of liquid state fermentation to preparing Janus mycelium macerates, four steps

from mycelium growth, membrane formation, osmotic treatment to drying process. Insets respectively indicate the process of PVA and glycerol permeating into hyphae during osmotic treatment, as well as the concept of macerate that PVA and glycerol are sequestered by hyphae after dehydration. Insets: Common wild *Trametes* species in Harbin Institute of Technology. Native mycelium membrane of Trametes versicolor via liquid state fermentation in scale of 185×235 mm. Janus mycelium macerates showcasing top surface A, bottom surface B and flexibility to bend at will.

We simplify the description of the macroscopic property of mycelium macerate, page 3, line 92-95.

"For air application, all samples were ambient-dried. The dried membrane without glycerol was brittle, highlighting the necessity for a plasticizer to reduce biopolymer interactions. Glycerol enabled mycelium macerate, TV@P-G to achieve high flexibility (bending: Fig. S3a and 3b; curling: Fig. S3c)."

We have moved the paragraph of the original manuscript about the Janus structure characterization behind our material preparation in Section 1, page 3, lines 104-110.

"The Janus structure of mycelium membrane is well reserved after maceration treatment. Difference in surface and cross section micromorphology of TV@P-G was observed by SEM (Fig. 1d). Viewed from cross section, the top layer corresponds to aerial hyphae that grows horizontally over the medium surface (layer A), whereas the bottom layer consists of nutritive hyphae growing inward medium (layer B). By altering the cultivation time, the Janus structure of TV@P-G was apparent in optical and SEM images (Fig. S6)."

We next transferred figures concerning structure characterization from previous Fig. 1 to revised Fig. 1b and 1c. Meanwhile, previous Fig. 2g has been adjusted as revised Fig. 1d.

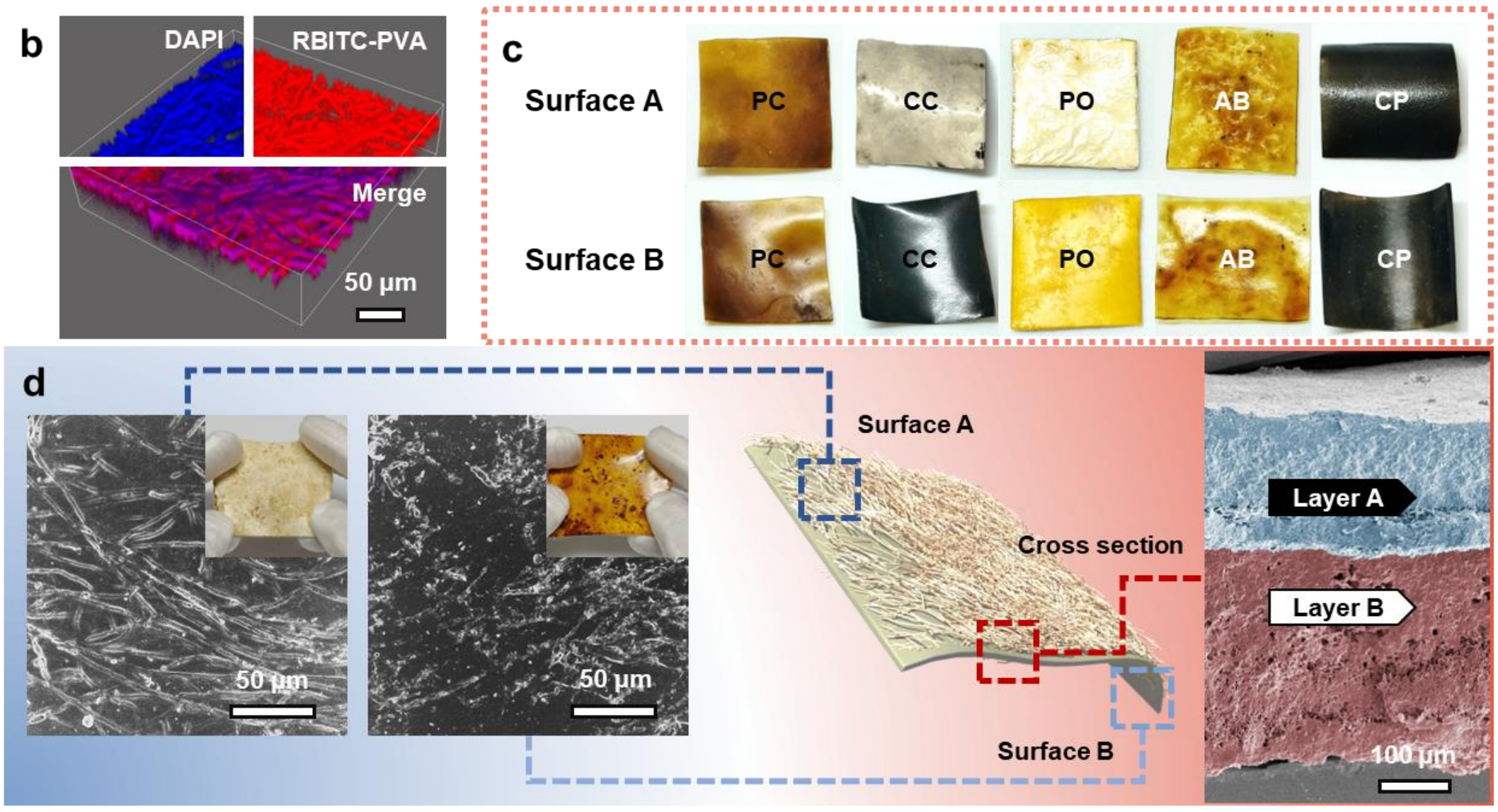

Revised Fig. 1b, 3D confocal images of DAPI (bule) dyed TV@(RBITC-PVA)-Gly (red) section. **c**, Optical images from top (surface A) and bottom (surface B) of Janus mycelium macerates fabricated from *Phanerochaete chrysosporium* (PC), *Copyinds comatus* (CC), *Pleurotus ostreatus* (PO), *Agaricus bisporus* (AB) and *Cladophialophora* sp. (CP). **d**, Schematic illustration of Janus TV@P-G structure and SEM images of top surface A, bottom surface B, and cross section. Layer A was pseudo-colored with blue and layer B was red in SEM image of cross section. The photographs shown as insets display top surface A and bottom surface B, respectively…

We have summarized (page 5, lines 145-147) and visualized (new Fig. 1f) the tunability ranges of different mechanical property parameters.

"Additionally, we defined the tunability range that referred to the degree of freedom of our method to modulate the mechanical properties of materials, intuitively quantifying the regulatory potential (Fig. 1f)."

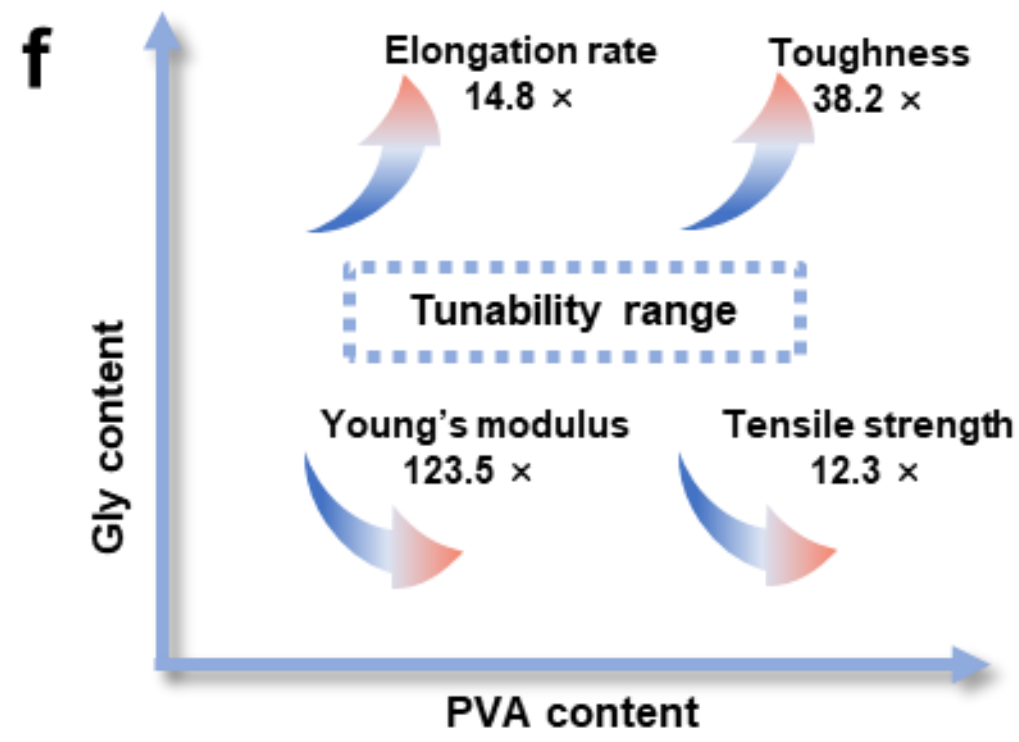

New Fig. 1f, Summary illustrating the tunability range of mechanical property parameters tailored by maceration engineering.

Overly detailed strengthening mechanism of PVA and plasticizing mechanism of glycerol have been moved to the Supplementary Information (page 26, lines 524-558) to avoid cluttering the main text. And the brief conclusion has been provided in main text, page 5, lines 150-153.

Supplementary Information: **"Strengthening mechanism of PVA and plasticizing mechanism of glycerol**

To elucidate the strengthening mechanism of PVA, the investigation on intermolecular interaction was carried out. In thermogravimetry (TG) tests (Fig. S15a), weight reduction and thermal decomposition of mycelium biomass were delayed by PVA, owing to their intermolecular interaction··· Therefore, as small molecule, glycerol promotes chains slide in mycelium elongation remarkably. Similarly, PVA forms close interaction with biomacromolecules (E_{int}, PVA-chitin = -84.99 kJ·mol^{-1}, E_{int}, PVA-glucan = -59.85 kJ·mol^{-1}) but strong intramolecular force (E_{int}, PVA-PVA = -92.09 kJ·mol^{-1}), thus PVA chains enhances the weak interaction between biomacromolecules, as non-covalent bonding agent (Fig. S19)."

Main text: "Strengthening mechanism of PVA and plasticizing mechanism of glycerol were both elucidated in our supplementary discussion (Fig. S12-17), which can be summarized that PVA enhances the hydrogen bond network among biomacromolecules, while glycerol promotes sliding of macromolecular chains."

We moved the part that discussed the differences in mechanical properties between layer A and layer B to the end of Section 2. We also adjusted the detailed descriptions into the figure captions of Fig. S18, retaining the main conclusions while adding transitional sentences (page 5, lines 154-159) before and after this paragraph.

"Based on the integral strengthening of mycelium macerate, mechanical property differences between Janus structure were investigated. Interestingly, layer B broke later than layer A during tensile tests (Fig. S18a-d). Additionally, Janus TV@P-G could be separated along the interface between layer A and B. Tensile tests of separated layer A and B (Fig. S18e) further confirmed that *TS*, *EG* and *T* of layer B were higher than layer A, whereas E of layer A was larger. The asymmetrical modulus is a basics of inner stress and deformation."

We have streamlined the Section 3 concerning hygroscopic deformation mechanism and used concise sentences to clarify the logical relationship, page 6, lines 188-189.

"The hygroscopic deformation of Janus mycelium macerates arises from differential skeletal expansion between layers, driven by asymmetry hygromechanical responses."

We have shortened the results of micro-CT by removing details of data processing method, pages 6, lines 197-201.

"Isolated layers A and B showed no statistically significant difference in planar expansion rates (Fig. S28), prompting

micro-CT examination of the intact macerate during hydration (Fig. 2h). Bulk moisture expansion rate (εb) was higher for layer A (9.27%) than layer B (3.38%) (Fig. 2i), revealing that layer A was dominant in hygroscopic deformation. Dry-state porosity was 3.75% higher in layer A (37.31±2.16% vs. 32.42±1.33%, Fig. S29)"

We adjusted the summarized illustration of hygroscopic deformation mechanism from previous Fig. 5a into revised Fig. 3f, in order to concentrate our discussion on hygroscopic deformation mechanism in Fig. 3.

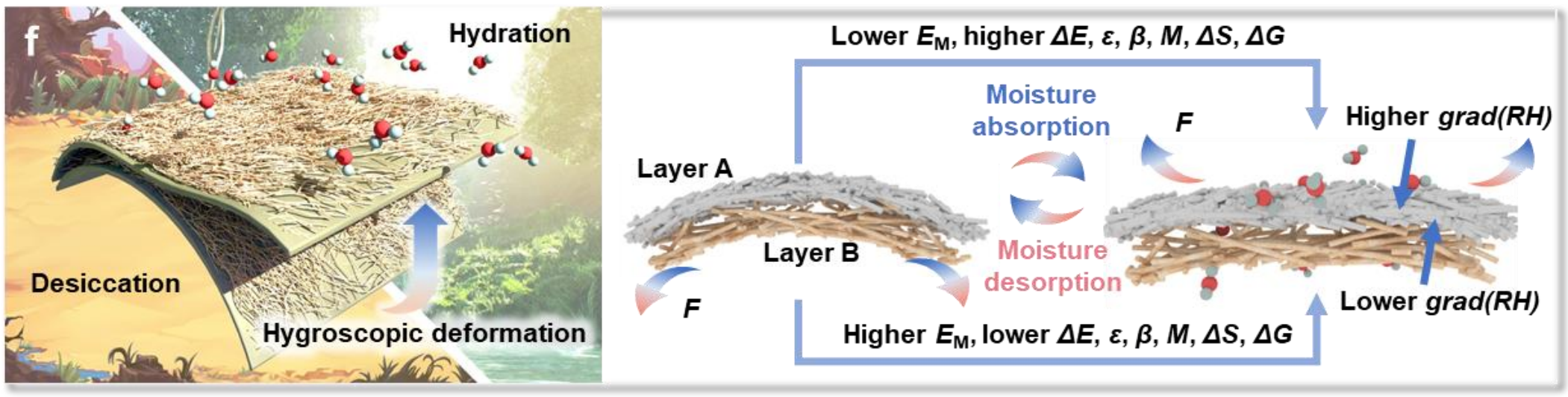

Revised Fig. 3 Mechanism investigation on hygroscopic deformation of Janus mycelium macerates… **f**, Schematic illustration of hygroscopic deformation mechanism.

We also adjusted the simulated results of radial distribution function and coordination number from previous Fig. 4f into revised Fig. S54.

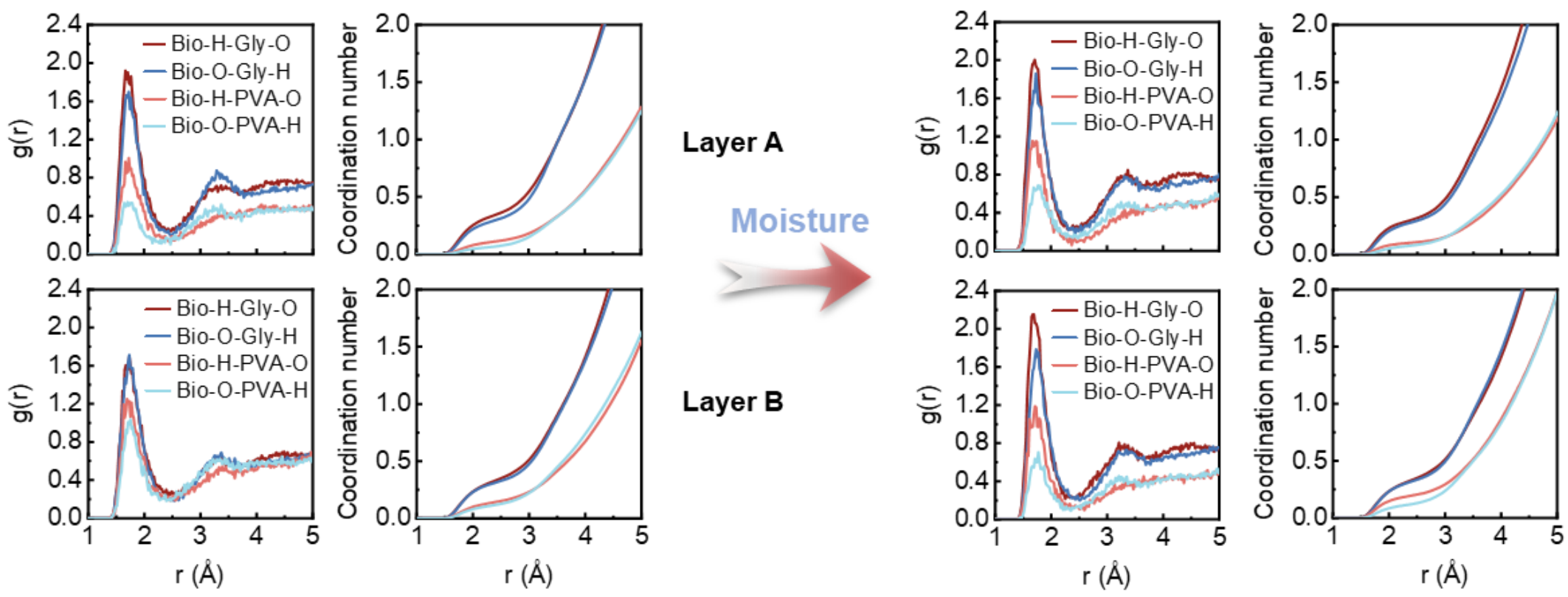

Revised Fig. S54 Influence of moisture on MD simulation radial distribution function and coordination number between biomacromolecules and PVA or glycerol in layer A and B.

To highlight the main thread, we have added a schematic diagram summarizing the research content of this part in revised Fig 5.

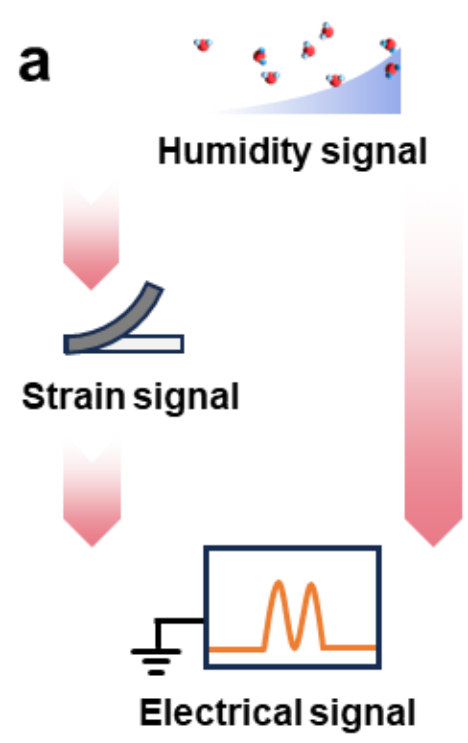

Revised Fig. 5 Janus mycelium macerate applied to sensing. **a**, Schematic diagram of mycelium macerate converting humidity signals into other signals, used to summarize the research content in this section.

We have systematically re-ordered all in-text references to figures and panels to follow the order of appearance in the manuscript main text. The figures have been re-numbered where necessary.

Page 6, lines 163-165: "…exhibiting excellent reversible hygroscopicity with an average moisture uptake of 374.93 mg·g^{-1} (25°C, 85% RH) in 5 consecutive cycles (Fig. 2a)."

Page 6, lines 175-176: "By contrast, constrained deformation tests with single end constrained revealed consistent bending orientation towards layer B across various width-length ratios (Fig. 2b, c, Fig. S23) and…"

Page 7, lines 207-208: "Moisture adsorption induced pore compression through skeletal expansion, reducing porosity in both layers (A: P = 9.38 ± 1.03%; B: P = 7.02 ± 1.05%) (Fig. S32) and homogenizing pore morphology, as evidenced by shifts in diameter distribution and sphericity (Fig. S33 and 34)."

We apologize for the incorrect citation of the Fig. 2f, g and have made corrections.

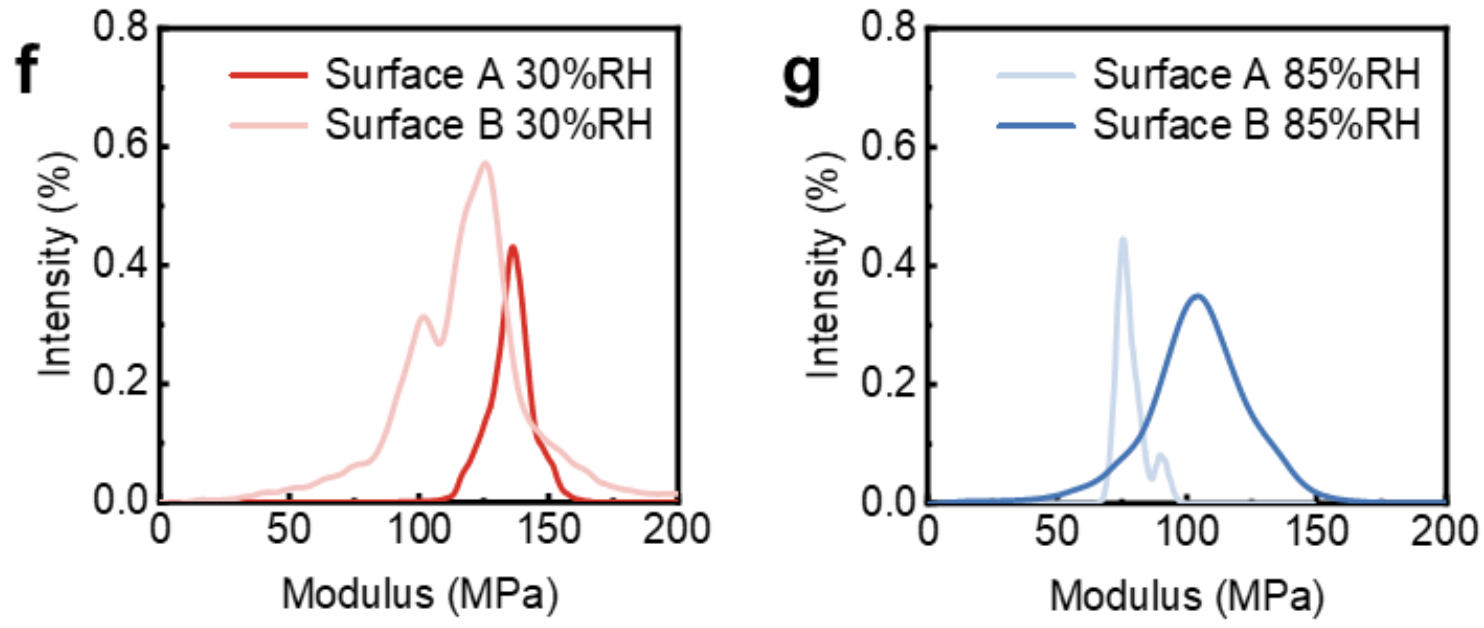

Revised Fig. 2f, g, Difference between DMT modulus distribution of Janus TV@P-G upon exposure to a dry environment (R.T., 30% RH) (**f**) and wet environment (R.T., 85% RH) (**g**), quantifying by AFM.

4. Each experiment requires descriptions with all relevant information so that others can replicate it, including sample geometries, statistics, environmental conditions, etc.

Response: We sincerely thank you for this valuable suggestion. We agree that sufficient experimental details are essential for reproducibility. Accordingly, we have revised the manuscript to include the missing information as follows:

1) Tensile testing: We now specify the ASTM D882-18 standard, sample dimensions (width in 10 mm, initial grip separation in 80 mm, total length in 100 mm), test speed (5 mm/min), and environmental conditions (23 ± 2°C, 30 ± 5% RH). Statistical details include n = 5, mean ± SD, and two-tailed t-test with $\alpha = 0.05$.

2) Humidity-responsive experiments: Sample sizes (5 × 20, 5 × 25, 5 × 30 mm), humidity changed conditions (from 30 ± 5% to 85 ± 1% RH), and the measured output (bending angle) are now clearly stated. Each condition was tested with n = 3. The humidification time for the samples made of TV were set at 8 minutes from empirical attempts, while for the other samples, it was set at 8 to 15 minutes.

3) Expansion ratio testing: Statistical details include n = 10, mean ± SD, and two-tailed t-test with $\alpha = 0.05$. Cohen's d was calculated as ($mean_1$ - $mean_2$) / pooled standard deviation.

Modification: We added detailed dimensions and shapes of all tested samples in pages 2-3, lines 82-85 of Supplementary Information, Section Mechanical tensile tests.

"Following ASTM D882-18 standard, sample dimensions were normalized in width of 10 mm, initial grip separation of 80 mm, total length of 100 mm. These rectangular shape specimens were prepared by a mold cutter. The thickness of specimens was measured by a vernier caliper. The test speed was set as 5 mm/min, and environmental conditions stayed in 23 ± 2°C, 30 ± 5% RH."

We also explicitly state that thickness could not be precisely controlled due to the fabrication process, and we provide thickness ranges in new Figure S9. All tensile results are engineering stress based on initial width, with thickness variations noted.

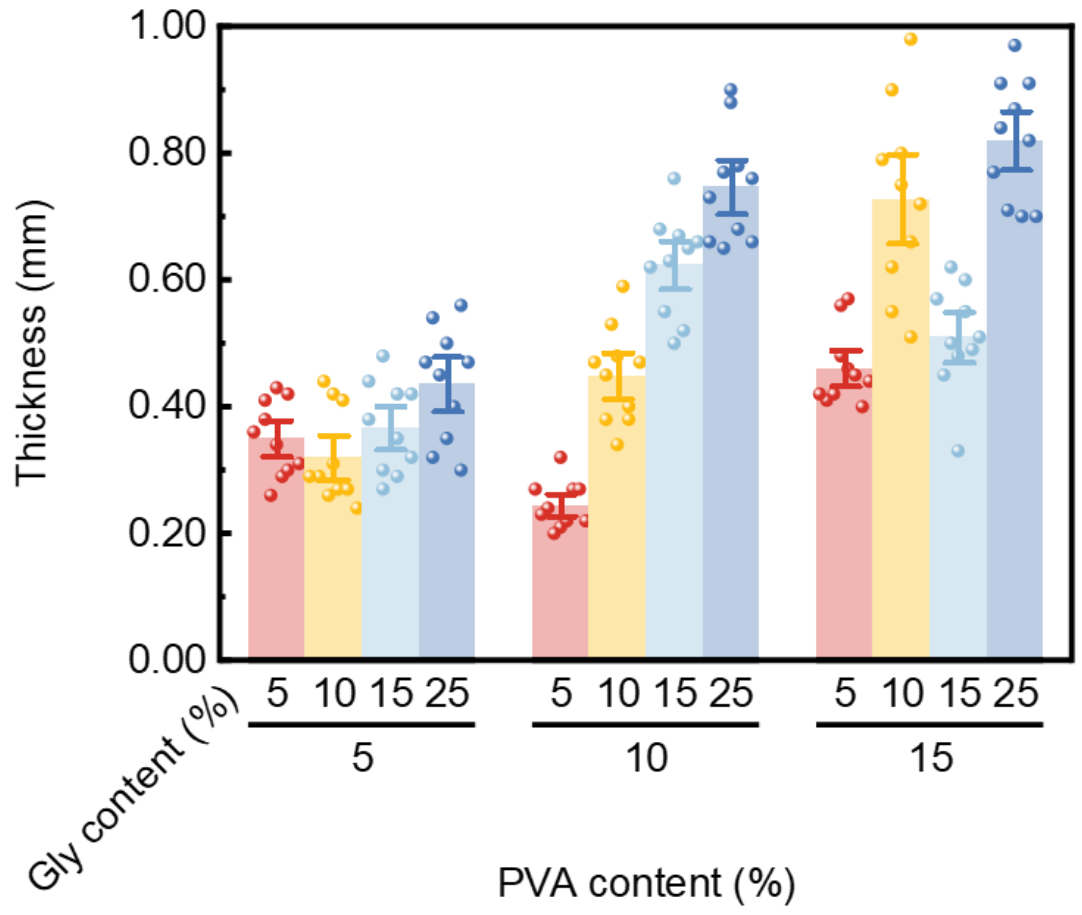

New Fig. S9 Thickness of TV@P-G macerates treated with different PVA and glycerol contents. Each group of experiments was repeated 10 times (n = 10). Data are expressed as mean ± standard deviation (SD).

Due to the large variation in mean values among the 12 experimental groups (fold differences), statistical comparisons were performed only between groups with overlapping or closely positioned mean values (difference < 20% of the larger mean). For these comparisons, two-tailed independent t-tests ($\alpha = 0.05$) were used. Groups with clearly separated mean values (> 1.5-fold difference) were considered significantly different without further testing. Detailed statistical results were supplemented as new Fig. S10.

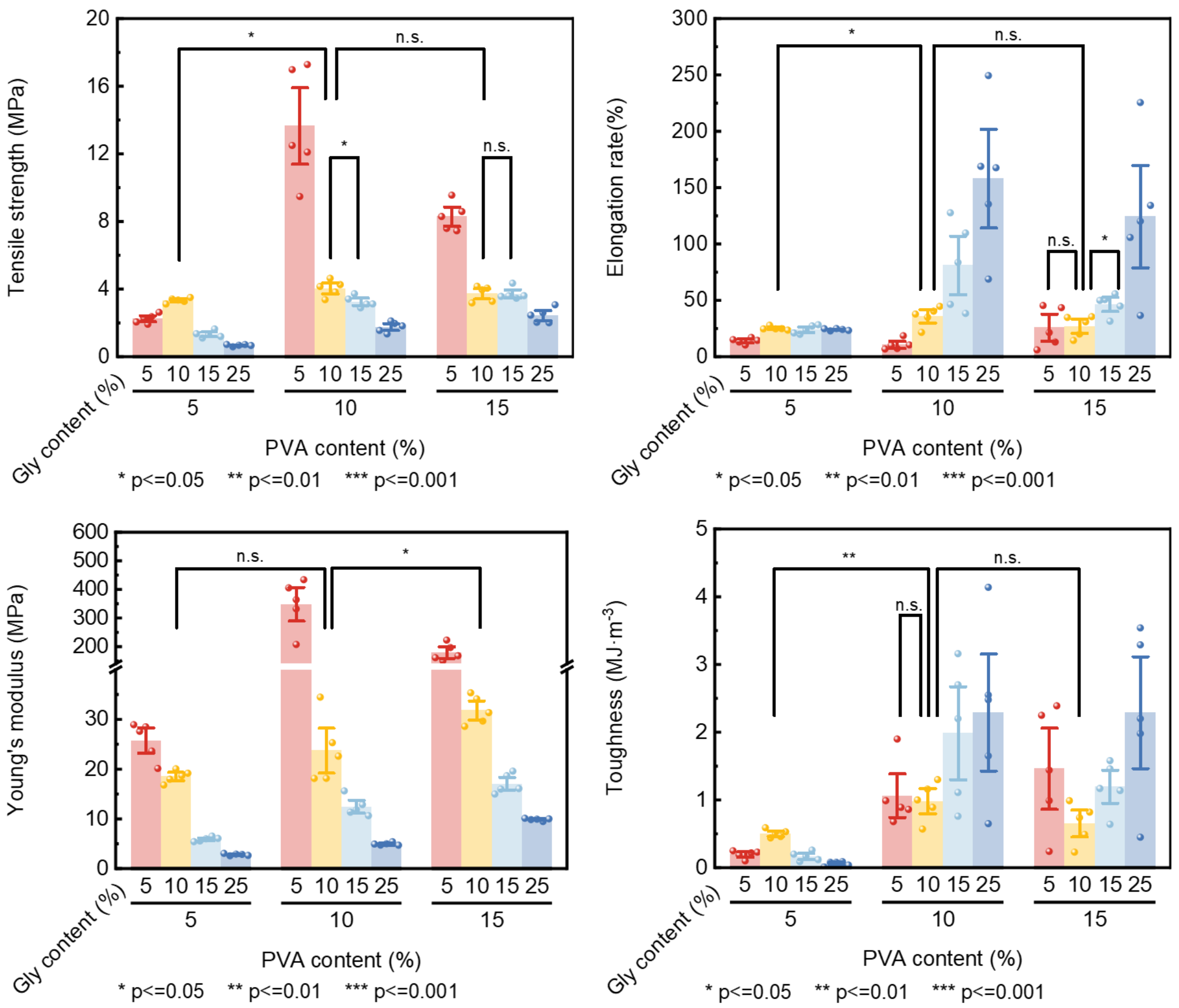

New Fig. S10 Statistically analyze the influence of different PVA and glycerol contents on the mechanical performance parameters (*TS*, *EG*, *E* and *T*) of TV@P-G macerate, and conduct screening by comprehensively considering the mechanical performance and material input. Each group of experiments was repeated 5 times (n = 5). Data are expressed as mean ± standard deviation (SD). A two-tailed t-test was used to compare similar data.

5. Experiments involving humidity appear to be rather inaccurate, as the humidified air is only a small jet from the device. I cannot imagine how a sensor nearby can measure the exact value here. For all experiments requiring an accurate value for relative humidity, use a chamber.

Response: We sincerely thank you for your careful check and comments. According to your professional suggestions, we repeated our experiments about hygroscopic deformation of mycelium macerate using a humidity chamber with the function of humidity controlling and visualization. Limited by the power of air pump and weak adsorption property of silica gel, the dehumidification process was slower than humidification, demonstrated by new Fig. S21. In the revised manuscript, we have repeated all experiments on investigating the differences in mycelium materials hygroscopic deformation in this humidity chamber. The detailed modifications are as follows:

Modification: We added the instrument model of humidity chamber in Supplementary Information, page 6, lines 152-154.

"…humidity chamber (model: UV-U3, voltage: 220 V, flow: 10-20 L/min, power: 24 W, vacuum: -90 kPa, YOUCHENGINDUSTRIALCO.LTD.)"

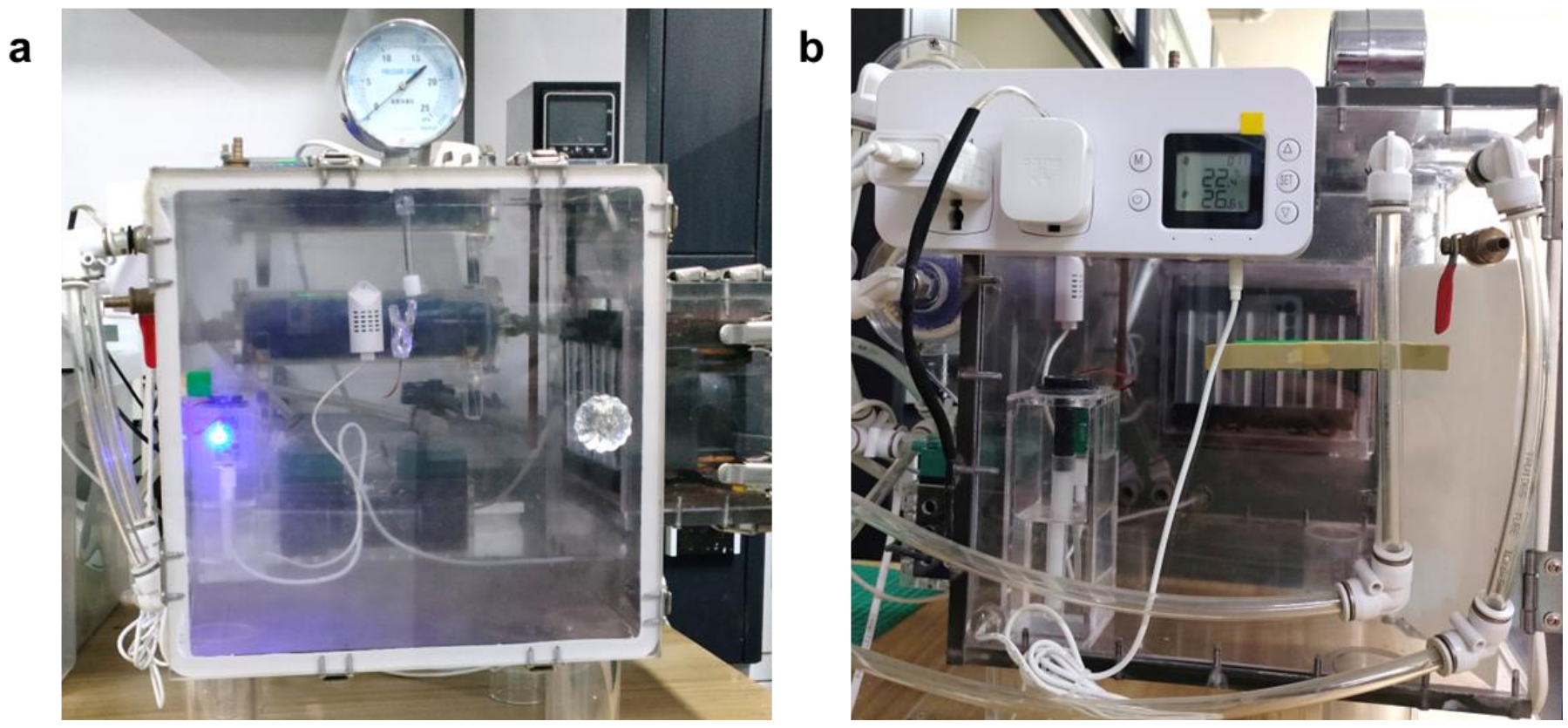

New Fig. S21 Optical images from the front (**a**) and left (**b**) view of the humidity chamber with inner humidity visualization, which was used to control the humidity and create a relatively homogeneous environment in this work.

Using this humidity chamber, we conducted a series of new experiments to investigate the influencing factors of material hygroscopic deformation, and replaced the original data in the manuscript. First, we repeated the hygroscopic deformation behaviors of samples with different width to length ratios (w/l) in the humidified chamber. Image-Pro Plus software was used to measure bending angle changes over time, displayed as new Fig. 2b. Photographs recording the specific deformation process were displayed in new Fig. 2c and new Fig. S23.

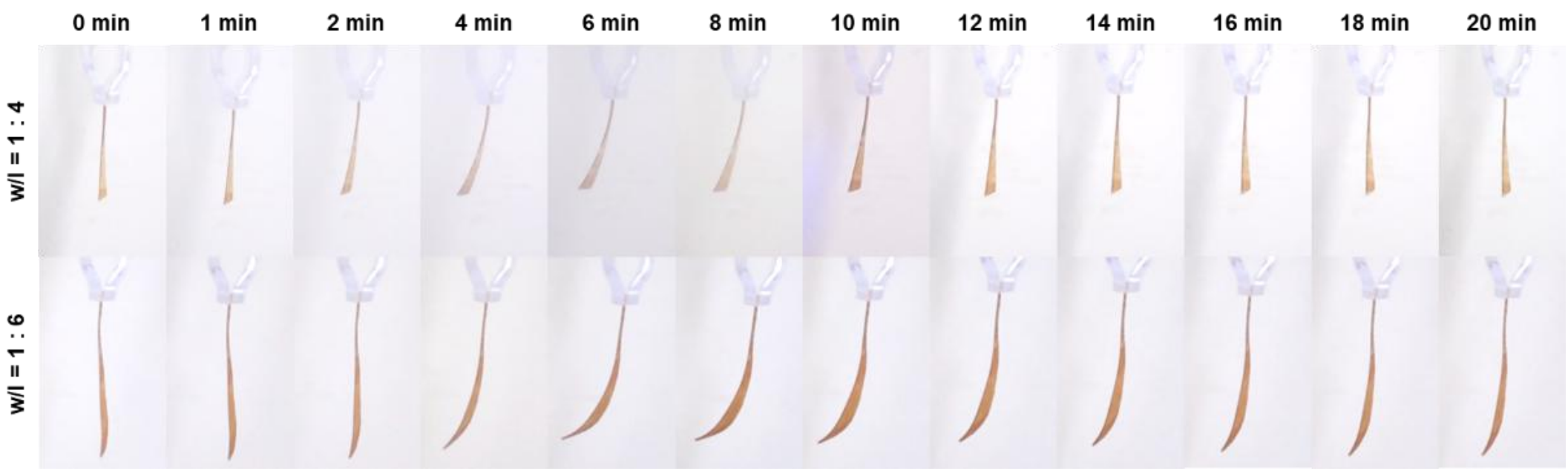

New Fig. S23 Optical images of TV@P-G specimen (5 × 20, 5 × 30 mm) with single fixed end deforming in hygroscopic process controlled in a humidity chamber, where humidity changed from 20% RH to 85% RH in 8 min, and decreased in the next 12 min.

We repeated the hygroscopic deformation behaviors of samples treated with different glycerol contents in the humidified chamber, and added the qualitative results in new Fig. S24. Image-Pro Plus software was used to measure bending angle changes over time, displayed as new Fig. 2d.

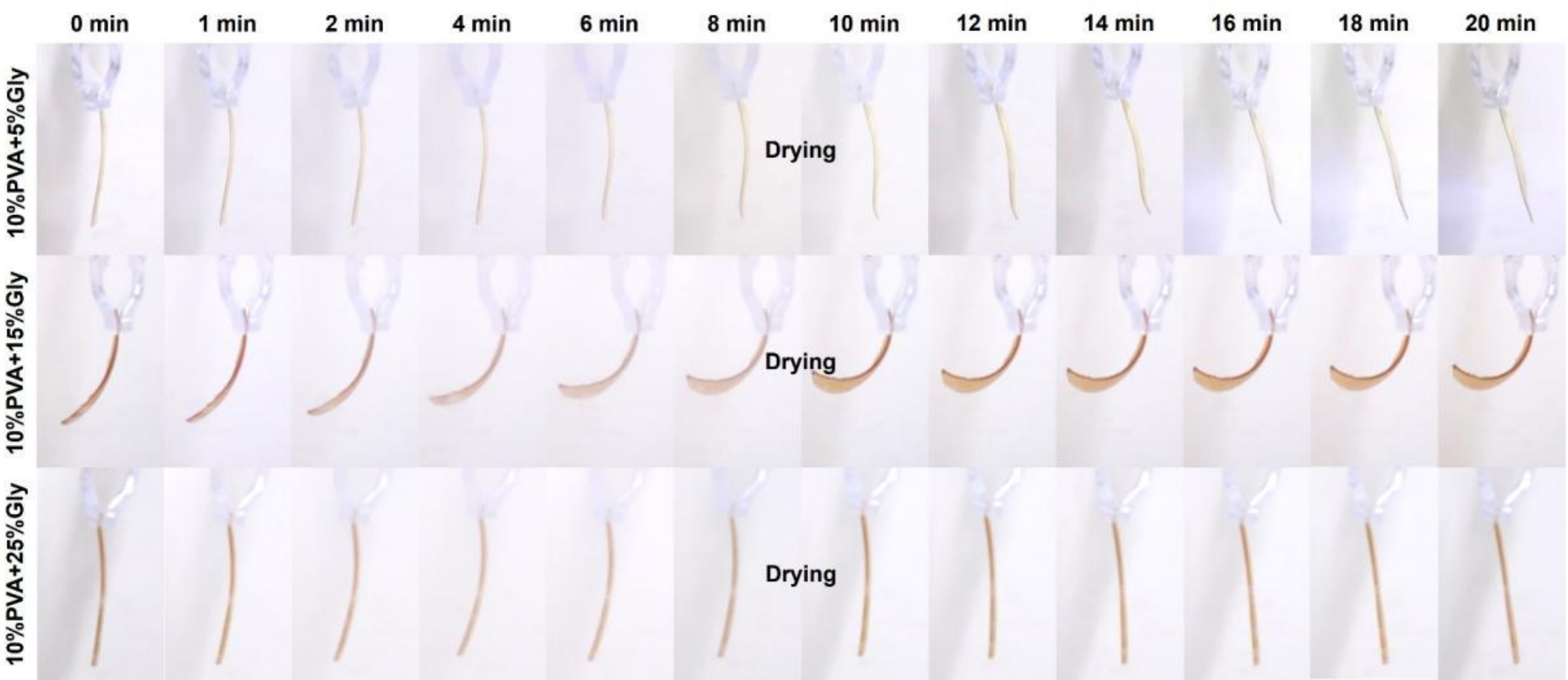

New Fig. S24 Optical images of various glycerol content treated TV@P-G specimen (5×25 mm) with single fixed end deforming in hygroscopic process controlled in a humidity chamber, where humidity changed from 20% RH to 85% RH in 8 min, and decreased in the next 12 min.

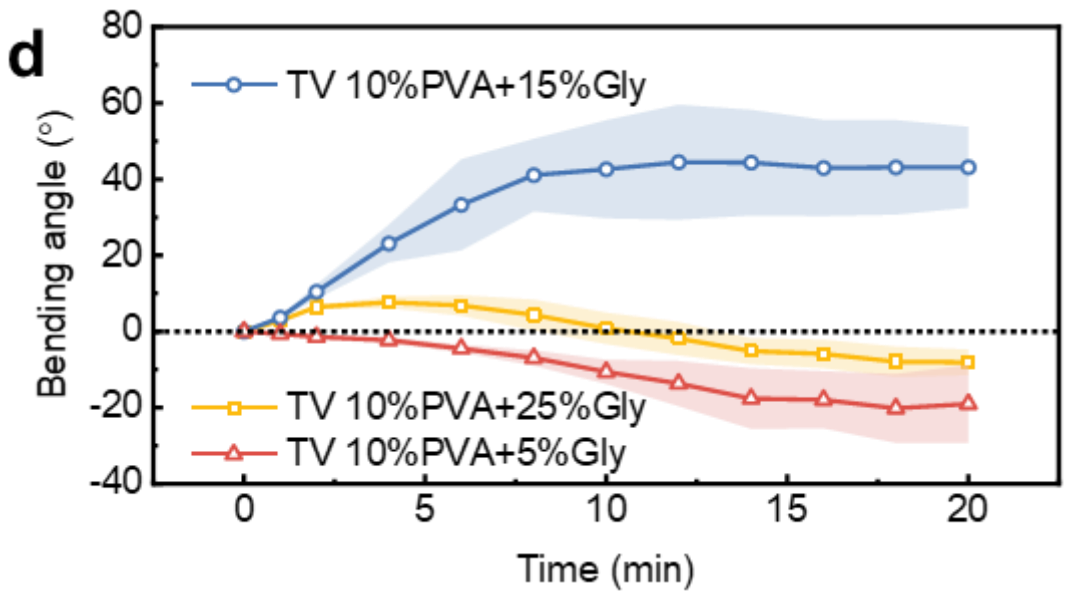

New Fig. 2d Bending angle changes of Janus TV@P-G treated with different Gly content (5%, 15% and 25%Gly), conducted in humidity chamber, n=3. The corresponding photograph was extracted from the video by time and the bending Angle was measured by Image-Pro Plus software. Error bars (mean ± SD) were connected and visualized as a shadow effect around the data points.

Lastly, we repeated the hygroscopic deformation behaviors of samples prepared by different fungal species in the humidified chamber, and presented the qualitative results in new Fig. S78.

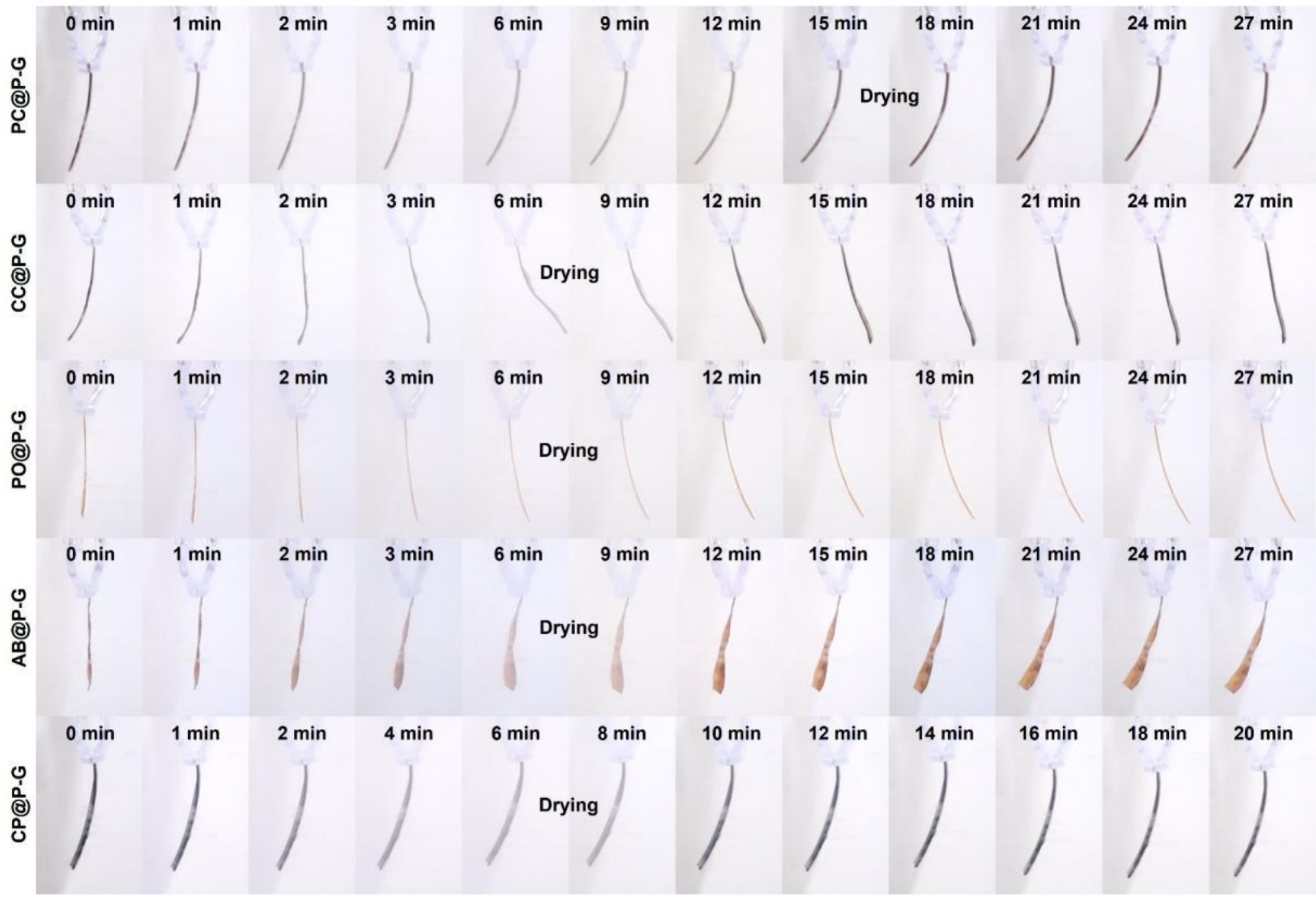

New Fig. S78 Optical images of PC@P-G, CC@P-G, PO@P-G, AB@P-G and CP@P-G with single fixed end deforming in hygroscopic process conducted with humidity chamber. Time marked in black and white respectively correspond to deformation and recovery process. The opposite curvature may involve multi-factor asymmetries between layers of different species, including density gradient differences, fractal dimension asymmetries, etc.

6. The contour maps in Fig. 2 are very detailed, but are based on only a few measurements in Fig. S9. How is all the missing information generated, or did the authors forget to include a data set? This is particularly interesting because the "best" result is not included in the measurements.

Response: We sincerely thank you for your careful check and comments. We revised these figures into heat maps and supplemented the data set in Table S2, based on our 12 groups of samples treated with PVA (5%, 10% and 15%) and glycerol (5%, 10%, 15% and 25%) contents.

The influence of PVA and glycerol on the material still reflects the trade-off between the material's strength and toughness. In the available data set, we selected group, 10%PVA+10%Gly as the optimal condition. This selection was based not only on the mechanical property range of mycelium material, considering that under this condition our material should have an elastic modulus of less than 50 MPa to meet the requirement of easy deformation, but also required a tensile strength of 3 MPa or more, an elastic modulus of 10 MPa or more, an elongation of 30% or more to enhance the toughness of the material.

Modification: We followed the reviewer's suggestions and increased the content of PVA beyond the optimal conditions selected. The samples with PVA content of 0 were excluded from the dataset because of their poor mechanical properties. The modified PVA contents were set at 5%, 10% and 15%, while the glycerol content was 5%, 10%, 15% and 25%. A total of 12 data points were used, with each group consisting of 5 parallel experiments. The stress-strain curves of the 12 groups of data are shown in new Fig. S7.

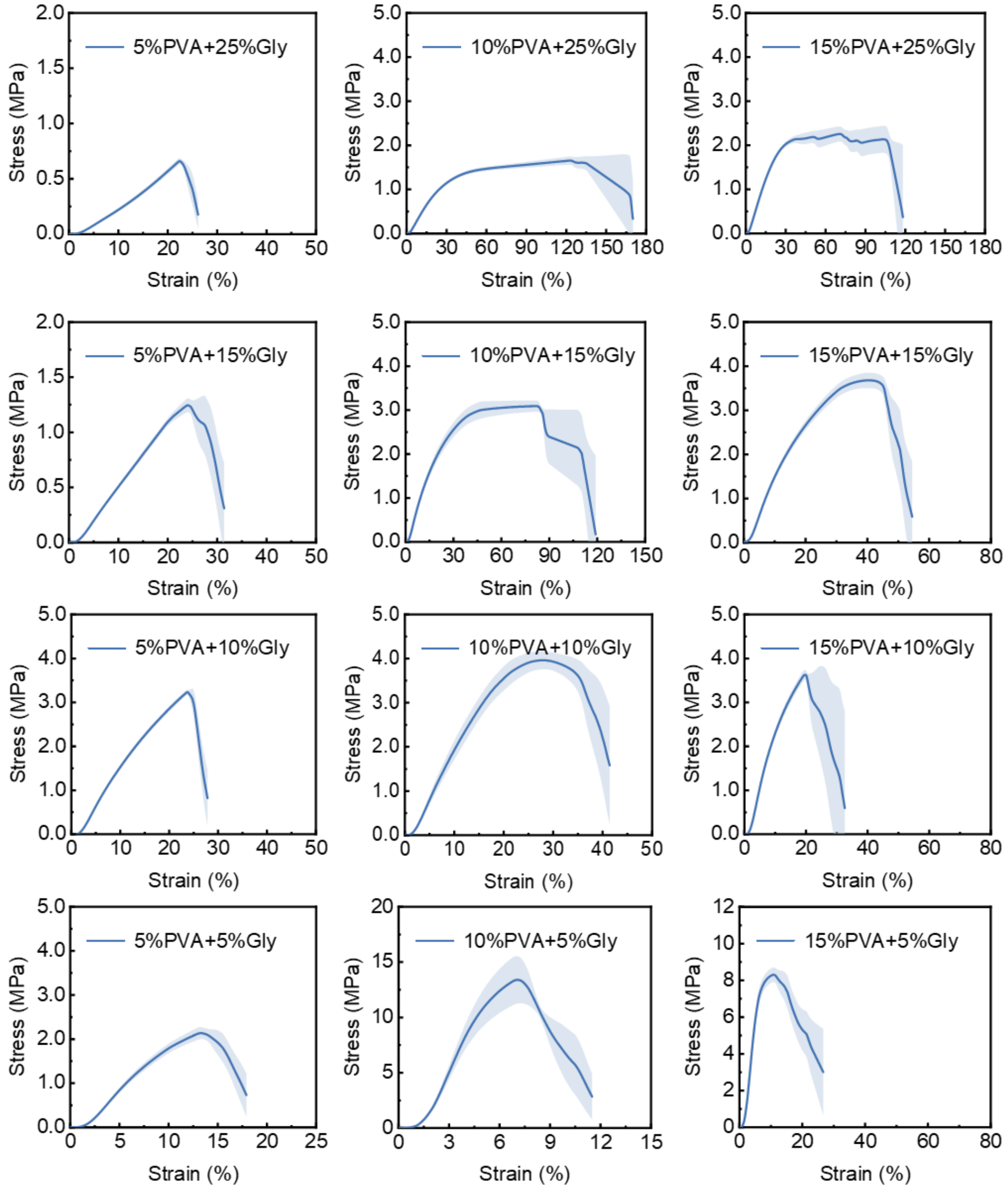

New Fig. S7 Tensile stress-strain curves of TV@P-G treated with 5-15% PVA and 5-25% glycerol, n=5. Error bars were connected using B-spline basis function and visualized as a shadow effect around the data points.

We have also placed photographs of specimen in new Fig. S8 and added specimen size in the figure note.

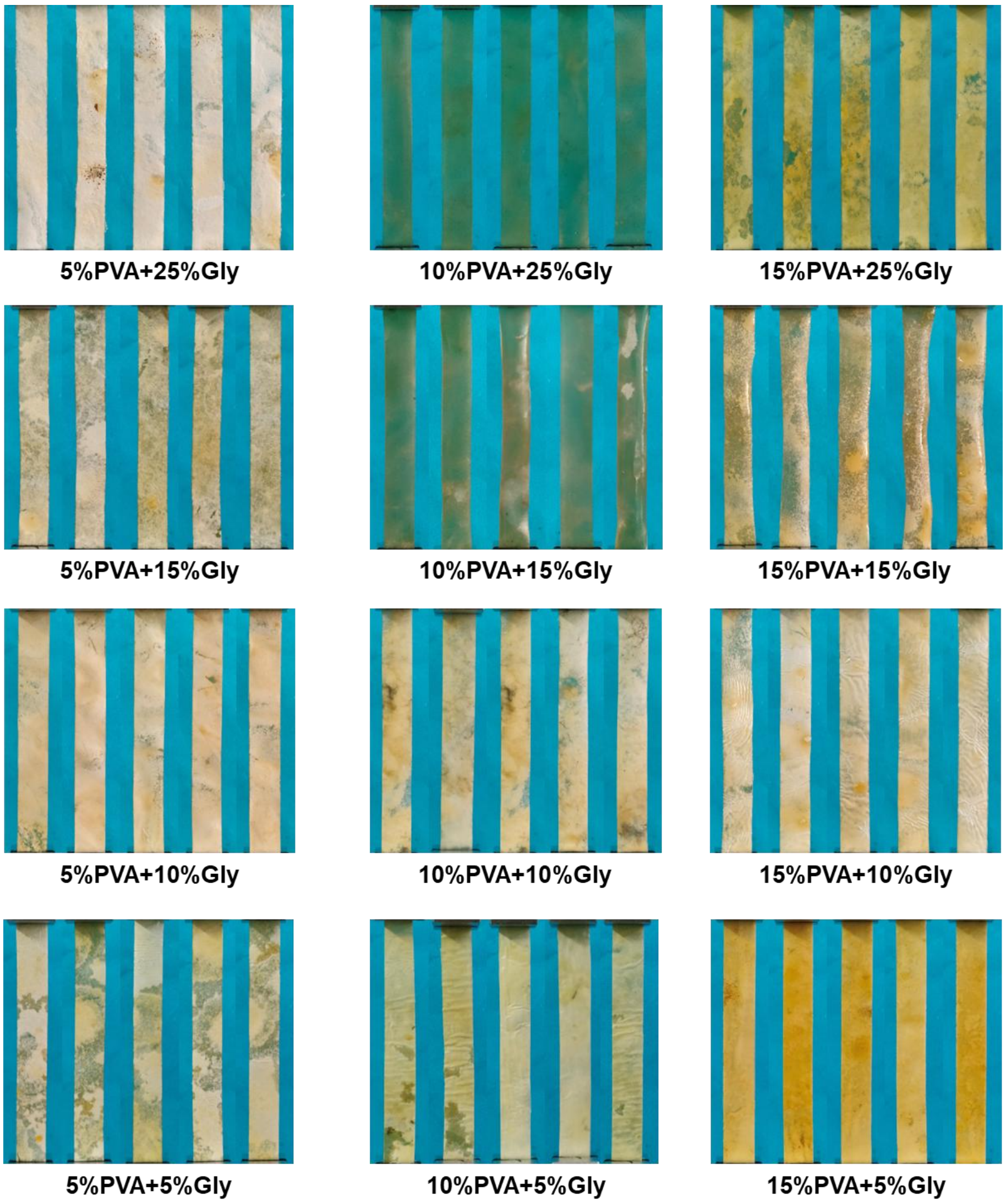

New Fig. S8 Optical images of mycelium macerate TV@P-G samples for tensile testing, n=5. Sample dimensions (width 10 mm, initial grip separation 80 mm, total length 100 mm).

We have changed contour maps of correlation between mechanical properties and treating agent contents into heat maps, according to our actual measurements. The revised Fig. 1e shows not artificially over-detailed results.

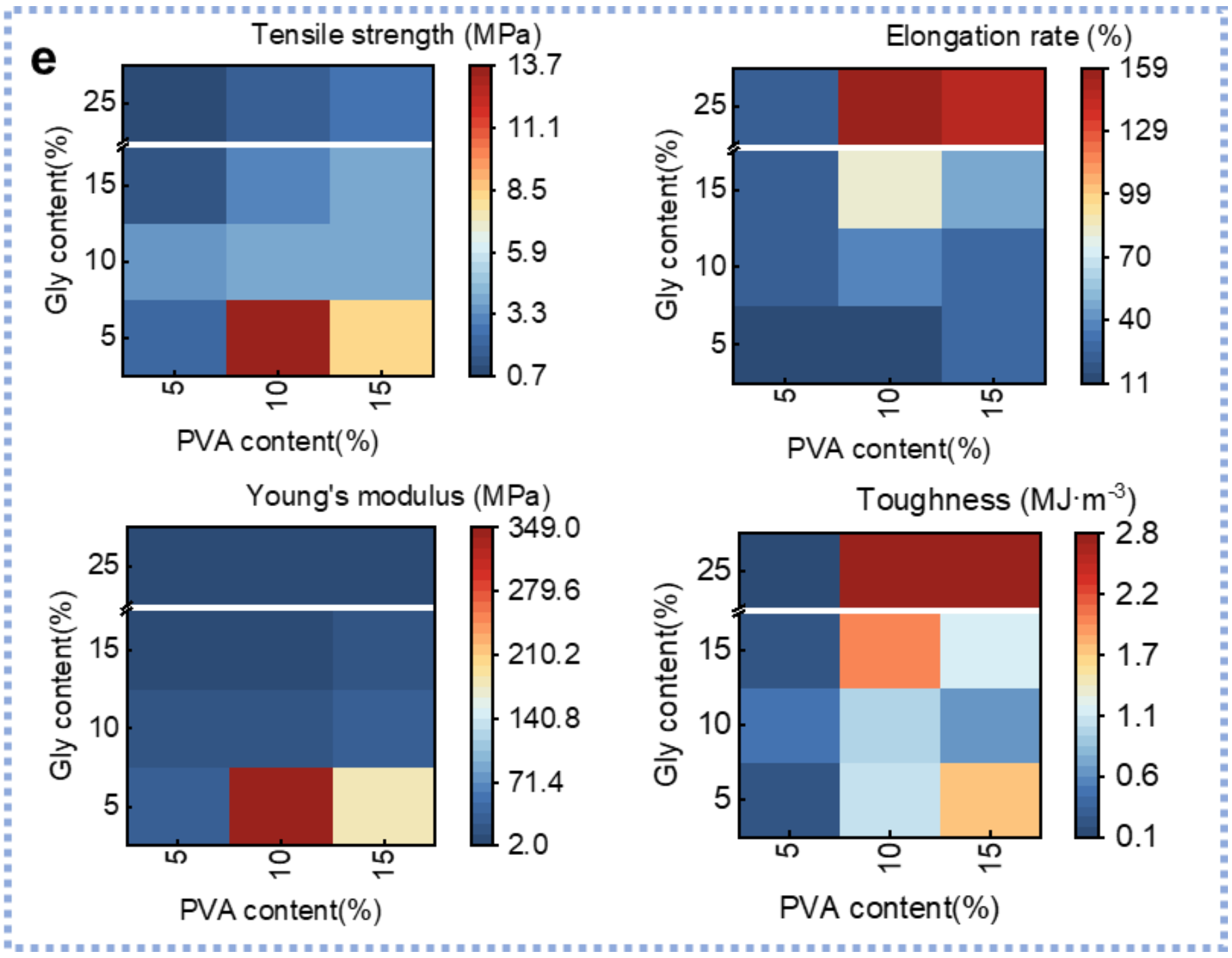

Revised Fig. 1e, Heat maps displaying correlation between mechanical properties (tensile strength, elongation rate, Young's modulus and toughness) and PVA, glycerol content.

For each mechanical parameter (tensile strength, elongation rate, Young's modulus and toughness), the mean value of each group was calculated based on n = 5 replicates. To quantify the tunability of our method, we define the tuning range as the ratio of the maximum mean value to the minimum mean value among the 12 groups. Pairwise comparisons between groups were performed using two-tailed independent t-tests (α = 0.05). The complete dataset, including individual data points, mean ± SD, and statistical significance markers, has been presented in new Figure S7 and new Table S2.

New Table S2 Mechanical properties of TV@P-G macerates

PVA content (%)	Glycerol content (%)	*E* (MPa)	*TS* (MPa)	*EG* (%)	*T* (MJ m^{-3})
5	5	25.8 ± 3.4	2.3 ± 0.2	14 ± 2	0.2 ± 0.1
5	10	18.5 ± 1.1	3.3 ± 0.1	25 ± 1	0.5 ± 0.1
5	15	5.9 ± 0.4	1.4 ± 0.2	24 ± 3	0.2 ± 0.1
5	25	2.8 ± 0.2	0.7 ± 0.0	24 ± 1	< 0.1
10	5	348.7 ± 78.6	13.7 ± 3.0	11 ± 4	1.1 ± 0.4
10	10	23.7 ± 6.0	4.1 ± 0.4	36 ± 8	1.0 ± 0.2
10	15	12.5 ± 1.7	3.3 ± 0.3	81 ± 35	2.0 ± 0.9
10	25	5.0 ± 0.3	1.8 ± 0.3	158 ± 58	2.3 ± 1.2
15	5	180.2 ± 26.4	8.3 ± 0.8	26 ± 16	1.5 ± 0.8
15	10	31.8 ± 2.6	3.7 ± 0.4	27 ± 8	0.7 ± 0.3
15	15	17.1 ± 1.8	3.7 ± 0.3	47 ± 8	1.2 ± 0.3
15	25	9.9 ± 0.2	2.4 ± 0.4	124 ± 61	2.3 ± 1.1

7. Demonstrations such as those in Fig. 2c are not meaningful in terms of the weight mentioned. The result can be easily manipulated because it is a poor experimental design. In general, the demonstrations are rather superficial and in the case of the sensors do not seem feasible. I suggest focusing on the bending for actuation and expanding the characterization (repeatability, performance over time, …) and design here, as this has the greatest potential.

Response: We sincerely thank the reviewer for this critical and constructive feedback. Accordingly, we have removed previous Fig. 2c and the related claims from the manuscript. Following the reviewer's helpful suggestion, we have tried to focus the manuscript on the bending actuation, which we agree has the greatest potential. Specifically, we have further conducted tests on the material's repeatability and performance over time (cyclic humidity response) to solidify the work. Regarding article writing, we have relocated the discussion of the regulatory mechanism governing material mechanical properties to the Supplementary Information. While the previous Section 2 of regarding mechanical properties modulation of mycelium macerates have been merged into the main text of the Section 1 on material preparation. Furthermore, in the Section 3 of the main text, we systematically investigated the mechanistic relationship between material moisture absorption and deformation. In addition, as suggested by the Question 3 of reviewer#3, we further supplemented a potential scenario in Section 4. Our samples can be made into deformation labels that detect prolonged high-humidity exposure in moisture-sensitive products, thus serving as a low-cost humidity history indicator that filters out short-term fluctuations.

Modification: Following the reviewers' suggestions, we removed the original Fig 2c. We also added cycling tests on hygroscopic deformation of TG@P-G macerate using humidity chamber, and the result was displayed in new Fig. S25, simultaneously suggested by the Question 4 of reviewer#1.

Page 6, lines 176-177: "The cyclic humidity response (Fig. S25) of mycelium macerate demonstrates that its deformation under high humidity conditions is reversible."

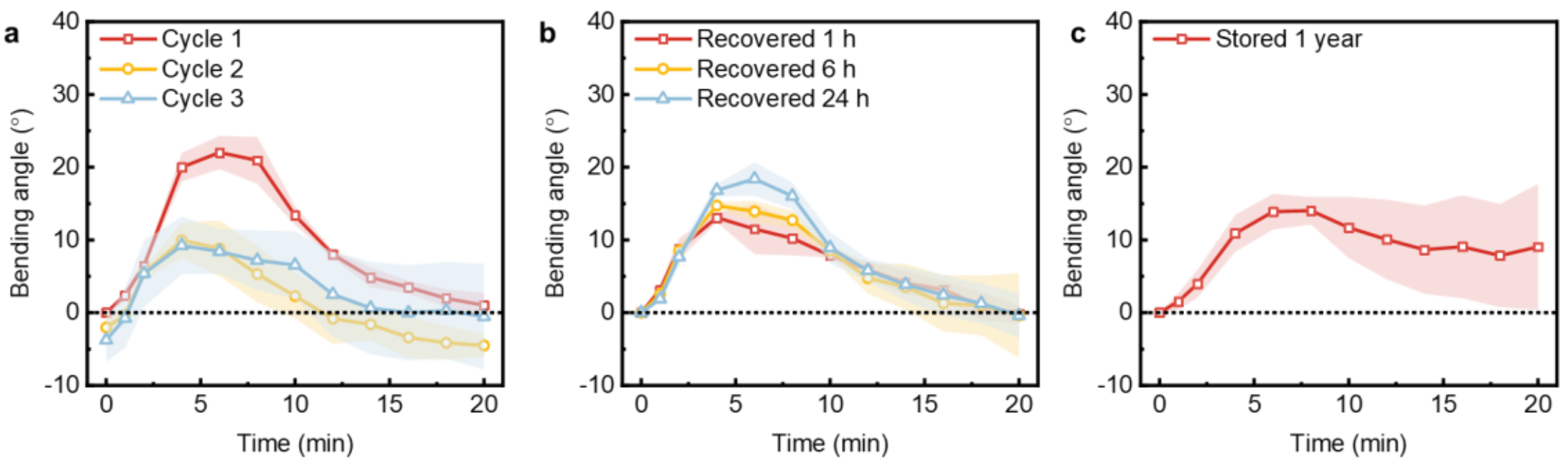

New Fig. S25 Cyclic bending angle changes of Janus TV@P-G (10%PVA+10%Gly) specimen (5 × 25 mm) in single fixed humidity response experiments, n=3. Humidity was controlled with humidity chamber. Error bars (mean ± SD) were connected and visualized as a shadow effect around the data points. Cycle 1 to 3 were continuous processes (**a**), while the "recovered cycles" were defined as the next cycle after 1-hour, 6-hour and 24-hour drying treatment to samples (**b**).

The clarification regarding sensing scenarios was added into revised main text on pages 11-12, lines 348-353 as follows.

"Unlike conventional humidity sensors that aim for fast response (seconds or less) to enable real-time monitoring, our device operates on a minute-scale timescale by design. This slow actuation and recovery provide an intrinsic low-pass filtering effect: short-term humidity spikes are effectively suppressed, while sustained high-humidity

events (minutes to hours) are integrated and retained as a measurable deformation. Accordingly, our approach is well-suited for applications requiring long-term exposure rather than real-time control.

8. Layer separation in general seems hard to do without damage. Address why your methods are reliable in this regard as it is not obvious.

Response: We thank the reviewer for highlighting the challenge of achieving damage-free layer separation. We acknowledge that complete separation of each layer is indeed challenging. For layer separation of dry mycelium macerate materials, the interface between layer A and B is evident. Moreover, the robustness of macerate ensured the integrity when peeling off each layer. To further avoid the impact from interface, we partially chose the surface and majority of layer A/B and excluded the layer around interface. We have supplemented our method in new Fig. S35a. As a result, we could separate layer A and B with less damage to compare their composition and hygroscopicity.

However, as the reviewer concerned, the fresh mycelium membrane was easily deformed and broken when separating with a scalpel, due to a water content of over 90%. Next, to address this problem, we employed a freezing microtome to peel off frozen mono layer from mycelium membrane embedded by optimal cutting temperature (OCT) compound, a mixture of polyethylene glycol (PEG) and PVA. Taking advantage of thickness setting function of microtome, combined with our SEM images of mycelium cross section, we could control peeled thickness from entire membrane. The step distance of samples was set as 50 μm. Similarly, to exclude the impact from interface, the interface part was peeled off together with mono layer A/B, while pure layer B/A was conserved, which is demonstrated as new Fig. S35b and c. Finally, after washing OCT compound from embedded remaining layer B/A, we then carried out ingredient testing on lipid, protein and polysaccharide.

Modification: Layer separation methods of mycelium macerate and fresh mycelium membrane were added in page 8, lines 208-215, of Supplementary Information. Meanwhile, the illustration diagram and experimental photograph were displayed as new Fig. S35.

"We used a freezing microtome (Leica CM1950) to peel off a frozen monolayer from the mycelium membrane, which had been embedded in optimal cutting temperature (OCT) compound, (Tissue-Tek®), a mixture of polyethylene glycol (PEG) and PVA. By utilizing the microtome's thickness adjustment function and referencing our SEM images of mycelium cross-sections, we were able to control the thickness of the peeled layer from the entire membrane. The step distance for sampling was set to 50 μm. To eliminate interfacial effects, the interface region was peeled off together with monolayers A and B, while the pure layers B/A were preserved, as shown in the new Fig. S35b and c. After removing the OCT compound from the remaining embedded B/A layer, we conducted ingredient testing for lipids, proteins, and polysaccharides."

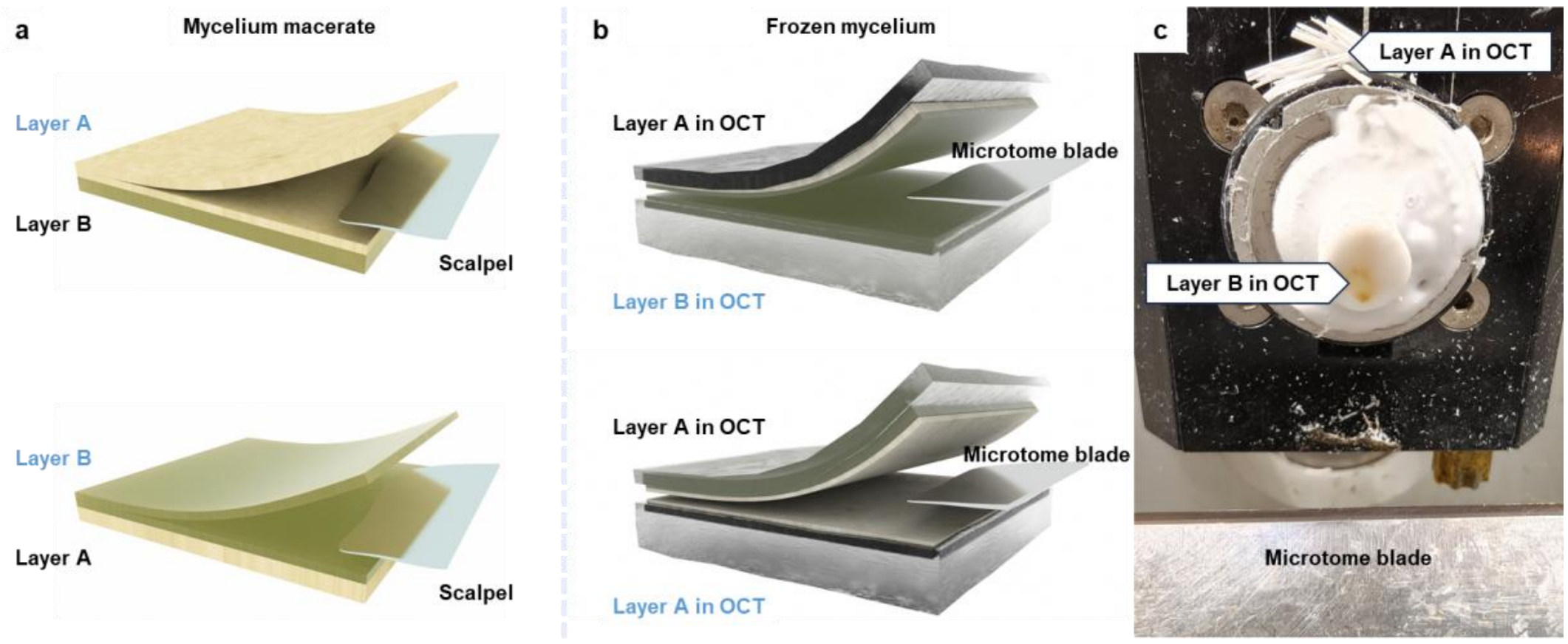

New Fig. S35 Layer separation methods. **a**, **b**, Schematic illustration for layer separation of mycelium macerate (**a**) and frozen (fresh) mycelium membrane (**b**), respectively. **c**, Optical image of peeling off layer A using a freezing microtome.

Response to the reviewers' comments (Reviewer#3)

Comments to the Author:

Nie and co-workers report the development of a mechanically robust, humidity-responsive, and biologically viable "living mycelium macerate" based on liquid-state fermented fungal membranes engineered through PVA–glycerol maceration. By exploiting the intrinsic Janus architecture of aerial and nutritive hyphal layers, the authors achieve asymmetric hygromechanical behavior that enables programmable actuation and humidity-to-electrical signal transduction. The work further demonstrates long-term dormancy, regeneration, and inheritance of structural and functional properties across multiple growth generations.

The study integrates multiscale characterization (SEM, AFM, micro-CT, SAXS, MIP), computational modeling (DFT and MD), mechanical testing, and in vitro assays to establish a comprehensive structure–property–function relationship. The reported elongation and toughness values are among the highest for flexible mycelium-based materials, and the concept of a regenerative, inheritable "dormant living actuator" is novel and conceptually impactful.

Overall, the manuscript is ambitious, interdisciplinary, and largely well executed. Therefore, I recommend the publication of this work in *Nature Communications*, provided the following comments are addressed:

Response: We are very grateful to the Reviewer#3 for the high recognition and affirmation of our work, as well as valuable suggestions. We have addressed all comments as detailed below. In the revised document, changes are highlighted in yellow for your convenience.

1. The main concern is about the reproducibility of mechanical properties. The authors have repeatedly claimed "record-breaking" mechanical performance among flexible mycelium materials. However, limited information about the mechanical tests have been provided, especially the sample sizes (n) and error bars that should be consistently emphasized in all mechanical and hygroscopic performance plots. The reproducibility of the performance is crucial for this kind of un-conventional living materials.

Response: We thank the reviewer for raising this important point regarding the reproducibility of mechanical properties. To address this concern, we have 1) added the sample size (n) and standard deviation to all relevant figures (mechanical and hygroscopic performance plots); 2) revised the main text to explicitly state the number of independent replicates and to clarify that error bars represent mean ± SD. More detailed information about sample geometries, error bars, environmental conditions have been added in each figure caption and method description.

Modification: According to your professional suggestions, we added detailed information about the mechanical tests in both the main text (page 15, lines 428-429) and Supplementary Information (page 2, lines 82-85).

Main text: "Insets, stress-strain curves of regrowth mycelium macerates (rectangular specimen with a width of 10 mm, a total length of 100 mm, and initial grip separation of 80 mm) through different regeneration pathways, and optical images of Janus surfaces of regenerated mycelium macerate."

Supplementary Information: "Following ASTM D882-18 standard, sample dimensions were normalized in width of

10 mm, initial grip separation of 80 mm, total length of 100 mm. These rectangular shape specimens were prepared by a mold cutter… The test speed was set as 5 mm/min, and environmental conditions stayed in 23 ± 2°C, 30 ± 5% RH."

Error bars were described in each figure caption.

Main text:

Fig. 2 …Error bars (mean ± SD) in **b** and **d** were connected and visualized as a shadow effect around the data points.

Fig. 3b, Moisture absorption kinetics curves of layer A and B, n = 3. Error bars were connected and visualized as a shadow effect around the data points.

Part of Supplementary Information:

Fig. S6 Correlation between Janus structure and cultivation time… **c**, **d**, Thickness changes (**c**) and proportion changes of layer A/B in total thickness (**d**), n = 10. Data are expressed as mean ± standard deviation (SD).

Fig. S7 Tensile stress-strain curves of TV@P-G treated with 5-15% PVA and 5-25% glycerol, n=5. Error bars were connected using B-spline basis function and visualized as a shadow effect around the data points.

Fig. S9 Thickness of TV@P-G macerates treated with different PVA and glycerol contents. Each group of experiments was repeated 10 times (n = 10). Data are expressed as mean ± standard deviation (SD).

Fig. S10 Statistically analyze the influence of different PVA and glycerol contents on the mechanical performance parameters (TS, EG, E and T) of TV@P-G macerate, and conduct screening by comprehensively considering the mechanical performance and material input. Each group of experiments was repeated 5 times (n = 5). Data are expressed as mean ± standard deviation (SD). A two-tailed t-test was used to compare similar data.

Fig. S19 Sorption kinetics curves of control groups, TV, TV 10%PVA, TV 10%Gly, n=3. Error bars (mean ± SD) were connected and visualized as a shadow effect around the data points.

Fig. S28 Statistics for comparison of expansion ratios of separated layer A and B using optical images of hygroscopic expansion, n=10. Data are expressed as mean ± standard deviation (SD). A two-tailed t-test was used to compare similar data. For t-tests: Cohen's d was calculated as ($mean_1$ - $mean_2$) / pooled standard deviation. Difference in area expansion ratio: p = 0.243, Cohen' d = 0.57. Difference in volume expansion ratio: p = 0.247 Cohen' d = 0.56.

Fig. S36b DVS curves of TV@P-G, separated layer A and B, n=3. Error bars (mean ± SD) were connected and visualized as a shadow effect around the data points.

Fig. S40 Percentage composition of PVA, Glycerol and biomass in TV@P-G fabricated through treatment with 0-25% glycerol, n=3. Error bars represented mean ± SD.

Fig. S41 Percentage composition of PVA, Glycerol and biomass in layer A and B (dried) of TV@P-G fabricated through treatment with 5-25% glycerol, n=3. Error bars represented mean ± SD.

Fig. S43 Weight reduction rates of separated layer A and B after dextranase treatment, n=3. Error bars represented mean ± SD. Residual biomass was corresponding to chitin…

Fig. S66 Determination of DPPH free radical clearance of native mycelium membrane and dormant macerate material, n = 3. Error bars represented mean ± SD.

Fig. S83 Soil degradation and weight reduction curve of TV@P-G, n = 3. After 100 days, mycelium macerates broke down into fragments until completely degraded impracticable to weigh. Error bars represented mean ± SD, were connected and visualized as a shadow effect around the data points.

2. Although the development is based on living materials, PVA, a non-degradable, non-biobased polymer has been used as the main network enhancer to improve the mechanical properties. The authors may clarify the end-of-life scenario for PVA-containing macerates in real-world deployment, and consider potential bio-based or biodegradable alternatives to PVA and their expected impact on mechanical and hygroscopic performance. In addition, it is not surprising to see the introduction of

strong polymer chains bringing hydrogen bonding can improve the mechanical properties of the pristine materials.

Response: Thank you for your constructive questions and suggestions. We followed the reviewer's comment by clarifying the end-of-life scenario for PVA-containing macerates. Based on life cycle assessment (LCA), we calculated the energy consumption and carbon emissions (GWP indicators of 7.63 kg CO_2-eq·kg^{-1}) during the material production process, and proposed measures for energy conservation and emission reduction. We also attempted to separate and recycle PVA from used macerate in the laboratory for secondary use, proving that the sustainability of our mycelium macerate can be optimized.

Although we had attempted to employed bio-based polymer, including carboxymethyl cellulose; polysaccharides from *Trametes,* pullulan, and pectin as alternatives to PVA, the tensile strength (0.3 ~ 0.6 MPa) and toughness (0.8 MJ·m^{-3}) of these polysaccharides treated mycelium macerates were far lower than PVA treated groups, performed in Fig S74 (previous Fig. S12). We supposed that the main chain of PVA consists of C-C bonds, while the side chains contain a large number of free hydroxyl groups, in addition, the PVA molecules are highly flexible, which can form strong hydrogen bonds with biological macromolecules of fungi cell wall. In contrast, the main chain of polysaccharides consists of rigid glucose ring. Although there are also a large number of hydroxyl groups, the spatial structure of the sugar molecules has greater steric hindrance than that of PVA, and the molecular chains are more rigid, making it difficult for them to form strong interactions with the substrate sufficiently. On the other hand, as the plasticizer, the small molecule, glycerol has difficulty establishing strong bridges of interaction between polysaccharides. Therefore, we also chose decaglycerol (DG) as an alternative plasticizer to glycerol, when PVA had been replaced by pullulan, considering that DG in short chains formed denser hydrogen bond and physical entanglement networks than glycerol.

Based on pullulan and decaglycerol treatment, we fabricated TV 10%Pul+10%DG macerate with Janus structure, and compared its mechanical performance and hygroscopic deformation behavior with PVA and glycerol treated groups. Different from TV@P-G macerates, TV 10%Pul+10%DG macerate performed as brittle material. Here, our experiment was restricted by the long incubation period of fungi and failed to set up multiple experimental groups of to fully regulate the mechanical properties of TV@Pul-DG. Moreover, the hygroscopic deformation phenomenon of this material shows a direction opposite to that of the TV@PVA-Gly group, and the bending angle is not obvious. We believe that this is due to the different distribution of Pul and DG molecules in the native Janus mycelium membrane compared to PVA and Gly, as well as the relatively high Young's modulus (117.0 MPa) of the material which limited deformation.

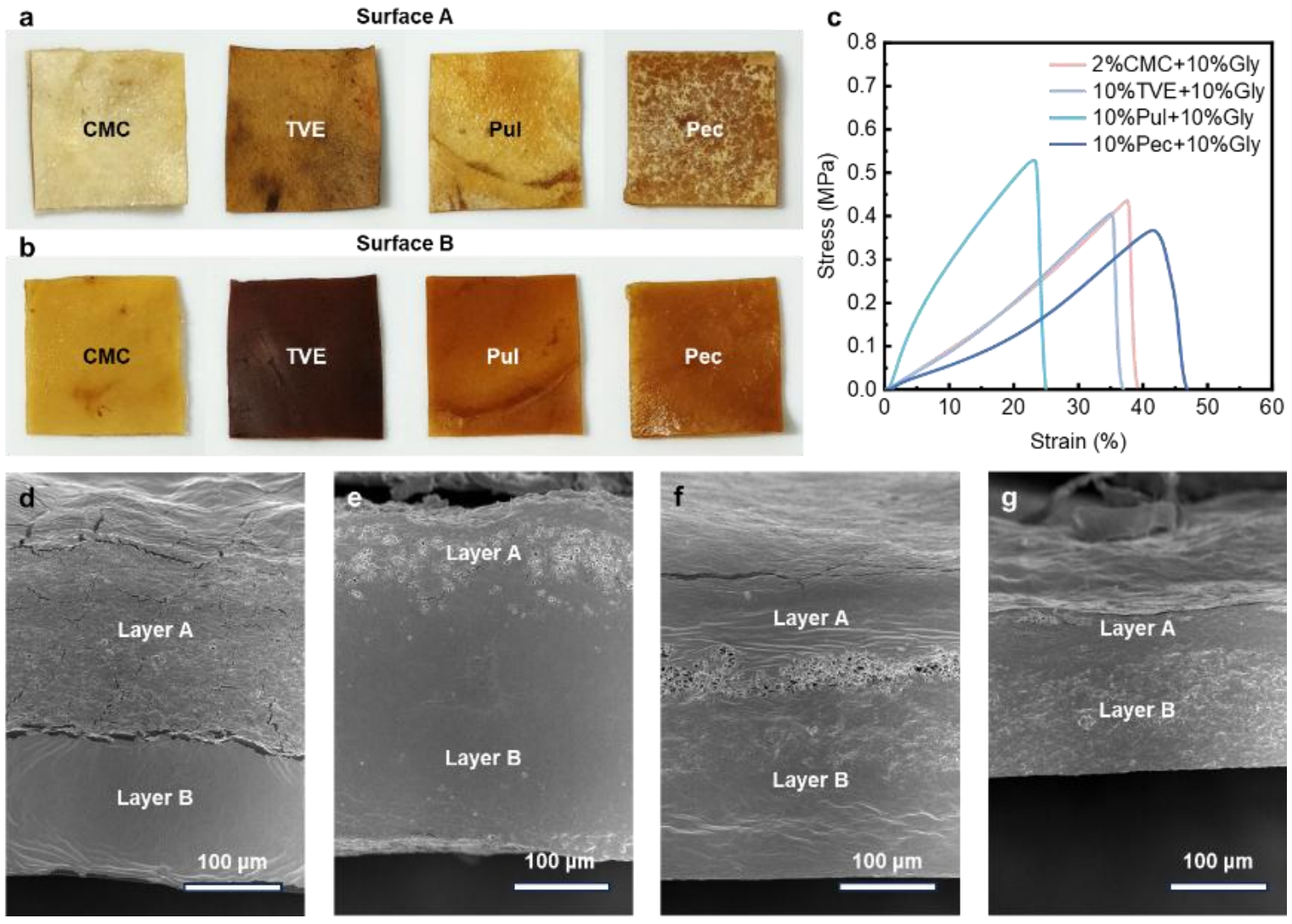

Fig. S74 Mycelium macerates bonded with polysaccharides. a, b, Optical images of Janus mycelium macerates TV@PSac-G viewed from surface A (**a**) and B (**b**). **c**, Tensile stress-strain curves of Trametes versicolor membrane treated with polysaccharides, and 10% glycerol, n=3. CMC: Carboxymethyl cellulose; TVE: polysaccharides from Trametes; Pul: pullulan; Pec: pectin. **d-g**, SEM images of TV@PSac-G cross section. TV@CMC-G, (**d**); TV@TVE-G, (**e**); TV@Pul-G, (**f**); TV@Pec-G, (**g**).

Modification: According to your professional suggestions and our experiment results, we have first added a cradle-to-gate LCA of mycelium macerate. We added the conclusion of LCA in revised main text (page 15, lines 431-433).

"…Additionally, life cycle assessment demonstrates that the carbon footprint of PVA-modified mycelium materials could be reduced through optimized PVA loading and bio-based alternatives for sustainability."

And we supplemented the detailed method (pages 14-17, lines 443-462) and analysis (page 63, lines 763-780) in Supplementary Information.

"…This study adheres to the framework of ISO 14040/14044 standards to perform a cradle-to-gate life cycle assessment (LCA) of the material production process. The assessment was conducted using SimaPro software, which is linked to background databases including Ecoinvent. The IPCC 2021 GWP100 methodology was employed to specifically evaluate the global warming potential (GWP) of the product over a 100-year time horizon, with results reported in units of 'kilograms of carbon dioxide equivalent (kg CO_2-eq)'. The functional unit was defined as 'the production of 1 kg of the sample'. The assessment scope encompassed all processes from raw material acquisition and energy production to the point of product dispatch (the 'gate'). This included the manufacturing of key raw materials (e.g., water, malt extract, glycerol, PVA), electricity consumption, and direct CO_2 emissions, but excluded post-dispatch stages such as transportation, usage, and end-of-life disposal. The model did not explicitly incorporate the construction and decommissioning of infrastructure (e.g., factory buildings and equipment) as capital goods, nor did it account for associated long-term emissions. A systematic analysis of the inventory analysis and impact assessment results was conducted to identify carbon emission hotspots within the process, thereby providing a scientific basis for subsequent process optimization and environmental decision-making."

Flowchart of production stages for mycelium macerate was shown as new Fig. S71. And the GWP indicators results was displayed in radar chart, new Fig. S72

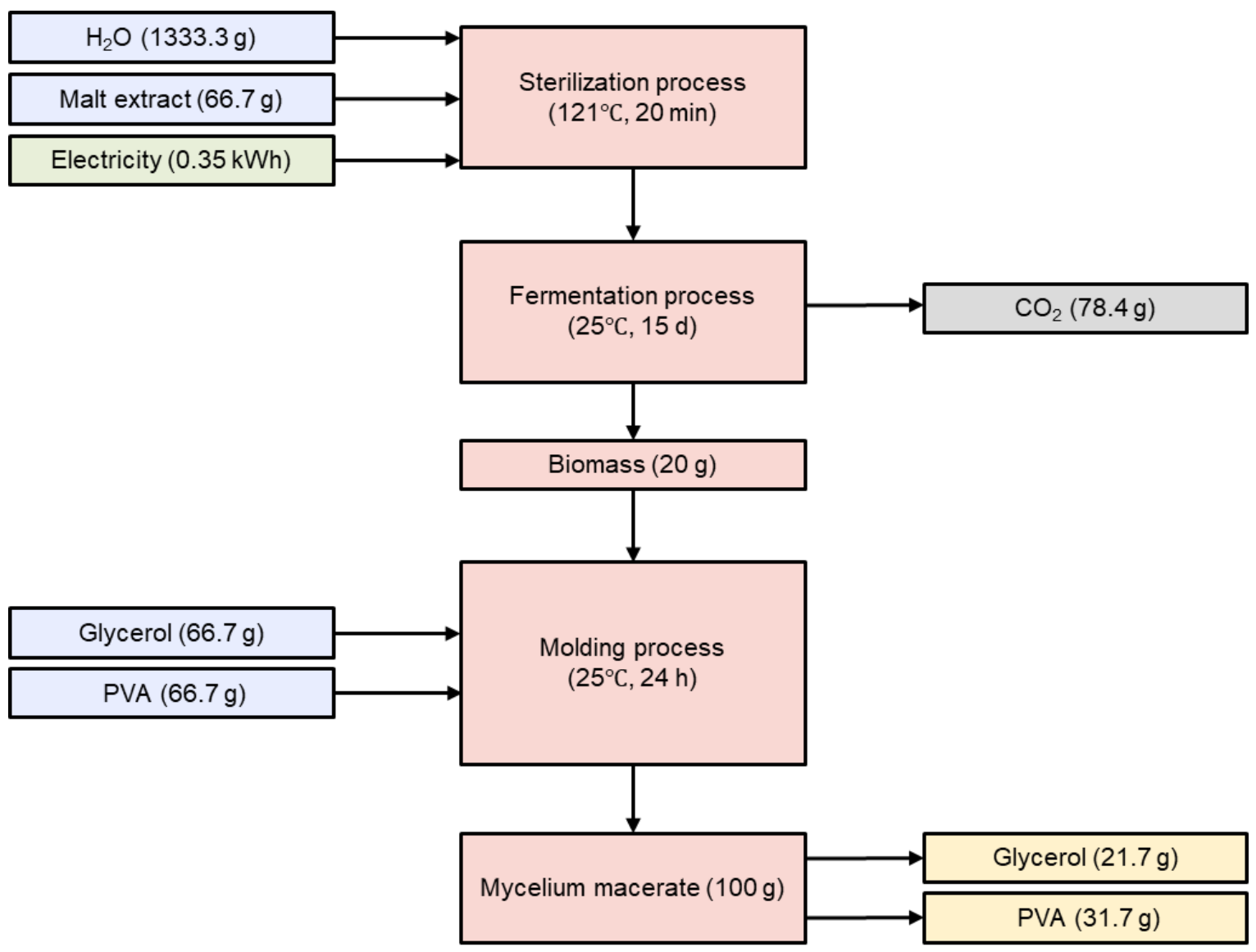

New Fig. S71 Flowchart of production stages for mycelium macerate (10%PVA+10%Gly). The quantities of raw materials and energy consumption in the flowchart are 10 times that of the experimental quantities.

"According to the GWP assessment outcomes, the total GWP associated with producing 1 kg of the product is 7.63 kg CO_2-eq. Notably, electricity consumption emerges as the largest source of carbon emissions, contributing 2.59 kg CO_2-eq, which accounts for 33.9% of total emissions. This underscores that the carbon intensity of electricity utilized in the production process is a critical driving factor. Optimizing process parameters to reduce energy consumption or transitioning to renewable electricity sources represents the most effective strategy for mitigating the carbon footprint of this process. Second, the LCA-PVA process (as a raw material input) contributes 1.88 kg CO_2-eq, representing 24.6% of total emissions and ranking as the second-largest emission source. PVA production itself is characterized by high energy demand (requiring 1.07 kWh of electricity and 5 kg of steam for 1 kg PVA) and direct CO_2 emissions (2.5 kg). Thus, selecting PVA alternatives or suppliers with lower environmental impacts is pivotal for reducing the overall carbon footprint. Third, glycerol, wheat flour, and direct CO_2 emissions from the process (784 g) contribute approximately 18.88%, 11.78%, and 10.28% of total emissions, respectively, while the environmental impact of water is negligible. More importantly, by taking advantage of the ability of fungi as natural decomectors, the culture medium can be replaced from high-carbon-footprint nutritional products to sugar-containing organic waste. For instance, in our laboratory, discarded mycelium was crushed and sterilized to produce culture medium for cultivating new mycelium, achieving the reuse of high-carbon-footprint materials and reducing the energy consumption in the preparation process of new batches of mycelium materials."

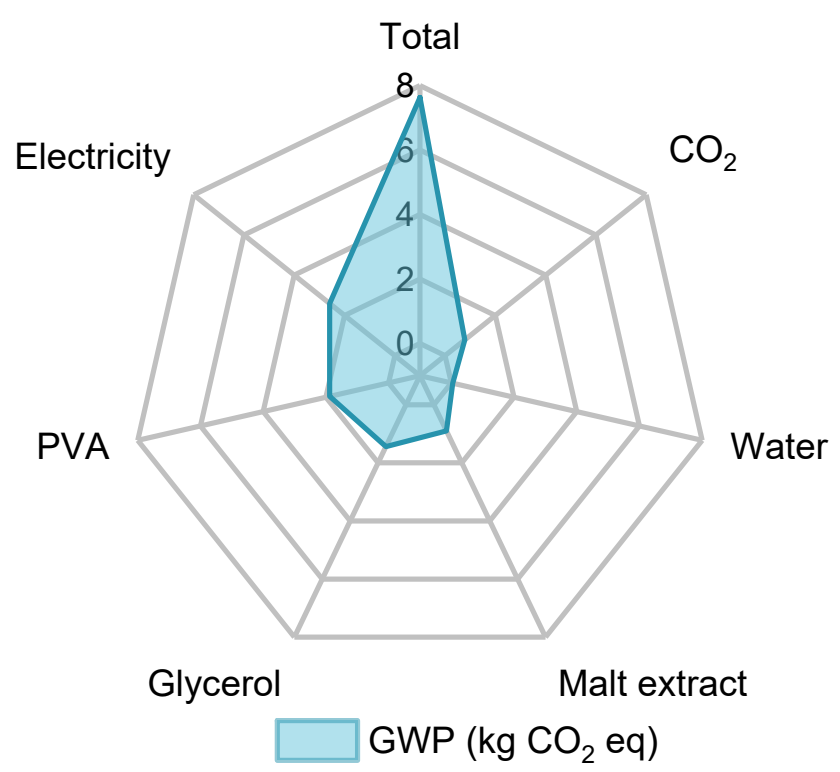

New Fig. S72 Contribution of different raw materials and energy consumption to GWP indicators of mycelium macerate (10%PVA+10%Gly).

The GWP data of mycelium macerate we modeled was calculated based on the raw material input in our experiment, the power of the laboratory instruments, and the separate modeling for PVA, displayed as new Table S7. Data required for modeling GWP of PVA was summarized in new Table S8-10 and calculated according to the process from VAc, PVAc to PVA. Last, we offered the calculation results of GWP for 1.0 kg mycelium macerate in new Table S11.

New Table S7 LCA inventory for 100 g mycelium macerate (10%PVA+10%Gly).

Items	Corresponding LCI	Amount	Unit
	Mycelium macerate (10%PVA+10%Gly)		
Sample	Products	100	g
	Materials/fuels		
Water	Water, decarbonised {GLO}| diethyl ether production | Cut-off, U	1333.3	g
Malt extract	Wheat flour {RoW}| market for wheat flour | Cut-off, U	96.7	g
Glycerine	Glycerine {RoW}| market for glycerine | Cut-off, U	44.9	g
PVA	Self-mode	34.9	g
	Electricity/heat		
Electricity	Electricity, low voltage {GLO}| market group for electricity, low voltage | Cut-off, U	0.35	kWh
	Emissions to air		
CO_2	Carbon dioxide	78.4	g

New Table S8 LCA inventory for 1.0 kg PVA production

Items	Corresponding LCI	Amount	Unit
	PVA		
PVA	Products	1	kg
	Materials/fuels		
VAc	Vinyl acetate {GLO}| market for vinyl acetate | Cut-off, U	1.65	kg
Methanol	Methanol {RoW}| market for methanol | Cut-off, U	0.45	kg
NaOH	Sodium hydroxide, without water, in 50% solution state {RoW}| market for sodium hydroxide, without water, in 50% solution state | Cut-off, U	0.08	kg
Water	Tap water {GLO}| market group for tap water | Cut-off, U	10	kg
	Electricity/heat		
Electricity	Electricity, low voltage {GLO}| market group for electricity, low voltage | Cut-off, U	1.07	kWh
Steam	Steam, in chemical industry {GLO}| smelting and refining of nickel concentrate, 16% Ni | Cut-off, U	5	kg
	Emissions to air		
CO_2	Carbon dioxide	2.5	kg

Continued new Table S8	Emissions to water		
COD	COD (Chemical Oxygen Demand), CN	120	g
	Waste to treatment		
Wastewater	Wastewater, average {RoW}| clinker production | Cut-off, U	5.1	m^3
MSW	Municipal solid waste {GLO}| treatment of municipal solid waste, unsanitary landfill, wet infiltration class (500mm) | Cut-off, U	0.15	kg

New Table S9 LCA inventory for 1.0 kg PVAc production

Items	Corresponding LCI	Amount	Unit
	PVAc		
PVAc	Products	1	kg
	Materials/fuels		
VAc	Vinyl acetate {GLO}| market for vinyl acetate | Cut-off, U	1.03	kg
Nitrogen	Nitrogen, liquid {RER}| Cut-off, U	0.00125	kg
Compressed air	compressed air, average installation, >30kW, 8 bar gauge, at supplynetwork {RER}| | Cut-off, U	1.73	m^3
Water	Tap water {GLO}| market group for tap water | Cut-off, U	2.48	kg
	Electricity/heat		
Electricity	Electricity, low voltage {GLO}| market group for electricity, low voltage | Cut-off, U	0.376	kWh
Steam	Steam, in chemical industry {GLO}| smelting and refining of nickel concentrate, 16% Ni | Cut-off, U	1.40	kg
Natural gas		0.885	MJ
	Emissions to air		
Heat	Heat waste | Cut-off, U	1.36	MJ

New Table S10 LCA inventory for 1.0 kg VAc production

Items	Corresponding LCI	Amount	Unit
	VAc		
VAc	Products	1	kg
	Materials/fuels		
Ethylene	Ethylene {RoW}| market for ethylene | Cut-off, U	0.334	kg
Acetic acid	Acetic acid, without water, in 98% solution state {GLO}| market for acetic acid, without water, in 98% solution state | Cut-off, U	0.705	kg
Oxygen	Oxygen, liquid {RoW}I market for oxygen, liquid | Cut-off, U	0.196	kg
Water	Tap water {GLO}| market group for tap water | Cut-off, U	2.48	kg
	Electricity/heat		
Electricity	Electricity, medium voltage {AU}| market for electricity, medium voltage I Cut-off, U	1.65	kWh
Natural gas	Heat, district or industrial, natural gas {RoW}I market for heat, district orindustrial, natural gas |Cut-off, U	1.856	MJ

**Continued new Table S10**	Emissions to air		
Acetic acid		0.00141	kg
Carbon dioxide		0.0623	kg
Ethylene		0.00067	kg
	Emissions to water		
BOD5	BOD5 (Bioloaical Oxygen Demand)	0.0696	kg
Acetic acid		0.000564	kg

New Table S11 The calculation results of GWP for 1.0 kg mycelium macerate based on IPCC 2021

Num	Damage category	GWP100
Unit		kg CO_2-eq
Total		7.63
CO_2	CO_2	0.784
Water	Water, decarbonised {GLO}| diethyl ether production | Cut-off, U	0.046
Malt extract	Wheat flour {RoW}| market for wheat flour | Cut-off, U	0.899
Glycerine	Glycerine {RoW}| market for glycerine | Cut-off, U	1.44
PVA	PVA	1.88
Electricity	Electricity, low voltage {GLO}| market group for electricity, low voltage | Cut-off, U	2.59

Then we considered the end-of-life scenario of mycelium macerate, where PVA could be recycled based on the method we used for quantifying PVA content in mycelium macerate. Flowchart of recycling stages for mycelium macerate was added as new Fig. S73.

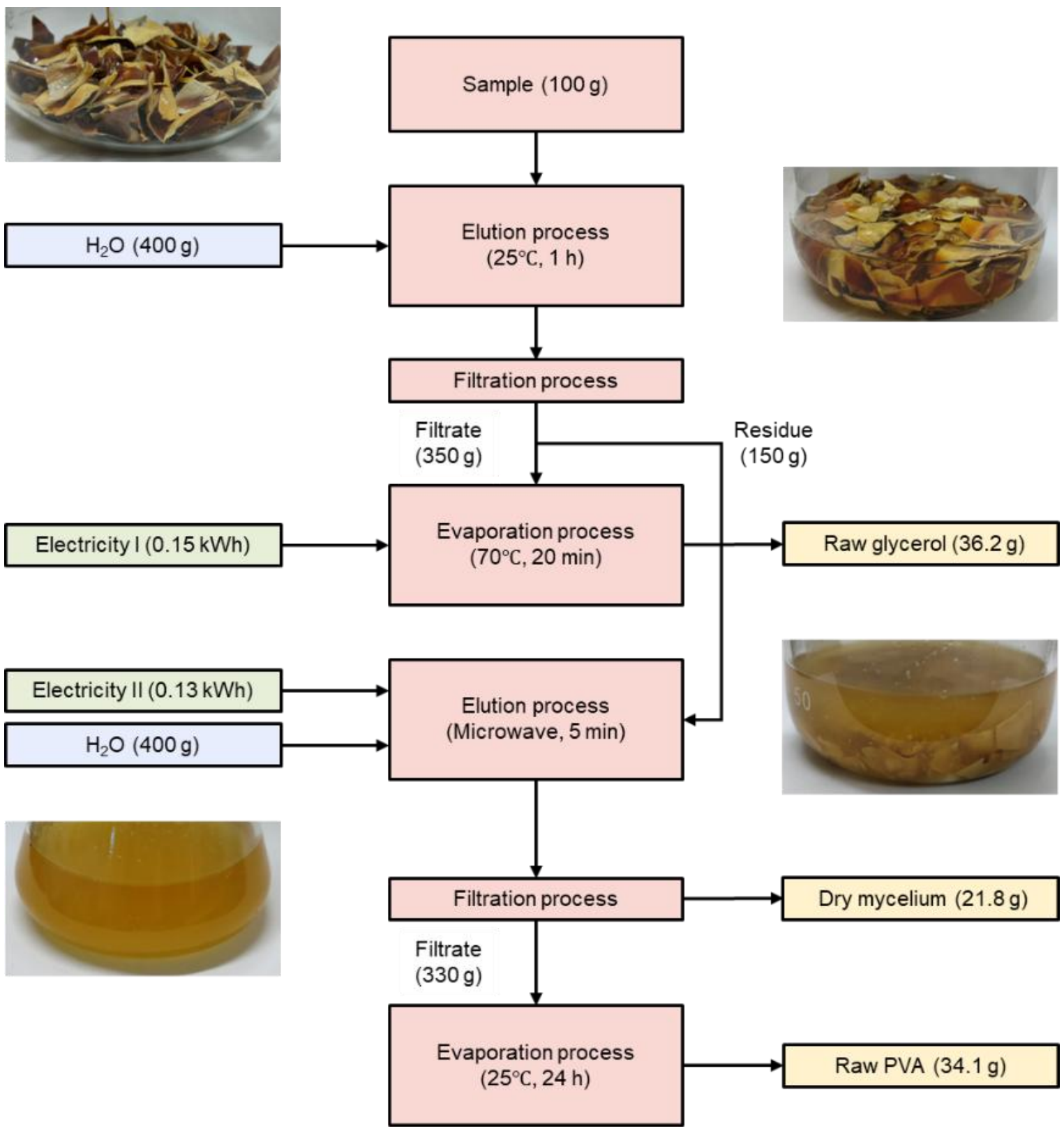

New Fig. S73 Flowchart of recycling stages for mycelium macerate (10%PVA+10%Gly). The quantities of materials and energy consumption in the flowchart are 10 times that of the experimental quantities.

We added mechanical and hygroscopic performance of all-bio-based mycelium macerate in new Fig. S75. And the corresponding conclusion was placed in Discussion section, page 15, lines 433-438.

"…For instance, natural polysaccharides (PSac), including carboxymethyl cellulose, polysaccharides from *Trametes*, pullulan and pectin could be adopted to replace PVA as bonding agent yet with a compromised mechanical performance (Fig. S74 and 75). Adoption of bio-based alternatives to PVA still demand our further exploration on flexibly customizing the mechanical properties and responsiveness of mycelium materials."

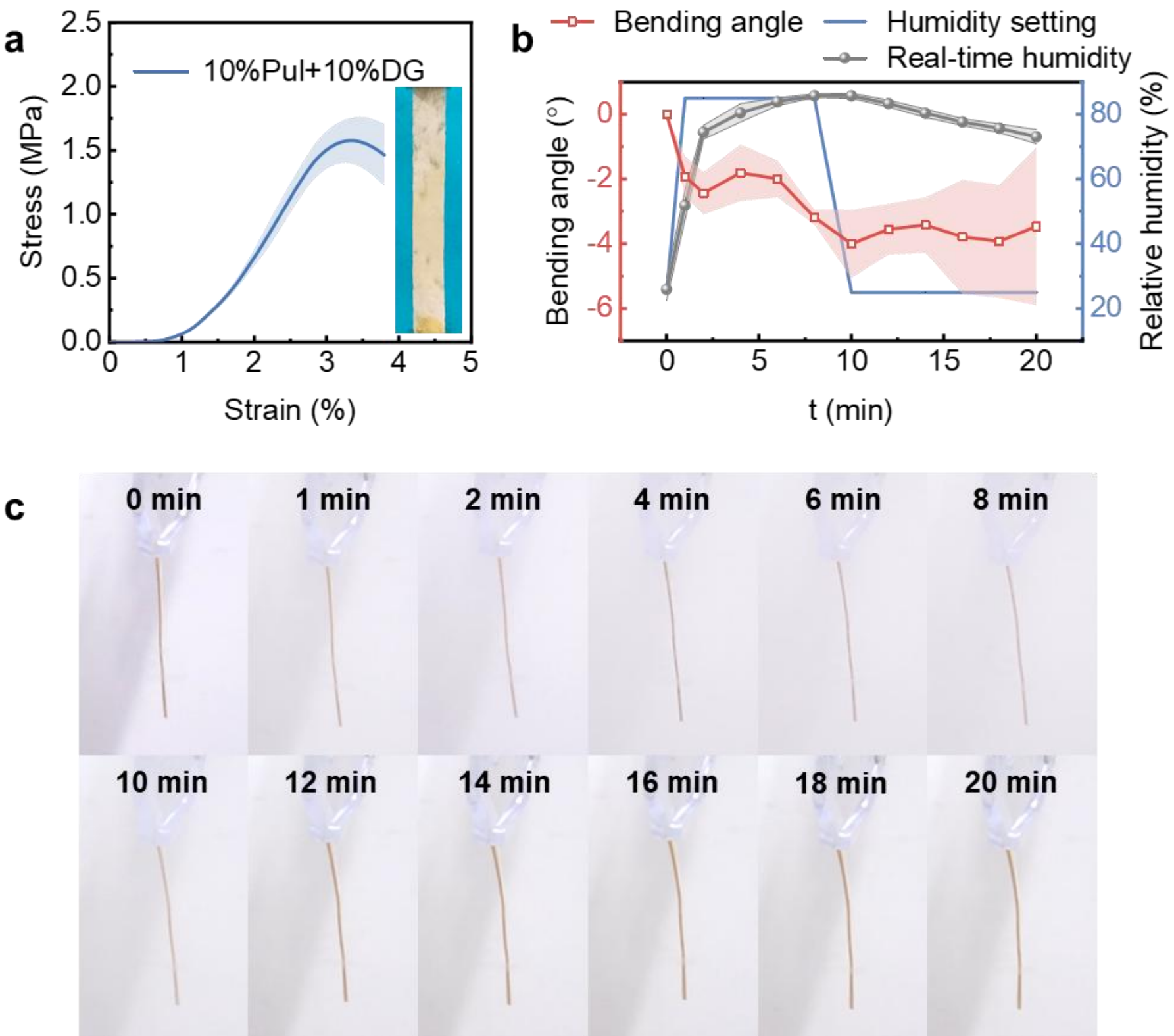

New Fig. S75 Mechanicl properties and hygroscopic deformation of all-biobased TV@Pul-DG macerate. **a**, Tensile curve and image of TV 10%Pul+10%DG specimen. **b**, Bending angle changes of TV@Pul-DG specimen (5 × 25 mm) in single fixed humidity response experiments, n=3. Humidity was controlled with humidity chamber. Error bars were connected and visualized as a shadow effect around the data points. **c**, Optical images of TV@Pul-DG specimen (5×25 mm) with single fixed end deforming in hygroscopic process controlled in a humidity chamber, where humidity changes was set and recorded as (**b**).

3. The demonstrated application for actuation and sensing operate on timescales of minutes, which limits applicability for many real-time sensing or robotic control scenarios. Any comments on this?

Response: Many thanks for the instructive comment. We have explained and supplemented the [Super stable moisture-responsive actuator via covalent crosslinking for efficient personal thermal management. *Adv. Mater.* **37**, e07267 (2025)] and designed an application scenario for slow-response actuators.

Humidity history indicator: Our samples can be made into irreversible or semi-reversible deformation labels, which can be used to detect whether products prone to moisture (such as medicines, precision instruments, and high-end foods) have been exposed to a long-term high-humidity environment during transportation or storage. By taking advantage of the characteristic that the material deforms due to moisture absorption and then recovers slowly, the material will only fully bend when the humidity exceeds the threshold and lasts for 5 min, serving as a warning sign

indicating "moisture absorption"; and even if the environment becomes dry again, the macerate will still remain deformed for at least 30 min, functioning as a “humidity history indicator”. This slow-response material can filter out short-term humidity fluctuations and truly reflect the long-term storage conditions.

Modification: According to your professional suggestions, some deletion and more detailed clarification related with sensing scenarios were added into revised main text on pages 11-12, lines 348-353 as follows.

“Unlike conventional humidity sensors that aim for fast response (seconds or less) to enable real-time monitoring, our device operates on a minute-scale timescale by design. This slow actuation and recovery provide an intrinsic low-pass filtering effect: short-term humidity spikes are effectively suppressed, while sustained high-humidity events (minutes to hours) are integrated and retained as a measurable deformation. Accordingly, our approach is well-suited for applications requiring long-term exposure logging rather than real-time control.”
