# Supplementary material for: Tailored living mycelium macerate for reproducible flexible actuators
Source: Nat Commun. 2026 Aug 12;17:9682. doi: 10.1038/s41467-026-76623-z (PMC13558639; doi:10.1038/s41467-026-76623-z)
Supplement: Supplementary file 4 [file 41467_2026_76623_MOESM2_ESM.docx]

**Description of Additional Supplementary Files**

**Supplementary Information**

**Description:** Supplementary tables, figures, supplementary text of discussion, and supplementary references are provided.

**Supplementary Movie 1** Hygroscopic deformation of mycelium macerates triggered by unilateral humidification

**Description:** This video verifies heterogeneous deformation of mycelium macerate’s Janus layers upon exposure to high humidity gradient, where layer A exhibits a more pronounced response.

**Supplementary Movie 2** Application of mycelium macerate in circuit control and feedback regulation

**Description:** This video records the entire process of myce strain gauge mediated humidity regulation from humidity increasing to control circuit switched on, and end with feedback regulation and humidity decreasing.

**Supplementary Movie 3** Resistance monitoring accompanied with hygroscopic deformation

**Description:** This video collected along with resistance monitoring by source meter unit reflects the synchronism of hygroscopic deformation and resistance fluctuation.

**Supplementary Data 1** Computational models

**Description:** Atomic coordinates of the optimized computational models and molecular dynamics trajectories at the initial and final configurations.
